# Organocatalytic Enantioselective Addition of 3‑Aryloxindoles to Ethenesulfonyl Fluoride

**DOI:** 10.1021/acs.orglett.5c04026

**Published:** 2025-11-20

**Authors:** Álvaro Castilla-Mocholí, Marc Montesinos-Magraner, Iñaki Fernández-Yagüe, Amparo Sanz-Marco, Carlos Vila, Gonzalo Blay

**Affiliations:** Departament de Química Orgànica, Facultat de Química, Universitat de València, C. Dr. Moliner 50, 46100 Burjassot, Spain

## Abstract

We report the synthesis of chiral sulfonyl fluorides bearing a carbon quaternary stereocenter. A commercially available organocatalyst (DHQD)_2_AQN enables the enantioselective addition of 3-substituted oxindoles to ethenesulfonyl fluoride (ESF), achieving excellent yields (27–99%) and enantioselectivities (89–99% ee) for 3-arylsubstituted oxindoles. This methodology shows high functional group tolerance, as demonstrated by successfully engaging halides, ethers, nitriles, acetals, ketals, or heteroaromatic substituents. The utility of the described products was proven by various synthetic transformations, including SuFEx reactions with complex biomolecules.

## Graphical abstract

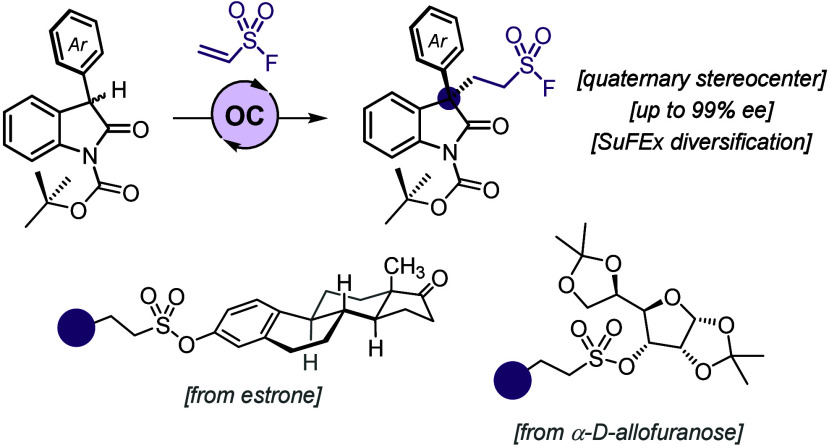

Sulfur (VI) fluoride exchange (SuFEx) reactions, a term coined by Sharpless in 2014,^1^ are a family of “click-reactions” that have emerged as robust and effective strategies for assembling modular intermolecular linkages. On one hand, the S­(VI)–F bond exhibits excellent resistance to hydrolysis under acidic or basic media and thermal stability and can be employed under redox conditions or light irradiation. On the other hand, the S­(VI)–F can be controllably and specifically cleaved under certain reaction conditions. This ideal balance between stability and reactivity has paved the way to an increasing variety of applications of SuFEx “click chemistry”.^2^

Sulfonyl fluorides are arguably the most attractive compounds in this vibrant field, with important applications in organic synthesis,^3a^ medicinal chemistry,^3b–3e^ chemical biology^3f,3g^ and materials science (Figure 1A).^3h–3j^ It is not surprising that the synthesis of sulfonyl fluorides has attracted the attention of the synthetic community over the last years.^4^ In contrast to the synthesis of arylsulfonyl fluorides, methodologies to obtain alkylsulfonyl fluorides are still underdeveloped. In particular, the synthesis of chiral alkyl sulfonyl fluorides is of great importance for the aforementioned applications in medicinal chemistry and chemical biology, in which the chirality of the products is of crucial interest. In this context, ethenesulfonyl fluoride (ESF) has emerged as a powerful reagent due to its superb reactivity as a Michael acceptor.^5^ The first organocatalytic enantioselective reaction with ESF was reported by the group of Yan by reacting 3-amido-2-oxindoles in the presence of a bifunctional quinine-derived squaramide.^6a^ The corresponding tetrasubstituted compounds were obtained with excellent enantioselectivities and could be converted into the corresponding spirocyclic sultams via an intramolecular SuFEx-type reaction (Figure 1B). *N*-2,2,2-Trifluoroethylisatin ketimines were also successfully added to ESF under similar conditions, as reported by the same group in 2021.^6b^ Zhang, Yan and co-workers also developed the addition of azlactones to ESF via cooperative organocatalysis with a chiral Brønsted base and an achiral hydrogen-bond donor. The corresponding products were readily converted into tetrasubstituted α-amino esters and lactones with potential interest in drug discovery.^6c^ The same research group also described the addition of 4-amido-5-hydroxypyrazoles to ESF, giving access to tetrasubstituted pyrazolones with excellent yields and enantioselectivties.^6d^ A few examples using metal catalysis or β-substituted alkenyl sulfonyl fluoride can also be found in the literature.^7^

**Figure 1:**
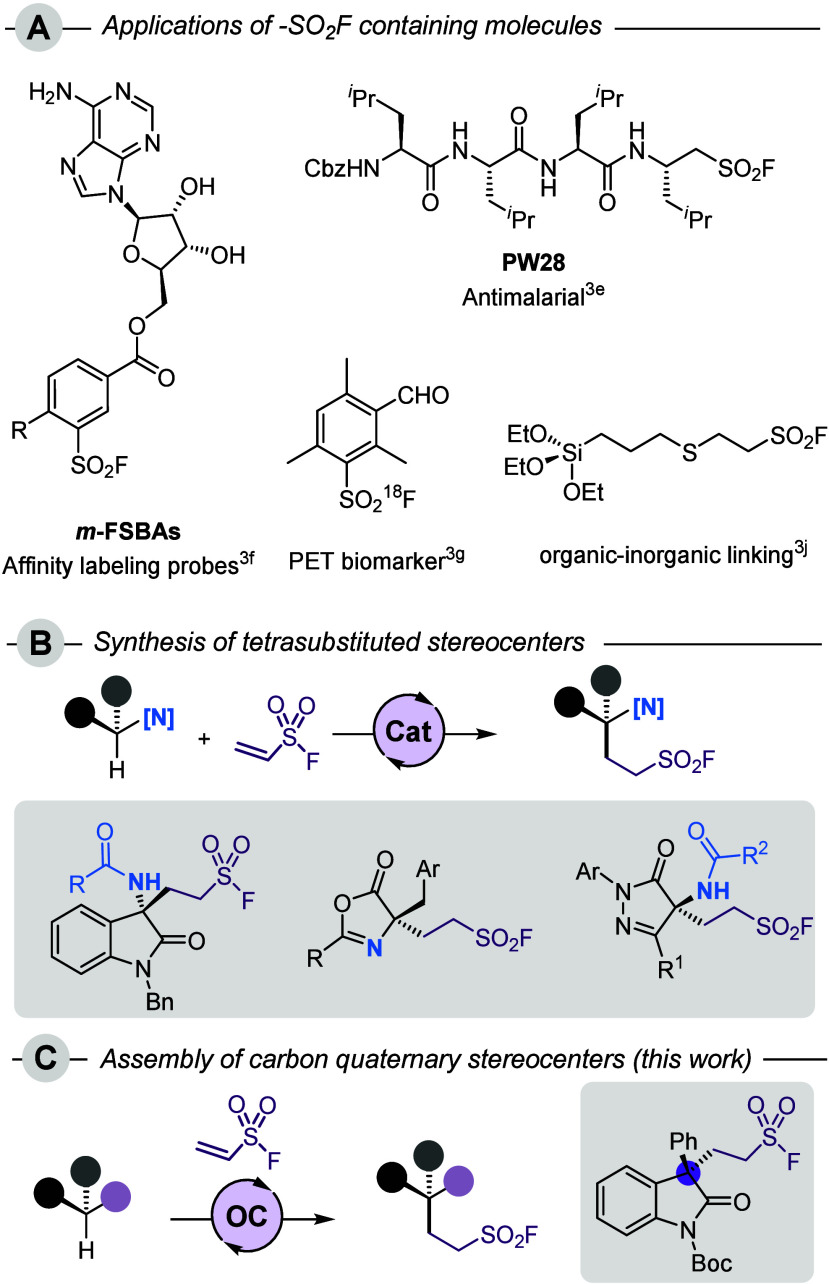
A: Examples of diverse sulfonyl fluorides with interesting applications. B: Existing methods for the enantioselective addition of nucleophiles to ESF. C: Synthesis of quaternary carbon stereocenters by enantioselective addition to ESF (this work).

Despite these interesting findings, the formation of enantioenriched sulfonyl fluorides containing a carbon quaternary stereocenter has not been reported to date (Figure 1C).^8^ For this purpose, we turned our attention to 3-aryl-2-oxindoles, which are known to undergo enantioselective and stereoconvergent C–C and C–X bond formation reactions. Upon treatment with an appropriate chiral base, these heterocycles behave as pro-chiral nucleophiles, which have been employed to prepare asymmetric 3,3-disubstituted oxindoles by alkylation, arylation or Michael addition reactions,^9^ including reactions with ethenyl arylsulfones and arylselenones.^9e–9j^

After an intensive catalyst optimization process for the enantioselective addition of 3-phenyloxindole **1a** to ESF (**2**),^10^ we established commercially available (DHQD)_2_AQN as the most promising organocatalyst. The desired addition product **2a** was obtained in quantitative yield and 75% ee using CH_2_Cl_2_ as the solvent and running the reaction at rt (Table 1, entry 1). Unlike in the reaction reported by Zhang and Yan, in our case the addition of 10 mol % of Schreiner’s thiourea did not lead to an improvement in the efficiency of the reaction (Table 1, entry 2).

**Table 1:**
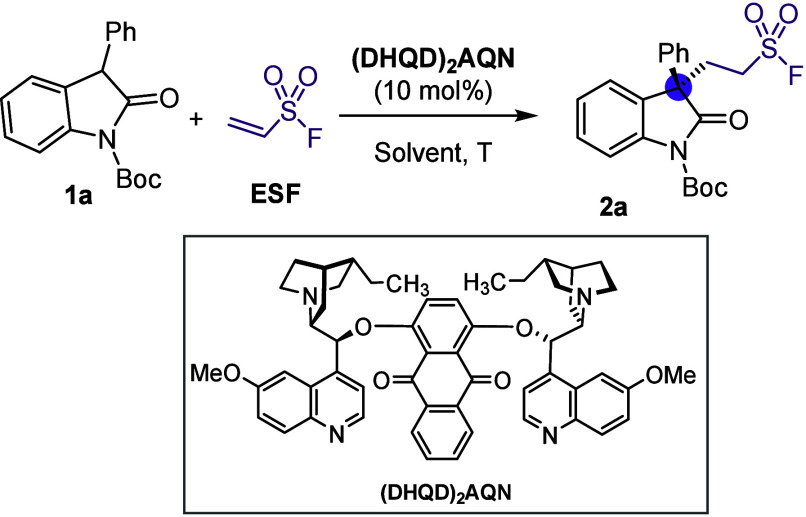
Enantioselective Reaction of 3-Phenyloxindole **1a** with ESF—Optimization Process^a^

Entry	Solvent	*T* (°C)	*t* (h)	Yield (%)^b^	ee (%)^c^
1	CH_2_Cl_2_	25	24	99	75
2^d^	CH_2_Cl_2_	25	24	97	76
3	CH_2_Cl_2_	0	24	99	84
4	CH_2_Cl_2_	–20	24	97	91
5	(CH_2_Cl)_2_	–20	24	97	89
6	Toluene	–20	24	95	88
7	MTBE	–20	24	96	89
8	EtOAc	–20	24	93	88
9	EtOH	–20	24	96	73
10	Et_2_O	–20	24	99	94
11	THF	–20	24	97	93
**12**	**THF**	**–78**	**3**	**97 (95)** ^h^	**97**
13^e^	THF	–78	3	76	–76
14^f^	THF	–78	3	95	95
15^g^	THF	–78	3	nd	-

a Reaction conditions: **1a** (0.1 mmol), ESF (1 equiv), (DHQD)_2_AQN (10 mol %), solvent (1 mL).

b Determined by ^1^H NMR using 1,3,5-trimethoxybenzene as IS.

c Determined by chiral HPLC.

d Reaction carried out in the presence of 10 mol % of Schreiner’s thiourea.

e (DHQ)_2_AQN (10 mol %) was used: negative values indicated the opposite enantiomer.

f (DHQD)_2_AQN (5 mol %) was used.

g No catalyst. nd: not detected.

h Isolated yields in parentheses.

We next studied the effect of temperature on the reaction outcome. Lowering the temperature of the reaction to 0 °C and −20 °C resulted in improved enantioselectivities (84% ee and 91% ee, respectively) (Table 1, entries 3–4). At this point, we investigated the solvent effect at −20 °C. Solvents such as 1,2-dichloroethane, toluene, methyl *tert*-butyl ether, ethyl acetate and ethanol resulted in slightly lower enantioselectivity (Table 1, entries 5–9). Other ethereal solvents, such as diethyl ether and tetrahydrofuran, allowed us to reach 94% and 93% ee, respectively (Table 1, entries 10–11). Of the two solvents, we chose tetrahydrofuran over diethyl ether due to its improved usability properties. Finally, we set up the reaction at −78 °C, and to our delight we obtained the desired product in high yield and with an excellent enantioselectivity (97% ee) (Table 1, entry 12). Unfortunately, pseudoentantiomer (DHQ)_2_AQN proved to be less efficient in terms of enantioinduction (76% ee, Table 1, entry 13). Reduction on the catalyst loading to 5 mol % had a minimal effect on the enantioselectivity, still obtaining the desired product in quantitative yield and 95% ee (Table 1, entry 14). Finally, no conversion was observed in the absence of the chiral catalyst (Table 1, entry 15).

We next studied the scope of the reaction (Scheme 1). First, we studied the effect of the protecting group on the nitrogen atom of oxindole **1**. Both *N*-acetyl- and *N*-benzyl-3-phenyloxindoles were shown to be less effective under the optimized conditions delivering the addition products **2b** and **2c** in lower yields and poor enantioselectivities (41%, 31% ee and 33%, 19% ee, respectively). The optimized reaction conditions were also tested in a 1 mmol scale, obtaining compound **2a** in comparable yield with conserved enantioselectivity (99%, 98% ee). On the other hand, 3-phenyloxindoles with different substituents in position 5 allowed us to obtain the desired sulfonylfluorides **2d**–**2g** in very high yields and excellent enantioselectivities independently of their electronic nature (89–99%, 97–98% ee). Single-crystal X-ray diffraction analysis of the 5-chloro derivative **2e** unambiguously established its absolute configuration as *R*, and the rest of the products were assumed to follow a uniform stereochemical mechanism. A strong electron-donor group such as a MeO– group is also well tolerated in the 6 position (**2h**, 91%, 98% ee) as well as a weak electron-withdrawing Cl (**2i**, 90%, 95% ee). Substitution in position 7 was also well tolerated, as demonstrated by the results obtained for fluorine-containing product **2j** (79%, 97% ee). Doubly substituted oxindole **2k** was employed to test the effect of substitution in the 4 position. As expected, a methyl group adjacent to the forming quaternary stereocenter resulted in a slow reaction with a diminished yield but still delivered very high enantioselectivity for compound **2k** (27%, 90% ee).

**Scheme 1:**
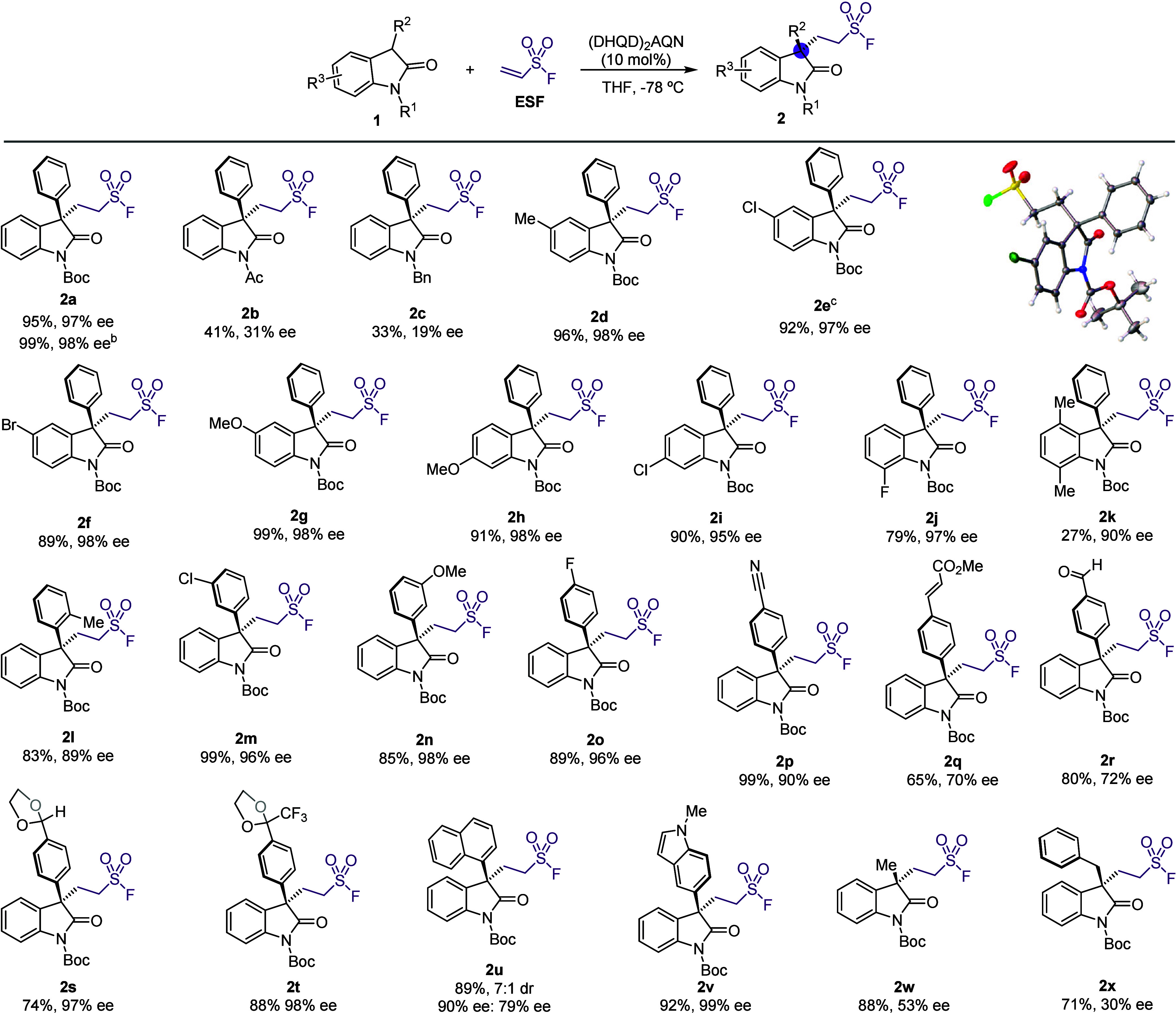
Scope of the Catalytic Enantioselective Reaction between 2-Oxindoles and ESF^a^

a Reaction conditions: **1** (0.1 mmol), **2** (0.1 mmol), (DHQD)_2_AQN (0.01 mmol), THF (1 mL) at −78 °C; yield of isolated product after column chromatography; dr determined by ^1^H NMR; ee determined by chiral HPLC over chiral stationary phase.
b Reaction conditions: **1** (1 mmol), ESF (1 mmol), (DHQD)_2_AQN (0.1 mmol), THF (10 mL) at −78 °C.
c Flack parameter: 0.017(13).

Regarding the scope of the 3-aryl group, we first tested sterically hindered 3-(2-methylphenyl)­oxindole **1l**. Remarkably, the addition product was obtained in good yield and high enantioselectivity (**2l**, 83%, 89% ee). Phenyl rings with substituents in the *meta* or *para* positions were more reactive, as can be expected for their diminished steric clash compared to the *ortho*-substitution. So, *m*-chloro, *m*-methoxy and *p*-fluoro derivatives, **2m**–**2o**, were obtained in high yields and excellent enantioselectivities (99–89%, 96–98% ee). At this point, we turned our attention to more challenging substrates in order to prove the functional group tolerance of our method. For this purpose, we reacted nitrile **1p** under the optimized conditions, obtaining the desired product **2p** in 99% yield and 90% ee. Next, we studied the reactivity of oxindoles bearing a carbonyl group, as evidenced by the obtention of acrylate **2q** and aldehyde **2r**. Both compounds were obtained in moderate to good yields (65% and 80%, respectively), but we observed a similar drop in enantioinduction (70–72% ee). We rationalize this result by assuming additional interactions between the carbonylic oxygen and the chiral catalyst, which cause an erosion in the enantioselectivity of the process. Fortunately, this drawback could be easily circumvented by using cyclic acetals as masking carbonyl groups. In this way, we managed to prepare dioxolane **3s** in high yield recovering the excellent enantioselectivity (74%, 97% ee). Reactive ketones can also be engaged using this approach, delivering CF_3_-containing cyclic acetal **2t** in excellent yield and enantioselectivty (88%, 98% ee). Extended aromatic substituents can also be employed as evidenced by 1-naphthyl substituted **2u**, which was obtained as a 7:1 mixture of atropisomers in a high yield. In this case, the major stereoisomer was obtained with high enantioselectivity, while the minor one was isolated with slightly decreased enantioselectivity (90% and 79% ee, respectively). Heteroaromatics are also compatible with our protocol. Indole **2v** was obtained with perfect selectivity, even in the presence of a highly nucleophilic *N*-methylindole moiety (92%, 99% ee). With these excellent results in hand, we turned our attention to 3-alkyloxindoles as substrates, which was shown to be more challenging. 3-Methyloxindole **2w** was obtained still in high yield (88%), but the enantioselectivty dropped down to 53% ee. This diminished enantioselectivity (30% ee) was also observed for benzyl-substituted product **2x**. Oxindoles bearing cyclic or branched substituents at C3 reacted slowly giving the expected products in very low yields. On the other hand, C3-unsubstituted *N*-Boc-oxindole gave the double addition product.^10^

Alkylsulfonyl fluoride **2a** was then tested in different SuFEx-type reactions in order to prove their synthetic potential (Scheme 2). By treating **2a** with morpholine in the presence of Et_3_N, formation of tertiary sulfonamide **3** occurred in high yield.^12^ Under these mild reaction conditions, concomitant *N*-Boc deprotection took place, delivering the corresponding free NH oxindole. Sulfonates, such as sesamol-derived **4**, can also be prepared in excellent yields, using Cs_2_CO_3_ as the base.^12^ Complexity of the nucleophiles can be increased, as demonstrated by the quantitative formation of compound **5**, which is formed by the addition of the natural steroid estrone. Moreover, highly hindered secondary alcohols, such as di-*O*-isopropylidene-α-d-allofuranose, can form the desired sulfonates. In this case, we used a strategic TMS-protected sugar, which facilitated the otherwise sluggish SuFEx reaction by means of the formation of thermodynamically stable TMS-F. The desired product **6** was formed with excellent selectivity in 76% yield.^13^ Finally, the orthogonal reactivity of the −SO_2_F functional group was proven by the engagement of compound **2f** in a Suzuki–Miyaura cross-coupling reaction. Biaryl **7** was obtained in 49% yield under nonoptimized reaction conditions.^14^

**Scheme 2:**
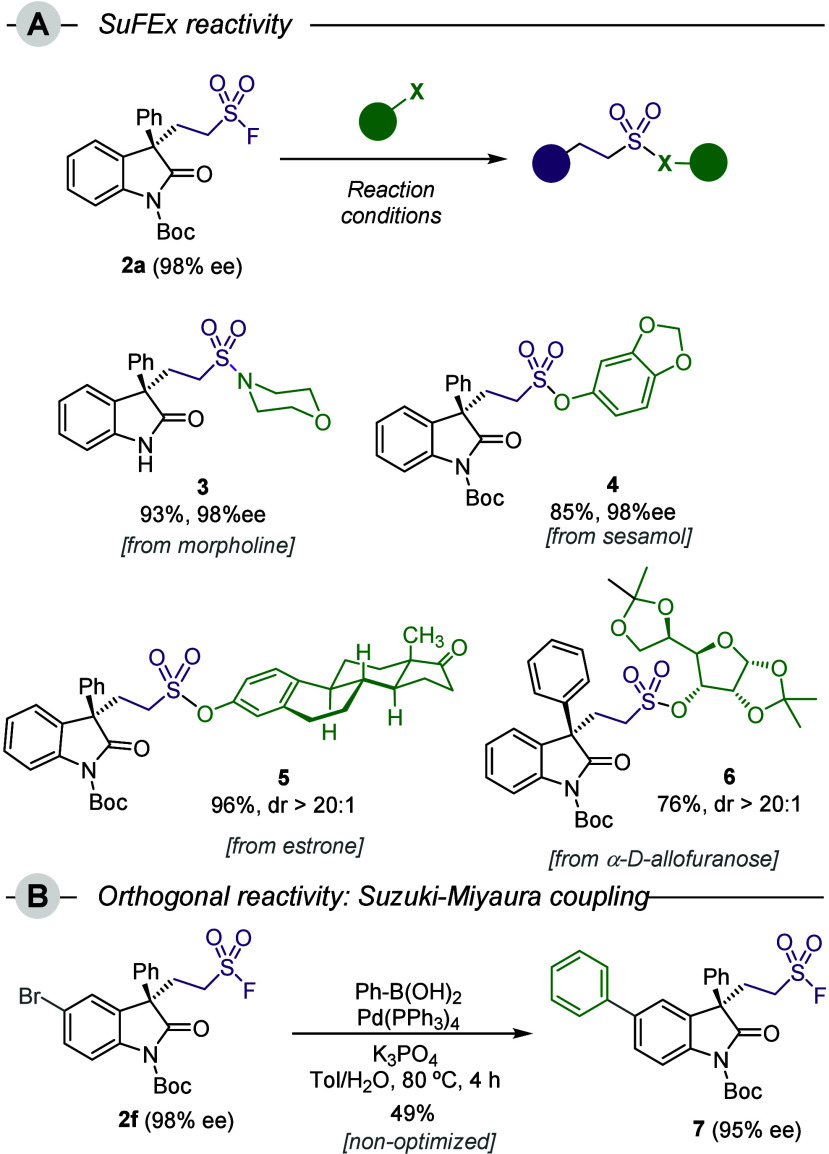
Synthetic Applications^a^

a For the reaction conditions, see the Supporting Information.

In conclusion, we have developed the enantioselective addition of 3-aryl-2-oxindoles to ESF catalyzed by commercially available (DHQD)_2_AQN. The corresponding alkyl sulfonyl fluorides were obtained in high yields and with excellent enantioselectivities in most cases. The *N*-Boc group on the oxindole proved to be crucial for both reactivity and selectivity, and the method exhibited good tolerance toward functional groups, such as halides, ethers, nitriles, acetals, ketals, and heteroaromatic substituents. In contrast, replacing the aryl substituent at the C-3 position of the oxindole with alkyl groups led to decreased reactivity and enantioselectivity. Further synthetic utility of the products was demonstrated through SuFEx reactions with biologically relevant nucleophiles. Finally, the orthogonality of the −SO_2_F group was highlighted by the successful Suzuki–Miyaura coupling of bromide **2f** with PhB­(OH)_2_.

## Supplementary Material



## Data Availability

The data underlying this study are available in the published article and in its Supporting Information and are also openly available in DRYAD at 10.5061/dryad.xpnvx0ktz

## Notes

Experimental procedures, characterization data, NMR spectra and HPLC traces (PDF)
