# Supplementary material for: Organocatalytic Enantioselective Addition of 3‑Aryloxindoles to Ethenesulfonyl Fluoride
Source: Org Lett. 2025 Nov 20;27(48):13211–6. doi: 10.1021/acs.orglett.5c04026 (PMC12687310; doi:10.1021/acs.orglett.5c04026)
Supplement: Supplementary file 1 [file ol5c04026_si_001.pdf]

Supporting Information

Organocatalytic Enantioselective Addition of 3-Aryloxindoles to Ethenesulfonyl Fluoride

Álvaro Castilla-Mocholí, Marc Montesinos-Magraner, Iñaki Fernández-Yagüe, Amparo Sanz-Marco, Carlos Vila, and Gonzalo Blay*

Departament de Química Orgànica, Facultat de Química, Universitat de València, C. Dr. Moliner 50, 46100-Burjassot, Spain

*E-mail: gonzalo.blay@uv.es.

Table of contents

Experimental Methods 2

Optimization 3

Catalyst Optimization 3

Optimization of the reaction conditions 4

General procedures and characterization data for compounds 1a-1z 5

General procedure 1.a[1,2] 5

General procedure 1.b[2] 10

General procedure 1.c[2] 15

General procedure 2[4,22] 17

Synthesis of carbonyl-containing 3-aryloxindoles 20

Synthesis of aliphatic 3-substituted oxindoles 22

General procedures and characterization for products 2a-2x 25

Unsuccessful Substrates 36

1 mmol-scale synthesis and transformations of compounds 2 38

X-ray structure, crystal data and structure refinement for compound 2e 43

NMR spectra of synthesized compounds 45

HPLC Analysis 136

References 164

Experimental Methods

Unless it is stated otherwise, reactants were obtained from commercial sources and used directly without further purification. Ethenesulfonyl fluoride CAS: 677-25-8) and $(DHQD)_2AQN$ (CAS: 176298-44-5) were purchased from Merck. Reactions were monitored with Merck 60 F254 (reference 5554 Merk) silica gel plates. The eluted TLC plates were visualized under 254 nm UV light and revealed with stain solutions of cerium molybdate or potassium permanganate. Flash column chromatography was carried out using a Silica Gel Merck 60 stationary phase (reference 109385 Merck), with a 0.040-0.063 mm particle range size. The eluent was made to flow through the column with an air pump. NMR spectra were recorded using a Bruker Avance III 300, Bruker AV400, or Bruker Neo500 instruments operating at 300, 400, or 500 MHz for ^{1}H and at 75, 101, or 125 MHz for ^{13}C, respectively. ^{19}F spectra were measured at 282 MHz (Avance III 300) or 471 MHz (Neo500). Signals of non-deuterated residual solvent were used as internal standard (7.26 ppm for ^{1} H and 77.16 ppm for ^{13}C in chloroform; and 4.87 ppm for ^{1}H and 49.00 ppm for ^{13}C in CD_3OD). Chemical shifts (δ) are expressed in ppm and coupling constants (J), in Hz. HRMS were recorded using a Waters Q-TOF spectrometer equipped with an electrospray source with a capillary voltage of 3.3 kV (ESI). Specific optical rotations were measured using a Bellingham+Stanley ADP430 polarimeter equipped with a LED light source, measuring at the sodium wavelength (D line, 589 nm) and a 1 dm path length cuvette. Concentrations are expressed in g/100 mL. Enantiomeric excesses were measured through HPLC analysis, using a Hitachi Elite Lachrom chromatograph with a Hitachi L-4500 or L-2455U UV diode array detectors. Daicel or Phenomenex columns with chiral stationary phases were employed, and the samples were eluted with mixtures of HPLC-grade hexane and isopropyl alcohol.

Optimization

Catalyst Optimization

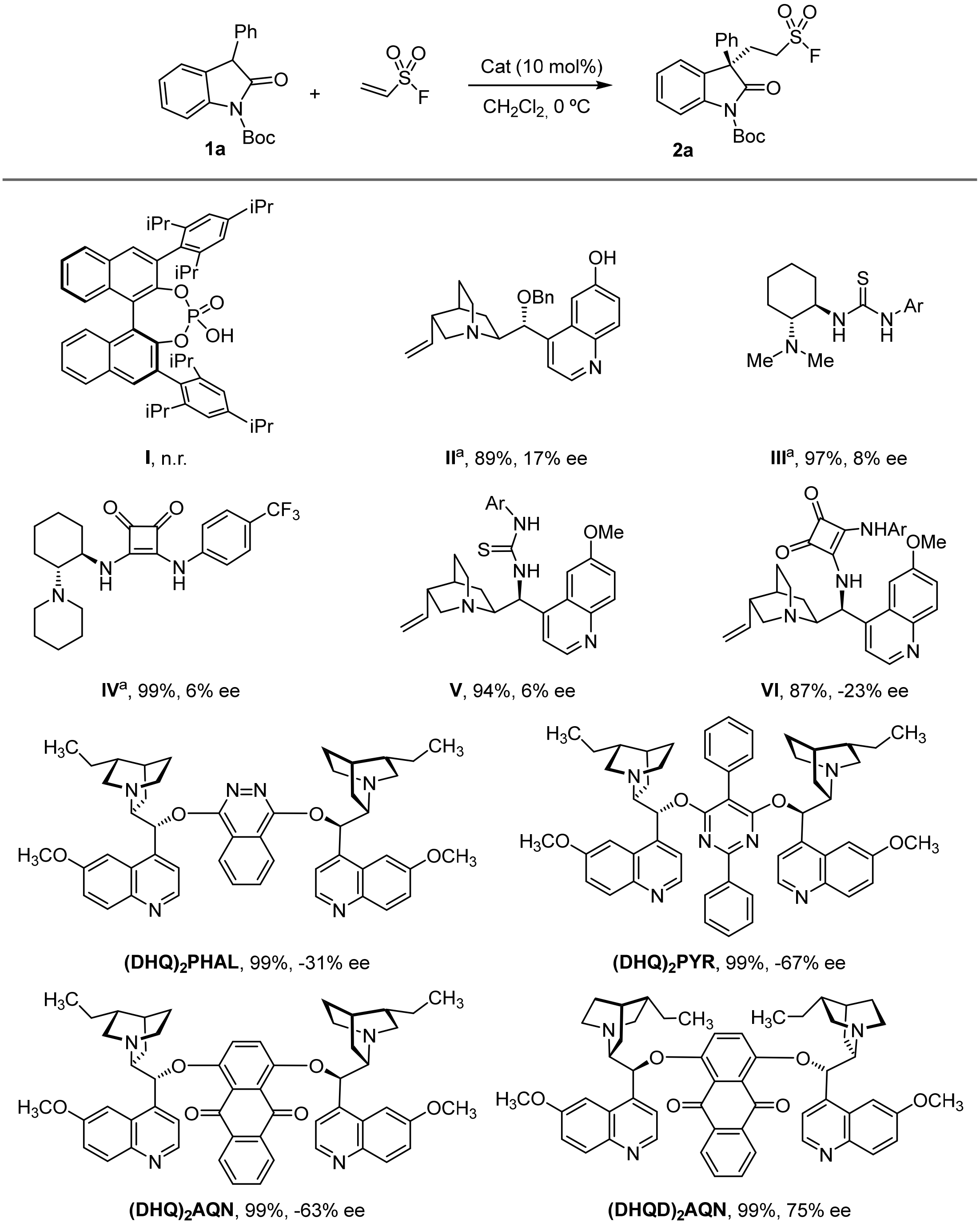

Figure S1. Enantioselective reaction between 3-phenyloxindole **1a** with ESF**,** catalyst study. Reaction conditions: **1a** (0.1 mmol), ESF (1 equiv.), **Catalyst** (10 mol%), CH_2Cl_2 (1 mL), rt. Yield was determined by ^{1}H-NMR using 1,3,5-trimethoxybenzene as IS. Enantiomeric excess was determined by chiral HPLC. Negative ee values indicate the formation of the opposite enantiomer.[a] Reaction performed at 0 ºC.

We started our catalyst optimization process studying the addition *N*-Boc-3-phenyloxindole (**1a**) to ethenesulfonyl fluoride (ESF) in CH_2Cl_2 at room temperature using different organocatalysts (Scheme 1). A strong Brønsted acid such as (*S*)-TRIP (**I**) demonstrated unable to catalyzed the desired reaction. Using benzyl-substituted cupreine **II** allowed us to obtain the desired addition

product 2**a** in 89% yield and with a modest enantioselectivity (17% ee) after 48 hours. Common commercially available bifunctional organocatalysts, such as Takemoto's thiourea (**III**) and Rawal's squaramide (**IV**) delivered better reactivity, but virtually racemic product (97%, 8% ee and 99%, 6% ee, respectively). Similarly, quinine-derived thiourea **V** and squaramide **VI** provided again very good reactivity but low enantioinduction (94%, 6% ee and 87%, -23% ee, respectively). We turned then our attention to a different family of cinchona alkaloids derivatives, such as those used in the renowned Sharpless asymmetric dihydroxylation. Although $(DHQ)_2$PHAL did not offered a significant improvement (96%, -31%ee), $(DHQ)_2$PYR and $(DHQ)_2$AQN showed an increased selectivity, yielding quantitative yields with promising enantioselectivities (67% and 63% ee, respectively). Finally, we tested the quinidine-derived $(DHQD)_2$AQN, which afforded the desired product in quantitative yield and 75% ee.

Optimization of the reaction conditions

We next evaluated various reaction conditions employing the $(DHQD)_2$AQN as the catalyst. At this stage, monitoring the reaction progress by TLC proved challenging, as the product and the starting material **1a** exhibited very similar Rf values. Therefore, all reactions were allowed to proceed for 24 hours to enable a more reliable comparison of reaction outcomes. A more detailed examination of the reaction conducted in THF revealed that complete conversion was achieved within 3 hours.

Table S1. Optimization of the reaction conditions

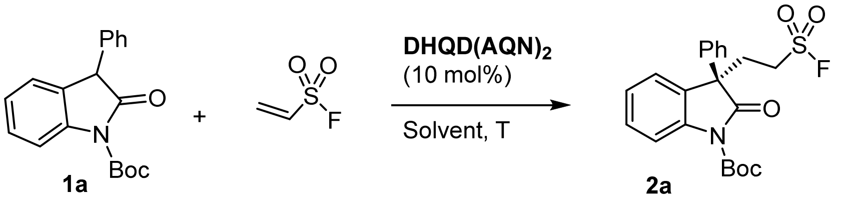

Entry	Solvent	T. (ºC)	t (h)	Yield (%)[b]	ee (%)[c]
1	CH_2Cl_2	25	24	99	75
2[d]	CH_2Cl_2	25	24	97	76
3	CH_2Cl_2	0	24	99	84
4	CH_2Cl_2	-20	24	97	91
5	$(CH_2Cl)_2$	-20	24	97	89
6	Toluene	-20	24	95	88
7	MTBE	-20	24	96	89
8	EtOAc	-20	24	93	88
9	EtOH	-20	24	96	73
10	Et_2O	-20	24	99	94
11	THF	-20	24	97	93
12	THF	-78	3	97 (95)[h]	97
13[e]	THF	-78	3	76	-76
14[f]	THF	-78	3	95	95
15[g]	THF	-78	3	nd	-

[a] Reaction conditions: **1a** (0.1 mmol), **ESF** (1 equiv.), **$(DHQD)_2$AQN** (10 mol%), solvent (1 mL).[b] Determined by ^{1}H-NMR using 1,3,5-trimethoxybenzene as IS.[c] Determined by chiral HPLC.[d] Reaction carried out in the presence of 10 mol% of Schreiner's thiourea. [e] **$(DHQ)_2$AQN** (10 mol%) was used: negative values indicated the opposite enantiomer. [f] **$(DHQD)_2$AQN** (5 mol%) was used. [g] No catalyst. nd: not detected. [h] Isolated yields in parenthesis.

General procedures and characterization data for compounds 1a-1z

General procedure 1.a[1,2]

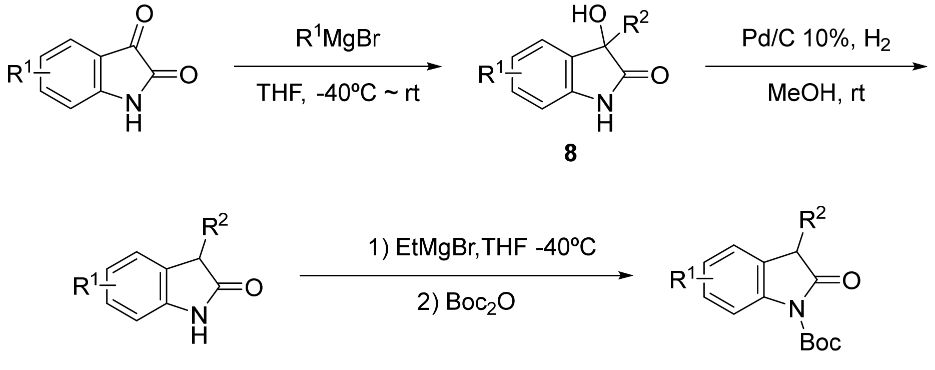

8

9

1

A septum-sealed flask equipped with a stirring bar was charged with the corresponding isatin (1.0 equiv.) under nitrogen atmosphere. Dry THF (15 mL) was added, and the resulting solution was cooled to -40 °C. A solution of R^1MgBr (3 M in Et_2O, 2.0 equiv.) was then added dropwise, and the reaction mixture was stirred at room temperature overnight. The reaction was cooled to 0 ºC, diluted with Et_2O (25 mL) and quenched with HCl 1 M (15 mL). The aqueous layer was extracted with Et_2O (3 x 25mL), and the combined organic phases were washed with water, brine and dried over $MgSO_4$. Solvents were removed *in vacuo* and the crude product was purified by silica gel column chromatography using DCM:EtOAc mixtures as eluent to yield the desired 3-substituted 3-hydroxyl-2-oxindole.

8 (1.0 equiv.) was dissolved in MeOH (30 mL), and 10% Pd/C (10 mol%) was added. The suspension was stirred overnight under hydrogen atmosphere (balloon) at room temperature. The mixture was filtered through Celite®, concentrated *in vacuo*, and the resulting residue was purified by silica gel column chromatography using hexane:EtOAc mixtures as eluent to give the corresponding product.

9 (1.0 equiv.) was dissolved in dry THF (7.5 mL) under nitrogen atmosphere and cooled to –40 °C. EtMgBr (3 M in Et_2O, 1.2 equiv.) was added dropwise, and the mixture was stirred for 30 min. Subsequently, Boc_2O (1.5 equiv.) was added, and the reaction was allowed to stir overnight at room temperature. The mixture was diluted with EtOAc (50 mL), quenched with saturated NH_4Cl (50 mL), and extracted with EtOAc (3 x 50 mL). The combined organic layers were washed with brine, dried over $MgSO_4$, and concentrated in vacuo. The crude residue was purified by silica gel column chromatography using hexane:EtOAc mixtures as eluent to afford the desired *N*-Boc protected 3-substituted oxindole.

3-hydroxy-3-phenylindolin-2-one (8a)[1]

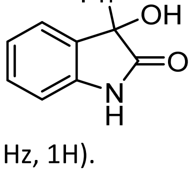

General procedure was followed using isatin (3.4 mmol, 500 mg, 1 equiv.) and phenylmagnesium bromide (3M in Et_2O, 6.8 mmol, 2.3 mL, 2 equiv.). Orange solid, 597 mg, 78% yield. **^{1}H NMR** (300 MHz, CD_3OD) δ 7.42 – 7.24 (m, 6H), 7.17 (dd, *J* = 7.6, 1.4 Hz, 1H), 7.04 (td, *J* = 7.5, 1.1 Hz, 1H), 6.97 (dt, *J* = 7.8, 0.9 Hz, 1H).

3-phenylindolin-2-one (9a)[3]

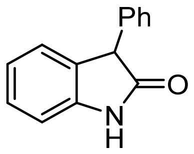

General procedure was followed using 3-hydroxy-3-phenylindolin-2-one (2.4 mmol, 304 mg, 1 equiv.) White solid, 377 mg, 75% yield. **^{1}H NMR** (300 MHz, $CDCl_3$) δ 8.85 (bs, 1H), 7.41 – 7.25 (m, 3H), 7.30 – 7.17 (m, 3H), 7.18 – 7.08 (m, 1H), 7.03 (td, *J* = 7.5, 1.1 Hz, 1H), 6.94 (dd, *J* = 7.7, 1.0 Hz, 1H), 4.64 (s, 1H).

tert-butyl 2-oxo-3-phenylindoline-1-carboxylate (1a)[1,2,4]

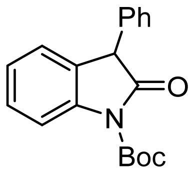

General procedure was followed using 3-phenylindolin-2-one (1.4 mmol, 200 mg, 1 equiv.). White solid, 300 mg, 70% yield. **^{1}H NMR** (300 MHz, $CDCl_3$) δ 7.96 – 7.90 (m, 1H), 7.41 – 7.29 (m, 4H), 7.22 – 7.14 (m, 4H), 4.73 (s, 1H), 1.63 (s, 9H). **^{13}C NMR** (75 MHz, $CDCl_3$) δ 174.1 (C), 149.5 (C), 140.6 (C), 136.4 (C), 129.1 (CH), 128.8 (CH), 128.0 (CH), 127.6 (C), 125.2 (CH), 124.7 (CH), 115.3 (CH), 84.6 (C), 52.7 (CH), 28.2 (CH_3). Signal at 128.8 corresponds to 2 overlapping aromatic CH singlets.

1-acetyl-3-phenylindolin-2-one (1b)[5]

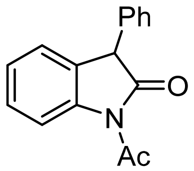

General procedure was followed using 3-phenylindolin-2-one (1.4 mmol, 290 mg, 1 equiv.) and Ac_2O (2.2 mmol, 1.1 mL, 1.5 equiv.) instead of Boc_2O. White solid, 91 mg, 26% yield. **^{1}H NMR** (300 MHz, $CDCl_3$) δ 8.31 (ddt, *J* = 8.2, 1.1, 0.6 Hz, 1H), 7.43 – 7.31 (m, 4H), 7.23 – 7.16 (m, 4H), 4.79 (d, *J* = 0.5 Hz, 1H), 2.66 (s, 3H). **^{13}C NMR** (75 MHz, $CDCl_3$) δ 176.6 (C), 171.2 (C), 141.0 (C), 136.3 (C), 129.2 (CH), 129.0 (CH), 128.7 (CH), 128.3 (CH), 127.8 (C), 125.6 (CH), 125.1 (CH), 116.9 (CH), 52.8 (CH), 26.8 (CH_3).

3-hydroxy-5-methyl-3-phenylindolin-2-one (8d)[6,7]

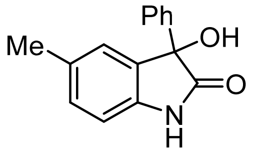

General procedure was followed using 5-methylisatin (3.1 mmol, 500 mg, 1 equiv.) and phenylmagnesium bromide (3M in Et_2O, 6.2 mmol, 2.1 mL, 2 equiv.). Orange solid, 477 mg, 64% yield.**^{1}H NMR** (300 MHz, CD_3OD) δ 7.40 – 7.34 (m, 2H), 7.34 – 7.22 (m, 3H), 7.10 (ddd, *J* = 7.9, 1.7, 0.7 Hz, 01), 7.01 – 6.94 (m, 1H), 6.85 (d, *J* = 7.9 Hz, 1H), 2.27 (s, 3H).

5-methyl-3-phenylindolin-2-one (9d)[8]

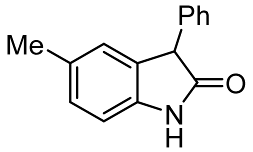

General procedure was followed using 3-hydroxy-5-methyl-3-phenylindolin-2-one (2.0 mmol, 477 mg, 1 equiv.). White solid, 296 mg, 67% yield. **^{1}H NMR** (300 MHz, CD_3OD) δ 7.40 – 7.21 (m, 3H), 7.21 – 7.10 (m, 2H), 7.06 (ddt, *J* = 7.8, 1.6, 0.8 Hz, 1H), 6.91 – 6.80 (m, 2H), 4.62 (s, 1H), 2.25 (s, 3H).

***tert*-butyl 5-methyl-2-oxo-3-phenylindoline-1-carboxylate (1d)**[2]

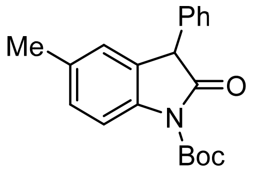

General procedure was followed using 5-methyl-3-phenylindolin-2-one (1.3 mmol, 296 mg, 1 equiv.). White solid, 250 mg, 60% yield. **^{1}H NMR** (300 MHz, $CDCl_3$) δ 7.80 (d, *J* = 8.3 Hz, 1H), 7.39 – 7.28 (m, 3H), 7.22 – 7.13 (m, 3H), 6.98 – 6.93 (m, 1H), 4.69 (s, 1H), 2.32 (s, 3H), 1.63 (s, 9H). **^{13}C NMR** (75 MHz, $CDCl_3$) δ 174.3 (C), 149.6 (C), 138.2 (C), 136.6 (C), 134.4 (C), 129.2 (CH), 129.0 (CH), 128.8 (CH), 128.0 (CH), 127.6 (C), 125.7 (CH), 115.0 (CH), 84.4 (C), 52.8 (CH), 28.2 (CH_3), 21.2 (CH_3).

3-hydroxy-6-methoxy-3-phenylindolin-2-one (8h)[9]

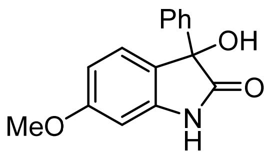

General procedure was followed using 6-methoxyisatin (2.8 mmol, 500 mg, 1 equiv.) and phenylmagnesium bromide (3M in Et_2O, 6.2 mmol, 2.1 mL, 2 equiv.). Orange solid, 320 mg, 44% yield. **^{1}H NMR** (300 MHz, CD_3OD) δ 7.40 – 7.34 (m, 2H), 7.33 – 7.22 (m, 3H), 7.10 – 7.03 (m, 1H), 6.62 – 6.54 (m, 2H), 3.80 (s, 3H).

6-methoxy-3-phenylindolin-2-one (9h)[8]

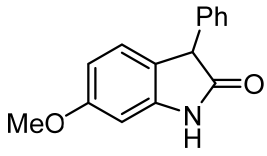

General procedure was followed using 3-hydroxy-6-methoxy-3-phenylindolin-2-one (1.3 mmol, 320 mg, 1 equiv.). Yellow solid, 160 mg, 54% yield. **^{1}H NMR** (300 MHz, $CDCl_3$) δ 8.60 (bs, 1H), 7.40 – 7.28 (m, 3H), 7.25 – 7.18 (m, 2H), 7.01 (d, *J* = 8.1 Hz, 1H), 6.57 (d, *J* = 2.3 Hz, 1H), 6.54 (s, 1H), 4.58 (s, 1H), 3.80 (s, 3H).

***tert*-butyl 6-methoxy-2-oxo-3-phenylindoline-1-carboxylate (1h)**[5]

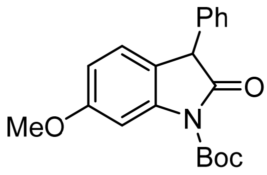

General procedure was followed using 6-methoxy-3-phenylindolin-2-one (0.7 mmol, 160 mg, 1 equiv.). Yellow solid, 114 mg, 48% yield. **^{1}H NMR** (300 MHz, $CDCl_3$) δ 7.58 (d, *J* = 2.4 Hz, 1H), 7.38 – 7.26 (m, 3H), 7.22 – 7.16 (m, 2H), 7.05 (dd, *J* = 8.3, 1.0 Hz, 1H), 6.71 (dd, *J* = 8.3, 2.4 Hz, 1H), 4.67 (s, 1H), 3.86 (s, 3H), 1.63 (s, 9H). **^{13}C NMR** (75 MHz, $CDCl_3$) δ 174.6 (C), 160.2 (C), 149.5 (C), 141.6 (C), 136.8 (C), 129.0 (CH), 128.7 (CH), 127.9 (CH), 125.7 (CH), 119.2 (C), 110.5 (CH), 101.9 (CH), 84.5 (C), 55.7 (CH_3), 52.2 (CH), 28.2 (CH_3).

7-fluoro-3-hydroxy-3-phenylindolin-2-one (8j)[5]

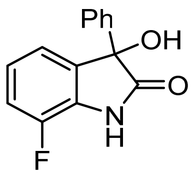

General procedure was followed using 7-fluoroisatin (3.0 mmol, 500 mg, 1 equiv.) and phenylmagnesium bromide (3M in Et_2O, 6.0 mmol, 2.0 mL, 2 equiv.). Orange solid, 430 mg, 60% yield. **^{1}H NMR** (300 MHz, CD_3OD) δ 7.49 – 7.23 (m, 5H), 7.19 – 6.86 (m, 3H).

7-fluoro-3-phenylindolin-2-one (9j)[5,9]

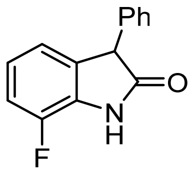

General procedure was followed using 7-fluoro-3-hydroxy-3-phenylindolin-2-one (1.8 mmol, 430 mg, 1 equiv.). White solid, 270 mg, 66% yield. **^{1}H NMR** (300 MHz, $CDCl_3$) δ 8.48 (bs, 1H), 7.40 – 7.27 (m, 3H), 7.24 – 7.18 (m, 2H), 7.08 – 6.89 (m, 3H), 4.68 (s, 1H).

***tert*-butyl 7-fluoro-2-oxo-3-phenylindoline-1-carboxylate (1j)[5]**

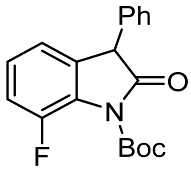

General procedure was followed using 7-fluoro-3-phenylindolin-2-one (1.1 mmol, 270 mg, 1 equiv.). White solid, 229 mg, 59% yield. **^{1}H NMR** (300 MHz, $CDCl_3$) δ 7.40 – 7.29 (m, 3H), 7.23 – 7.17 (m, 2H), 7.15 – 7.06 (m, 2H), 6.99 – 6.92 (m, 1H), 4.77 (s, 1H), 1.61 (s, 9H). **^{13}C NMR** (75 MHz, $CDCl_3$) δ 173.3 (C), 148.6 (d, *J* = 251.2 Hz, C), 147,7 (C), 135.6 (C), 130.7 (d, *J* = 2.1 Hz, C), 129.0 (CH), 128.6 (CH), 128.1 (CH), 127.35 (d, *J* = 9.5 Hz, C), 125.6 (d, *J* = 7.0 Hz, CH) 120.9 (d, *J* = 3.6 Hz, CH), 116.9 (d, *J* = 20.5 Hz, CH), 85.1 (C), 52.8 (d, *J* = 1.8 Hz, CH), 27.7 (CH_3). **^{19}F NMR** (282 MHz, $CDCl_3$) δ -120.2.

3-hydroxy-3-(o-tolyl)indolin-2-one (8l)[7,10]

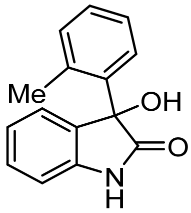

General procedure was followed using isatin (3.4 mmol, 500 mg, 1 equiv.) and 2-methylphenylmagnesium bromide (2M in Et_2O, 6.0 mmol, 3.4 mL, 2 equiv.). Orange solid, 445 mg, 55% yield. **^{1}H NMR** (300 MHz, CD_3OD) δ 8.01 (dd, *J* = 7.8, 1.2 Hz, 1H), 7.35 – 7.25 (m, 2H), 7.20 (td, *J* = 7.4, 1.4 Hz, 1H), 7.05 (d, *J* = 7.4 Hz, 1H), 7.01 – 6.87 (m, 3H), 1.85 (s, 3H).

3-(o-tolyl)indolin-2-one (9l)[11]

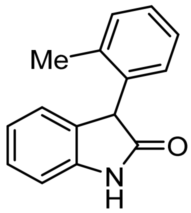

General procedure was followed using 3-hydroxy-3-(o-tolyl)indolin-2-one (1.9 mmol, 445 mg, 1 equiv.). White solid, 310 mg, 73% yield. **^{1}H NMR** (300 MHz, $CDCl_3$) δ 9.15 (bs, 1H), 7.33 – 7.09 (m, 4H), 7.07 – 6.82 (m, 4H), 4.87 (bs, 1H), 2.36 (bs, 3H).

***tert*-butyl 2-oxo-3-(o-tolyl)indoline-1-carboxylate (1l)[12]**

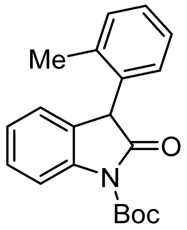

General procedure was followed using 3-(*o*-tolyl)indolin-2-one (1.4 mmol, 310 mg, 1 equiv.). White solid, 287 mg, 64% yield. **^{1}H NMR** (300 MHz, $CDCl_3$) δ 7.96 – 7.88 (m, 1H), 7.35 (dddd, *J* = 8.3, 7.4, 1.5, 0.9 Hz, 1H), 7.25 – 7.16 (m, 2H), 7.15 – 7.01 (m, 3H), 6.87 (bs, 1H), 4.99 (s, 1H), 2.40 (bs, 3H), 1.64 (s, 9H). **^{13}C NMR** (75 MHz, $CDCl_3$) δ 174.1 (C), 149.6 (C), 140.5 (C), 137.5 (C), 135.3 (C), 131.2 (CH), 128.6 (CH), 128.1 (C), 128.1 (CH), 126.5 (CH), 124.8 (CH), 124.7 (CH), 115.2 (CH), 84.5 (C), 50.5 (bs, CH), 28.2 (CH_3), 20.0 (CH_3). A ^{13}C NMR aromatic CH signal was not detected, presumably due to the presence of rotamers.

3-hydroxy-3-(3-methoxyphenyl)indolin-2-one (8n)[11]

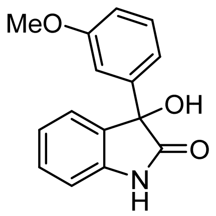

General procedure was followed using isatin (3.4 mmol, 500 mg, 1 equiv.) and 3-methoxyphenylmagnesium bromide (1M in Et_2O, 6.0 mmol, 6.8 mL, 2 equiv.). Orange solid, 355 mg, 66% yield. **^{1}H NMR** (300 MHz, CD_3OD) δ 7.61 – 7.51 (m, 1H), 7.29 (td, *J* = 7.7, 1.4 Hz, 1H), 7.22 – 7.14 (m, 2H), 7.13 – 6.91 (m, 3H), 6.89 – 6.76 (m, 1H), 3.77 (s, 3H).

3-(3-methoxyphenyl)indolin-2-one (9n)[11]

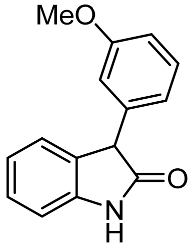

General procedure was followed using 3-hydroxy-3-(3-methoxyphenyl)indolin-2-one (1.8 mmol, 355 mg, 1 equiv.). White solid, 336 mg, 78% yield. **^{1}H NMR** (300 MHz, $CDCl_3$) δ 8.98 (bs, 1H), 7.32 – 7.22 (m, 2H), 7.15 (d, *J* = 7.4 Hz, 1H), 7.04 (td, *J* = 7.5, 0.9 Hz, 1H), 6.95 (d, *J* = 7.8 Hz, 1H), 6.89 – 6.81 (m, 2H), 6.80 – 6.76 (m, 1H), 4.63 (s, 3H), 3.79 (s, 3H).

***tert*-butyl 3-(3-methoxyphenyl)-2-oxoindoline-1-carboxylate (1n)**[13]

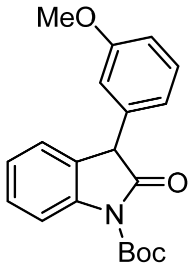

General procedure was followed using 3-(3-methoxyphenyl)indolin-2-one (1.4 mmol, 336 mg, 1 equiv.). White solid, 380 mg, 81% yield. **^{1}H NMR** (300 MHz, $CDCl_3$) δ 7.92 (d, *J* = 8.1 Hz, 1H), 7.42 – 7.30 (m, 1H), 7.31 – 7.20 (m, 1H), 7.21 – 7.13 (m, 2H), 6.84 (ddd, *J* = 8.3, 2.5, 0.8 Hz, 1H), 6.78 (d, *J* = 7.6 Hz, 1H), 6.77 – 6.69 (m, 1H), 4.69 (s, 1H), 3.77 (s, 3H), 1.63 (s, 9H). **^{13}C NMR** (75 MHz, $CDCl_3$) δ 173.8 (C), 159.9 (C), 149.4 (C), 140.5 (C), 137.7 (C), 129.9 (CH), 128.7 (CH), 127.3 (C), 125.1 (CH), 124.6 (CH), 121.0 (CH), 115.1 (CH), 114.6 (CH), 113.2 (CH), 84.4 (C), 55.3 (CH_3), 52.5 (CH), 28.1 (CH_3).

3-(4-fluorophenyl)-3-hydroxyindolin-2-one (8o)[11]

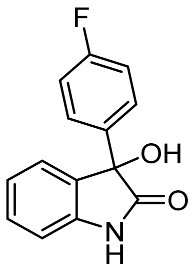

General procedure was followed using isatin (3.4 mmol, 500 mg, 1 equiv.) and 4-fluorophenylmagnesium bromide (1M in Et_2O, 6.0 mmol, 6.8 mL, 2 equiv.). Orange solid, 640 mg, 76% yield. **^{1}H NMR** (300 MHz, MeOD) δ 7.44 – 7.35 (m, 2H), 7.30 (td, *J* = 7.7, 1.3 Hz, 1H), 7.18 (ddd, *J* = 7.4, 1.3, 0.5 Hz, 1H), 7.10 – 7.00 (m, 3H), 7.00 – 6.93 (m, 1H).

3-(4-fluorophenyl)indolin-2-one (9o)[11]

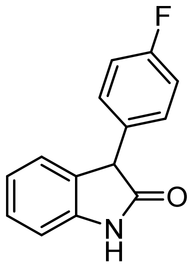

General procedure was followed using 3-(4-fluorophenyl)-3-hydroxyindolin-2-one (2.6 mmol, 640 mg, 1 equiv.). White solid, 455 mg, 74% yield. **^{1}H NMR** (300 MHz, $CDCl_3$) δ 8.83 (bs, 1H), 7.29 – 7.16 (m, 3H), 7.12 (d, *J* = 7.3 Hz, 1H), 7.08 – 6.98 (m, 3H), 6.94 (d, *J* = 7.8 Hz, 1H), 4.62 (s, 1H).

***tert*-butyl 3-(4-fluorophenyl)-2-oxoindoline-1-carboxylate (1o)**[1]

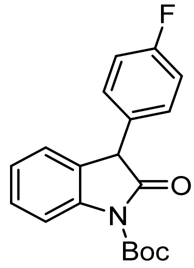

General procedure was followed using 3-(4-fluorophenyl)indolin-2-one (1.9 mmol, 455 mg, 1 equiv.). White solid, 320 mg, 50% yield. **^{1}H NMR** (300 MHz, $CDCl_3$) δ 7.93 (d, *J* = 8.2 Hz, 1H), 7.37 (dddd, *J* = 8.0, 7.0, 2.1, 0.9 Hz, 1H), 7.24 – 7.10 (m, 4H), 7.10 – 6.97 (m, 2H), 4.71 (s, 1H), 1.63 (s, 9H). **^{13}C NMR** (75 MHz, $CDCl_3$) δ 173.9 (C), 162.6 (d, *J* = 246.8 Hz, C), 149.4 (C), 140.6 (C), 132.1 (d, *J* = 3.3 Hz, C), 130.4 (d, *J* = 8.3 Hz, CH), 129.0 (CH), 127.2 (C), 125.0 (d, *J* = 24.5 Hz, CH), 116.1 (CH), 115.8 (CH), 115.3 (CH), 84.7 (C), 51.9 (CH), 28.2 (CH_3). **^{19}F NMR** (282 MHz, $CDCl_3$) δ -114.9.

General procedure 1.b[2]

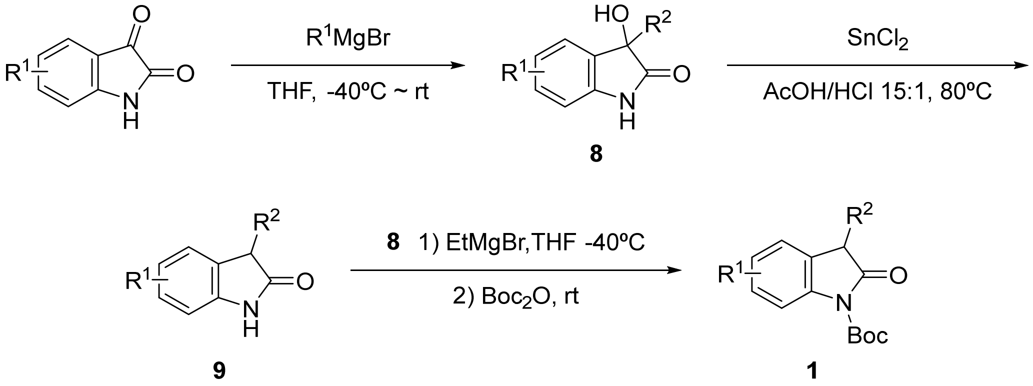

A septum-sealed flask equipped with a stirring bar was charged with the corresponding isatin (1.0 equiv.) under nitrogen atmosphere. Dry THF (15 mL) was added, and the resulting solution was cooled to -40 °C. A solution of R^1MgBr (3 M in Et_2O, 2.0 equiv.) was then added dropwise, and the reaction mixture was stirred at room temperature overnight. The reaction was cooled to 0 ºC, diluted with Et_2O (25 mL) and quenched with HCl 1 M (15 mL). The aqueous layer was extracted with Et_2O (3 x 25mL), and the combined organic phases were washed with water, brine and dried over $MgSO_4$. Solvents were removed *in vacuo* and the crude product was purified by silica gel column chromatography using DCM:EtOAc mixtures as eluent to yield the desired 3-substituted 3-hydroxyl-2-oxindole.

8 (1.0 equiv.) was suspended in AcOH/HCl (15 mL,15:1, v/v) and heated to 80 °C in an oil bath. $SnCl_2$ (2.0 equiv.) was added, and the mixture was stirred for 1.5 h. The reaction was then diluted with EtOAc (50 mL), quenched with saturated $NaHCO_3$ (50 mL) followed by Na_2CO_3(s) until neutral pH was reached, and extracted with EtOAc (3 x 50 mL). The combined organic layers

were washed with NaOH0 0.1 M (30 mL) and brine, dried over $MgSO_4$, and concentrated under reduced pressure. The crude product was purified by silica gel column chromatography using hexane:EtOAc mixtures as eluent to give the corresponding product.

9 (1.0 equiv.) was dissolved in dry THF (7.5 mL) under nitrogen atmosphere and cooled to –40 °C. EtMgBr (3 M in Et_2O, 1.2 equiv.) was added dropwise, and the mixture was stirred for 30 min. Subsequently, Boc_2O (1.5 equiv.) was added, and the reaction was allowed to stir overnight at room temperature. The mixture was diluted with EtOAc (50 mL), quenched with saturated NH_4Cl (50 mL), and extracted with EtOAc (3 x 50 mL). The combined organic layers were washed with brine, dried over $MgSO_4$, and concentrated in vacuo. The crude residue was purified by silica gel column chromatography using hexane:EtOAc mixtures as eluent to afford the desired *N*-Boc protected 3-substituted oxindole.

1-benzyl-3-hydroxy-3-phenylindolin-2-one[14]

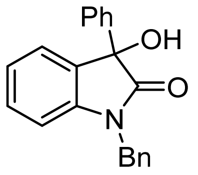

General procedure was followed using *N*-benzylisatin (2.1 mmol, 500 mg, 1 equiv.), which had previously been synthesized within the research group, and phenylmagnesium bromide (3M in Et_2O, 6.2 mmol, 2.1 mL, 2 equiv.). White solid, 400 mg, 61% yield. **^{1}H NMR** (300 MHz, $CDCl_3$) δ 7.45 – 7.39 (m, 2H), 7.38 – 7.24 (m, 9H), 7.22 (dd, *J* = 7.8, 1.3 Hz,1 H), 7.04 (td, *J* = 7.6, 0.9 Hz, 1H), 6.79 (d, *J* = 7.8 Hz, 1H), 5.05 (d, *J* = 15.7 Hz, 1H), 4.83 (d, *J* = 15.7 Hz, 1H).

1-benzyl-3-phenylindolin-2-one (1c)[4]

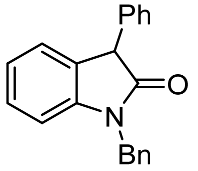

General procedure was followed using 1-benzyl-3-hydroxy-3-phenylindolin-2-one (1.3 mmol, 400 mg, 1 equiv.). Yellow solid, 288 mg, 75% yield. **^{1}H NMR** (300 MHz, $CDCl_3$) δ 7.41 – 7.28 (m, 8H), 7.27 – 7.22 (m, 3H), 7.21 – 7.16 (m, H1), 7.04 (td, *J* = 7.5, 0.9 Hz, 1H), 6.81 (d, *J* = 7.8 Hz, 1H), 5.03 (d, *J* = 15.6 Hz, 1H), 4.92 (d, *J* = 15.6 Hz, 1H), 4.73 (s, 1H). **^{13}C NMR** (75 MHz, $CDCl_3$) δ 176.2 (C), 143.7 (C), 136.9 (C), 136.0 (C), 129.1 (CH), 129.0 (C), 128.9 (CH), 128.6 (CH), 128.4 (CH), 127.8 (CH), 127.7 (CH), 127.5 (CH), 125.3 (CH), 122.9 (CH), 109.3 (CH), 52.2 (CH), 44.1 (CH_2).

5-chloro-3-hydroxy-3-phenylindolin-2-one (8e)[7]

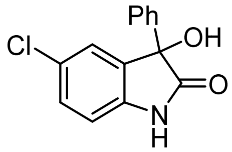

General procedure was followed using 5-chloroisatin (2.8 mmol, 500 mg, 1 equiv.) and phenylmagnesium bromide (3M in Et_2O, 6.2 mmol, 2.1 mL, 2 equiv.). Orange solid, 514 mg, 72% yield. **^{1}H NMR** (300 MHz, CD_3OD) δ 7.40 – 7.23 (m, 6H), 7.13 (d, *J* = 1.9 Hz, 1H), 6.95 (d, *J* = 8.1 Hz, 1H).

5-chloro-3-phenylindolin-2-one (9e)[10]

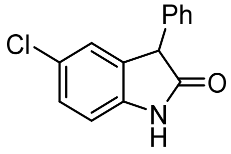

General procedure was followed using 5-chloro-3-hydroxy-3-phenylindolin-2-one (1.9 mmol, 514 mg, 1 equiv.). White solid, 340 mg, 73% yield. **^{1}H NMR** (300 MHz, $CDCl_3$) δ 8.46 (bs, 1H), 7.44 – 7.29 (m, 3H), 7.25 – 7.17 (m, 3H), 7.13 – 7.08 (m, 1H), 6.86 (d, *J* = 8.3 Hz, 1H), 4.62 (s, 1H).

te*rt*-butyl 5-chloro-2-oxo-3-phenylindoline-1-carboxylate (1e)[5]

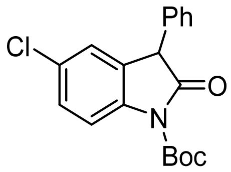

General procedure was followed using 5-chloro-3-phenylindolin-2-one (1.4 mmol, 340 mg, 1 equiv.).White solid, 332 mg, 69% yield. **^{1}H NMR** (300 MHz, $CDCl_3$) δ 7.89 (d, *J* = 8.7 Hz, 1H), 7.40 – 7.31 (m, 4H), 7.21 – 7.15 (m, 2H), 7.14 (dd, *J* = 2.3, 1.2 Hz, 1H), 4.70 (s, 1H), 1.62 (s, 9H). **^{13}C NMR** (75 MHz, $CDCl_3$) δ 173.3 (C), 149.3 (C), 139.1 (C), 135.7 (C), 130.2 (C), 129.3 (C), 129.2 (CH), 128.9 (CH), 128.7 (CH), 128.3 (CH), 125.4 (CH), 116.5 (CH), 84.9 (C), 52.6 (CH), 28.2 (CH_3).

5-bromo-3-hydroxy-3-phenylindolin-2-one (8f)[15]

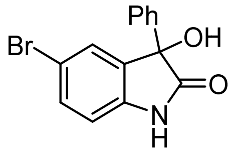

General procedure was followed using 5-bromoisatin (2.2 mmol, 500 mg, 1 equiv.) and phenylmagnesium bromide (3M in Et_2O, 6.2 mmol, 2.1 mL, 2 equiv.). Orange solid, 402 mg, 60% yield. **^{1}H NMR** (300 MHz, CD_3OD) δ 7.44 (dd, *J* = 8.3, 2.1 Hz, 1H), 7.40 – 7.28 (m, 5H), 7.26 (d, *J* = 2.0 Hz, 1H), 6.90 (d, *J* = 8.3 Hz, 1H).

5-bromo-3-phenylindolin-2-one (9f)[10]

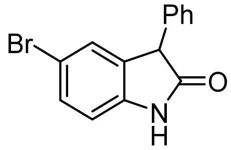

General procedure was followed using 5-bromo-3-hydroxy-3-phenylindolin-2-one (1.3 mmol, 402 mg, 1 equiv.) White solid, 350 mg, 92% yield. **^{1}H NMR** (300 MHz, $CDCl_3$) δ 9.03 (bs, 1H), 7.44 – 7.28 (m, 4H), 7.26 – 7.14 (m, 3H), 6.81 (d, *J* = 8.3 Hz, 1H), 4.63 (s, 1H).

te*rt*-butyl 5-bromo-2-oxo-3-phenylindoline-1-carboxylate (1f)[5]

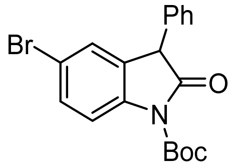

General procedure was followed using 5-bromo-3-phenylindolin-2-one (1.2 mmol, 350 mg, 1 equiv.). White solid, 244 mg, 52% yield. **^{1}H NMR** (300 MHz, $CDCl_3$) δ 7.84 (d, *J* = 8.7 Hz, 1H), 7.49 (ddd, *J* = 8.7, 2.2, 0.9 Hz, 1H), 7.41 – 7.31 (m, 3H), 7.28 (dd, *J* = 2.1, 1.2 Hz, 1H), 7.21 – 7.10 (m, 2H), 4.71 (s, 1H), 1.62 (s, 9H). **^{13}C NMR** (75 MHz, $CDCl_3$) δ 173.2 (C), 149.3 (C), 139.7 (C), 135.7 (C), 131.8 (CH), 129.7 (C), 129.2 (CH), 128.7 (CH), 128.3 (CH), 128.2 (CH), 117.7 (C), 116.9 (CH), 85.0 (C), 52.5 (CH), 28.2 (CH_3).

3-hydroxy-5-methoxy-3-phenylindolin-2-one (8g)[16]

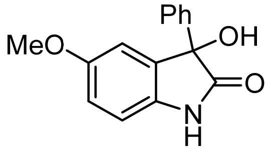

General procedure was followed using 5-methoxyisatin (2.8 mmol, 500 mg, 1 equiv.) and phenylmagnesium bromide (3M in Et_2O, 6.2 mmol, 2.1 mL, 2 equiv.). Orange solid, 480 mg, 67% yield. **^{1}H NMR** (300 MHz, CD_3OD) δ 7.55 – 7.47 (m, 2H), 7.44 – 7.31 (m, 3H), 6.99 (dd, *J* = 8.3, 0.6 Hz, 1H), 6.95 – 6.85 (m, 2H), 3.80 (s, 3H).

5-methoxy-3-phenylindolin-2-one (9g)[10]

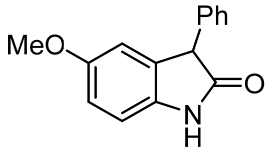

General procedure was followed using 3-hydroxy-5-methoxy-3-phenylindolin-2-one (1.9 mmol, 480 mg, 1 equiv.). Yellow solid, 240 mg, 54% yield. **^{1}H NMR** (300 MHz, $CDCl_3$) δ 8.32 (bs, 1H), 7.39 – 7.29 (m, 3H), 7.25 – 7.18 (m, 2H), 6.89 – 6.68 (m, 3H), 4.61 (s, 1H), 3.74 (s, 3H).

***tert*-butyl 5-methoxy-2-oxo-3-phenylindoline-1-carboxylate (1g)**[17]

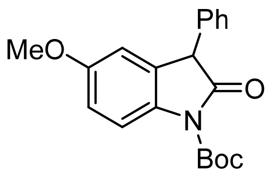

General procedure was followed using 5-methoxy-3-phenylindolin-2-one (1.0 mmol, 240 mg, 1 equiv.). Yellow solid, 238 mg, 67% yield. **^{1}H NMR** (300 MHz, $CDCl_3$) δ 7.85 (d, *J* = 8.9 Hz, 1H), 7.40 – 7.28 (m, 3H), 7.24 – 7.16 (m, 2H), 6.89 (ddd, *J* = 9.0, 2.7, 0.8 Hz, 1H), 6.72 (dd, *J* = 2.6, 1.0 Hz, 1H), 4.70 (s, 1H), 3.76 (s, 3H), 1.62 (s, 9H). **^{13}C NMR** (75 MHz, $CDCl_3$) δ 174.1 (C), 157.1 (C), 149.6 (C), 136.4 (C), 134.0 (C), 129.1 (CH), 128.8 (CH), 128.8 (C), 128.0 (CH), 116.2 (CH), 113.8 (CH), 111.2 (CH), 84.3 (C), 55.8 (CH_3), 53.0 (CH), 28.2 (CH_3).

6-chloro-3-hydroxy-3-phenylindolin-2-one (8i)[10]

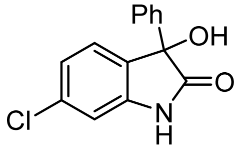

General procedure was followed using 6-chloroisatin (2.8 mmol, 500 mg, 1 equiv.) and phenylmagnesium bromide (3M in Et_2O, 6.2 mmol, 2.1 mL, 2 equiv.). Orange solid, 440 mg, 61% yield. **^{1}H NMR** (300 MHz, CD_3OD) δ 7.43 – 7.36 (m, 2H), 7.35 – 7.20 (m, 3H), 7.17 – 7.08 (m, 1H), 7.03 – 6.97 (m, 2H).

6-chloro-3-phenylindolin-2-one (9i)[5,8]

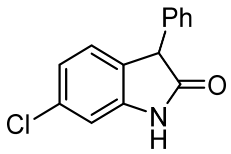

General procedure was followed using 6-chloro-3-hydroxy-3-phenylindolin-2-one (1.6 mmol, 440 mg, 1 equiv.). White solid, 330 mg, 78% yield. **^{1}H NMR** (300 MHz, $CDCl_3$) δ 8.58 (bs, 1H), 7.34 (dddt, *J* = 6.9, 5.1, 3.4, 1.7 Hz, 3H), 7.23 – 7.16 (m, 2H), 7.09 – 6.98 (m, 2H), 6.96 (d, *J* = 1.7 Hz, 1H), 4.60 (s, 1H).

tert-butyl 6-chloro-2-oxo-3-phenylindoline-1-carboxylate (1i)[5,18]

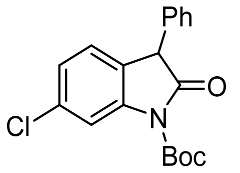

General procedure was followed using 6-chloro-3-phenylindolin-2-one (1.3 mmol, 330 mg, 1 equiv.). White solid, 352 mg, 77% yield. **^{1}H NMR** (300 MHz, $CDCl_3$) δ 8.02 (d, *J* = 1.9 Hz, 1H), 7.34 (dtd, *J* = 6.6, 5.2, 1.5 Hz, 3H), 7.17 (dt, *J* = 8.0, 2.0 Hz, 3H), 7.08 (dd, *J* = 8.0, 0.9 Hz, 1H), 4.69 (s, 1H), 1.63 (s, 9H). **^{13}C NMR** (75 MHz, $CDCl_3$) δ 173.5 (C), 149.3 (C), 141.5 (C), 135.9 (C), 134.6 (C), 129.2 (CH), 128.7 (CH), 128.2 (CH), 126.0 (CH), 125.9 (C), 124.8 (CH), 116.0 (CH), 85.1 (C), 52.3 (CH), 28.2 (CH_3).

3-hydroxy-3-(naphthalen-1-yl)indolin-2-one (8u)[20]

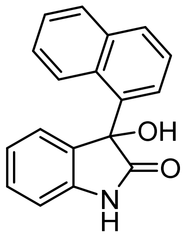

General procedure was followed using isatin (3.4 mmol, 500 mg, 1 equiv.). The required Grignard reagent was prepared *in situ* following a reported procedure.[3] Orange solid, 870 mg, 93% yield. **^{1}H NMR** (300 MHz, CD_3OD) δ 8.11 (d, *J* = 7.3 Hz, 1H), 7.89 – 7.81 (m, 2H), 7.66 (d, *J* = 7.7 Hz, 1H), 7.59 – 7.50 (m, 1H), 7.37 (ddd, *J* = 8.1, 6.9, 1.1 Hz, 1H), 7.33 – 7.18 (m, 2H), 7.07 (d, *J* = 7.8 Hz, 1H), 6.94 (dtd, *J* = 14.8, 7.4, 1.0 Hz, 2H).

3-(naphthalen-1-yl)indolin-2-one (9u)[9]

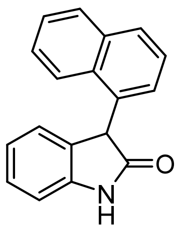

General procedure was followed using 3-hydroxy-3-(naphthalen-1-yl)indolin-2-one (3.2 mmol, 870 mg, 1 equiv.). White solid, 820 mg, 99% yield. Crude 3-hydroxy-3-(naphthalen-1-yl)indolin-2-one was used without purification.

tert-butyl 3-(naphthalen-1-yl)-2-oxoindoline-1-carboxylate (1u)[21]

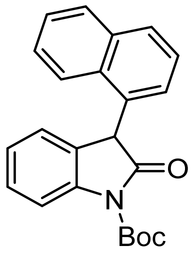

General procedure was followed using 3-(naphthalen-1-yl)indolin-2-one (3.2 mmol, 820 mg, 1 equiv.). White solid, 730 mg, 63% yield, isolated as a mixture of rotamers. Signals for the major rotamer: **^{1}H NMR** (300 MHz, $CDCl_3$) δ 8.00 (d, *J* = 8.2 Hz, 1H), 7.90 (d, *J* = 7.9 Hz, 1H), 7.83 (d, *J* = 8.3 Hz, 1H), 7.77 – 7.32 (m, 5H), 7.21 – 6.95 (m, 3H), 5.62 (bs, 1H), 1.64 (s, 9H). **^{13}C NMR** (75 MHz, $CDCl_3$) δ 173.9 (C), 149.7 (C), 134.4 (C), 129.1 (C), 129.0 (CH), 128.7 (CH), 128.3 (C), 127.0 (C), 126.9 (CH), 126.1 (CH), 125.5 (CH), 124.8 (CH), 124.1 (C), 115.3 (CH), 84.6(C), 48.2 (bs, CH), 28.2 (CH_3). Broad signals were observed for ^{1}H NMR and four ^{13}C NMR aromatic CH signals were not detected due to the presence of rotamers.

General procedure 1.c[2]

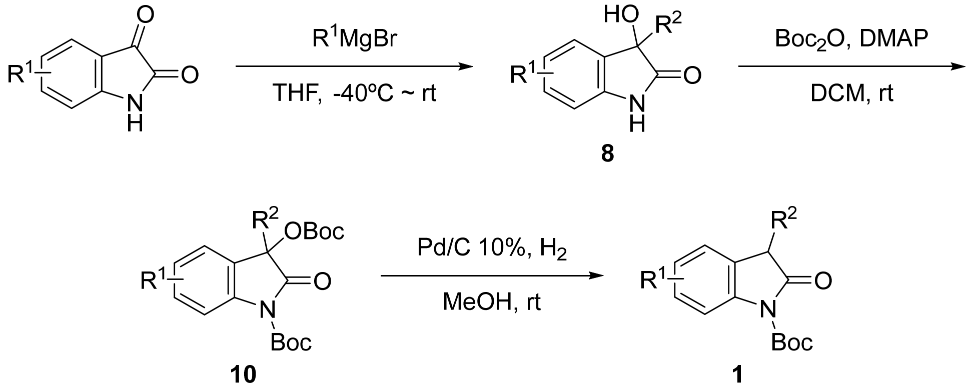

A septum-sealed flask equipped with a stirring bar was charged with the corresponding isatin (1.0 equiv.) under nitrogen atmosphere. Dry THF (15 mL) was added, and the resulting solution was cooled to -40 °C. A solution of R^1MgBr (3 M in Et_2O, 2.0 equiv.) was then added dropwise, and the reaction mixture was stirred at room temperature overnight. The reaction was cooled to 0 ºC, diluted with Et_2O (25 mL) and quenched with HCl 1 M (15 mL). The aqueous layer was extracted with Et_2O (3 x 25mL), and the combined organic phases were washed with water, brine and dried over $MgSO_4$. Solvents were removed *in vacuo* and the crude product was purified by silica gel column chromatography using DCM:EtOAc mixtures as eluent to yield the desired 3-substituted 3-hydroxyl-2-oxindole.

8 (1.0 equiv.) and DMAP (10 mol%) were dissolved in DCM (20 mL) under nitrogen atmosphere. Boc_2O (2.0 equiv.) was added at room temperature, and the mixture was stirred overnight. The reaction mixture was then diluted with EtOAc (50 mL), quenched with saturated NH_4Cl (25 mL), and extracted with EtOAc (3 x 50 mL). The combined organic layers were washed successively with water and brine, dried over $MgSO_4$, and concentrated under reduced pressure. The crude product was purified by silica gel column chromatography using hexane:EtOAc mixtures as eluent to furnish the desired compound.

10 (1.0 equiv.) was dissolved in MeOH (20 mL), and 10% Pd/C (10 mol%) was added. The suspension was stirred under hydrogen atmosphere (balloon) at room temperature overnight. After completion, the reaction mixture was filtered through a pad of Celite®, and the filtrate was concentrated *in vacuo*. The resulting residue was purified by silica gel column chromatography using hexane:EtOAc mixtures as eluent to yield the desired *N*-Boc protected 3-substituted oxindole.

3-hydroxy-4,7-dimethyl-3-phenylindolin-2-one (8k)

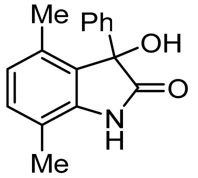

General procedure was followed using 4,7-dimethylisatin (2.9 mmol, 500 mg, 1 equiv.) and phenylmagnesium bromide (3M in Et_2O, 6.2 mmol, 2.1 mL, 2 equiv.). Orange solid, 330 mg, 46% yield. **^{1}H NMR** (300 MHz, $CDCl_3$) δ 8.25 (bs, 1H), 7.40 – 7.21 (m, 5H), 7.00 (d, *J* = 7.9 Hz, 1H), 6.74 (d, *J* = 7.9 Hz, 1H), 2.21 (s, 3H), 2.03 (s, 3H).

tert-butyl 3-((_tert_-butoxycarbonyl)oxy)-4,7-dimethyl-2-oxo-3-phenylindoline-1-carboxylate (10k)

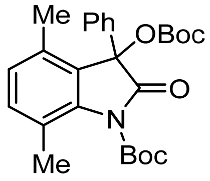

General procedure was followed using 3-hydroxy-4,7-dimethyl-3-phenylindolin-2-one (1.5 mmol, 330 mg, 1 equiv.) White solid, 571 mg, 84% yield. **^{1}H NMR** (300 MHz, $CDCl_3$) δ 7.36 – 7.21 (m, 5H), 7.16 (d, *J* = 7.9 Hz, 1H), 6.93 (d, *J* = 7.9 Hz, 1H), 2.27 (s, 3H), 2.03 (s, 3H), 1.56 (s, 9H), 1.40 (s, 9H).

4,7-dimethyl-3-phenylindolin-2-one (1k)

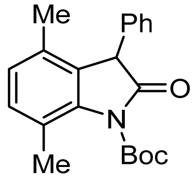

General procedure was followed using *tert*-butyl 3-((*tert*-butoxycarbonyl)oxy)-4,7-dimethyl-2-oxo-3-phenylindoline-1-carboxylate (1.3 mmol, 571 mg, 1 equiv.). White solid, 225 mg, 51% yield. **^{1}H NMR** (300 MHz, $CDCl_3$) δ 7.35 – 7.26 (m, 3H), 7.16 – 7.11 (m, 2H), 7.08 (d, *J* = 7.9 Hz, 1H), 6.88 (d, *J* = 7.9 Hz, 1H), 4.61 (s, 1H), 2.26 (s, 3H), 1.92 (s, 3H), 1.59 (s, 9H). **^{13}C NMR** (75 MHz, $CDCl_3$) δ 175.0 (C), 149.7 (C), 139.2 (C), 135.8 (C), 132.5 (C), 131.7 (CH), 129.1 (CH), 128.4 (CH), 127.9 (CH), 126.9 (C), 126.1 (CH), 120.7 (C), 84.9 (C), 52.5 (CH), 27.9 (CH_3), 19.4 (CH_3), 18.5 (CH_3). **HRMS (ESI-TOF)** calc'd for $[C_{21}H_{23}NO_3+H]^+$: 338.1751; found: 338.1758.

3-hydroxy-3-methylindolin-2-one (8w)[1]

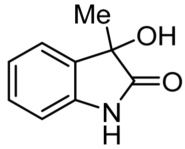

General procedure was followed using isatin (3.4 mmol, 500 mg, 1 equiv.) and methylmagnesium bromide (3M in Et_2O, 6.2 mmol, 2.1 mL, 2 equiv.). Orange solid, 450 mg, 81% yield. **^{1}H NMR** (300 MHz, CD_3OD) δ 7.34 (ddd, *J* = 7.4, 1.3, 0.6 Hz, 1H), 7.23 (td, *J* = 7.7, 1.3 Hz, 1H), 7.04 (td, *J* = 7.5, 1.0 Hz, 1H), 6.88 (dt, *J* = 7.8, 0.9 Hz, 1H), 1.50 (s, 3H).

tert-Butyl 3-((tert-butoxycarbonyl)oxy)-3-methyl-2-oxoindoline-1-carboxylate (10w)[1]

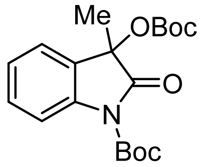

General procedure was followed using 3-hydroxy-3-methylindolin-2-one (2.8 mmol, 450 mg, 1 equiv.). Orange solid, 930 mg, 91% yield. **^{1}H NMR** (300 MHz, $CDCl_3$) δ 7.87 (dt, *J* = 8.2, 0.8 Hz, 1H), 7.44 – 7.29 (m, 2H), 7.17 (td, *J* = 7.5, 1.0 Hz, 1H), 1.65 (s, 9H), 1.64 (s, 3H), 1.34 (s, 9H).

tert-butyl 2-oxo-3-methylindoline-1-carboxylate (1w)[1]

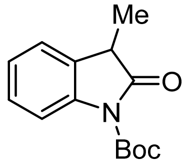

General procedure was followed using tert-Butyl 3-((tert-butoxycarbonyl)oxy)-3-methyl-2-oxoindoline-1-carboxylate (2.6 mmol, 930 mg, 1 equiv.). White solid, 465 mg, 75% yield. **^{1}H NMR** (300 MHz, $CDCl_3$) δ 7.81 (ddt, *J* = 8.2, 1.1, 0.6 Hz, 1H), 7.33 – 7.26 (m, 1H), 7.26 – 7.20 (m, 1H), 7.15 (td, *J* = 7.4, 1.1 Hz, 1H), 3.56 (q, *J* = 7.6 Hz, 1H), 1.64 (s, 9H), 1.52 (d, *J* = 7.6 Hz, 3H). **^{13}C NMR** (75 MHz, $CDCl_3$) δ 177.0 (C), 149.5 (C), 139.9 (C), 129.5 (C), 128.2 (CH), 124.5 (CH), 123.6 (CH), 115.1 (CH), 84.4 (C), 41.2 (CH), 28.2 (CH_3), 16.1 (CH_3).

General procedure 2[4,22]

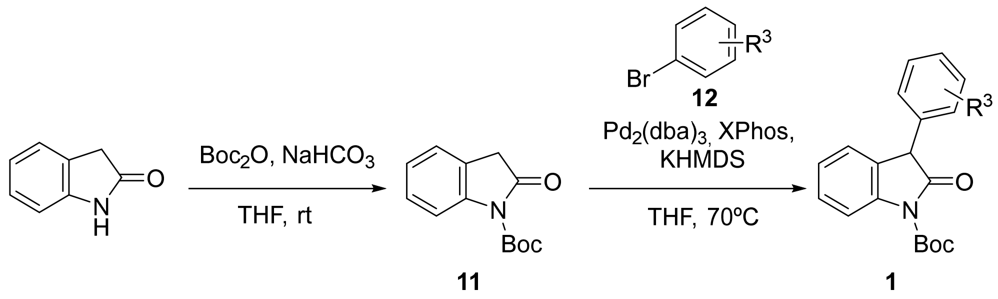

In a round bottom flask, 2-oxindole (37.5 mmol, 5.0 g, 1 equiv.) was dissolved in THF (280 mL). To this solution, Boc_2O (94 mmol, 20.5g, 2.5 equiv.) and $NaHCO_3$ (323 mmol, 28.2 g, 8.6 equiv.) were then added, and the mixture was stirred at room temperature overnight. The reaction mixture was filtered, and the solid was washed with THF. The filtrates were combined, concentrated under reduced pressure, and the crude residue was purified by silica gel column chromatography using hexane:EtOAc mixtures as eluent to furnish the corresponding *N*-Boc oxindole.

In a screw-cap tube, **11** (0.5 mmol, 117 mg, 1 equiv.), $Pd(dba)_2$ (0.01 mmol, 6 mg, 2 mol%.), and XPhos (0.14 mmol, 67 mg, 30 mol%) were introduced under nitrogen atmosphere. Anhydrous, degassed THF (0.8 mL) and aryl bromide (0.55 mmol,1.1 equiv.) were added sequentially. The mixture was heated to 70 °C in an oil bath, after which KHMDS (0.5 M in toluene, 0.55 mmol, 1.1 mL,1.1 equiv.) was added. After 30 minutes, the reaction was quenched with saturated NH_4Cl (2 mL), diluted with water (20 mL) and DCM (20 mL), and extracted with DCM (3 x 20 mL). The combined organic layers were washed with water and brine, dried over $MgSO_4$, and concentrated *in vacuo*. The crude material was purified by silica gel column chromatography usinh hexane/EtOAc mixtures as eluent to provide the desired *N*-Boc-3-aryloxindole.

Regarding the synthesis of compounds **1s**, **1t**, and **1v**, the required aryl bromides were not commercially available and therefore had to be synthetised

***tert*-butyl 2-oxoindoline-1-carboxylate (11)[4]**

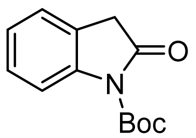

General procedure was followed using 2-oxindole (37.5 mmol, 5.0 g, 1 equiv.). White solid, 3.32 g, 38% yield. **^{1}H NMR** (300 MHz, $CDCl_3$) δ 7.86 – 7.74 (m, 1H), 7.34 – 7.23 (m, 2H), 7.14 (td, *J* = 7.5, 1.0 Hz, 1H), 3.66 (s, 1H), 1.65 (s, 4H).

***tert*-butyl 3-(3-chlorophenyl)-2-oxoindoline-1-carboxylate (1m)[17]**

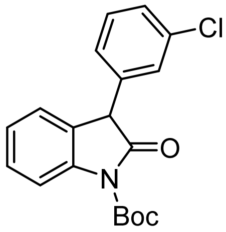

General procedure was followed using 1-bromo-3-chlorobenzene (0.55 mmol, 67µL, 1.1 equiv.). White solid, 77 mg, 45% yield. **^{1}H NMR** (300 MHz, $CDCl_3$) δ 7.94 (d, *J* = 8.2 Hz, 1H), 7.42 – 7.35 (m, 1H), 7.32 – 7.24 (m, 2H), 7.22 – 7.08 (m, 4H), 4.70 (s, 1H), 1.63 (s, 9H). **^{13}C NMR** (75 MHz, $CDCl_3$) δ 173.4 (C), 149.4 (C), 140.6 (C), 138.2 (C), 134.9 (C), 130.3 (CH), 129.1 (CH), 128.8 (CH), 128.3 (CH), 127.2 (CH), 126.7 (C), 125.2(CH), 124.9 (CH), 115.4 (CH), 84.8 (C), 52.2 (CH), 28.2 (CH_3).

tert-butyl 3-(4-cyanophenyl)-2-oxoindoline-1-carboxylate (1p)

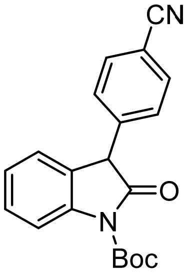

General procedure was followed using 4-bromobenzonitrile (0.55 mmol, 100 mg, 1.1 equiv.). Yellow solid, 86 mg, 50% yield. **^{1}H NMR** (300 MHz, $CDCl_3$) δ 7.95 (d, *J* = 8.3 Hz, 1H), 7.74 – 7.61 (m, 2H), 7.44 – 7.37 (m, 1H), 7.37 – 7.31 (m, 2H), 7.21 (td, *J* = 7.5, 0.9 Hz, 1H), 7.13 (d, *J* = 7.5 Hz, 1H), 4.79 (s, 1H), 1.63 (s, 9H). **^{13}C NMR** (75 MHz, $CDCl_3$) δ 173.2 (C), 149.6 (C), 141.9 (C), 141.2 (C), 133.2 (CH), 130.1 (CH), 129.9 (CH), 126.4 (C), 125.6 (CH), 125.5 (CH), 119.0 (C), 116.0 (CH), 112.5 (C), 85.5 (C), 52.9 (CH), 28.6 (CH_3). **HRMS (ESI-TOF)** calc'd for $[C_{20}H_{18}N_2O_3+H]^+$: 335.1390, found: 335.1389.

2-(4-bromophenyl)-1,3-dioxolane (12s)[23]

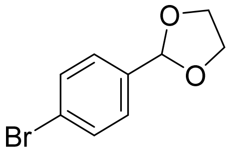

A literature procedure was carried out.[6] A mixture of ethylene glycol (58 mmol, 3.6g, 2.0 equiv.), 4-bromobenzaldehyde (29 mmol, 5.3 g, 1.0 equiv.) and *p*-toluenesulfonic acid monohydrate (0.9 mmol, 5.1 mg, 3 mol%) in dry toluene (40 mL) was refluxed in a round-bottom flask equipped with a Dean-Stark trap overnight. The reaction mixture was cooled down to room temperature, diluted with Et_2O (40 mL), quenched with saturated $NaHCO_3$ (40 mL) and extracted with Et_2O (3 x 40 mL). The combined organic layers were dried over $MgSO_4$ and solvents were removed *in vacuo*. The residue obtained was purified by silica gel column chromatography using hexane/EtOAc mixtures as eluent to afford 2-(4-bromophenyl)-1,3-dioxolane as a white solid. 3.26g, 49% yield. **^{1}H NMR** (300 MHz, $CDCl_3$) δ 7.58 – 7.47 (m, 2H), 7.42 – 7.29 (m, 2H), 5.77 (s, 1H), 4.16 – 4.07 (m, 2H), 4.07 – 3.97 (m, 2H).

tert-butyl 3-(4-(1,3-dioxolan-2-yl)phenyl)-2-oxoindoline-1-carboxylate (1s)

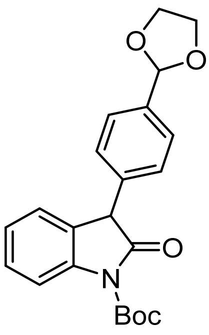

General procedure was followed using 2-(4-bromophenyl)-1,3-dioxolane (0.55 mmol, 130 mg, 1.1 equiv.). Yellow solid, 113 mg, 59% yield. **^{1}H NMR** (300 MHz, $CDCl_3$) δ 7.92 (dq, *J* = 8.1, 0.7 Hz, 1H), 7.48 – 7.42 (m, 2H), 7.36 (dddd, *J* = 8.1, 7.1, 2.0, 0.9 Hz, 1H), 7.24 – 7.08 (m, 14), 5.81 (s, 1H), 4.74 (s, 1H), 4.13 – 4.06 (m, 2H), 4.06 – 3.98 (m, 2H), 1.62 (s, 9H). **^{13}C NMR** (75 MHz, $CDCl_3$) δ 173.8 (C), 149.5 (C), 140.6 (C), 137.9 (C), 137.3 (C), 129.2 (CH), 128.8 (CH), 127.4 (C), 127.1 (CH), 125.2 (CH), 124.8 (CH), 115.3 (CH), 103.5 (CH), 84.6 (C), 65.4 (CH_2), 52.5 (CH), 28.2 (CH_3). **HRMS (ESI-TOF)** calc'd for $[C_{22}H_{23}NO_5+Na]^+$: 404.1468; found: 404.1477.

2-(4-bromophenyl)-2-(trifluoromethyl)-1,3-dioxolane (12t)[24]

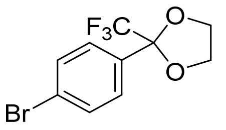

A literature procedure was carried out.[7] A solution of 1-(4-bromophenyl)-2,2,2-trifluoroethan-1-one (2.0 mmol, 500 mg, 1.0 equiv.) and 2-chloroethanol (14.8 mmol, 0.99 mL, 7.5 equiv.) in a mixture of DMF/THF (6 mL, v/v, 2:1) under argon atmosphere was cooled to -60 ºC. To this mixture, a solution of *t*-BuOK (14.8 mmol, 1.7 g, 7.5 equiv.) in DMF (3 mL) was added dropwise for 30 minutes and then stirred for 1.5 h. The reaction was quenched with saturated NH_4Cl (50 mL), rinsed with brine (30 mL) and water (30 mL), and then extracted with EtOAc (3 x 50 mL). The

combined organic layers were washed with water (70 mL) four times to remove the remaining DMF, dried over $MgSO_4$ and solvents were removed *in vacuo* to afford the 2-(4-bromophenyl)-2-(trifluoromethyl)-1,3-dioxolane as a white solid, which was used without any further purification.

tert-butyl 2-oxo-3-(4-(2-(trifluoromethyl)-1,3-dioxolan-2-yl)phenyl)indoline-1-carboxylate (1t)

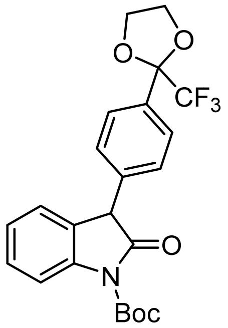

General procedure was followed using 2-(4-bromophenyl)-2-(trifluoromethyl)-1,3-dioxolane (0.55 mmol, 163 mg, 1.1 equiv.).White solid, 83 mg, 37% yield. **^{1}H NMR** (300 MHz, $CDCl_3$) δ 7.93 (d, *J* = 8.2 Hz, 1H), 7.59 (d, *J* = 8.1 Hz, 2H), 7.42 – 7.34 (m, 1H), 7.25 – 7.20 (m, 2H), 7.19 – 7.14 (m, 2H), 4.75 (s, 1H), 4.32 – 4.23 (m, 2H), 4.09 – 4.00 (m, 2H), 1.63 (s, 9H). **^{13}C NMR** (75 MHz, $CDCl_3$) δ 173.7 (C), 144.9 (C), 140.7 (C), 137.9 (C), 134.5 (C), 129.0 (CH), 128.5 (CH), 127.7 (CH), 127.1 (C), 125.3 (CH), 124.8 (CH), 115.4 (CH), 84.7 (C), 66.9 (CH_2), 52.4 (CH), 28.2 (CH_3). CF_3 and ketal quadruplet signals were not detected due to both low signal intensity and its coupling with fluorine nuclei. **^{19}F NMR** (282 MHz, $CDCl_3$) δ -82.1. **HRMS (ESI-TOF)** calc'd for $[C_{23}H_{22}F_3NO_5+Na]^+$: 472.1342; found: 472.1346.

5-bromo-1-methyl-1*H*-indole (12v)[25]

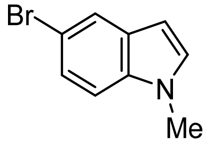

A literature procedure was carried out.[8] In a round bottom flask, 5-bromoindole (8 mmol, 1.6 g, 1.0 equiv.) was dissolved in dry THF (20 mL) under nitrogen atmosphere. To this solution, NaH (60% suspension in mineral oil, 9.6 mmol, 384 mg, 1.2 equiv.) was added at 0 ºC. The mixture was stirred for 15 minutes and then a solution of iodomethane (8 mmol, 1.14g, 1.0 equiv.) in THF (5 mL) was added at 0 ºC. The reaction mixture was stirred at room temperature overnight. The reaction was quenched with saturated NH_4Cl (10 mL), extracted three times with EtOAc (3 x 20mL) and the combined organic layers were dried over $MgSO_4$. Solvents were removed *in vacuo* and the crude product was purified by silica gel column chromatography using hexane/EtOAc mixtures as eluent to afford the desired product as a white solid. 1.55 g, 92% yield. **^{1}H NMR** (300 MHz, $CDCl_3$) δ 7.75 (dd, *J* = 1.9, 0.6 Hz, 1H), 7.30 (ddd, *J* = 8.7, 1.9, 0.4 Hz, 1H), 7.19 (dt, *J* = 8.7, 0.7 Hz, 1H), 7.05 (d, *J* = 3.1 Hz, 1H), 6.42 (dd, *J* = 3.1, 0.9 Hz, 1H), 3.78 (s, 3H).

tert-butyl 3-(1-methyl-1H-indol-5-yl)-2-oxoindoline-1-carboxylate (1v)

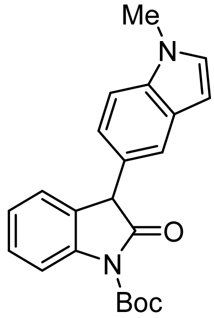

General procedure was followed using 5-bromo-1-methyl-1*H*-indole (0.55 mmol, 115 mg, 1.1 equiv.).Brown solid, 155 mg, 78 % yield. **^{1}H NMR** (500 MHz, $CDCl_3$) δ 7.94 (d, *J* = 8.2 Hz, 1H), 7.41 (s, 1H), 7.39 – 7.33 (m, 1H), 7.29 (d, *J* = 8.5 Hz, 1H), 7.23 – 7.13 (m, 2H), 7.04 (dt, *J* = 5.8, 2.0 Hz, 2H), 6.42 (d, *J* = 3.1 Hz, 1H), 4.81 (s, 1H), 3.77 (s, 3H), 1.63 (s, 9H). **^{13}C NMR** (126 MHz, $CDCl_3$) δ 174.8 (C), 149.7 (C), 140.6 (C), 136.5 (C), 129.6 (CH), 128.9 (C), 128.7 (C), 128.5 (CH), 127.4 (C), 125.3 (CH), 124.7 (CH), 122.3 (CH), 121.1 (CH), 115.1 (CH), 109.9 (CH), 101.2 (CH), 84.3 (C), 52.9 (CH), 33.0 (CH_3), 28.2 (CH_3). **HRMS (ESI-TOF)** calc'd for $[C_{22}H_{22}N_2O_3+H]^+$: 363.1703; found: 363.1704.

Synthesis of carbonyl-containing 3-aryloxindoles

Synthesis of substrate 1q

Substrate **1q** was prepared following a modified reported procedure.[26]

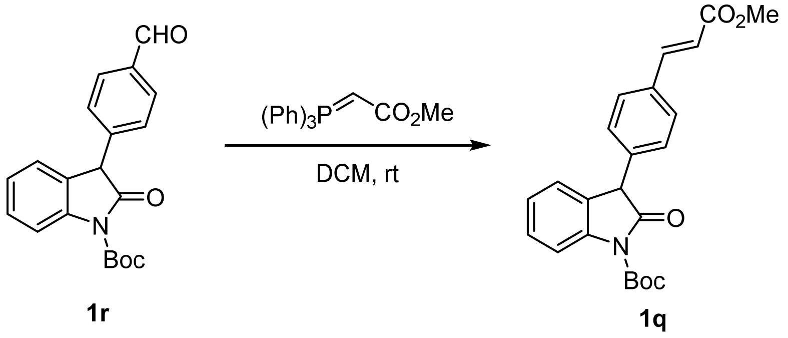

Compound **1r** (0.89 mmol, 300 mg, 1.0 equiv.) was dissolved in DCM (5 mL), and methyl 2-(triphenylphosphoranylidene)acetate (1.3 mmol, 435 mg,2.2 equiv.) was added in a single portion. The resulting mixture was stirred overnight at room temperature. The reaction was diluted with DCM (50 mL) and quenched with saturated NH_4Cl (25 mL). The aqueous phase was extracted three times with DCM (3 x 50 mL), and the combined organic layers were washed with brine, dried over $MgSO_4$, and concentrated *in vacuo*. The crude residue was purified by silica gel column chromatography using hexane/EtOAc mixtures as eluent to afford pure compound **1q**

tert-butyl (E)-3-(4-(3-methoxy-3-oxoprop-1-en-1-yl)phenyl)-2-oxoindoline-1-carboxylate (1q)

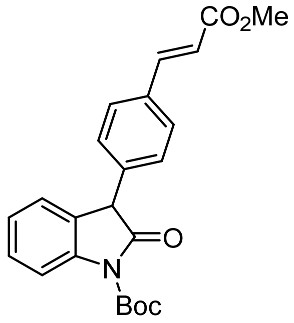

Yellow oil, 185 mg, 45% yield. **^{1}H NMR** (300 MHz, $CDCl_3$) δ 7.93 (d, *J* = 8.2 Hz, 1H), 7.67 (d, *J* = 16.1 Hz, 1H), 7.53 – 7.47 (m, 2H), 7.38 (tdd, *J* = 7.0, 2.4, 0.9 Hz, 1H), 7.25 – 7.20 (m, 2H), 7.20 – 7.12 (m, 2H), 6.42 (d, *J* = 16.0 Hz, 1H), 4.75 (s, 1H), 3.80 (s, 3H), 1.63 (s, 9H). **^{13}C NMR** (75 MHz, $CDCl_3$) δ 173.6 (C), 167.5 (C), 149.1 (C), 144.3 (CH), 140.1 (C), 138.6 (C), 134.2 (C), 129.4 (CH), 129.0 (CH), 128.7 (CH), 126.7 (C) 125.2 (CH), 124.9 (CH), 118.3 (CH), 115.4 (CH), 84.8 (C), 52.5 (CH), 51.9 (CH_3), 28.2 (CH_3). **HRMS (ESI-TOF)** calc'd for $[C_{23}H_{23}NO_5+H]^+$: 394.1649; found: 394.1654.

Synthesis of substrate 1r

Substrate **1r** was prepared following a modified reported procedure.[27]

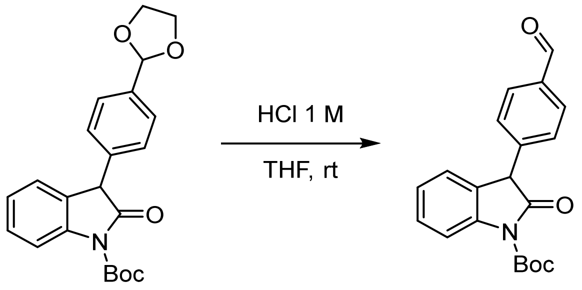

1s **1r**

A solution of compound **1s** (4.6 mmol, 1.8 g, 1.0 equiv.) in THF (70 mL) was treated with HCl 1 M (70 mL) and stirred at room temperature for 4 h. The reaction mixture was then diluted with DCM (150 mL), neutralized with saturated $NaHCO_3$ (100 mL), and extracted three times with DCM (3 x 100 mL). The combined organic layers were washed with brine, dried over $MgSO_4$, and concentrated under reduced pressure. The resulting residue was purified by silica gel column chromatography using hexane/EtOAc mixtures as eluent to afford pure compound **1r.**

***tert*-butyl 3-(4-formylphenyl)-2-oxoindoline-1-carboxylate (1r)**

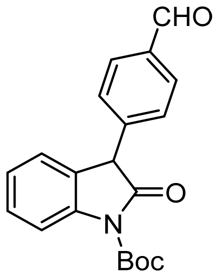

Yellow solid, 1.1 g, 70% yield. **^{1}H NMR** (300 MHz, $CDCl_3$) δ 10.01 (s, 1H), 7.95 (d, *J* = 8.1 Hz, 1H), 7.90 – 7.82 (m, 2H), 7.45 – 7.34 (m, 3H), 7.23 – 7.10 (m, 2H), 4.82 (s, 1H), 1.63 (s, 9H). **^{13}C NMR** (75 MHz, $CDCl_3$) δ 191.8, 173.1 (C), 149.3 (C), 143.0 (C), 140.7 (C), 136.1 (C), 130.4 (CH), 129.6 (CH), 129.3 (CH), 126.5 (C), 125.2 (CH), 125.0 (CH), 115.5 (CH), 84.9 (C), 52.7 (CH), 28.2 (CH_3).**HRMS (ESI-TOF)** calc'd for $[C_{20}H_{19}NO_4+H]^+$: 338.1387; found: 338.1389.

Synthesis of aliphatic 3-substituted oxindoles

Synthesis of substrates 1x-1z

Substrates **1x-z** were prepared following a modified reported procedure.[17,28,29]

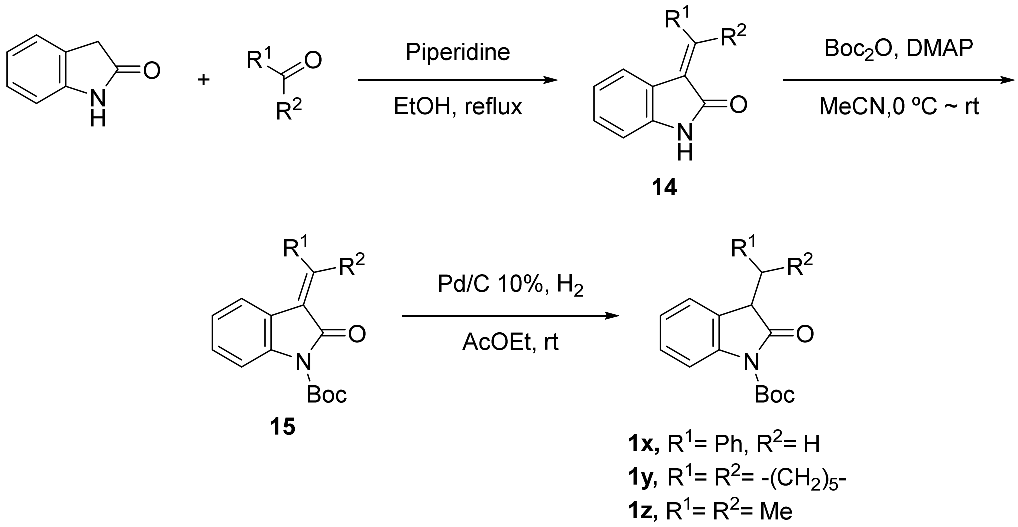

In a dry round bottom flask, 2-oxindole (1.0 equiv.) was dissolved in EtOH (0.5 M). To this solution, the corresponding carbonylic compound (1:1 ratio in volume with EtOH) and piperidine (10 equiv.) were sequentially added, and the mixture was stirred at room temperature for 3 hours. The reaction was diluted with EtOAc (30 mL) and washed with $KHSO_4$ 1 M (15 mL), water (15 mL) and brine (15 mL). The organic layer was dried over $MgSO_4$, solvents were removed *in vacuo* and the resulting crude products **14y** and **14z** were used without any further purification. Compound **14x** could be isolated after cooling the reaction mixture to 0 ºC , followed by filtration of the resulting precipitate, which was subsequently washed with cold EtOH.

14 (1.0 equiv.) was dissolved in MeCN (0.5 M) and cooled to 0 ºC. To this solution, DMAP (12 mol%) and Boc_2O (1.2 equiv.) were added and the resulting mixture was stirred at room temperature overnight. The reaction mixture was then cooled to 0ºC and quenched with saturated NH_4Cl (50 mL) and extracted three times with EtOAc (3 x 50mL. The combined organic layers were washed with HCl 0.5 M (50 mL) and saturated $NaHCO_3$ (50 mL), dried over $MgSO_4$ and concentrated under vacuum. The crude product was purified by silica gel column chromatography using hexane/DCM mixtures as eluent to afford compounds **15x-z**.

15 (1 equiv.) was dissolved in EtOAc (0.1 M) and 10% Pd/C (15 mol%) was added. The resulting suspension was stirred under hydrogen atmosphere (ballon) overnight at room temperature. The reaction mixture was filtered through Celite®, concentrated *in vacuo* and the crude product was purified by silica gel column chromatography using hexane/EtOAc mixtures as eluent to afford compounds **1x-z**.

3-benzylideneindolin-2-one (14x)[30]

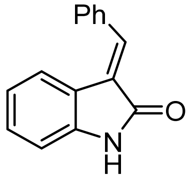

General procedure was followed using 2-oxindole (3.8 mmol, 430 mg, 1 equiv.) and benzaldehyde. Yellow solid, 240 mg, 29% yield. **^{1}H NMR** (300 MHz, $CDCl_3$) δ 8.72 (bs, 1H), 7.85 (s, 1H), 7.72 – 7.60 (m, 3H), 7.54 – 7.39 (m, 3H), 7.22 (td, *J* = 7.7, 1.0 Hz, 1H), 7.00 – 6.74 (m, 2H).

***tert*-butyl 3-benzylidene-2-oxoindoline-1-carboxylate (15x)[31]**

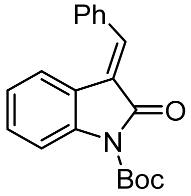

General procedure was followed using 3-benzylideneindolin-2-one (1.1 mmol, 240 mg, 1 equiv.). Yellow solid, 314 mg, 85 % yield. **^{1}H NMR** (300 MHz, $CDCl_3$) δ 7.91 (d, *J* = 8.0 Hz, 1H), 7.88 (s, 1H), 7.71 – 7.58 (m, 3H), 7.51 – 7.41 (m, 3H), 7.36 – 7.27 (m, 1H), 6.98 (td, *J* = 7.7, 1.0 Hz, 1H), 1.67 (s, 9H).

***tert*-butyl 3-benzyl-2-oxoindoline-1-carboxylate (1x)[17]**

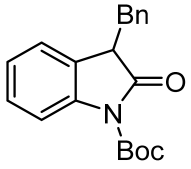

General procedure was followed using *tert*-butyl 3-benzylidene-2-oxoindoline-1-carboxylate (1.0 mmol, 314 mg, 1 equiv.). Yellow solid, 270 mg, 85% yield. **^{1}H NMR** (300 MHz, $CDCl_3$) δ 7.73 (d, *J* = 8.2 Hz, 1H), 7.30 – 7.20 (m, 4H), 7.18 – 7.12 (m, 2H), 7.00 (td, *J* = 7.5, 1.0 Hz, 1H), 6.74 (d, *J* = 7.5 Hz, 1H), 3.82 (dd, *J* = 9.1, 4.5 Hz, 1H), 3.51 (dd, *J* = 13.7, 4.5 Hz, 1H), 2.95 (dd, *J* = 13.7, 9.1 Hz, 1H), 1.63 (s, 9H). **^{13}C NMR** (75 MHz, $CDCl_3$) δ 175.6 (C), 149.3 (C), 140.1 (C), 137.5 (C), 129.6 (CH), 128.6 (CH), 128.3 (CH), 127.3 (C), 127.0 (CH), 124.5 (CH), 124.0 (CH), 115.0 (CH), 84.4 (C), 47.7 (CH), 37.8 (CH_2), 28.2 (CH_3).

***tert*-butyl 3-cyclohexylidene-2-oxoindoline-1-carboxylate (15y)[32]**

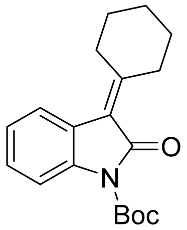

General procedure was followed using 2-oxindole (5.0 mmol, 666 mg, 1 equiv.) and cyclohexanone. Yellow oil, 760 mg, 46% yield.**^{1}H NMR** (500 MHz, $CDCl_3$) δ 7.87 (dd, *J* = 8.2, 1.1 Hz, 1H), 7.65 (d, *J* = 7.8 Hz, 1H), 7.27 – 7.22 (m, 1H), 7.11 (td, *J* = 7.6, 1.1 Hz, 1H), 3.36 – 3.30 (m, 2H), 2.92 – 2.85 (m, 2H), 1.87 – 1.79 (m, 2H), 1.81 – 1.73 (m, 2H), 1.72 – 1.67 (m, 2H), 1.65 (s, 9H).

***tert*-butyl 3-cyclohexyl-2-oxoindoline-1-carboxylate (1y)[12]**

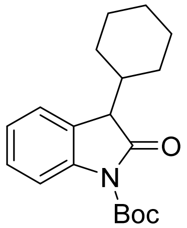

General procedure was followed using *tert*-butyl 3-cyclohexylidene-2-oxoindoline-1-carboxylate (2.4 mmol, 760 mg, 1 equiv.). Colourless oil, 580 mg, 77% yield. **^{1}H NMR** (500 MHz, $CDCl_3$) δ 7.80 (d, *J* = 8.1 Hz, 1H), 7.31 – 7.24 (m, 2H), 7.19 – 7.10 (m, 1H), 3.42 (d, *J* = 3.4 Hz, 1H), 2.21 – 2.06 (m, 1H), 1.80 – 1.67 (m, 2H), 1.67 – 1.59 (m, 11H), 1.57 – 1.50 (m, 1H), 1.37 (qd, *J* = 12.4, 3.3 Hz, 1H), 1.31 – 1.18 (m, 3H), 1.16 – 1.04 (m, 1H). **^{13}C NMR** (126 MHz, $CDCl_3$) δ 176.0 (C), 149.4 (C), 140.7 (C), 128.0 (CH), 127.2 (C), 124.3 (CH), 124.1 (CH), 114.9 (CH), 84.3 (C), 52.0 (CH), 42.1 (CH), 30.0 (CH_2), 28.8 (CH_2), 28.3 (CH_3), 26.8 (CH_2), 26.4 (CH_2), 26.1 (CH_2).

tert-butyl 2-oxo-3-(propan-2-ylidene)indoline-1-carboxylate (14z)[33]

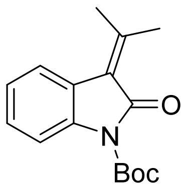

General procedure was followed using 2-oxindole (5.0 mmol, 666 mg, 1 equiv.) and acetone. Colourless oil, 940 mg, 69% yield. **^{1}H NMR** (500 MHz, $CDCl_3$) δ 7.89 (d, *J* = 8.0 Hz, 1H), 7.58 (d, *J* = 7.7 Hz, 1H), 7.31 – 7.24 (m, 1H), 7.15 (td, *J* = 7.6, 1.1 Hz, 1H), 2.61 (s, 3H), 2.41 (s, 3H), 1.66 (s, 9H).

tert-butyl 3-isopropyl-2-oxoindoline-1-carboxylate (1z)[34]

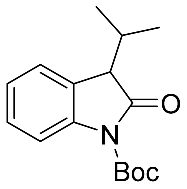

General procedure was followed using *tert*-butyl 2-oxo-3-(propan-2-ylidene)indoline-1-carboxylate (3.5 mmol, 940 mg, 1 equiv.). Colourless solid, 852 mg, 88% yield. **^{1}H NMR** (300 MHz, $CDCl_3$) δ 7.88 – 7.71 (m, 1H), 7.34 – 7.24 (m, 2H), 7.15 (dd, *J* = 7.5, 1.1 Hz, 1H), 3.46 (d, *J* = 3.4 Hz, 1H), 2.50 (heptd, *J* = 6.9, 3.6 Hz, 1H), 1.64 (s, 9H), 1.07 (d, *J* = 7.0 Hz, 3H), 0.95 (d, *J* = 6.9 Hz, 3H). **^{13}C NMR** (75 MHz, $CDCl_3$) δ 175.8 (C), 149.4 (C), 140.7 (C), 128.1 (CH), 126.9 (C), 124.2 (CH), 124.1 (CH), 114.9 (CH), 84.3 (C), 52.0 (CH), 31.9 (CH), 28.3 (CH_3), 19.5 (CH_3), 18.5 (CH_3).

General procedures and characterization for products 2a-2x

General non enantioselective procedure for the synthesis products 2a-2x

In a screw cap tube, **1a** (0.1 mmol, 1.0 equiv.) was dissolved in anhydrous THF (1 mL, 0.1 M solution) at room temperature. To this solution, triethylamine (0.1 mmol, 13.9 µL, 1.0 equiv.) and ESF (0.1 mmol, 8.3 µL, 1.0 equiv.) were added and the resulting mixture was stirred at room temperature for 4 h. Solvents were removed *in vacuo* and the crude product was purified by silica gel column chromatography to afford compound **2a**.

General procedure for the enantioselective synthesis of products 2a-2x

In a screw cap tube, **1a** (0.1 mmol, 1.0 equiv.) and $(DHQD)_2AQN$ (0.01 mmol, 8.6 mg, 0.1 equiv.) were dissolved in anhydrous THF (1 mL, 0.1 M solution). The resulting solution was cooled at -78 ºC under N_2 atmosphere, ESF (0.1 mmol, 8.3 µL, 1.0 equiv.) was added and the resulting mixture was stirred at -78 ºC for 4 h. Solvents were removed *in vacuo* and the crude product was purified by silica gel column chromatography to afford compound **2a**.

***tert*-butyl *(R)*-3-(2-(fluorosulfonyl)ethyl)-2-oxo-3-phenylindoline-1-carboxylate (2a)**

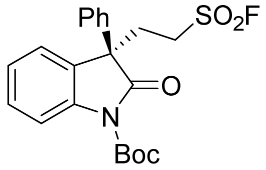

General procedure was followed using **1a** (0.1 mmol, 30.9 mg, 1.0 equiv.). The crude product was purified by silica gel chromatography (hexanes/EtOAc 9:1) affording pure **2a** as a white solid (39.8 mg, 95% yield). Compound (*R*)-**2a** was obtained with a 97% enantiomeric excess determined by HPLC using a Chiralpak IC column [n-hexane/*i*-PrOH (90:10), 1.0 mL/min]: τ_{major}= 6.8 min, τ_{minor}= 5.8 min. $[\alpha]_D^{25}$= +26.1 (c = 0.63, $CHCl_3$, 97% ee). **^{1}H NMR** (300 MHz, $CDCl_3$) δ 7.97 (dt, *J* = 8.2, 0.9 Hz, 1H), 7.43 (ddd, *J* = 8.2, 6.3, 2.7 Hz, 1H), 7.40 – 7.30 (m, 5H), 7.30 – 7.26 (m, 2H), 3.38 – 3.14 (m, 2H), 3.07 (ddd, *J* = 13.4, 12.0, 4.7 Hz, 1H), 2.71 (ddd, *J* = 13.3, 12.4, 3.7 Hz, 1H), 1.64 (s, 9H). **^{13}C NMR** (75 MHz, $CDCl_3$) δ 175.4 (C), 148.9 (C), 139.7 (C), 137.6 (C), 129.8 (CH), 129.3 (CH), 128.7 (C), 128.6 (CH), 126.8 (CH), 125.4 (CH), 124.5 (CH), 116.0 (CH), 85.4 (C), 55.1 (C), 47.00 (d, *J* = 18.1 Hz, CH_2), 31.5 (CH_2), 28.2 (CH_3). **^{19}F NMR** (282 MHz, $CDCl_3$) δ 52.8. **HRMS (ESI-TOF)** calc'd for $[C_{21}H_{22}FNO_5S+Na]^+$: 442,1095; found: 442,1086.

***(R)*-2-(1-acetyl-2-oxo-3-phenylindolin-3-yl)ethane-1-sulfonyl fluoride (2b)**

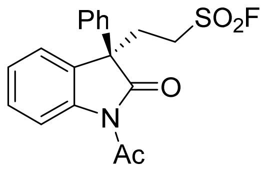

General procedure was followed using **1b** (0.1 mmol, 25.1 mg, 1.0 equiv.). The crude product was purified by silica gel chromatography (hexanes/Et_2O 9:1) affording pure **2b** as a white solid (14.7 mg, 41% yield). Compound (*R*)-**2b** was obtained with a 31% enantiomeric excess determined by HPLC using a Chiralpak IC column [n-hexane/*i*-PrOH (90:10), 1.0 mL/min]: τ_{major}= 12.0 min, τ_{minor}= 8.3 min. $[\alpha]_D^{25}$= +10.6 (c = 0.24, $CHCl_3$, 31% ee). **^{1}H NMR** (300 MHz, $CDCl_3$) δ 8.39 – 8.31 (m, 1H), 7.46 (ddd, *J* = 8.2, 7.4, 1.6 Hz, 1H), 7.42 – 7.20 (m, 7H), 3.44 – 2.96 (m, 3H), 2.76 (ddd, *J* = 13.2, 11.7, 3.7 Hz, 1H), 2.66 (s, 3H). **^{13}C NMR** (75 MHz, $CDCl_3$) δ 177.9 (C), 170.8 (C), 140.1 (C), 137.4 (C), 130.0 (CH), 129.5 (CH), 128.8 (CH), 128.8 (C), 126.7 (CH), 126.2 (CH), 124.4 (CH), 117.6 (CH), 55.3 (C), 47.0 (d, *J* = 18.4 Hz, CH_2), 31.3 (CH_2) 26.8 (CH_3). **^{19}F NMR** (282 MHz, $CDCl_3$) δ 52.9. **HRMS (ESI-TOF)** calc'd for $[C_{18}H_{16}FNO_4S+H]^+$: 362.0857; found: 362.0857.

(R)-2-(1-benzyl-2-oxo-3-phenylindolin-3-yl)ethane-1-sulfonyl fluoride (2c)

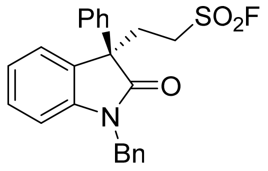

General procedure was followed using **1c** (0.1 mmol, 29.9 mg, 1.0 equiv.). The crude product was purified by silica gel chromatography (hexanes/DCM 7:3) affording pure **2c** as a yellow oil (14.3 mg, 33% yield). Compound (*R*)-**2c** was obtained with a 19% enantiomeric excess determined by HPLC using a Chiralpak IC column [n-hexane/*i*-PrOH (98:02), 1.0 mL/min]: τ_{major}= 29.3 min, τ_{minor}= 24.4 min. $[\alpha]_D^{25}$= -12.6 (c = 0.25, $CHCl_3$, 22% ee). **^{1}H NMR** (300 MHz, $CDCl_3$) δ 7.41 – 7.16 (m, 12H), 7.09 (td, *J* = 7.6, 1.1 Hz, 1H), 6.84 (dd, *J* = 8.2, 1.1 Hz, 1H), 4.98 (d, *J* = 15.5 Hz, 1H), 4.86 (d, *J* = 15.5 Hz, 1H), 3.38 – 3.12 (m, 2H), 3.01 (ddd, *J* = 13.5, 12.0, 5.0 Hz, 1H), 2.68 (ddd, *J* = 13.5, 11.8, 4.7 Hz, 1H). **^{13}C NMR** (75 MHz, $CDCl_3$) δ 177.1 (C), 142.5 (C), 137.7 (C), 135.6 (C), 130.5 (C), 129.3 (CH), 129.3 (CH), 129.1 (CH), 128.3 (CH), 128.1 (CH), 127.4 (CH), 126.7 (CH), 124.6 (CH), 123.6 (CH), 110.2 (CH), 54.6 (C), 46.8 (d, *J* = 18.0 Hz, CH_2), 44.2 (CH_2), 31.1 (CH_2). **^{19}F NMR** (282 MHz, $CDCl_3$) δ 52.6. **HRMS (ESI-TOF)** calc'd for $[C_{23}H_{20}FNO_3S+H]^+$: 410.1221; found: 410.1229.

***tert*-butyl (R)-3-(2-(fluorosulfonyl)ethyl)-5-methyl-2-oxo-3-phenylindoline-1-carboxylate (2d)**

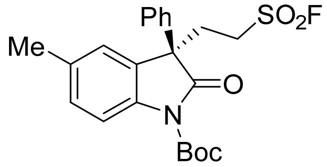

General procedure was followed using **1d** (0.1 mmol, 32.3 mg, 1.0 equiv.). The crude product was purified by silica gel chromatography (hexanes/EtOAc 9:1) affording pure **2d** as a colourless oil (41.4 mg, 96% yield). Compound (*R*)-**2d** was obtained with a 98% enantiomeric excess determined by HPLC using a Chiralpak IC column [n-hexane/*i*-PrOH (90:10), 1.0 mL/min]: τ_{major}= 7.9 min, τ_{minor}= 6.6 min. $[\alpha]_D^{25}$= +34.2 (c = 0.77, $CHCl_3$, 98% ee). **^{1}H NMR** (300 MHz, CDCl3) δ 7.83 (d, *J* = 8.4 Hz, 1H), 7.41 – 7.28 (m, 5H), 7.22 (ddd, *J* = 8.5, 1.9, 0.8 Hz, 1H), 7.06 (dd, *J* = 1.7, 1.0 Hz, 1H), 3.44 – 3.14 (m, 2H), 3.06 (ddd, *J* = 13.3, 11.8, 5.0 Hz, 1H), 2.69 (ddd, *J* = 13.4, 12.3, 4.1 Hz, 1H), 2.40 (s, 3H), 1.64 (s, 9H). **^{13}C NMR** (75 MHz, $CDCl_3$) δ 175.5 (C), 149.0 (C), 137.8 (C), 137.3 (C), 135.3 (C), 130.3 (CH), 129.3 (CH), 128.7 (C), 128.5 (CH), 126.8 (CH), 124.9 (CH), 115.7 (CH), 85.2 (C), 55.2 (C), 47.0 (d, *J* = 18.1 Hz, CH_2), 31.4 (CH_2), 28.2 (CH_3), 21.3 (CH_3). **^{19}F NMR** (282 MHz, $CDCl_3$) δ 52.8. **HRMS (ESI-TOF)** calc'd for $[C_{22}H_{24}FNO_5S+NH_4]^+$: 451.1697; found: 451.1695.

***tert*-butyl (R)-5-chloro-3-(2-(fluorosulfonyl)ethyl)-2-oxo-3-phenylindoline-1-carboxylate (2e)**

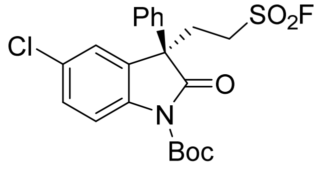

General procedure was followed using **1e** (0.1 mmol, 34.4 mg, 1.0 equiv.). The crude product was purified by silica gel chromatography (hexanes/Et_2O 9:1) affording pure **2e** as a white solid (41.7 mg, 92% yield). Compound (*R*)-**2e** was obtained with a 97% enantiomeric excess determined by HPLC using a Chiralpak IC column [n-hexane/*i*-PrOH (90:10), 1.0 mL/min]: τ_{major}= 11.3 min, τ_{minor}= 5.98 min. $[\alpha]_D^{25}$= +39.0 (c = 0.70, $CHCl_3$, 95% ee); **^{1}H NMR** (300 MHz, $CDCl_3$) δ 7.93 (d, *J* = 8.8 Hz, 1H), 7.43 – 7.33 (m, 4H), 7.32 – 7.28 (m, 2H), 7.24 (d, *J* = 2.2 Hz, 1H), 3.37 – 3.21 (m, 2H), 3.05 (ddd, *J* = 13.7, 9.7, 7.2 Hz, 1H), 2.68 (ddd, *J* = 13.7, 9.6, 7.1 Hz, 1H), 1.64 (s, 9H). **^{13}C NMR** (75 MHz, $CDCl_3$) δ 174.7 (C), 148.7 (C), 138.1 (C), 136.8 (C), 130.9 (C), 130.8 (C), 129.8 (CH), 129.6 (CH), 128.8 (CH), 126.6 (CH), 124.6 (CH), 117.2 (CH), 85.8 (C), 55.1 (C), 46.8 (d, *J* = 18.3 Hz, CH_2), 31.2 (CH_2), 28.2 (CH_3). **^{19}F NMR** (282 MHz, $CDCl_3$) δ 52.9. **HRMS (ESI-TOF)** calc'd for $[C_{21}H_{21}ClFNO_5S+H]^+$: 453.0886; found: 453.0875.

***tert*-butyl *(R)*-5-bromo-3-(2-(fluorosulfonyl)ethyl)-2-oxo-3-phenylindoline-1-carboxylate (2f)**

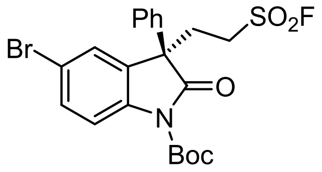

General procedure was followed using **1f** (0.1 mmol, 38.8 mg, 1.0 equiv.). The crude product was purified by silica gel chromatography (hexanes/Et_2O 9:1) affording pure **2f** as a colourless oil (44.2 mg, 89% yield). Compound (*R*)-**2f** was obtained with a 98% enantiomeric excess determined by HPLC using a Chiralpak IC column [n-hexane/*i*-PrOH (90:10), 1.0 mL/min]: τ_{major}= 13.2 min, τ_{minor}= 6.0 min. $[\alpha]_D^{25}$= +41.5 (c = 0.74, $CHCl_3$, 98% ee); **^{1}H NMR** (300 MHz, $CDCl_3$) δ 7.87 (d, *J* = 8.8 Hz, 1H), 7.55 (dd, *J* = 8.7, 2.1 Hz, 1H), 7.45 – 7.17 (m, 6H), 3.45 – 3.21 (m, 2H), 3.14 – 2.88 (m, 1H), 2.68 (ddd, *J* = 13.7, 9.5, 7.1 Hz, 1H), 1.64 (s, 9H). **^{13}C NMR** (75 MHz, $CDCl_3$) δ 174.6 (C), 148.7 (C), 138.6 (C), 136.8 (C), 132.7 (CH), 131.2 (C), 129.6 (CH), 128.8 (CH), 127.4 (CH), 126.6 (CH), 118.4 (C), 117.6 (CH), 85.8 (C), 55.1 (C), 46.8 (d, *J* = 18.3 Hz, CH_2), 31.2 (CH_2), 28.2 (CH_3). **^{19}F NMR** (282 MHz, $CDCl_3$) δ 52.9. **HRMS (ESI-TOF)** calc'd for $[C_{21}H_{21}BrFNO_5S+NH_4]^+$: 515.0648; found: 515.0648.

***tert*-butyl *(R)*-3-(2-(fluorosulfonyl)ethyl)-5-methoxy-2-oxo-3-phenylindoline-1-carboxylate (2g)**

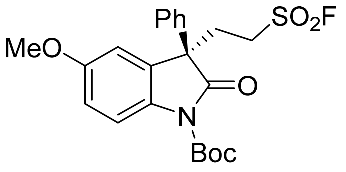

General procedure was followed using **1g** (0.1 mmol, 33.9 mg, 1.0 equiv.). The crude product was purified by silica gel chromatography (hexanes/Et_2O 8:2) affording pure **2g** as a yellow oil (45.0 mg, 99% yield). Compound (*R*)-**2g** was obtained with a 98% enantiomeric excess determined by HPLC using a Chiralpak AD-H column [n-hexane/*i*-PrOH (90:10), 1.0 mL/min]: τ_{major}= 8.2 min, τ_{minor}= 7.5 min. $[\alpha]_D^{25}$= -3.2 (c = 0.75, $CHCl_3$, 98% ee). **^{1}H NMR** (300 MHz, $CDCl_3$) δ 7.89 (d, *J* = 9.0 Hz, 1H), 7.43 – 7.25 (m, 5H), 6.94 (dd, *J* = 9.0, 2.7 Hz, 1H), 6.79 (d, *J* = 2.6 Hz, 1H), 3.82 (s, 3H), 3.48 – 3.14 (m, 2H), 3.08 (ddd, *J* = 13.4, 12.0, 4.6 Hz, 1H), 2.68 (td, *J* = 12.8, 3.6 Hz, 1H), 1.63 (s, 9H). **^{13}C NMR** (75 MHz, $CDCl_3$) δ 175.4 (C), 157.5 (C), 149.0 (C), 137.6 (C), 132.9 (C), 130.0 (C), 129.3 (CH), 128.6 (CH), 126.8 (CH), 117.0 (CH), 114.2 (CH), 110.8 (CH), 85.2 (C), 55.9 (CH_3), 55.4 (C), 47.0 (d, *J* = 18.1 Hz, CH_2), 31.3 (CH_2), 28.2 (CH_3). **^{19}F NMR** (282 MHz, $CDCl_3$) δ 52.8. **HRMS (ESI-TOF)** calc'd for $[C_{22}H_{24}FNO_6S+NH_4]^+$: 467.1647; found: 467.1641.

tert-butyl (R)-3-(2-(fluorosulfonyl)ethyl)-6-methoxy-2-oxo-3-phenylindoline-1-carboxylate (2h)

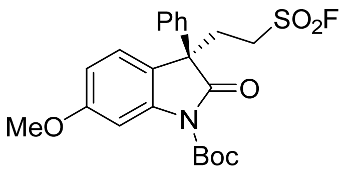

General procedure was followed using **1h** (0.1 mmol, 33.9 mg, 1.0 equiv.). The crude product was purified by silica gel chromatography (hexanes/Et_2O 9:1) affording pure **2h** as a white solid (40.7 mg, 91% yield). Compound (*R*)-**2h** was obtained with a 98% enantiomeric excess determined by HPLC using a Chiralpak IC column [n-hexane/*i*-PrOH (90:10), 1.0 mL/min]: τ_{major}= 19.4 min, τ_{minor}= 7.7 min. Colourless Oil; $[\alpha]_D^{25}$= +7.5 (c = 0.67, $CHCl_3$, 98% ee); **^{1}H NMR** (300 MHz, $CDCl_3$) δ 7.60 (d, *J* = 2.4 Hz, 1H), 7.41 – 7.29 (m, 5H), 7.16 (d, *J* = 8.4 Hz, 1H), 6.82 (dd, *J* = 8.4, 2.4 Hz, 1H), 3.87 (s, 3H), 3.37 – 3.10 (m, 2H), 3.04 (ddd, *J* = 13.4, 12.0, 4.8 Hz, 1H), 2.79 – 2.58 (m, 1H), 1.64 (s, 9H). **^{13}C NMR** (75 MHz, $CDCl_3$) δ 175.8 (C), 160.8 (C), 148.9 (C), 140.8 (C), 138.0 (C), 129.3 (CH), 128.5 (CH), 126.8 (CH), 125.2 (CH), 120.0 (C), 111.0 (CH), 102.6 (CH), 85.3 (C), 55.8 (CH_3), 54.7 (C), 47.1 (d, *J* = 18.0 Hz, CH_2), 31.7 (CH_2), 28.2 (CH_3). **^{19}F NMR** (282 MHz, $CDCl_3$) δ 52.8. **HRMS (ESI-TOF)** calc'd for $[C_{22}H_{24}FNO_6S+NH_4]^+$: 467.1647; found: 467.1645.

tert-butyl (R)-6-chloro-3-(2-(fluorosulfonyl)ethyl)-2-oxo-3-phenylindoline-1-carboxylate (2i)

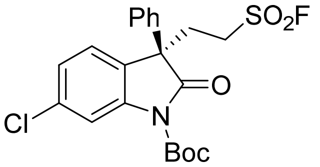

General procedure was followed using **1i** (0.1 mmol, 34.4 mg, 1.0 equiv.). The crude product was purified by silica gel chromatography (hexanes/Et_2O 9:1) affording pure **2i** as a white solid (41.0 mg, 90% yield). Compound (*R*)-**2i** was obtained with a 95% enantiomeric excess determined by HPLC using a Chiralpak IC column [n-hexane/*i*-PrOH (90:10), 1.0 mL/min]: τ_{major}= 13.60 min, τ_{minor}= 6.0 min. $[\alpha]_D^{25}$= +18.3 (c = 0.68, $CHCl_3$, 95% ee). **^{1}H NMR** (300 MHz, $CDCl_3$) δ 7.96 (d, *J* = 1.9 Hz, 1H), 7.37 – 7.15 (m, 6H), 7.11 (d, *J* = 8.1 Hz, 1H), 3.30 – 3.03 (m, 2H), 2.95 (ddd, *J* = 13.6, 11.5, 5.3 Hz, 1H), 2.59 (ddd, *J* = 13.6, 12.0, 4.4 Hz, 1H), 1.55 (s, 9H). **^{13}C NMR** (75 MHz, $CDCl_3$) δ 174.9 (C), 148.7 (C), 140.6 (C), 137.0 (C), 135.6 (C), 129.5 (CH), 128.8 (CH), 127.1 (C), 126.7 (CH), 125.5 (CH), 125.4 (CH), 116.8 (CH), 85.9 (C), 54.9 (C), 46.9 (d, *J* = 18.5 Hz, CH_2), 31.3 (CH_2), 28.1 (CH_3). **^{19}F NMR** (282 MHz, $CDCl_3$) δ 52.9. **HRMS (ESI-TOF)** calc'd for $[C_{21}H_{21}ClFNO_5S+NH_4]^+$: 471.1151; found: 471.1150.

tert-butyl (R)-7-fluoro-3-(2-(fluorosulfonyl)ethyl)-2-oxo-3-phenylindoline-1-carboxylate (2j)

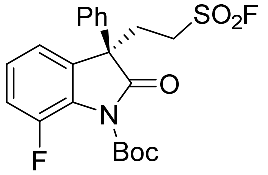

General procedure was followed using **1j** (0.1 mmol, 32.7 mg, 1.0 equiv.). The crude product was purified by silica gel chromatography (hexanes/Et_2O 9:1) affording pure **2j** as a colourless oil (34.3 mg, 79% yield). Compound (*R*)-**2j** was obtained with a 97% enantiomeric excess determined by HPLC using a Chiralpak IC column [n-hexane/*i*-PrOH (90:10), 1.5 mL/min]: τ_{major}= 9.8 min, τ_{minor}= 7.6 min. $[\alpha]_D^{25}$= +15.9 (c =0.50, $CHCl_3$, 98% ee); **^{1}H NMR** (300 MHz, $CDCl_3$) δ 7.45 – 7.29 (m, 5H), 7.29 – 7.22 (m, 1H), 7.18 (ddd, *J* = 10.8, 8.4, 1.3 Hz, 1H), 7.08 (dd, *J* = 7.3, 1.4 Hz, 1H), 3.41 – 3.16 (m, 2H), 3.06 (ddd, *J* = 13.6, 11.2, 5.6 Hz, 1H), 2.71 (ddd, *J* = 13.6, 11.7, 4.8 Hz, 1H), 1.61 (s, 9H). **^{13}C NMR** (75 MHz, $CDCl_3$) δ 174.9 (C), 148.9 (d, *J* = 252.7 Hz, C), 147.2 (C), 136.9 (C), 132.3 (d, *J* = 1.9 Hz, C), 129.5 (CH), 128.8 (CH), 126.6 (CH), 126.6 (d, *J* = 9.8 Hz, C), 126.4 (d, *J* = 7.0 Hz, CH), 120.3 (d, *J* = 3.7 Hz, CH), 118.0 (d, *J* = 20.5 Hz, CH), 86.1 (C), 55.6 (d, *J* = 1.4 Hz, C), 46.7 (d, *J* = 18.3 Hz, CH_2), 31.3 (CH_2), 27.8 (CH_3). **^{19}F NMR** (282 MHz,

$CDCl_3$) δ 52.9, -119.0. **HRMS (ESI-TOF)** calc'd for $[C_{21}H_{21}F_2NO_5S+NH_4]^+$: 455.1447; found: 455.1445.

tert-butyl _(R)_-3-(2-(fluorosulfonyl)ethyl)-4,7-dimethyl-2-oxo-3-phenylindoline-1-carboxylate (2k)

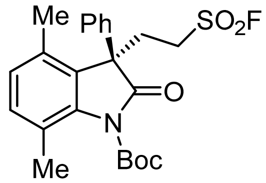

General procedure was followed using **1k** (0.1 mmol, 33.7 mg, 1.0 equiv.). The crude product was purified by silica gel chromatography (hexanes/EtOAc 95:5) affording pure **2k** as a white solid (12.0 mg, 27% yield). Compound (*R*)-**2k** was obtained with a 90% enantiomeric excess determined by HPLC using a Chiralpak IC column [n-hexane/*i*-PrOH (90:10), 1.0 mL/min]: τ_{major}= 5.1 min, τ_{minor}= 5.5 min. $[\alpha]_D^{25}$= +40.0 (c = 0.03, $CHCl_3$, 90% ee); **^{1}H NMR** (300 MHz, $CDCl_3$) δ 7.39 – 7.27 (m, 3H), 7.24 – 7.04 (m, 3H), 6.95 (d, *J* = 7.9 Hz, 1H), 3.37 – 3.09 (m, 1H), 2.97 – 2.77 (m, 2H), 2.27 (s, 3H), 2.00 (s, 3H), 1.59 (s, 9H). **^{13}C NMR** (75 MHz, $CDCl_3$) δ 176.1 (C), 149.2 (C), 138.9 (C), 137.1 (C), 132.8 (CH), 132.6 (C), 129.3 (CH), 128.4 (CH), 127.6 (CH), 127.2 (C), 126.5 (CH), 121.4 (C), 85.8 (C), 56.0 (C), 47.1 (d, *J* = 18.3 Hz, CH2), 28.2 (CH2), 27.8 (CH3), 19.3 (CH3), 18.1 (CH3). **^{19}F NMR** (282 MHz, $CDCl_3$) δ 52.8. **HRMS (ESI-TOF)** calc'd for $[C_{23}H_{26}FNO_5S+NH_4]^+$: 465.1854; found: 465.1832.

tert-butyl _(R)_-3-(2-(fluorosulfonyl)ethyl)-2-oxo-3-(o-tolyl)indoline-1-carboxylate (2l)

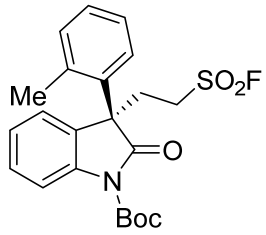

General procedure was followed using **1l** (0.1 mmol, 32.3 mg, 1.0 equiv.). The crude product was purified by silica gel chromatography (hexanes/Et_2O 9:1) affording pure **1l** as a white solid (35.5 mg, 83% yield). Compound (*R*)-**1l** was obtained with a 89% enantiomeric excess determined by HPLC using a Chiralpak IC column [n-hexane/*i*-PrOH (90:10), 1.0 mL/min]: τ_{major}= 7.3 min, τ_{minor}= 8.5 min. $[\alpha]_D^{25}$= -79.3 (c = 0.30, $CHCl_3$, 89% ee); **^{1}H NMR** (300 MHz, $CDCl_3$) δ 7.89 (d, *J* = 8.2 Hz, 1H), 7.56 – 7.47 (m, 1H), 7.36 (tdd, *J* = 7.9, 3.2, 1.5 Hz, 2H), 7.26 (dd, *J* = 14.9, 1.3 Hz, 1H), 7.20 – 7.07 (m, 2H), 6.88 (dd, *J* = 7.6, 1.4 Hz, 1H), 3.65 – 3.32 (m, 2H), 3.07 (ddd, *J* = 13.8, 12.2, 4.0 Hz, 1H), 2.54 (ddd, *J* = 13.8, 12.5, 4.5 Hz, 1H), 1.79 (s, 3H), 1.68 (s, 9H).**^{13}C NMR** (75 MHz, $CDCl_3$) δ 175.8 (C), 149.1 (C), 139.1 (C), 137.3 (C), 135.3 (C), 133.1 (CH), 130.3 (C), 129.4 (CH), 128.7 (CH), 126.8 (CH), 126.5 (CH), 125.7 (CH), 123.6 (CH), 115.3 (CH), 85.5 (C), 54.7 (C), 46.4 (d, *J* = 17.7 Hz, CH_2), 31.9 (CH_2), 28.3 (CH_3), 20.1 (CH_3). **^{19}F NMR** (282 MHz, $CDCl_3$) δ 53.0. **HRMS (ESI-TOF)** calc'd for $[C_{22}H_{24}FNO_5S+NH_4]^+$: 451.1697; found: 451.1685.

tert-butyl _(R)_-3-(3-chlorophenyl)-3-(2-(fluorosulfonyl)ethyl)-2-oxoindoline-1-carboxylate (2m)

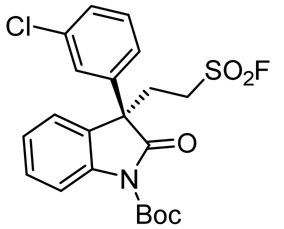

General procedure was followed using **1m** (0.1 mmol, 34.4 mg, 1.0 equiv.). The crude product was purified by silica gel chromatography (hexanes/Et_2O 9:1) affording pure **1m** as a colourless oil (45.0 mg, 99% yield). Compound (*R*)-**1m** was obtained with a 96% enantiomeric excess determined by HPLC using a Chiralpak IC column [n-hexane/*i*-PrOH (90:10), 1.0 mL/min]: τ_{major}= 7.7 min, τ_{minor}= 6.1 min. $[\alpha]_D^{25}$= +45.4 (c = 0.76, $CHCl_3$, 96% ee); **^{1}H NMR** (300 MHz, $CDCl_3$) δ 7.98 (dt, *J* = 8.2, 0.9 Hz, 1H), 7.46 (ddd, *J* = 8.2, 7.3, 1.7 Hz, 1H), 7.37 – 7.17 (m, 6H), 3.36 – 3.22 (m, 1H), 3.18 – 2.94 (m, 2H), 2.79 – 2.52 (m, 1H), 1.64 (s, 9H). **^{13}C NMR** (75 MHz, $CDCl_3$) δ 174.7 (C), 148.8 (C), 139.8 (C), 139.6 (C), 135.3 (C), 130.5 (CH), 130.1 (CH), 128.9 (CH), 127.7 (C), 127.2 (CH), 125.6 (CH), 125.1 (CH), 124.5 (CH), 116.2 (CH), 85.6 (C), 54.9 (C), 46.9 (d, *J* = 18.4 Hz, CH_2), 31.6 (CH_2), 28.2 (CH_3). **^{19}F NMR** (282 MHz, $CDCl_3$) δ 52.9. **HRMS (ESI-TOF)** calc'd for $[C_{21}H_{21}ClFNO_5S+NH_4]^+$: 471.1151; found: 471.1153.

tert-butyl _(R)_-3-(2-(fluorosulfonyl)ethyl)-3-(3-methoxyphenyl)-2-oxoindoline-1-carboxylate (2n)

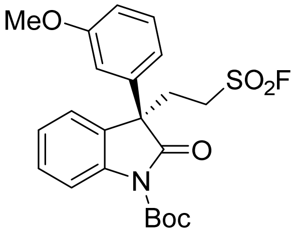

General procedure was followed using **1n** (0.1 mmol, 33.9 mg, 1.0 equiv.). The crude product was purified by silica gel chromatography (hexanes/Et_2O 9:1) affording pure **2n** as a colourless oil (38.3 mg, 85% yield). Compound (*R*)-**2n** was obtained with a 98% enantiomeric excess determined by HPLC using a Chiralpak OD-H column [n-hexane/*i*-PrOH (90:10), 1.0 mL/min]: τ_{major}= 9.1 min, τ_{minor}= 6.39 min. $[\alpha]_D^{25}$= +26.2 (c = 0.64, $CHCl_3$, 98% ee); **^{1}H NMR** (300 MHz, $CDCl_3$) δ 7.95 (dd, *J* = 8.2, 1.0 Hz, 1H), 7.50 – 7.35 (m, 1H), 7.34 – 7.22 (m, 3H), 6.96 – 6.87 (m, 1H), 6.87 – 6.81 (m, 2H), 3.77 (s, 3H), 3.41 – 3.13 (m, 2H), 3.03 (ddd, *J* = 13.5, 11.9, 5.0 Hz, 1H), 2.70 (ddd, *J* = 13.5, 12.4, 4.0 Hz, 1H), 1.64 (s, 9H).**^{13}C NMR** (75 MHz, $CDCl_3$) δ 175.2 (C), 160.2 (C), 148.9 (C),139.7 (C), 139.0 (C), 130.3 (CH), 129.8 (CH), 128.6 (C), 125.4 (CH), 124.5 (CH), 119.0 (CH), 115.9 (CH), 113.4 (CH), 113.3 (CH), 85.4 (C), 55.4 (CH_3), 55.0 (C), 47.0 (d, *J* = 18.1 Hz, CH_2), 31.4 (CH_2), 28.2 (CH_3). **^{19}F NMR** (282 MHz, $CDCl_3$) δ 52.7. **HRMS (ESI-TOF)** calc'd for $[C_{22}H_{24}FNO_6S+Na]^+$: 472.1201; found: 472.1187.

tert-butyl _(R)_-3-(4-fluorophenyl)-3-(2-(fluorosulfonyl)ethyl)-2-oxoindoline-1-carboxylate (2o)

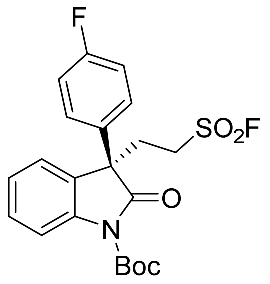

General procedure was followed using **1o** (0.1 mmol, 32.7 mg, 1.0 equiv.). The crude product was purified by silica gel chromatography (hexanes/Et_2O 9:1) affording pure **2o** as a white solid (38.7 mg, 89% yield). Compound (*R*)-**2o** was obtained with a 96% enantiomeric excess determined by HPLC using a Chiralpak IC column [n-hexane/*i*-PrOH (90:10), 1.0 mL/min]: τ_{major}= 8.0 min, τ_{minor}= 6.4 min. $[\alpha]_D^{25}$= +47.4 (c = 0.65, $CHCl_3$, 96% ee); **^{1}H NMR** (300 MHz, $CDCl_3$) δ 7.97 (dt, *J* = 8.2, 0.9 Hz, 1H), 7.45 (ddd, *J* = 8.2, 7.3, 1.7 Hz, 1H), 7.38 – 7.21 (m, 4H), 7.11 – 6.95 (m, 2H), 3.39 – 3.20 (m, 1H), 3.20 – 2.94 (m, 2H), 2.68 (td, *J* = 12.5, 2.7 Hz, 1H), 1.64 (s, 9H). **^{13}C NMR** (75 MHz, $CDCl_3$) δ 175.1 (C), 162.6 (d, *J* = 248.9 Hz, C), 148.7 (C), 139.7 (C), 133.2 (d, *J* = 3.3 Hz, C), 129.9 (CH), 128.7 (d, *J* = 8.2 Hz, CH), 128.1 (C), 125.4 (CH), 124.4 (CH), 116.3 (CH), 116.0 (d, J = 2.9 Hz, CH), 85.4 (C), 54.5 (C), 46.8 (d, *J* = 18.3 Hz, CH_2) 31.6 (CH_2), 28.1 (CH_3). **^{19}F NMR** (282 MHz, $CDCl_3$) δ 52.9, -113.7. **HRMS (ESI-TOF)** calc'd for $[C_{21}H_{21}F_2NO_5S+NH_4]^+$: 455.1447; found: 455.1439.

tert-butyl _(R)_-3-(4-cyanophenyl)-3-(2-(fluorosulfonyl)ethyl)-2-oxoindoline-1-carboxylate (2p)

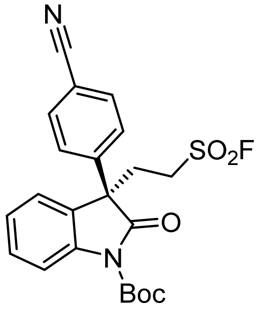

General procedure was followed using **1p** (0.1 mmol, 33.4 mg, 1.0 equiv.). The crude product was purified by silica gel chromatography (hexanes/EtOAc 9:1) affording pure **2p** as a yellow oil (44.1 mg, 99% yield). Compound (*R*)-**2p** was obtained with a 90% enantiomeric excess determined by HPLC using a Chiralpak IC column [n-hexane/*i*-PrOH (80:20), 1.0 mL/min]: τ_{major}= 28.0 min, τ_{minor}= 23.20 min. $[\alpha]_D^{25}$= +37.3 (c = 0.64, $CHCl_3$, 90% ee); **^{1}H NMR** (300 MHz, $CDCl_3$) δ 7.99 (dt, *J* = 8.3, 0.8 Hz, 1H), 7.70 – 7.59 (m, 2H), 7.54 – 7.42 (m, 3H), 7.34 (td, *J* = 7.5, 1.1 Hz, 1H), 7.28 – 7.22 (m, 1H), 3.37 – 3.18 (m, 1H), 3.16 – 2.96 (m, 2H), 2.77 – 2.63 (m, 1H), 1.63 (s, 9H). **^{13}C NMR** (75 MHz, $CDCl_3$) δ 174.3 (C), 148.6 (C), 142.7 (C), 139.9 (C), 133.0 (CH), 130.4 (CH), 127.8 (CH), 127.0 (C), 125.8 (CH), 124.5 (CH), 118.2 (C), 116.3 (CH), 112.7 (C), 85.9 (C), 55.3 (C), 46.8 (d, *J* = 18.8 Hz, CH_2), 31.5 (CH_2), 28.1 (CH_3). **^{19}F NMR** (282 MHz, $CDCl_3$) δ 53.1. **HRMS (ESI-TOF)** calc'd for $[C_{22}H_{21}FN_2O_5S+NH_4]^+$: 462.1493; found: 462.1489.

(R)-tert-butyl (R,E)-3-(2-(fluorosulfonyl)ethyl)-3-(4-(3-methoxy-3-oxoprop-1-en-1-yl)phenyl)-2-oxoindoline-1-carboxylate (2q)

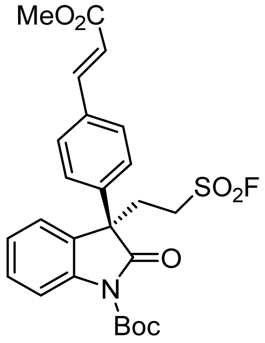

General procedure was followed using **1q** (0.1 mmol, 39.3 mg, 1.0 equiv.). The crude product was purified by silica gel chromatography (hexanes/EtOAc 7:3) affording pure **2q** as a yellow oil (32.5 mg, 65% yield). Compound (*R*)-**2q** was obtained with a 70% enantiomeric excess determined by HPLC using a Chiralpak AS-H column [n-hexane/*i*-PrOH (90:10), 1.0 mL/min]: τ_{major}= 27.9min, τ_{minor}= 15.9 min. $[\alpha]_D^{25}$= +15.1 (c = 0.40, $CHCl_3$, 77% ee); **^{1}H NMR** (300 MHz, $CDCl_3$) δ 7.97 (d, *J* = 8.2 Hz, 1H), 7.64 (d, *J* = 16.0 Hz, 1H), 7.54 – 7.41 (m, 3H), 7.39 – 7.32 (m, 2H), 7.34 – 7.21 (m, 2H), 6.42 (d, *J* = 16.0 Hz, 1H), 3.80 (s, 3H), 3.38 – 2.99 (m, 3H), 2.70 (td, *J* = 12.6, 2.8 Hz, 1H), 1.63 (s, 9H). **^{13}C NMR** (75 MHz, $CDCl_3$) δ 174.9 (C), 167.3 (C), 148.8 (C), 143.6 (CH), 139.8 (C), 139.5 (C), 134.7 (C), 130.0 (CH), 128.8 (CH), 128.1 (C), 127.4 (CH), 125.5 (CH) , 124.5 (CH), 119.1 (CH), 116.1 (CH), 85.6 (C), 55.1 (C), 51.9 (CH_3), 46.9 (d, *J* = 18.2 Hz, CH_2), 31.4 (CH_2), 28.2 (CH_3). **^{19}F NMR** (282 MHz, $CDCl_3$) δ 52.9. **HRMS (ESI-TOF)** calc'd for $[C_{25}H_{26}FNO_7S+NH_4]^+$: 521.1752; found: 521.1745.

***tert*-butyl *(R)*-3-(2-(fluorosulfonyl)ethyl)-3-(4-formylphenyl)-2-oxoindoline-1-carboxylate (2r)**

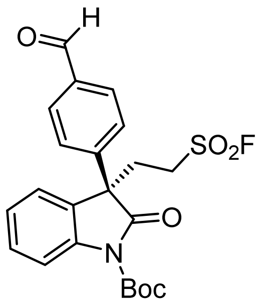

General procedure was followed using **1r** (0.1 mmol, 33.7 mg, 1.0 equiv.). The crude product was purified by silica gel chromatography (DCM/EtOAc 99:1) affording pure **2r** as a yellow oil (35.9 mg, 80% yield). Compound (*R*)-**2r** was obtained with a 72% enantiomeric excess determined by HPLC using a Lux i-Amylose-1column [n-hexane/*i*-PrOH (95:5), 1.0 mL/min]: τ_{major}= 15.9 min, τ_{minor}= 17.8 min. $[\alpha]_D^{25}$= +5.3 (c = 0.60, $CHCl_3$, 73% ee); **^{1}H NMR** (300 MHz, $CDCl_3$) δ 10.01 (s, 1H), 8.02 – 7.96 (m, 1H), 7.93 – 7.82 (m, 2H), 7.56 – 7.43 (m, 3H), 7.33 (td, *J* = 7.5, 1.1 Hz, 1H), 7.29 – 7.23 (m, 1H), 3.38 – 3.03 (m, 3H), 2.83 – 2.66 (m, 1H), 1.64 (s, 9H). **^{13}C NMR** (75 MHz, $CDCl_3$) δ 191.5 (C), 174.6 (C), 148.7 (C), 144.0 (C), 139.8 (C), 136.2 (C), 130.4 (CH), 130.2 (CH), 127.7 (CH), 127.7 (C), 125.7 (CH), 124.5 (C), 116.2 (CH), 85.7(C), 55.4 (C), 46.8 (d, *J* = 18.5 Hz, CH_2), 31.4 (CH_2), 28.1 (CH_3).**^{19}F NMR** (282 MHz, $CDCl_3$) δ 53.0. **HRMS (ESI-TOF)** calc'd for $[C_{22}H_{22}FNO_6S+NH_4]^+$: 465.1490; found: 465.1483.

***tert*-butyl (*R)*-3-(4-(1,3-dioxolan-2-yl)phenyl)-3-(2-(fluorosulfonyl)ethyl)-2-oxoindoline-1-carboxylate (2s)**

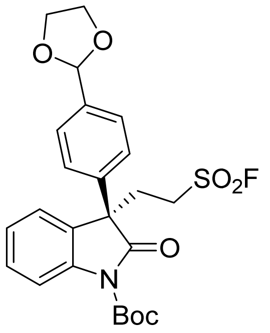

General procedure was followed using **1s** (0.1 mmol, 38.1 mg, 1.0 equiv.). The crude product was purified by silica gel chromatography (hexanes/Et_2O 7:3) affording pure **2s** as a yellow oil (36.5 mg, 74% yield). Compound (*R*)-**2s** was obtained with a 97% enantiomeric excess determined by HPLC using a Chiralpak IC column [n-hexane/*i*-PrOH (90:10), 1.0 mL/min]: τ_{major}= 20.3 min, τ_{minor}= 17.3 min. $[\alpha]_D^{25}$= +19.4 (c = 0.67, $CHCl_3$, 97% ee); **^{1}H NMR** (300 MHz, $CDCl_3$) δ 7.96 (dt, *J* = 8.2, 0.8 Hz, 1H), 7.50 – 7.39 (m, 3H), 7.37 – 7.30 (m, 2H), 7.29 – 7.20 (m, 2H), 5.79 (s, 1H), 4.09 (ddd, *J* = 7.1, 5.9, 3.7 Hz, 2H), 4.05 – 3.93 (m, 2H), 3.46 – 3.12 (m, 2H), 3.06 (ddd, *J* = 13.4, 11.9, 4.8 Hz, 1H), 2.80 – 2.62 (m, 1H), 1.63 (s, 9H). **^{13}C NMR** (75 MHz, $CDCl_3$) δ 175.1 (C), 148.8 (C), 139.6 (C), 138.4 (C), 138.3 (C), 129.7 (CH), 128.4 (C), 127.3 (CH), 126.8 (CH), 125.3 (CH), 124.4 (CH), 115.9 (CH), 103.0 (CH), 85.3 (C), 65.4 (CH_2), 54.9 (C), 46.8 (d, *J* = 18.3 Hz, CH_2), 31.2 (CH_2), 28.1 (CH_3). **^{19}F NMR** (282 MHz, $CDCl_3$) δ 52.8. **HRMS (ESI-TOF)** calc'd for $[C_{24}H_{26}FNO_7S+NH_4]^+$: 509.1752; found: 509.1746.

***tert*-butyl (*R)*-3-(2-(fluorosulfonyl)ethyl)-2-oxo-3-(4-(2-(trifluoromethyl)-1,3-dioxolan-2-yl) phenyl)indoline-1-carboxylate (2t)**

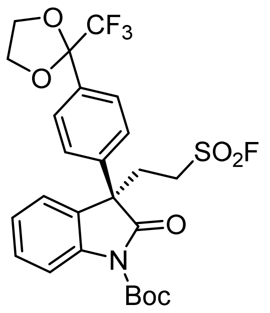

General procedure was followed using **1t** (0.1 mmol, 44.9 mg, 1.0 equiv.). The crude product was purified by silica gel chromatography (hexanes/EtOAc 9:1) affording pure **2t** as a white solid (49.1 mg, 88% yield). Compound (*R*)-**2t** was obtained with a 98% enantiomeric excess determined by HPLC using a Chiralpak IC column [n-hexane/*i*-PrOH (90:10), 1.0 mL/min]: τ_{major}= 8.4 min, τ_{minor}= 6.5 min. $[\alpha]_D^{25}$= +26.4 (c = 0.82, $CHCl_3$, 98% ee); **^{1}H NMR** (300 MHz, $CDCl_3$) δ 7.95 – 7.80 (m, 1H), 7.53 (d, *J* = 8.4 Hz, 2H), 7.37 (ddd, *J* = 8.2, 6.8, 2.1 Hz, 1H), 7.32 – 7.25 (m, 2H), 7.24 – 7.14 (m, 2H), 4.25 – 4.10 (m, 2H), 4.03 – 3.91 (m, 2H), 3.31 – 2.91 (m, 3H), 2.63 (td, *J* = 12.6, 3.0 Hz, 1H), 1.56 (s, 9H). **^{13}C NMR** (75 MHz, $CDCl_3$) δ 175.1 (C), 148.8 (C), 139.8 (C), 139.1 (C), 135.2 (C), 129.9 (CH), 128.2 (C), 127.9 (CH) 126.6 (CH), 125.5 (CH), 124.6 (CH), 122.8 (q, *J* = 288.5 Hz, CF_3). 116.1 (CH), 104.5 (q, *J* = 32.3 Hz, C), 85.5 (C), 67.0 (CH_2), 55.0 (C), 46.9 (d, *J* = 18.2 Hz, CH_2), 31.5 (CH_2), 28.2 (CH_3). **^{19}F NMR** (282 MHz, $CDCl_3$) δ 52.8, -82.1. **HRMS (ESI-TOF)** calc'd for $[C_{25}H_{25}F_4NO_7S+NH_4]^+$: 577.1626; found: 577.1636.

tert-butyl (R)-3-(2-(fluorosulfonyl)ethyl)-3-(naphthalen-1-yl)indoline-1-carboxylate (2u)

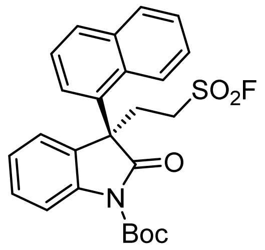

General procedure was followed using **1u** (0.1 mmol, 35.9 mg, 1.0 equiv.). The crude product was purified by silica gel chromatography (hexanes/EtOAc 9:1) affording pure **2u** as a colourless oil (46.1 mg, 89% yield, 7:1 diastereomeric ratio determined by ^{1}H NMR). Compound (*R*)-**2u** was obtained with a 90% enantiomeric excess for the major diastereomer and 79% for the minor diastereomer, both determined by HPLC using a Chiralpak AD column [n-hexane/*i*-PrOH (90:10), 1.0 mL/min]. $[\alpha]_D^{25}$= -61.4 (c = 0.77, $CHCl_3$, 7:1 dr, 90/79% ee). **HRMS (ESI-TOF)** calc'd for $[C_{25}H_{24}FNO_5S+NH_4]^+$: 487.1697; found: 487.1697.

Major diastereomer: τ_{major}= 8.9 min, τ_{minor}= 5.9 min. **^{1}H NMR** (300 MHz, $CDCl_3$) δ 8.05 (d, *J* = 8.2 Hz, 1H), 7.95 – 7.77 (m, 2H), 7.76 – 7.66 (m, 1H), 7.58 (t, *J* = 7.8 Hz, 1H), 7.48 – 7.33 (m, 2H), 7.36 – 7.20 (m, 1H), 7.16 – 7.01 (m, 2H), 6.88 (dd, *J* = 7.6, 1.4 Hz, 1H), 3.73 – 3.56 (m, 1H), 3.54 – 3.32 (m, 1H), 3.29 – 3.11 (m, 1H), 2.89 – 2.60 (m, 1H), 1.70 (s, 9H). **^{13}C NMR** (75 MHz, $CDCl_3$) δ 175.9 (C), 149.2 (C), 138.5 (C), 135.0 (C), 132.5 (C), 131.7 (C), 131.0 (C), 130.3 (CH), 129.7 (CH), 129.6 (CH), 127.0 (CH), 126.0 (CH), 125.8 (CH), 125.8 (CH), 125.1 (CH), 123.5 (CH), 123.2 (CH), 115.9 (CH), 85.6 (C), 55.0 (C), 46.5 (d, *J* = 17.9 Hz, CH_2), 32.5 (CH_2), 28.2 (CH_3). **^{19}F NMR** (282 MHz, $CDCl_3$) δ 53.2.

Minor diastereomer: τ_{major}= 6.9 min, τ_{minor}= 5.2 min **^{1}H NMR** (300 MHz, $CDCl_3$) δ 8.13 (d, *J* = 1.9 Hz, 1H), 7.95 – 7.80 (m, 2H), 7.72 (td, *J* = 7.5, 1.2 Hz, 1H), 7.58 (t, *J* = 7.8 Hz, 1H), 7.48 – 7.33 (m, 2H), 7.36 – 7.20 (m, 1H), 7.16 – 7.01 (m, 2H), 6.79 (d, *J* = 8.1 Hz, 1H), 3.73 – 3.56 (m, 1H), 3.54 – 3.32 (m, 1H), 3.29 – 3.11 (m, 1H), 2.89 – 2.60 (m, 1H), 1.70 (s, 9H). **^{13}C NMR** (75 MHz, $CDCl_3$) δ 175.4 (C), 149.0 (C), 138.5 (C), 135.3 (C), 131.8 (C), 130.9 (C), 130.5 (CH), 130.1 (CH), 129.8 (CH), 127.2 (CH), 126.1 (CH), 125.8 (CH), 124.4 (CH), 122.9 (CH), 116.7 (CH), 86.2 (C), 54.7 (C), 46.3 (d, *J* = 17.7 Hz, CH_2), 32.3 (CH_2), 28.2 (CH_3). 2 **^{19}F NMR** (282 MHz, $CDCl_3$) δ 53.3. Two ^{13}C NMR signals were not detected due to low intensity of the minor diastereomer signals and overlapping with major diastereomer signals.

tert-butyl (R)-3-(2-(fluorosulfonyl)ethyl)-3-(1-methyl-1H-indol-5-yl)indoline-1-carboxylate (2v)

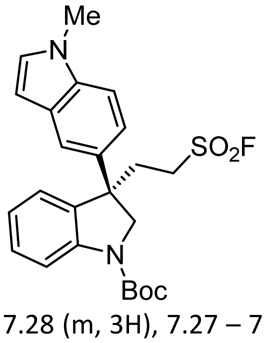

General procedure was followed using **1v** (0.1 mmol, 36.2 mg, 1.0 equiv.). The crude product was purified by silica gel chromatography (hexanes/EtOAc 8:2) affording pure **2v** as a brown oil (42.1 mg, 92% yield). Compound (*R*)-**2v** was obtained with a 99% enantiomeric excess determined by HPLC using a Chiralpak IC column [n-hexane/*i*-PrOH (90:10), 1.0 mL/min]: τ_{major}= 14.8 min, τ_{minor}= 12.2 min. $[\alpha]_D^{25}$= +11.5 (c = 0.70, $CHCl_3$, 99% ee); **^{1}H NMR** (300 MHz, $CDCl_3$) δ 8.01 – 7.94 (m, 1H), 7.49 (dd, *J* = 2.0, 0.7 Hz, 1H), 7.43 (ddd, *J* = 8.2, 6.7, 2.3 Hz, 1H), 7.36 – 7.28 (m, 3H), 7.27 – 7.22 (m, 1H), 7.06 (d, *J* = 3.1 Hz, 1H), 6.44 (dd, *J* = 3.1, 0.8 Hz, 1H), 3.77 (s, 3H), 3.44 – 3.22 (m, 2H), 3.13 (ddd, *J* = 13.5, 11.5, 5.3 Hz, 1H), 2.78 (ddd, *J* = 13.4, 12.0, 4.3 Hz, 1H), 1.64 (s, 9H). **^{13}C NMR** (75 MHz, $CDCl_3$) δ 176.0 (C), 149.1 (C), 139.6 (C), 136.3 (C), 130.0 (CH), 129.6 (C), 129.3 (CH), 128.6 (C), 128.2 (C), 125.1 (CH), 124.5 (CH), 120.0 (CH), 119.3 (CH), 115.8 (CH), 110.1 (CH), 101.4 (CH), 85.0 (C), 55.0 (C), 47.1 (d, *J* = 17.8 Hz CH_2), 32.9 (CH_3), 31.6 (CH_2),

28.1 (CH_3). **^{19}F NMR** (282 MHz, $CDCl_3$) δ 52.7. **HRMS (ESI-TOF)** calc'd for $[C_{24}H_{25}FN_2O_5S+NH_4]^+$: 490.1806; found: 490.1795.

tert-butyl _(R)_-3-(2-(fluorosulfonyl)ethyl)-3-methyl-2-oxoindoline-1-carboxylate (2w)

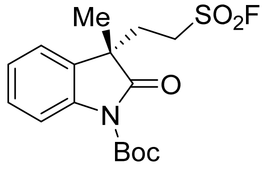

General procedure was followed using **1w** (0.1 mmol, 24.7 mg, 1.0 equiv.). The crude product was purified by silica gel chromatography (hexanes/EtOAc 9:1) affording pure **2w** as a colourless oil (31.3 mg, 88% yield). Compound (*R*)-**2w** was obtained with a 53% enantiomeric excess determined by HPLC using a Chiralpak AD-H column [n-hexane/*i*-PrOH (98:2), 1.0 mL/min]: τ_{major}= 7.7 min, τ_{minor}= 6.9 min. $[\alpha]_D^{25}$= +11.9 (c = 0.52, $CHCl_3$, 56% ee). **^{1}H NMR** (300 MHz, $CDCl_3$) δ 7.92 – 7.83 (m, 1H), 7.36 (ddd, *J* = 8.1, 6.1, 2.9 Hz, 1H), 7.23 (td, *J* = 4.3, 2.5 Hz, 2H), 3.34 – 3.02 (m, 2H), 2.54 (ddd, *J* = 13.6, 12.4, 4.9 Hz, 1H), 2.30 (td, *J* = 13.3, 4.0 Hz, 1H), 1.66 (s, 9H), 1.49 (s, 3H). **^{13}C NMR** (75 MHz, $CDCl_3$) δ 177.3 (C), 149.0 (C), 139.0 (C), 130.4 (C), 129.3 (CH), 125.4 (CH), 122.5 (CH), 115.7 (CH), 85.2 (C), 47.1 (C), 46.7 (d, *J* = 18.2 Hz, CH_2), 31.8 (CH_2), 28.2 (CH_3), 24.5 (CH_3).**^{19}F NMR** (282 MHz, $CDCl_3$) δ 52.5. **HRMS (ESI-TOF)** calc'd for $[C_{16}H_{20}FNO_5S+H]^+$: 358.1119; found: 358.1117.

tert-butyl (_S_)-3-benzyl-3-(2-(fluorosulfonyl)ethyl)-2-oxoindoline-1-carboxylate (2x)

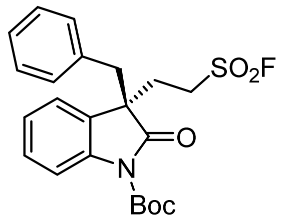

General procedure was followed using **1x** (0.1 mmol, 32.3 mg, 1.0 equiv.). The crude product was purified by silica gel chromatography (hexanes/Et_2O 9:1) affording pure **2x** as a yellow solid (30.6 mg, 71% yield). Compound (*R*)-**2x** was obtained with a 30% enantiomeric excess determined by HPLC using a Chiralpak IC column [n-hexane/*i*-PrOH (90:10), 1.0 mL/min]: τ_{major}= 8.2 min, τ_{minor}= 7.2 min. $[\alpha]_D^{25}$= -3.1 (c = 0.51, $CHCl_3$, 31% ee). **^{1}H NMR** (300 MHz, $CDCl_3$) δ 7.67 – 7.56 (m, 1H), 7.37 – 6.94 (m, 6H), 6.78 (dt, *J* = 6.7, 1.6 Hz, 2H), 3.32 – 3.16 (m, 2H), 3.09 – 2.90 (m, 2H), 2.76 (td, *J* = 13.0, 4.6 Hz, 1H), 2.47 (td, *J* = 13.1, 3.5 Hz, 1H), 1.57 (s, 9H). **^{13}C NMR** (75 MHz, $CDCl_3$) δ 176.3 (C), 148.3 (C), 139.8 (C), 133.6 (C), 129.8 (CH), 129.4 (CH), 127.9 (CH), 127.3 (CH), 127.1(C), 124.8 (CH), 123.1 (CH), 115.3 (CH), 84.7 (C), 53.3 (C), 46.7 (d, *J* = 18.3 Hz, CH_2), 45.4 (CH_2), 29.9 (CH_3), 28.0 (CH_3). **^{19}F NMR** (282 MHz, $CDCl_3$) δ 52.6. **HRMS (ESI-TOF)** calc'd for $[C_{22}H_{24}FNO_5S+NH_4]^+$: 451.1697; found: 451.1690.

Unsuccessful Substrates

We have also studied secondary 3-alkylsubstituted oxindoles, in particular cyclohexyl and isopropyl-substituted derivatives **1y** and **1z**, respectively. These substrates showed to be less reactive and, after 72 h at 4 ºC, delivered the desired product in just 16% and 6% yield, respectively (determined by ^{1}H NMR using 1,3,5-trimethoxybenzene as internal standard). The enantioselectivity for compound **2y** was modest (23%ee), while for compound **2z** measurement was not possible. Moreover, we also engaged styryl sulfonyl fluoride, but no product formation was observed after 72 hours at room temperature. Finally, double addition product **17** was observed when *N*-Boc oxindole **11** was used as the nucleophile.

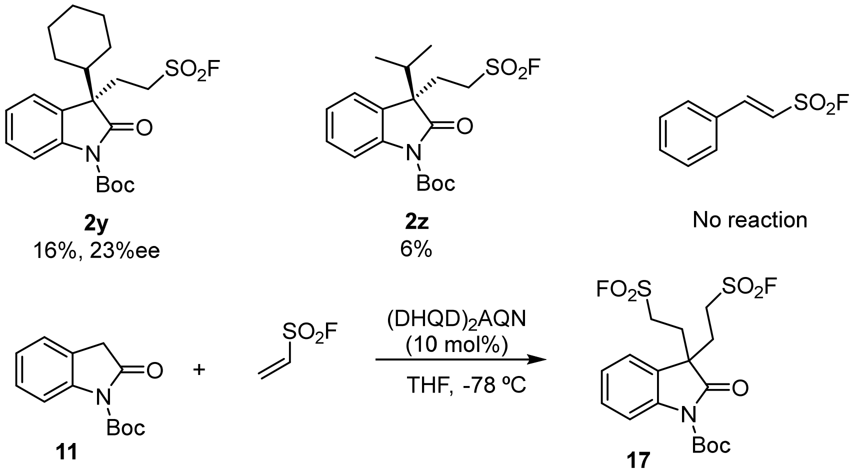

***tert*-butyl (*S*)-3-(cyclohexylmethyl)-3-(2-(fluorosulfonyl)ethyl)-2-oxoindoline-1-carboxylate (2y)**

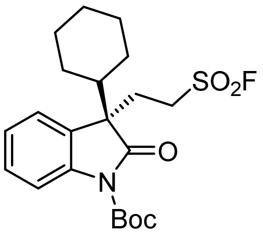

General procedure was followed using **1y** (0.1 mmol, 31.5 mg, 1.0 equiv.). Compound (*S*)-**2y** was obtained in 16% yield, and with a 23% enantiomeric excess determined by HPLC using a Chiralpak IC column [n-hexane/*i*-PrOH (90:10), 1.0 mL/min]: τ_{major}= 6.9 min, τ_{minor}= 5.6 min. **^{1}H NMR** (500 MHz, $CDCl_3$) δ 7.85 (d, *J* = 8.2 Hz, 1H), 7.36 (ddd, *J* = 8.4, 6.4, 2.6 Hz, 1H), 7.25 – 7.18 (m, 2H), 3.25 – 3.09 (m, 1H), 2.97 (dddd, *J* = 14.3, 12.8, 5.8, 3.5 Hz, 1H), 2.58 (td, *J* = 13.1, 4.6 Hz, 1H), 2.38 (td, *J* = 13.2, 3.6 Hz, 1H), 1.86 (tt, *J* = 11.8, 3.1 Hz, 1H), 1.81 – 1.72 (m, 2H), 1.65 (s, 9H), 1.64 – 1.60 (m, 2H), 1.56 – 1.52 (m, 1H), 1.27 – 1.14 (m, 3H), 1.08 – 0.95 (m, 1H), 0.79 (qd, *J* = 12.6, 3.5 Hz, 1H). **^{13}C NMR** (126 MHz, $CDCl_3$) δ 177.4 (C), 148.8 (C), 140.1 (C), 129.2 (CH), 127.9 (C),125.0 (CH), 123.5 (CH), 115.3 (CH), 85.2 (C), 55.0 (C), 46.8 (d, *J* = 17.9 Hz, CH_2), 46.1 (CH), 28.5 (CH_2), 28.2 (CH_3), 27.4 (CH_2), 26.8 (CH_2), 26.5 (CH_2), 26.1 (CH_2), 26.0 (CH_2). **^{19}F NMR** (471 MHz, $CDCl_3$) δ 52.9. **HRMS (ESI-TOF)** calc'd for $[C_{21}H_{28}FNO_5S+NH_4]^+$: 443.2010; found: 443.2001.

***tert*-butyl (*S*)-3-(2-(fluorosulfonyl)ethyl)-3-isobutyl-2-oxoindoline-1-carboxylate (2z)**

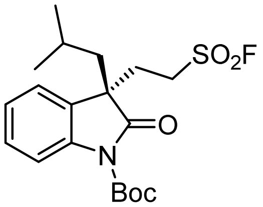

General procedure was followed using **1z** (0.1 mmol, 27.5 mg, 1.0 equiv.). Compound (*S*)-**2z** was obtained in 6% yield, determined by ^{1}H NMR using 1,3,5-trimethoxybenzene as internal standard. Enantiomeric excess was not determined. **^{1}H NMR** (500 MHz, $CDCl_3$) δ 7.87 (d, *J* = 8.3 Hz, 1H), 7.42 – 7.33 (m, 1H), 7.26 – 7.18 (m, 2H), 3.18 (dddd, *J* = 14.2,

12.9, 4.6, 3.2 Hz, 1H), 2.96 (dddd, *J* = 14.3, 12.7, 5.9, 3.6 Hz, 1H), 2.59 (td, *J* = 13.1, 4.6 Hz, 1H), 2.38 (td, *J* = 13.2, 3.6 Hz, 1H), 2.20 (hept, *J* = 6.8 Hz, 1H), 1.65 (s, 9H), 1.02 (d, *J* = 6.9 Hz, 3H), 0.78 (d, *J* = 6.7 Hz, 3H). **^{13}C NMR** (126 MHz, $CDCl_3$) δ 177.1 (C), 148.8 (C), 140.2 (C), 129.3 (CH), 127.3 (C), 125.0 (CH), 123.5 (CH), 115.4 (CH), 85.2 (C), 54.8 (C), 46.9 (d, *J* = 17.9 Hz, CH_2), 36.3 (CH), 28.9 (CH_2), 28.2 (CH_3), 17.3 (CH_3), 17.0 (CH_3). **^{19}F NMR** (471 MHz, $CDCl_3$) δ 52.9. **HRMS (ESI-TOF)** calc'd for $[C_{18}H_{24}FNO_5S+NH_4]^+$:403.1697; found:403.1693.

tert-butyl 3,3-bis(2-(fluorosulfonyl)ethyl)-2-oxoindoline-1-carboxylate (17)

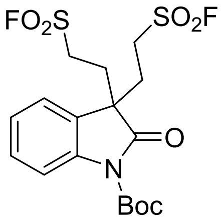

General procedure was followed using **11** (0.1 mmol, 23.3 mg, 1.0 equiv.). The crude product was purified by silica gel chromatography (hexanes/EtOAc 9:1) affording pure **17** as a white solid (18.7 mg, 41% yield). **^{1}H NMR** (300 MHz, $CDCl_3$) δ 7.92 (dt, *J* = 8.3, 0.9 Hz, 1H), 7.46 (ddd, *J* = 8.2, 7.5, 1.5 Hz, 1H), 7.33 (td, *J* = 7.5, 1.1 Hz, 1H), 7.26 (ddd, *J* = 7.5, 1.6, 0.6 Hz, 1H), 3.21 (dddd, *J* = 14.3, 12.8, 4.8, 3.5 Hz, 2H), 3.00 (dddd, *J* = 14.3, 12.4, 5.5, 3.9 Hz, 2H), 2.61 (td, *J* = 13.0, 12.4, 4.8 Hz, 2H), 2.38 (td, *J* = 13.1, 3.8 Hz, 2H), 1.67 (s, 9H). **^{13}C NMR** (75 MHz, $CDCl_3$) δ 174.9 (C), 148.3 (C), 139.7 (C), 130.8 (CH), 126.3 (CH), 125.8 (C), 122.7 (CH), 116.3 (CH), 86.3 (C), 49.9 (C), 46.1 (d, *J* = 19.0 Hz, CH_2), 31.3 (CH_2), 28.2 (CH_3). **^{19}F NMR** (282 MHz, $CDCl_3$) δ 53.1. **HRMS (ESI-TOF)** calc'd for $[C_{17}H_{21}F_2NO_7S_2+NH_4]^+$:471.1066; found:471.1061.

1 mmol-scale synthesis and transformations of compounds 2

1 mmol-scale synthesis of compound 2a

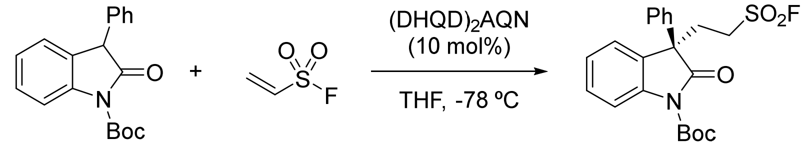

1a 1.0 mmol, 310 mg **ESF** 1.0 mmol, 83 μL ***(R)*-2a** 423 mg (99% yield), 98% ee

In a round bottom flask, **1a** (1.0 mmol, 310 mg, 1.0 equiv.) and $(DHQD)_2AQN$ (0.1 mmol, 86 mg, 0.1 equiv.) were dissolved in anhydrous THF (10 mL, 0.1 M solution). The resulting solution was cooled at -78 ºC under N_2 atmosphere, ESF (1.0 mmol, 83 µL, 1.0 equiv.) was added and the resulting mixture was stirred at -78 ºC for 4 h. Solvents were removed *in vacuo* and the crude product was purified by silica gel column chromatography (hexanes/EtOAc 9:1) to afford compound **2a** (423 mg, 99% yield) and 98% enantiomeric excess.

Sulfonamide synthesis *via* SuFEx click reactions

Compound **3** was obtained following a reported procedure.[35]

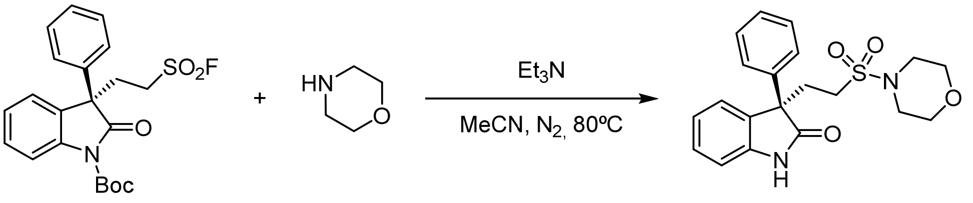

2a **3**

In a screw cap tube, **2a** (0.1 mmol, 41.9 mg, 1.0 equiv.) and morpholine (0.2 mmol, 17.3 µL, 2.0 equiv.) were dissolved in 1 mL of anhydrous MeCN under N_2 atmosphere at room temperature. To this solution Et_3N was added (0.2 mmol, 27.9 µL, 0.2 equiv.) and the resulting mixture was stirred at 80ºC overnight in an oil bath to achieve full conversion. The reaction mixture was concentrated *in vacuo* and the resulting residue was purified by silica gel column chromatography.

***(R)-tert*-butyl 3-(2-(morpholinosulfonyl)ethyl)-2-oxo-3-phenylindoline-1-carboxylate (3)**

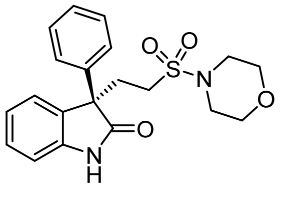

Procedure was followed using **2a** (0.1 mmol, 41.9 mg, 1.0 equiv.). The crude product was purified by silica gel chromatography (DCM/EtOAc 6:4) affording pure **3** as a white solid (35.8 mg, 93% yield). Compound (*R*)-**3** was obtained with a 98% enantiomeric excess determined by HPLC using a Chiralpak AD-H column [n-hexane/*i*-PrOH (90:10), 1.0 mL/min]: τ_{major}= 65.3 min, τ_{minor}= 61.2 min. $[\alpha]_D^{25}$= +34.5 (c = 0.60, $CHCl_3$, 98% ee); **1H NMR** (300 MHz, CDCl3) δ 8.85 (s, 1H), 7.43 – 7.27 (m, 6H), 7.21 (dd, *J* = 7.5, 1.3 Hz, 1H), 7.11 (td, *J* = 7.6, 1.1 Hz, 1H), 6.98 (d, *J* = 7.6 Hz, 1H), 3.78 – 3.64 (m, 4H), 3.27 – 3.14 (m, 4H), 2.99 – 2.71 (m, 3H), 2.70 – 2.54 (m, 1H).**^{13}C NMR** (75 MHz, $CDCl_3$) δ 179.9 (C), 140.6 (C), 138.3 (C), 131.5 (C), 129.1 (CH), 129.1 (CH), 128.1 (CH), 126.8 (CH), 124.9 (CH), 123.4 (CH), 110.8

(CH), 66.6 (CH_2), 45.9 (CH_2), 45.1 (CH_2), 30.7 (CH_2). **HRMS (ESI-TOF)** calc'd for $[C_{20}H_{22}N_2O_4S+H]^+$: 387.1373; found: 387.1377.

Sulfonate arylesters synthesis *via* SuFEx click reactions

Compounds **4** and **5** were obtained following a reported procedure.[35]

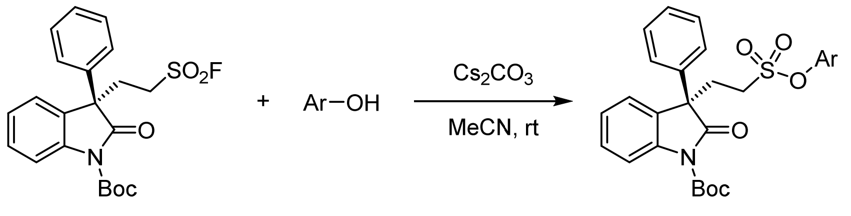

In a screw cap tube, **2a** (0.1 mmol, 41.9 mg, 1.0 equiv.), the corresponding phenol (0.1 mmol,1.0 equiv.) and Cs_2CO_3 (0.1 mmol, 32.6 mg, 1.0 equiv.) were dissolved in 1 mL of anhydrous MeCN at room temperature. The resulting mixture was stirred at rt overnight to achieve full conversion. The reaction mixture was concentrated *in vacuo* and the resulting residue was purified by silica gel column chromatography.

(R)-tert-butyl 3-(2-((benzo[*d*][1,3]dioxol-5-yloxy)sulfonyl)ethyl)-2-oxo-3-phenylindoline-1-carboxylate (4)

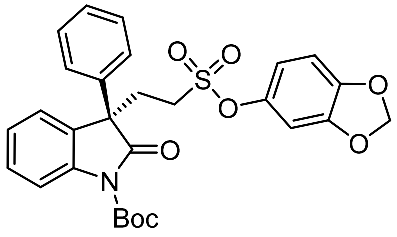

General procedure was followed using sesamol (0.1 mmol, 13.8 mg, 1.0 equiv.). The crude product was purified by silica gel chromatography (hexanes/EtOAc 8:2) affording pure **4** as a colourless oil (45.6 mg, 85% yield). Compound (*R*)-**4** was obtained with a 98% enantiomeric excess determined by HPLC using a Chiralpak IC column [n-hexane/*i*-PrOH (80:20), 1.0 mL/min]: τ_{major}= 34.8 min, τ_{minor}= 18.11 min. $[\boldsymbol{\alpha}]_D^{25}$= +33.0 (c = 0.76, $CHCl_3$, 98% ee); **^{1}H NMR** (300 MHz, $CDCl_3$) δ 7.87 (dt, *J* = 8.3, 0.9 Hz, 1H), 7.38 – 7.30 (m, 1H), 7.29 – 7.24 (m, 4H), 7.24 – 7.16 (m, 3H), 6.70 – 6.63 (m, 2H), 6.61 – 6.53 (m, 1H), 5.91 (s, 2H), 3.17 – 2.97 (m, 2H), 2.96 – 2.82 (m, 1H), 2.78 – 2.62 (m, 1H), 1.55 (s, 9H). **^{13}C NMR** (75 MHz, $CDCl_3$) δ 175.6 (C), 149.0 (C), 148.4 (C), 146.8 (C), 143.0 (C), 139.8 (C), 138.0 (C), 129.6 (CH), 129.2 (CH), 129.0 (C), 128.4 (CH), 126.9 (CH), 125.2 (CH), 124.7 (CH), 115.8 (CH), 115.0 (CH), 108.2 (CH), 104.4 (CH), 102.2 (CH_2), 85.2 (C), 55.3 (C), 46.2 (CH_2), 31.9 (CH_2), 28.2 (CH_3). **HRMS (ESI-TOF)** calc'd for $[C_{28}H_{27}NO_8S+NH_4]^+$: 555.1796; found: 555.1798.

tert-butyl (_R_)-3-(2-((((8_R_,9_S_,13_S_,14_S_)-13-methyl-17-oxo-7,8,9,11,12,13,14,15,16,17-decahydro -6H-cyclopenta[_a_]phenanthren-3-yl)oxy)sulfonyl)ethyl)-2-oxo-3-phenylindoline-1-carboxylate (5)

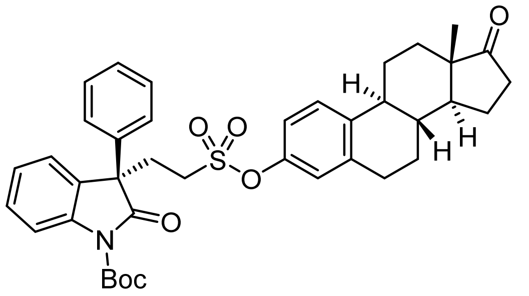

General procedure was followed using estrone (0.1 mmol, 27.0 mg, 1.0 equiv.). The crude product was purified by silica gel chromatography (DCM/Et_2O 95:5) affording pure **5** as a colourless oil (64.2 mg, 96% yield). Compound **5** was obtained with a >20:1 diastereomeric ratio, determined by ^1H NMR. $[\boldsymbol{\alpha}]_{\boldsymbol{D}}^{\boldsymbol{25}}$ = +78.9 (c = 1.07, $CHCl_3$, >20:1 dr). **^{1}H NMR** (500 MHz, $CDCl_3$) δ 7.94 (d, *J* = 8.2 Hz, 1H), 7.41 (dq, *J* = 8.2, 4.1 Hz, 1H), 7.35 (d, *J* = 4.3 Hz, 4H), 7.33 – 7.22 (m, 4H), 7.02 – 6.93 (m, 2H), 3.22 – 3.09 (m, 2H), 3.02 – 2.87 (m, 3H), 2.80 (td, *J* = 13.8, 13.2, 3.6 Hz, 1H), 2.57 – 2.47 (m, 1H), 2.39 (dq, *J* = 12.2, 3.4, 2.9 Hz, 1H), 2.27 (td, *J* = 10.8, 4.4 Hz, 1H), 2.15 (dt, *J* = 18.9, 8.9 Hz, 1H), 2.09 – 1.93 (m, 3H), 1.63 (s, 9H), 1.59 – 1.39 (m, 6H), 0.91 (s, 3H). **^{13}C NMR** (75 MHz, $CDCl_3$) δ 220.7 (C), 175.7 (C), 149.0 (C), 147.0 (C), 139.8 (C), 139.2 (C), 139.0 (C), 138.1 (C), 129.5 (CH), 129.2 (CH), 129.0 (C), 128.4 (CH), 127.0 (CH), 126.9 (CH), 125.2 (CH), 124.7 (CH), 122.1 (CH), 119.3 (CH), 115.8 (CH), 85.2 (C), 55.4 (C), 50.5 (CH), 48.0 (C), 46.3 (CH_2), 44.2 (CH), 38.0 (CH), 36.0 (CH_2), 31.9 (CH_2), 31.6 (CH_2), 29.4 (CH_2), 28.2 (CH_3), 26.3 (CH_2), 25.8 (CH_2), 21.7 (CH_2), 13.9 (CH_3). **HRMS (ESI-TOF)** calc'd for $[C_{39}H_{43}NO_7S+NH_4]^+$: 687.3099; found: 687.3095.

O-sulfonation of carbohydrates *via* SuFEx click reaction

Compound **6** was obtained following a reported procedure.[35,36]

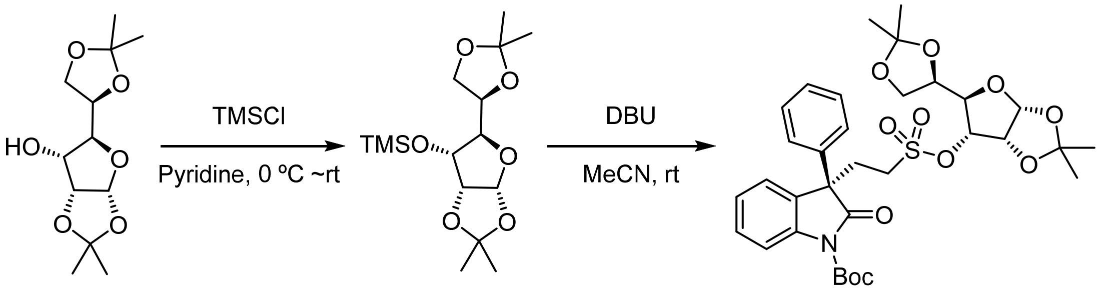

16 **6**

In a flask, 1,2:5,6-di-*O*-isopropylidene-α-D-allofuranose (1.0 mmol, 260 mg, 1.0 equiv.) was dissolved in pyridine (10 mL, 0.1 M solution) under N_2 atmosphere. To this solution TMSCl (1.2 mmol, 130 mg, 1.2 equiv.) was added at 0 ºC and the resulting mixture was stirred overnight at rt. The solution was diluted with EtOAc, washed three times with water and the organic layer was dried over $MgSO_4$. Solvents were removed *in vacuo* and the crude product was purified by silica gel column chromatography to afford the desired product.

In a screw cap tube, **2a** (0.1 mmol, 41.9 mg, 1.0 equiv.) and the TMS-protected 1,2:5,6-di-*O*-isopropylidene-α-D-allofuranose (0.11 mmol, 36.6 mg, 1.1 equiv.) were dissolved in 1 mL of dry MeCN under N_2 atmosphere. To this solution, DBU (0.02 mmol, 3.0 µL, 0.2 equiv.) was added and the mixture was stirred at rt overnight to achieve full conversion. Solvents were removed *in vacuo* and the resulting crude product was purified by silica gel column chromatography to afford the desired product.

(((3a*R*,5*R*,6*R*,6a*R*)-5-((*R*)-2,2-dimethyl-1,3-dioxolan-4-yl)-2,2-dimethyltetrahydrofuro[2,3-*d*][1,3]dioxol-6-yl)oxy)trimethylsilane (16)

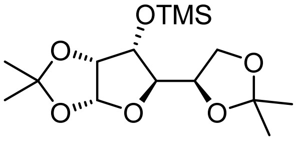

White solid, 55 mg, 17% yield. ^{1}**H NMR** (300 MHz, $CDCl_3$) δ 5.75 (d, *J* = 3.7 Hz, 1H), 4.49 – 4.42 (m, 1H), 4.36 (td, *J* = 7.1, 2.9 Hz, 1H), 4.11 – 4.03 (m, 2H), 4.00 (s, 1H), 3.98 (d, *J* = 1.0 Hz, 1H), 1.57 (s, 3H), 1.45 (s, 3H), 1.37 (s, 3H), 1.34 (s, 3H), 0.19 (s, 9H).

***tert-butyl* (*R*)-3-(2-((((3a*R*,5*R*,6*R*,6a*R*)-5-((*R*)-2,2-dimethyl-1,3-dioxolan-4-yl)-2,2-dimethyl tetrahydrofuro[2,3-*d*][1,3]dioxol-6-yl)oxy)sulfonyl)ethyl)-2-oxo-3-phenylindoline-1 carboxylate (6)**

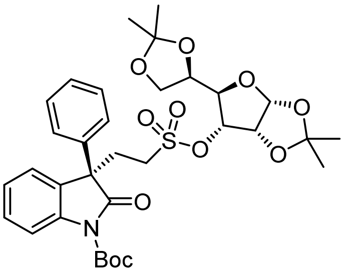

Procedure was followed using **2a** (0.1 mmol, 41.9 mg, 1.0 equiv.). The crude product was purified by silica gel chromatography (hexanes/EtOAc 7:3) affording pure **6** as a white solid (50.0 mg, 76% yield). Compound **6** was obtained with a >20:1 diastereomeric ratio, determined by ^{1}H NMR. $[\alpha]_D^{25}$= +39.5 (c = 0.83, $CHCl_3$, >20:1 dr); ^{1}**H NMR** (300 MHz, $CDCl_3$) δ 7.93 (dt, *J* = 8.1, 0.9 Hz, 1H), 7.39 (ddd, *J* = 8.2, 5.5, 3.5 Hz, 1H), 7.37 – 7.28 (m, 4H), 7.30 – 7.22 (m, 3H), 5.82 – 5.75 (m, 1H), 4.80 – 4.69 (m, 2H), 4.33 – 4.21 (m, 1H), 4.15 – 4.07 (m, 1H), 4.03 (dd, *J* = 8.7, 6.8 Hz, 1H), 3.87 (dd, *J* = 8.7, 6.0 Hz, 1H), 3.24 – 2.99 (m, 3H), 2.73 (td, *J* = 9.9, 2.5 Hz, 1H), 1.63 (s, 9H), 1.53 (s, 3H), 1.35 (s, 6H), 1.28 (s, 3H). ^{13}**C NMR** (75 MHz, $CDCl_3$) δ 175.6 (C), 149.0 (C), 139.7 (C), 138.2 (C), 129.5 (C), 129.4 (CH), 129.1 (CH), 128.3 (CH), 126.9 (CH), 125.1 (CH), 124.6 (CH), 115.7 (CH), 113.8 (C), 110.3 (C), 104.0 (CH), 85.1 (C), 78.0 (CH), 76.7 (CH), 74.8 (CH), 65.6 (CH_2), 55.3 (C), 47.7 (CH_2), 31.4 (CH_2), 28.2 (CH_3), 26.8 (CH_3), 26.8 (CH_3), 26.2 (CH_3), 25.0 (CH_3). **HRMS (ESI-TOF)** calc'd for $[C_{33}H_{41}NO_{11}S+NH_4]^+$: 677.2739; found: 677.2751.

Suzuki coupling with compound 2f

Compound **7** was obtained following a reported procedure.[37]

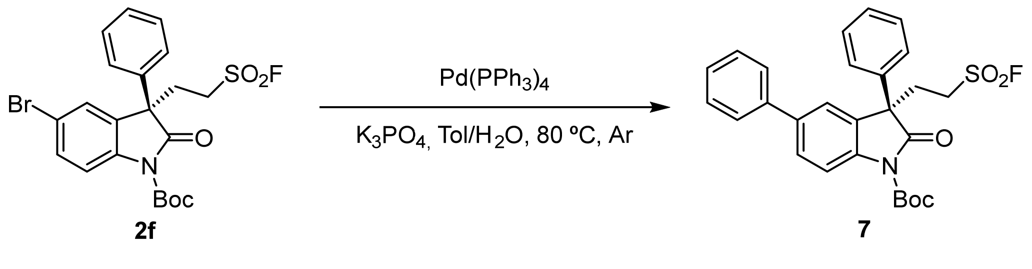

In a screw cap tube, **2f** (0.075 mmol, 37.6 mg, 1.0 equiv.) was dissolved in 1 mL of a degasified mixture of Tol/H2O (10:3 v/v) under Ar atmosphere at rt. To this solution, phenylboronic acid (0.11 mmol, 13.8 mg, 1.5 equiv.), $Pd(Ph_3)_4$ (0.004 mmol, 4.4 mg, 0.05 equiv.) and K_3PO_4 (0.19 mmol, 40.0 mg, 2.5 equiv.) were added. The resulting mixture was stirred at 80 ºC in an oil bath for 4 hours. The crude reaction mixture was diluted with toluene, washed three times with water, brine and the organic layer was dried over $MgSO_4$. Solvents were removed *in vacuo* and the resulting residue was purified by silica gel column chromatography.

tert-butyl (R)-3-(2-(fluorosulfonyl)ethyl)-2-oxo-3,5-diphenylindoline-1-carboxylate (7)

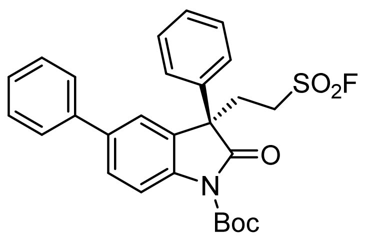

Procedure was followed using **2f** (0.075 mmol, 37.6 mg, 1.0 equiv.). The crude product was purified by silica gel chromatography (hexanes/EtOAc 95:5) affording pure **7** as a white solid (24.2 mg, 49% yield). Compound (*R*)-**7** was obtained with a 95% enantiomeric excess determined by HPLC using a Chiralpak IC column [n-hexane/*i*-PrOH (90:10), 1.0 mL/min]: τ_{major}= 8.2 min, τ_{minor}= 7.2 min. $[\alpha]_D^{25}$= +77.4 (c = 0.40, $CHCl_3$, 95% ee); **^{1}H NMR** (300 MHz, $CDCl_3$) δ 8.03 (d, *J* = 8.5 Hz, 1H), 7.66 (dd, *J* = 8.5, 1.9 Hz, 1H), 7.60 – 7.54 (m, 2H), 7.51 – 7.43 (m, 3H), 7.42 – 7.31 (m, 6H), 3.43 – 3.23 (m, 2H), 3.21 – 3.03 (m, 1H), 2.77 (ddd, *J* = 13.5, 11.5, 4.8 Hz, 1H), 1.66 (s, 9H). **^{13}C NMR** (75 MHz, $CDCl_3$) δ 175.4 (C), 148.9 (C), 139.9 (C), 138.9 (C), 138.8 (C), 137.5 (C), 129.4 (CH), 129.4 (C), 129.2 (CH), 128.6 (CH), 128.5 (CH), 127.9 (CH), 127.1 (CH), 126.8 (CH), 123.0 (CH), 116.3 (CH), 85.5 (C), 55.3 (C), 47.02 (d, *J* = 18.2 Hz, CH_2), 31.5 (CH_2), 28.2 (CH_3).**^{19}F NMR** (282 MHz, $CDCl_3$) δ 52.8. **HRMS (ESI-TOF)** calc'd for $[C_{27}H_{26}FNO_5S+NH_4]^+$: 513.1854; found: 513.1835.

X-ray structure, crystal data and structure refinement for compound 2e

Single crystal X-ray diffraction data was collected on Bruker D8 Venture diffractometer. Crystals suitable for X-ray single-crystal diffraction analysis were obtained through slowly evaporating the mixture of CH_2Cl_2 and n-hexane solution of **2e** under air at room temperature. The absolute configuration of compound **2e** is determined by anomalous dispersion with Mo Kα radiation (λ = 0.7093 Å) as X-ray source for X ray diffraction experiment, and a Flack parameter of 0.017(13) is obtained as result. CIF file for **2e** has been deposited at the Cambridge Crystallographic Data Centre with deposition number 2489843. of compound **2e.**

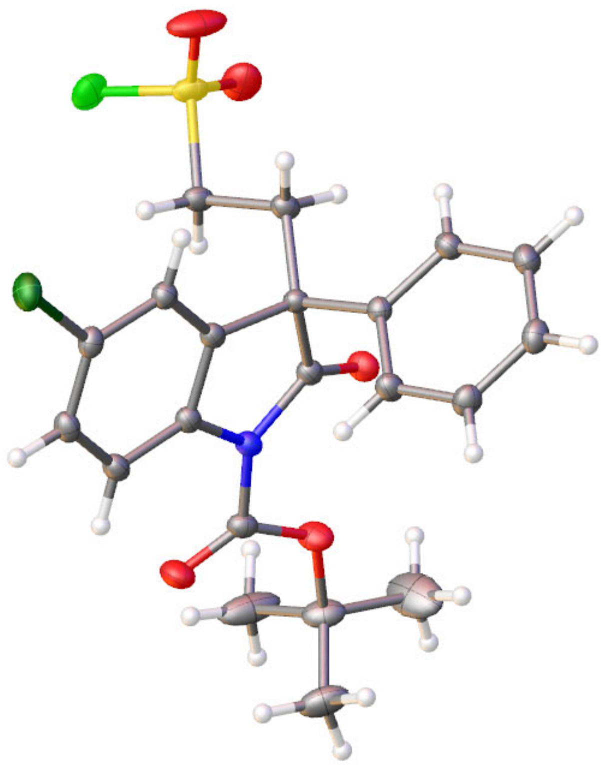

Figure S2. Ortep plot for the X-ray structure of compound **2e**. Thermal ellipsoids drawn at the 50% probability level. Flack parameter 0.017 (13).

Crystal data and structure refinement for 2e

Empirical formula	$C_{21} H_{21} Cl F N O_5 S$
Formula weight	453.90
Temperature	120K
Wavelength	0.71073 Å
Crystal system	Monoclinic
Space group	$P2_1$
Unit cell dimensions **2e**	a = 9.6791(4) Å α = 90º

	b = 10.6975(3) Å	β = 102.540(1)º
	c = 10.4383(4) Å	γ = 90º
Volume	1055.02(7) $Å^3$	
Z	4	
Density (calculated)	1.429 Mg/m^1	
F(000)	472	
Crystal size	1.340 x 0.130 x 0.130 mm^{-1}	
Final R indices [I>2sigma(I)]	R1= 0.0313	wR2 = 0.0670
Absolute structure parameter	0.017(13)	

NMR spectra of synthesized compounds

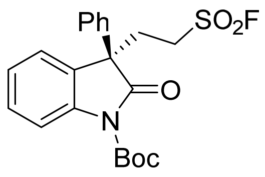

2a

^{1}H NMR (300 MHz, $CDCl_3$)

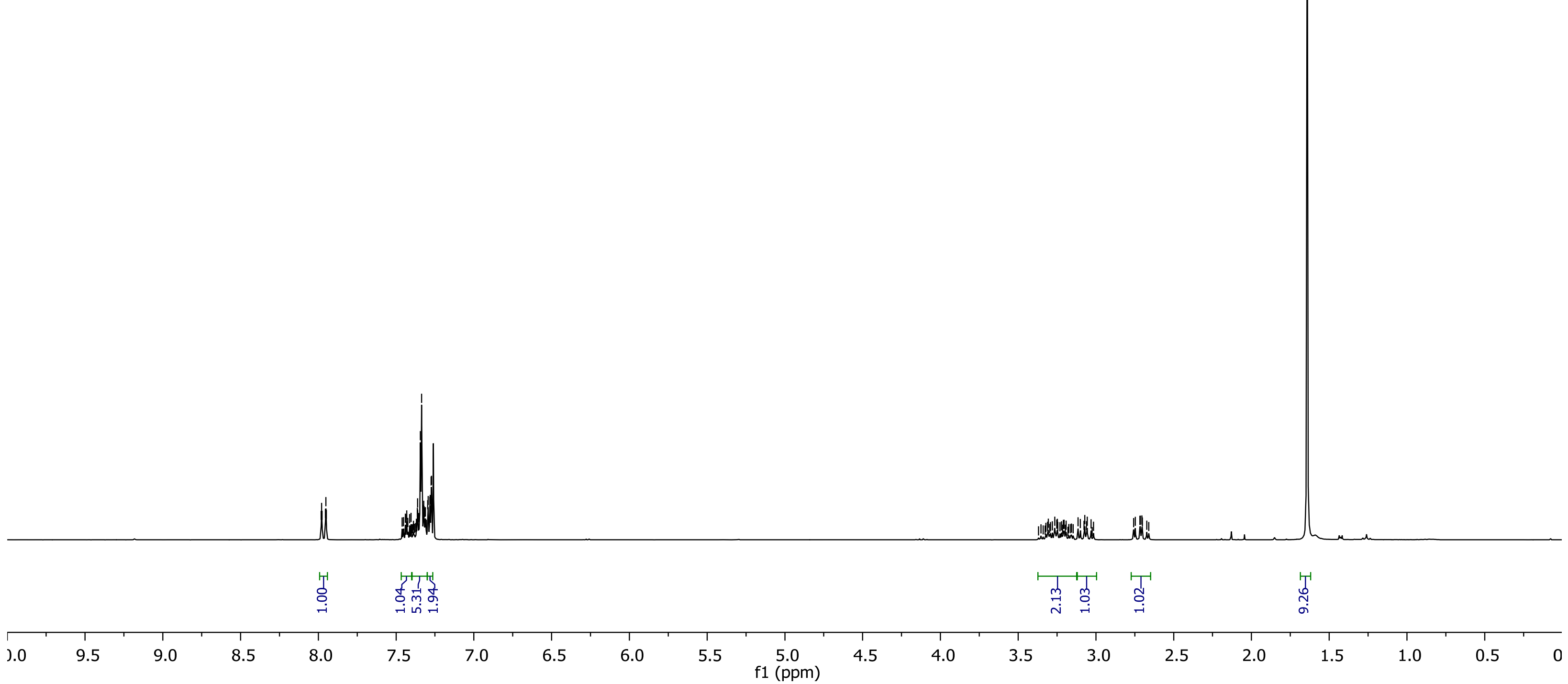

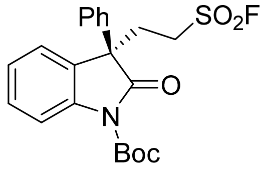

2a

^{13}C NMR (75 MHz, $CDCl_3$)

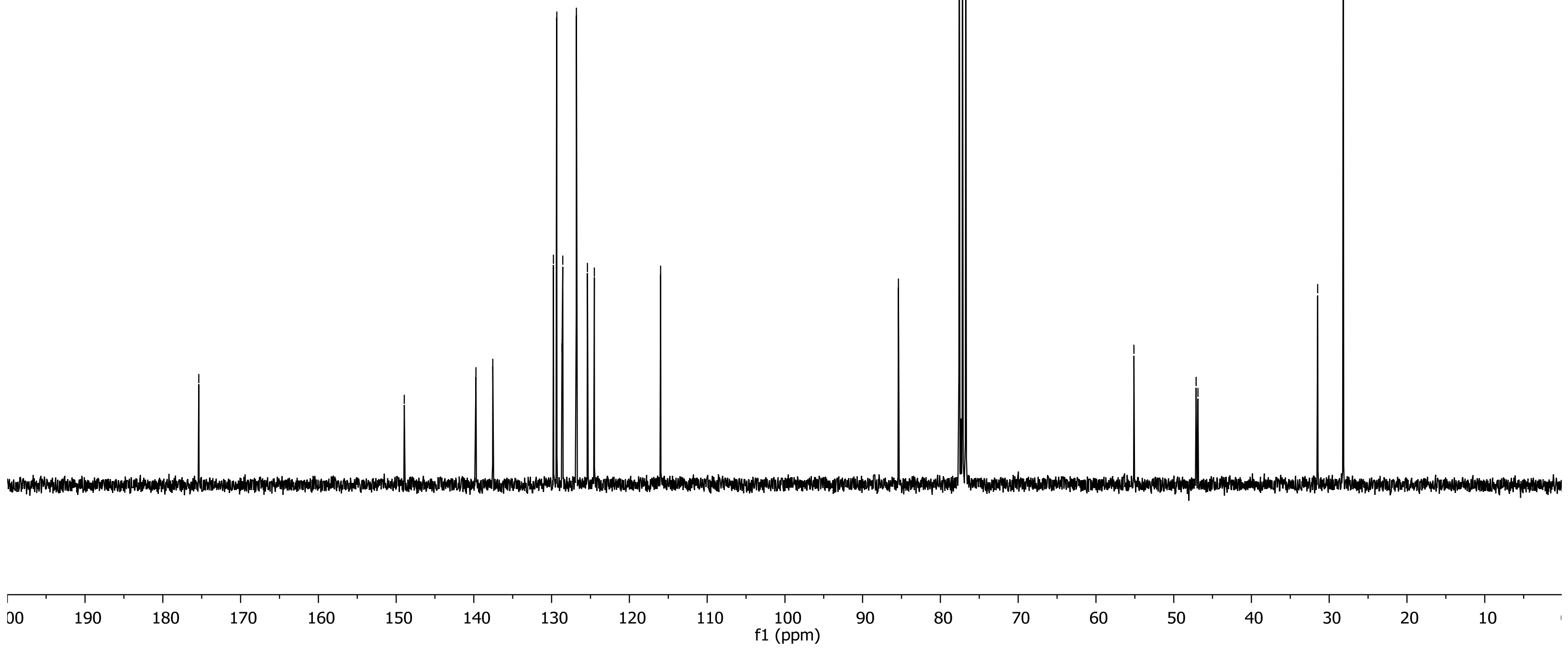

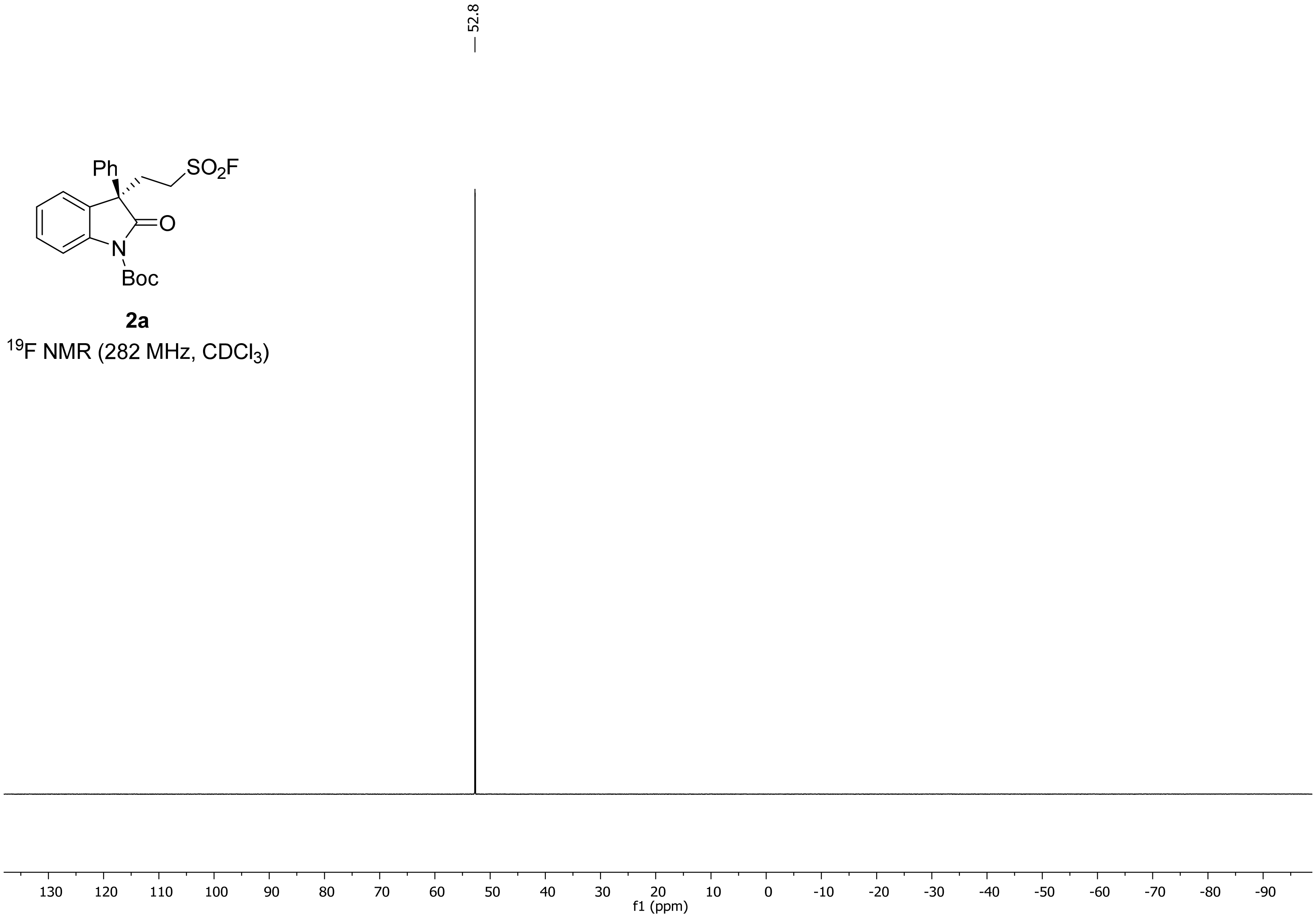

2a

^{19}F NMR (282 MHz, $CDCl_3$)

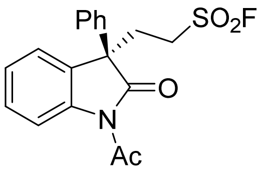

2b

^1H NMR (300 MHz, $CDCl_3$)

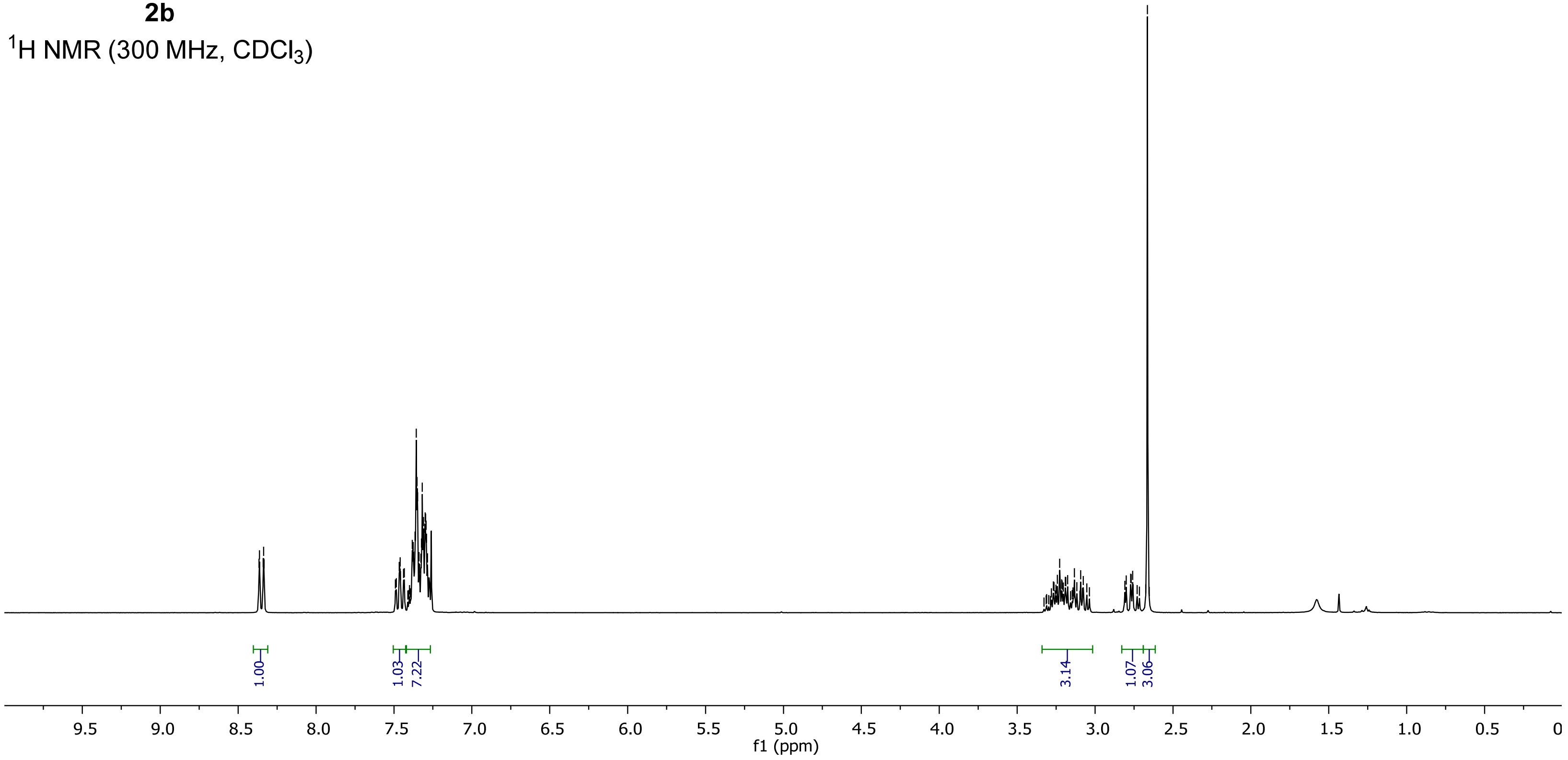

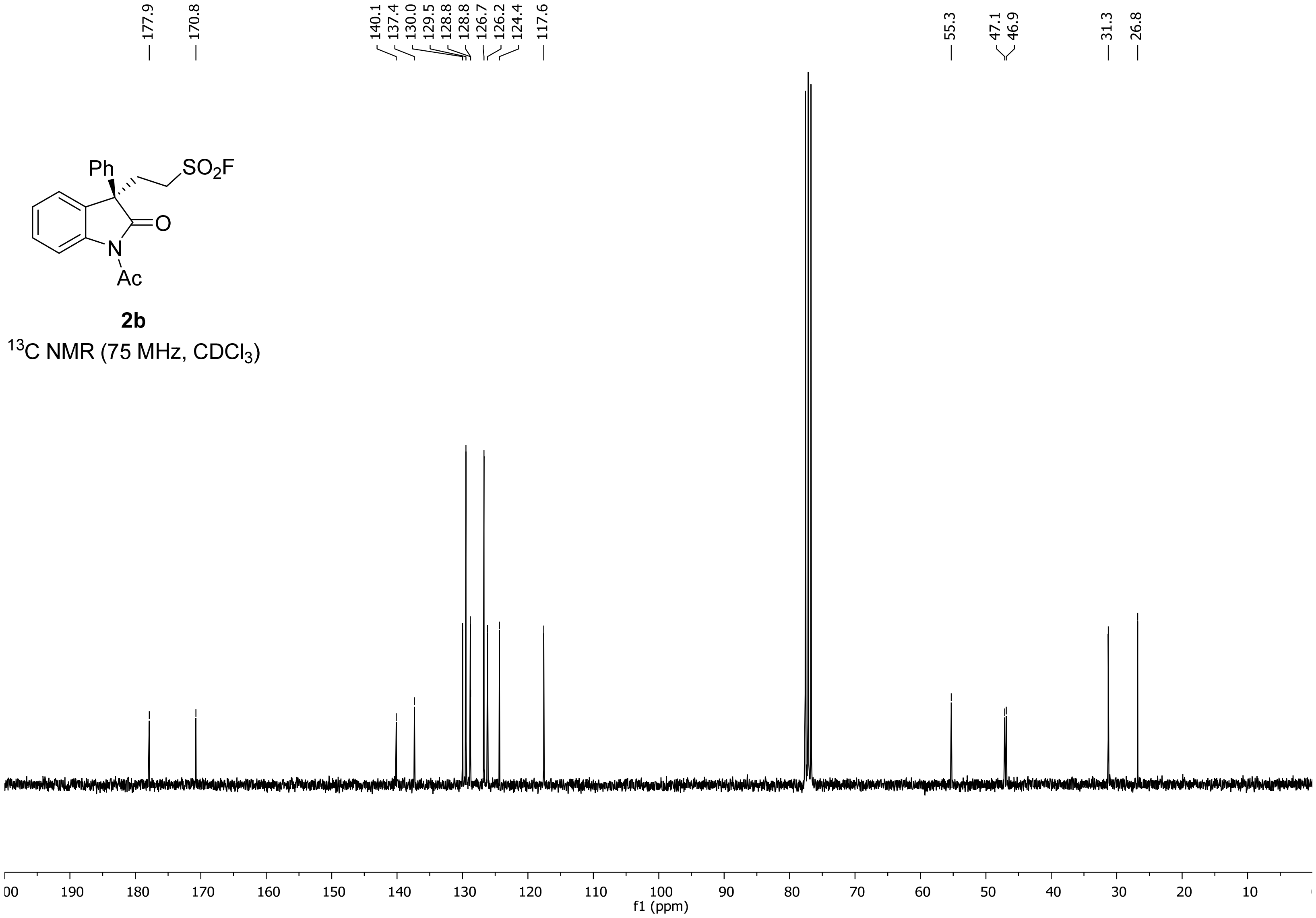

2b

^{13}C NMR (75 MHz, $CDCl_3$)

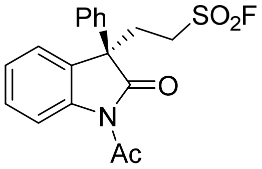

2b

^{19}F NMR (282 MHz, $CDCl_3$)

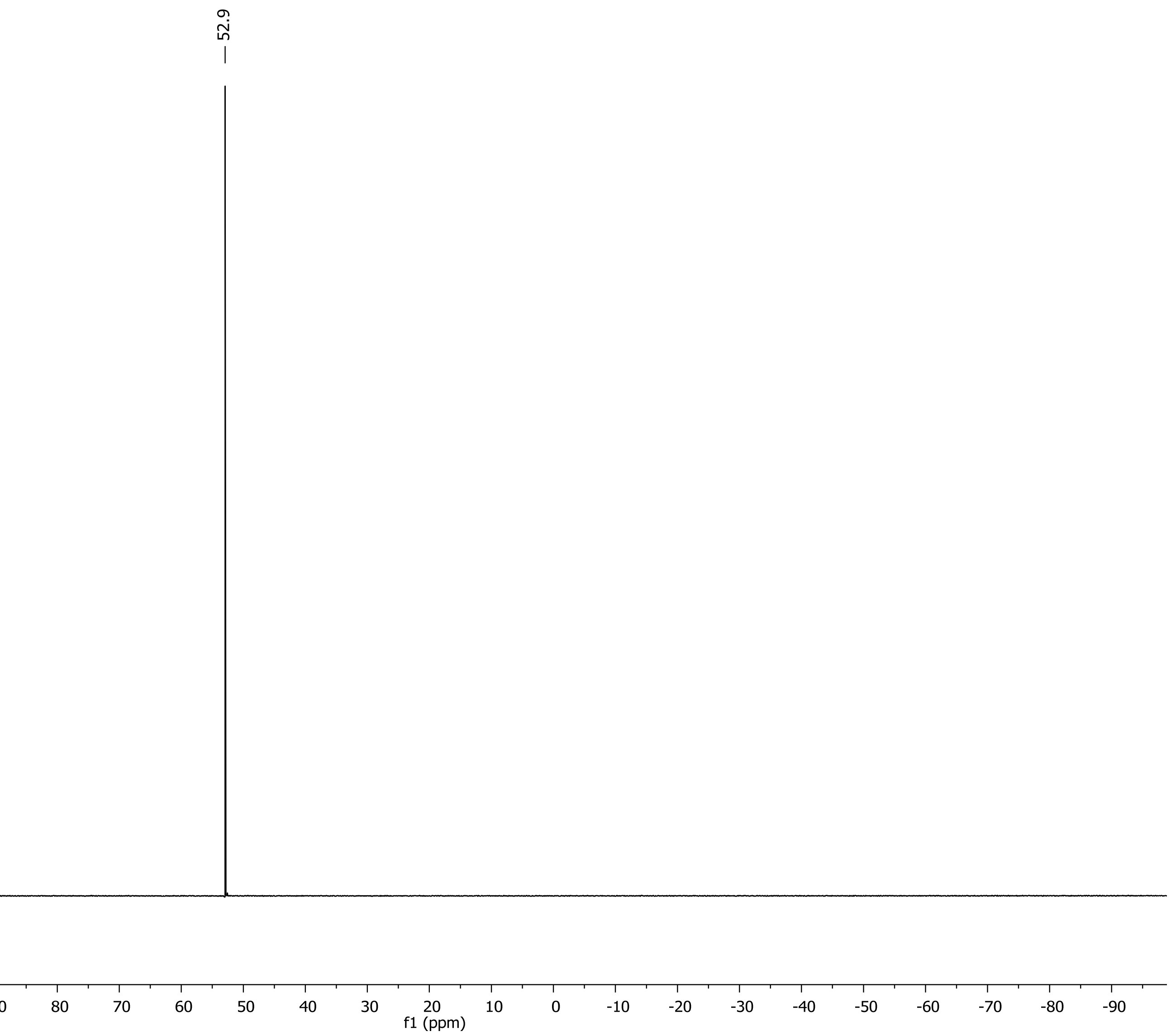

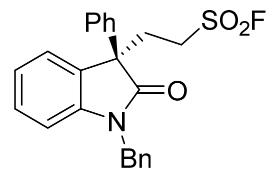

2c

^{1}H NMR (300 MHz, $CDCl_3$)

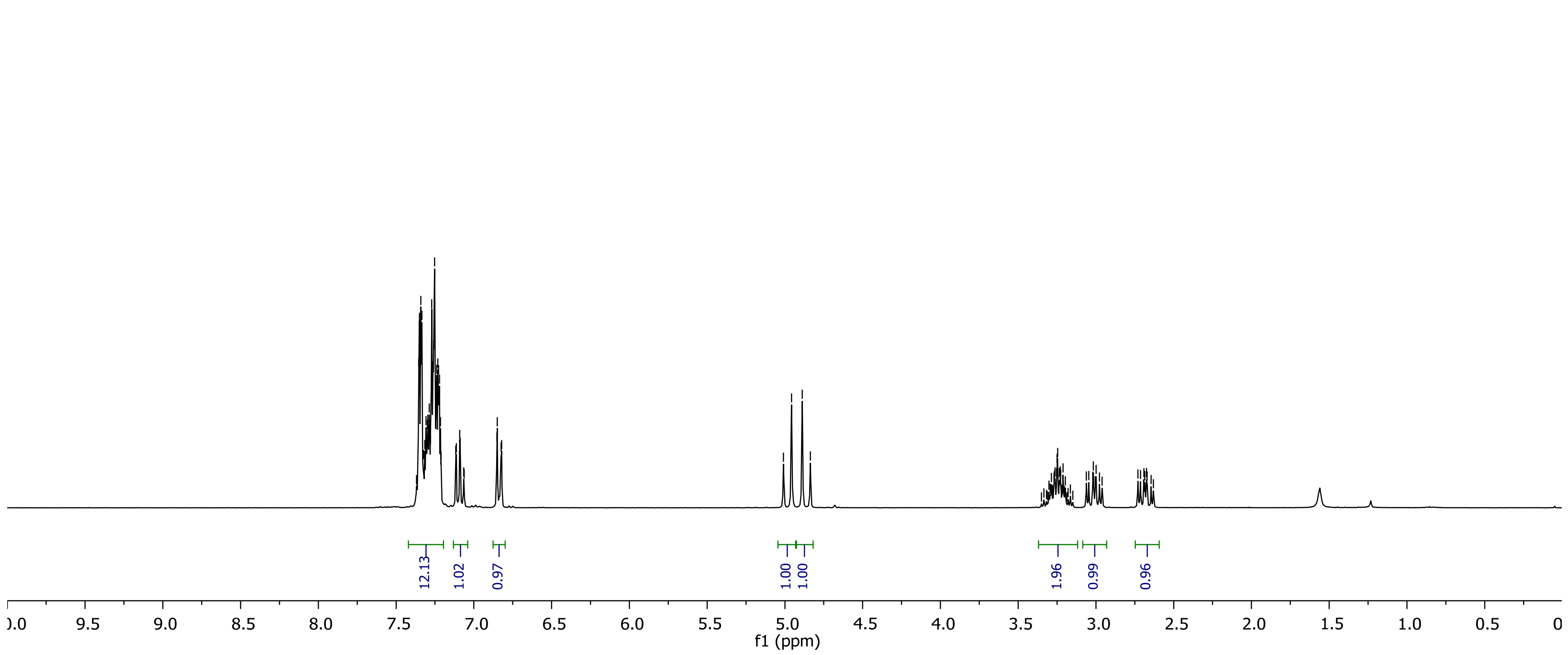

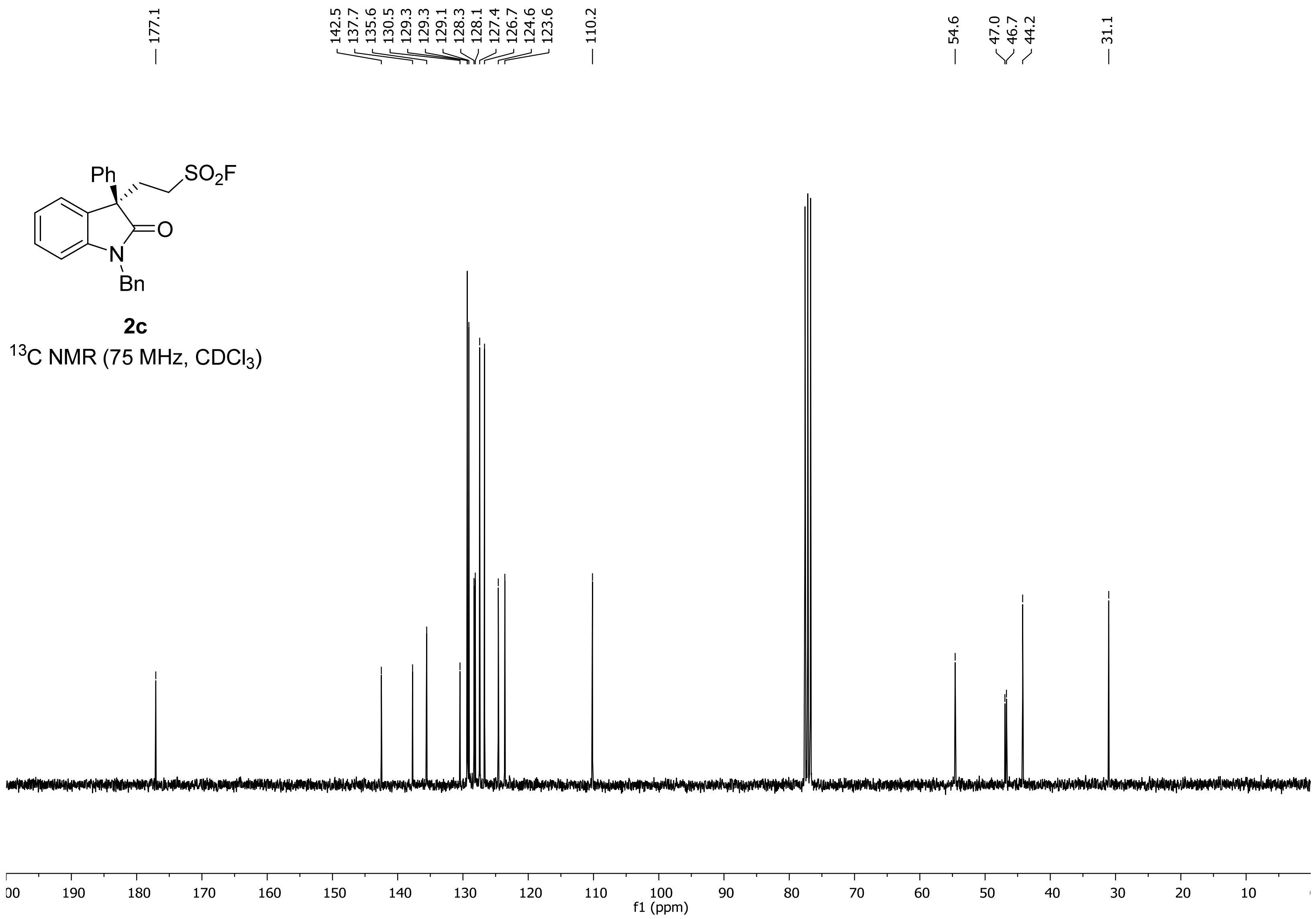

2c

^{13}C NMR (75 MHz, $CDCl_3$)

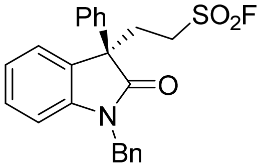

2c

^{19}F NMR (282 MHz, $CDCl_3$)

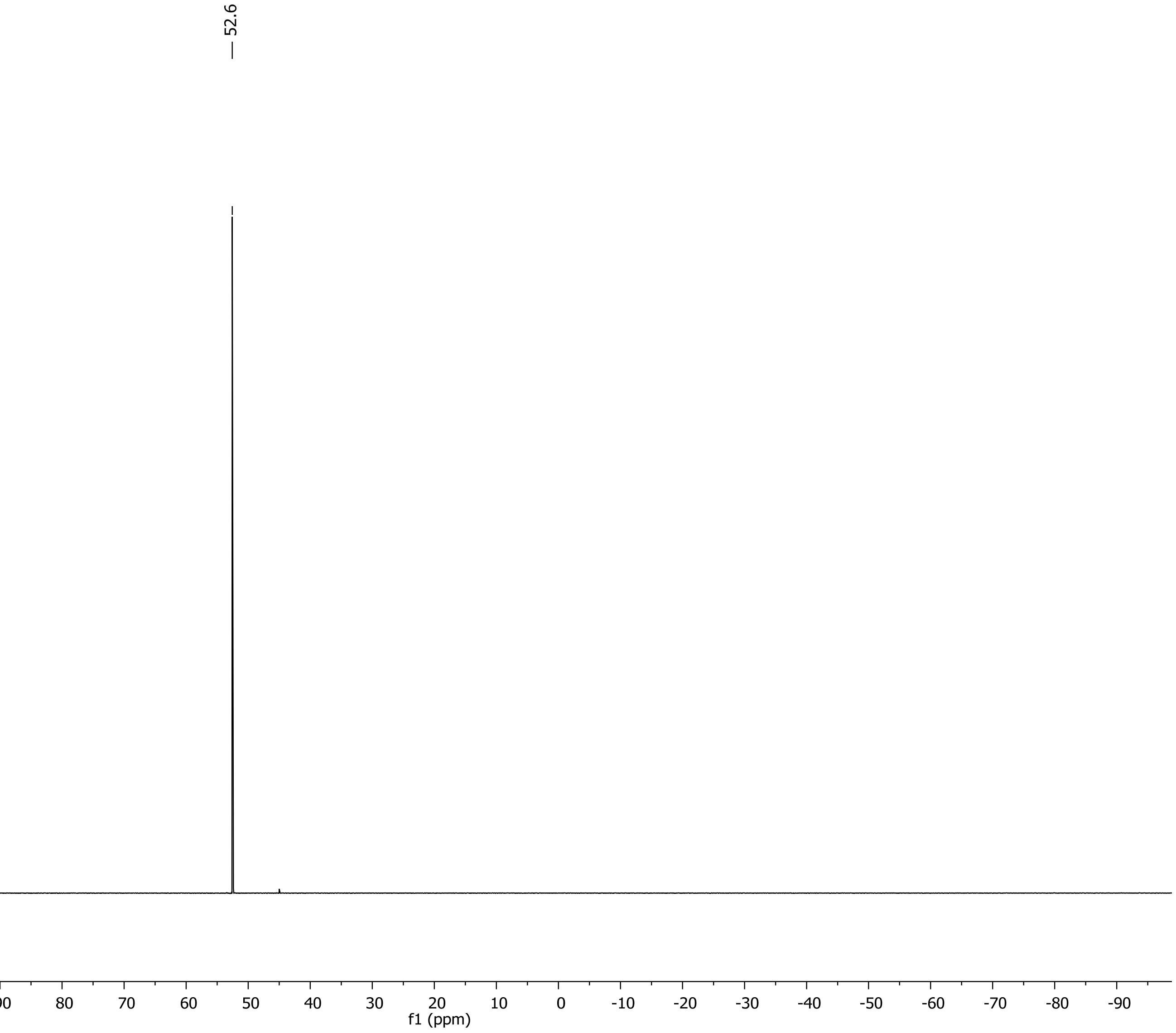

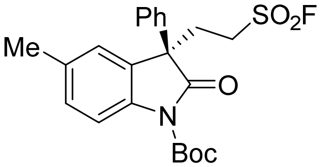

2d

^{1}H NMR (300 MHz, $CDCl_3$)

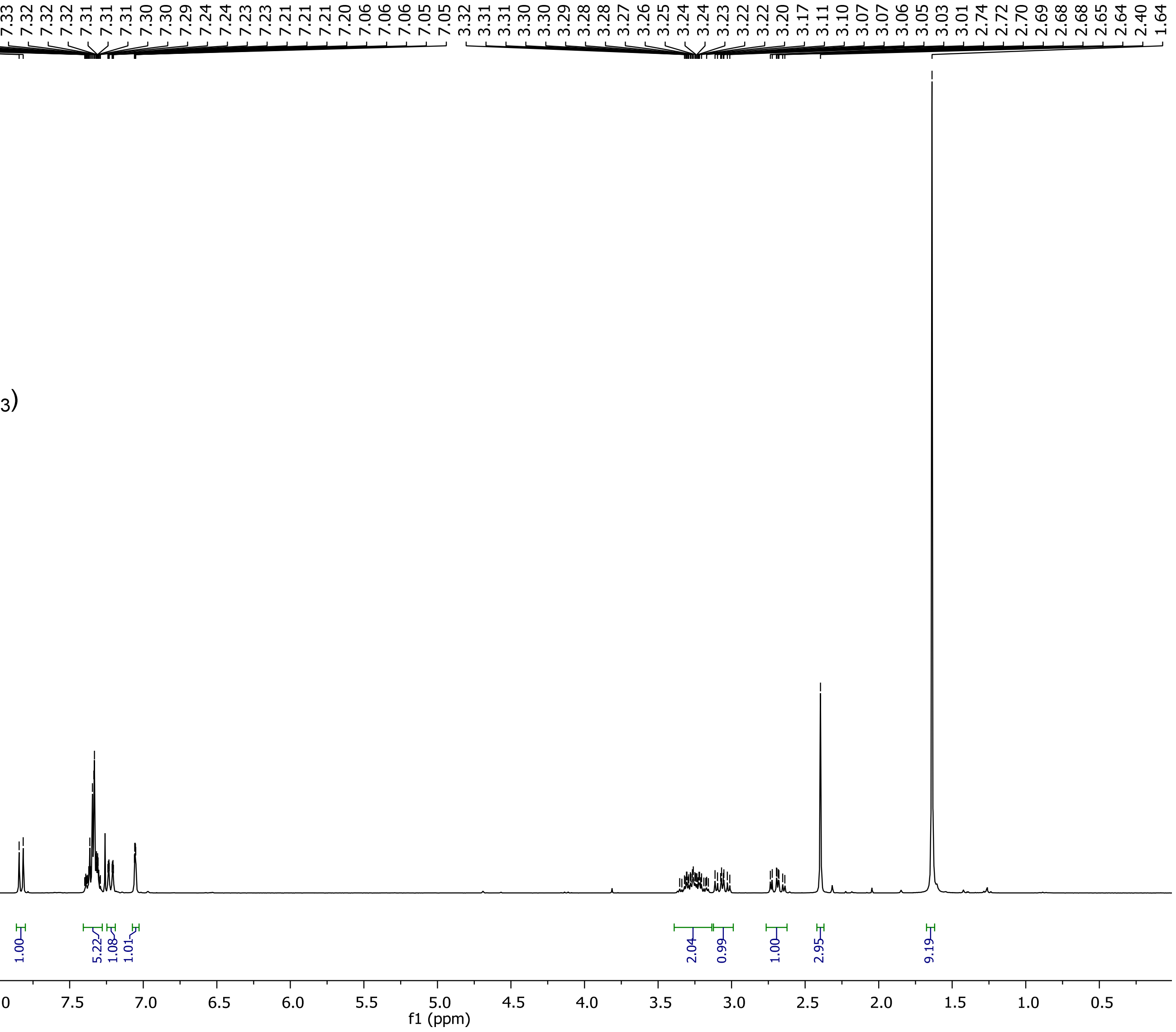

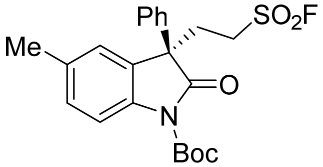

2d

^{13}C NMR (75 MHz, $CDCl_3$)

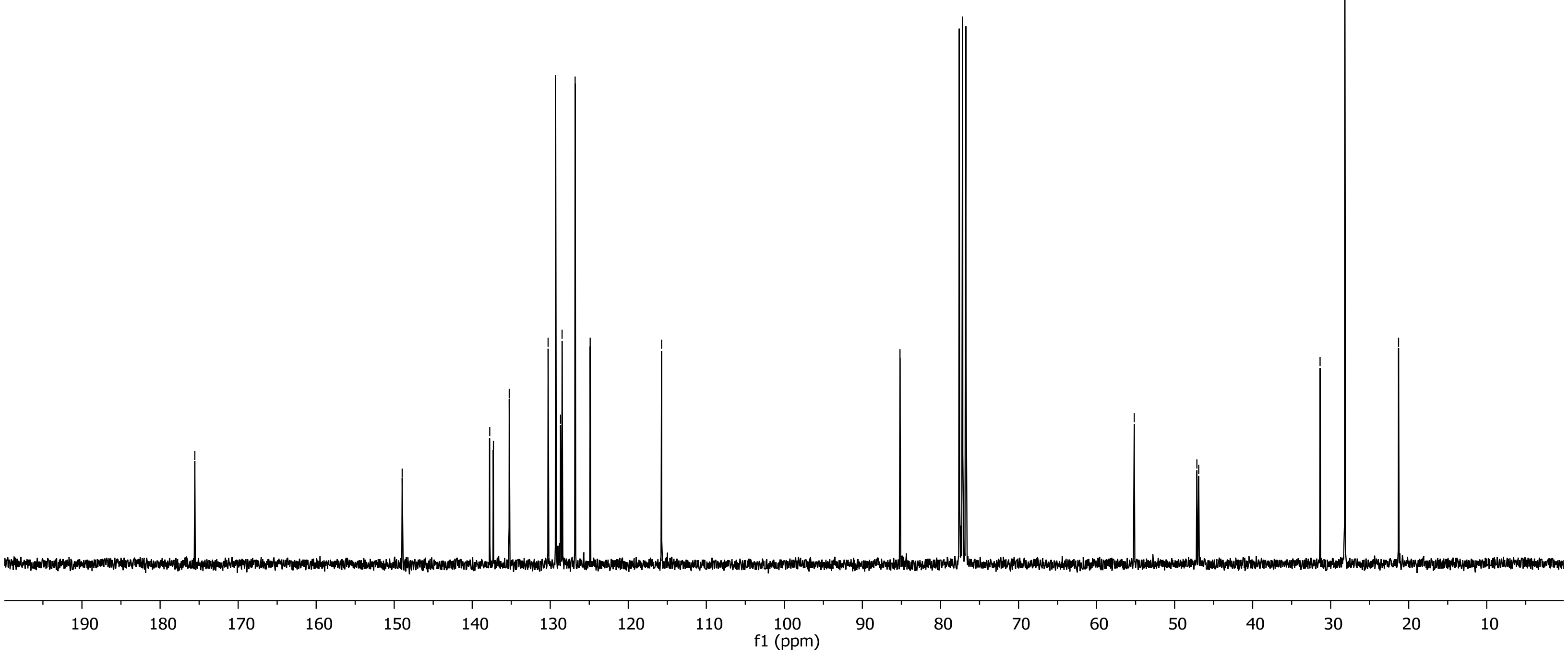

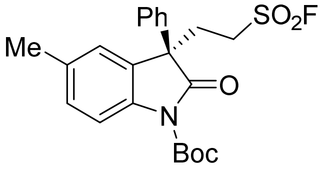

2d

^{19}F NMR (282 MHz, $CDCl_3$)

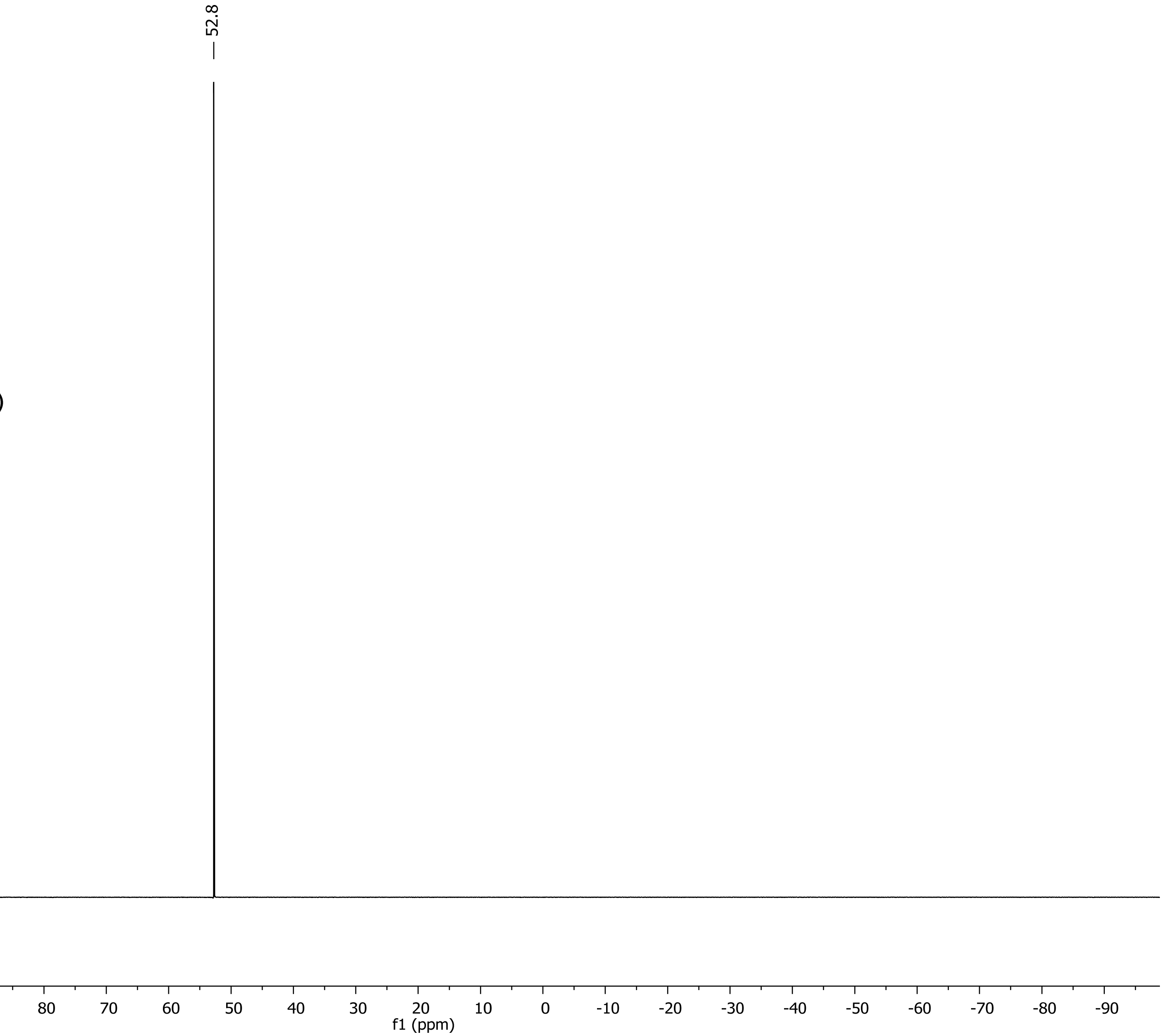

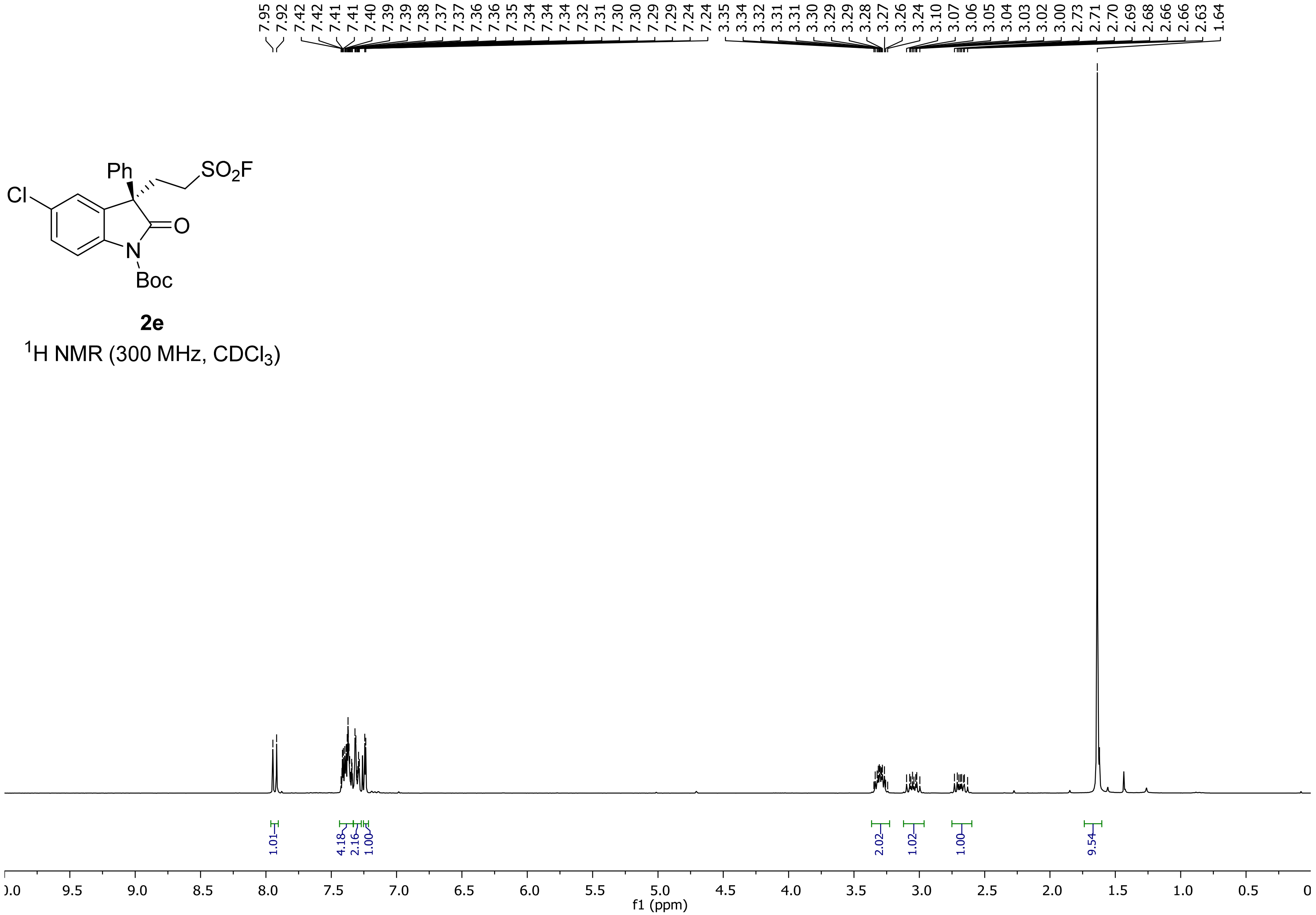

2e

^{1}H NMR (300 MHz, $CDCl_3$)

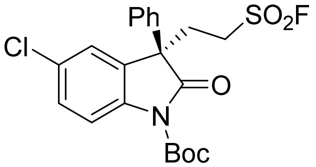

2e

^{13}C NMR (75 MHz, $CDCl_3$)

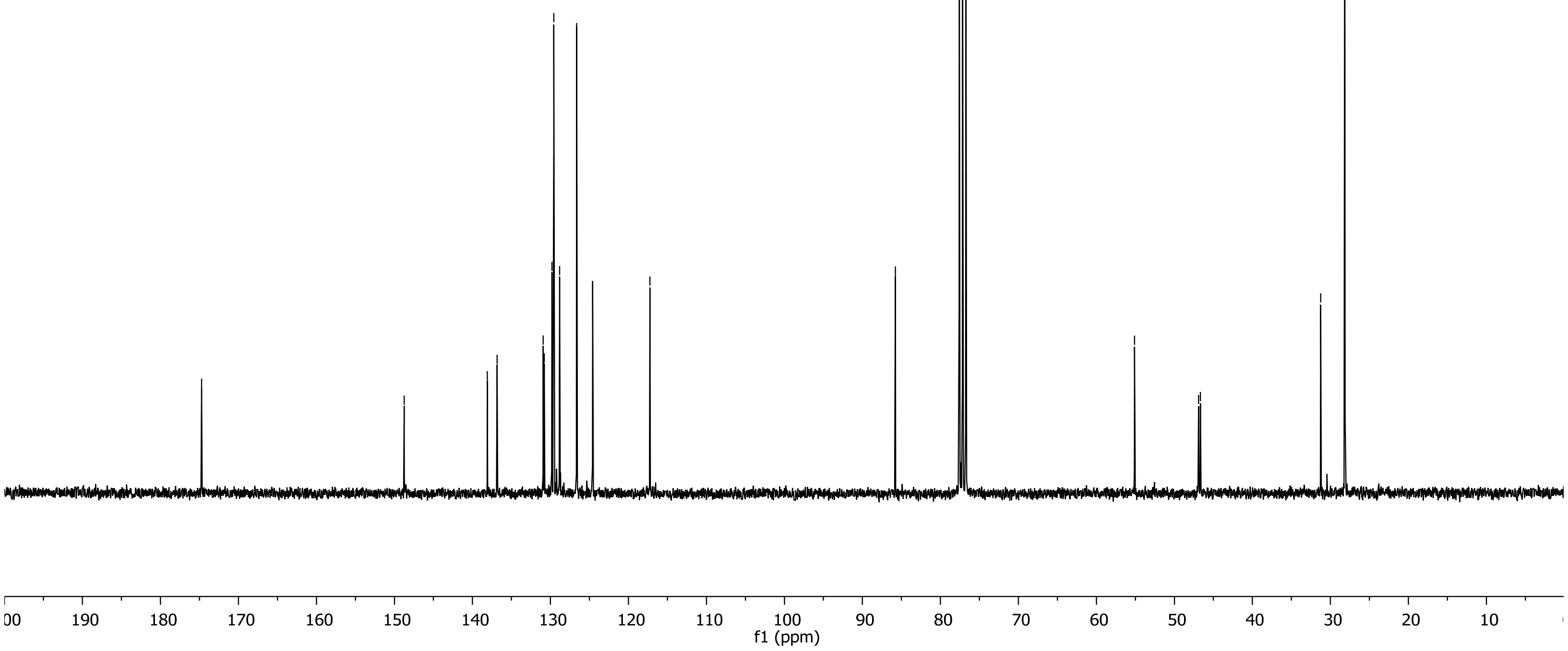

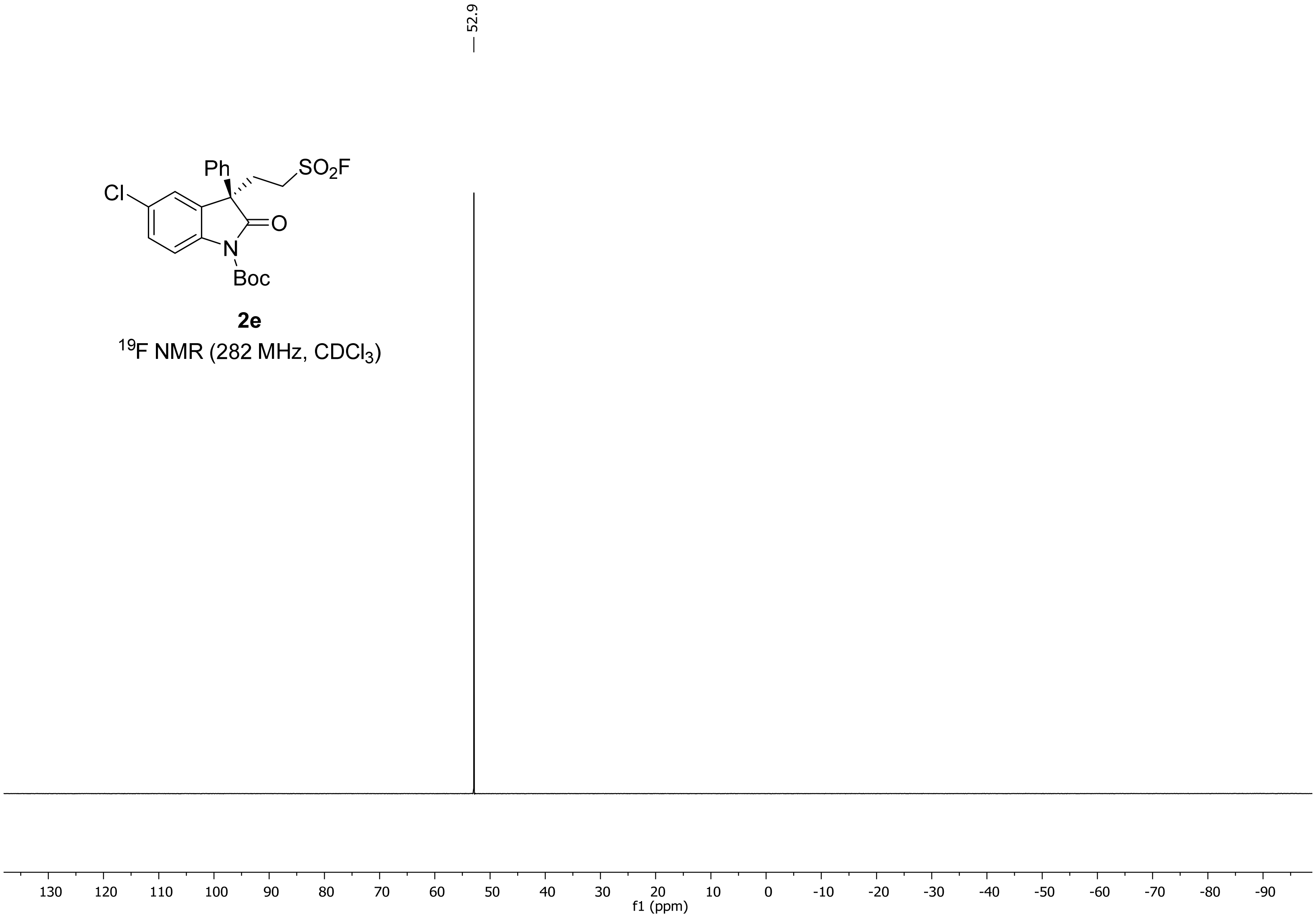

2e

^{19}F NMR (282 MHz, $CDCl_3$)

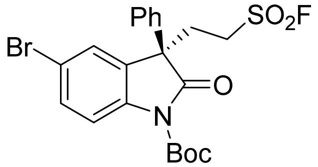

2f

^1H NMR (300 MHz, $CDCl_3$)

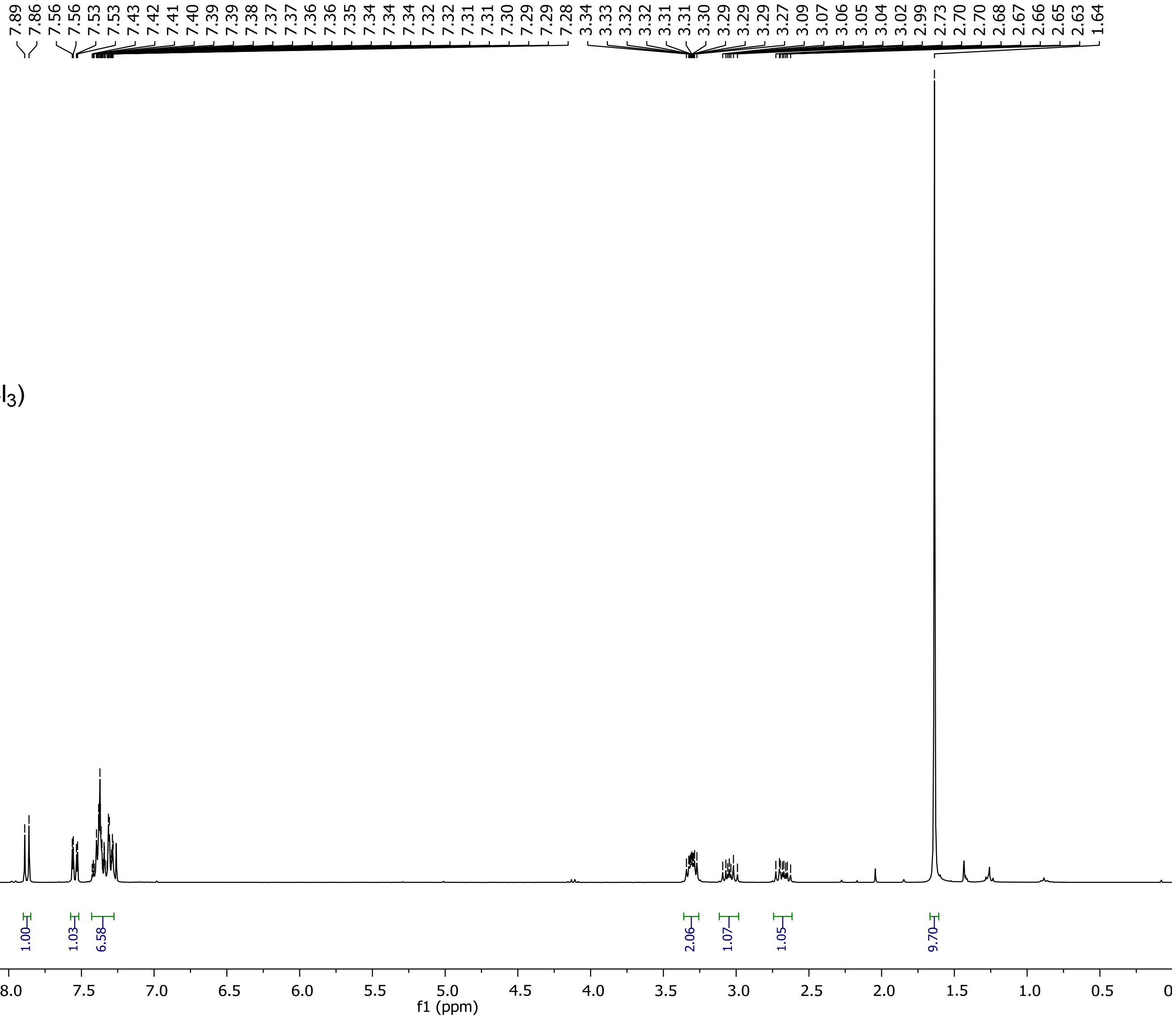

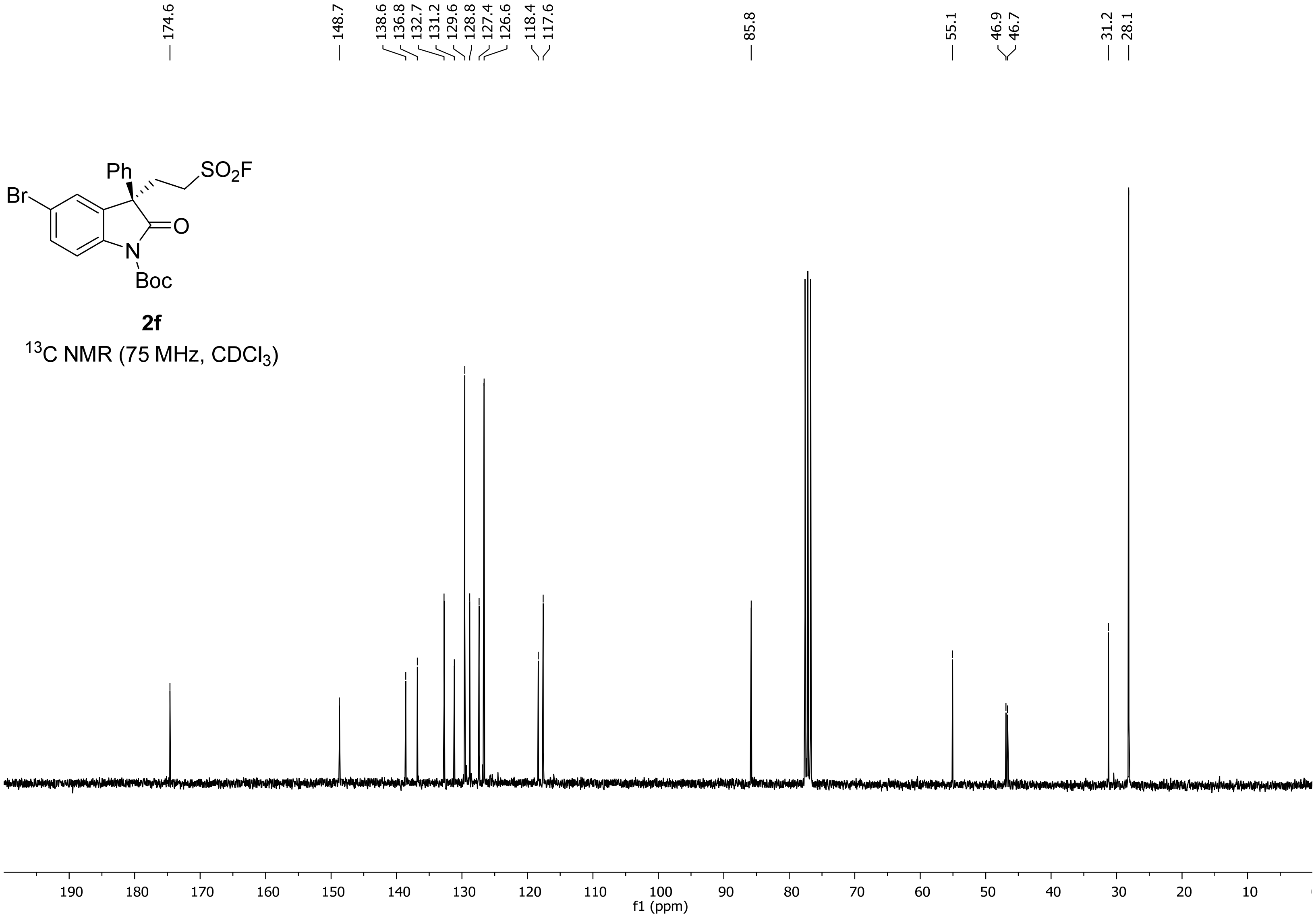
Ph
SO2F
Br
O
N
Boc
2f
13C NMR (75 MHz, CDCl3)
174.6
148.7
138.6
136.8
132.7
131.2
129.6
128.8
127.4
126.6
118.4
117.6
85.8
55.1
46.9
46.7
31.2
28.1
190 180 170 160 150 140 130 120 110 100 90 80 70 60 50 40 30 20 10
f1 (ppm)

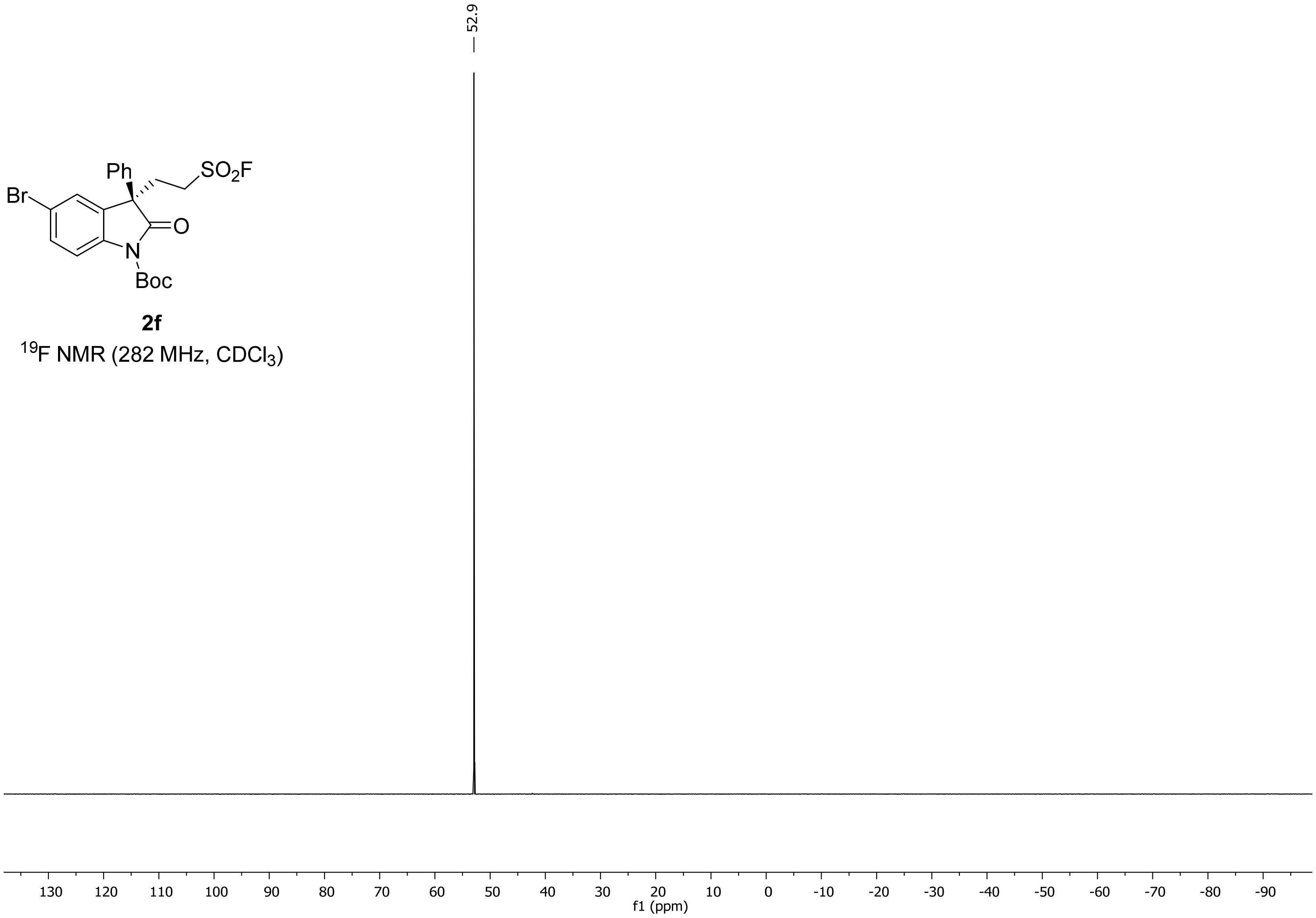

2f

^{19}F NMR (282 MHz, $CDCl_3$)

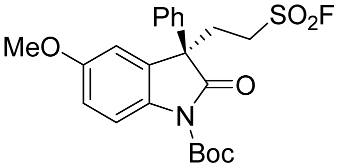

2g

^{1}H NMR (300 MHz, $CDCl_3$)

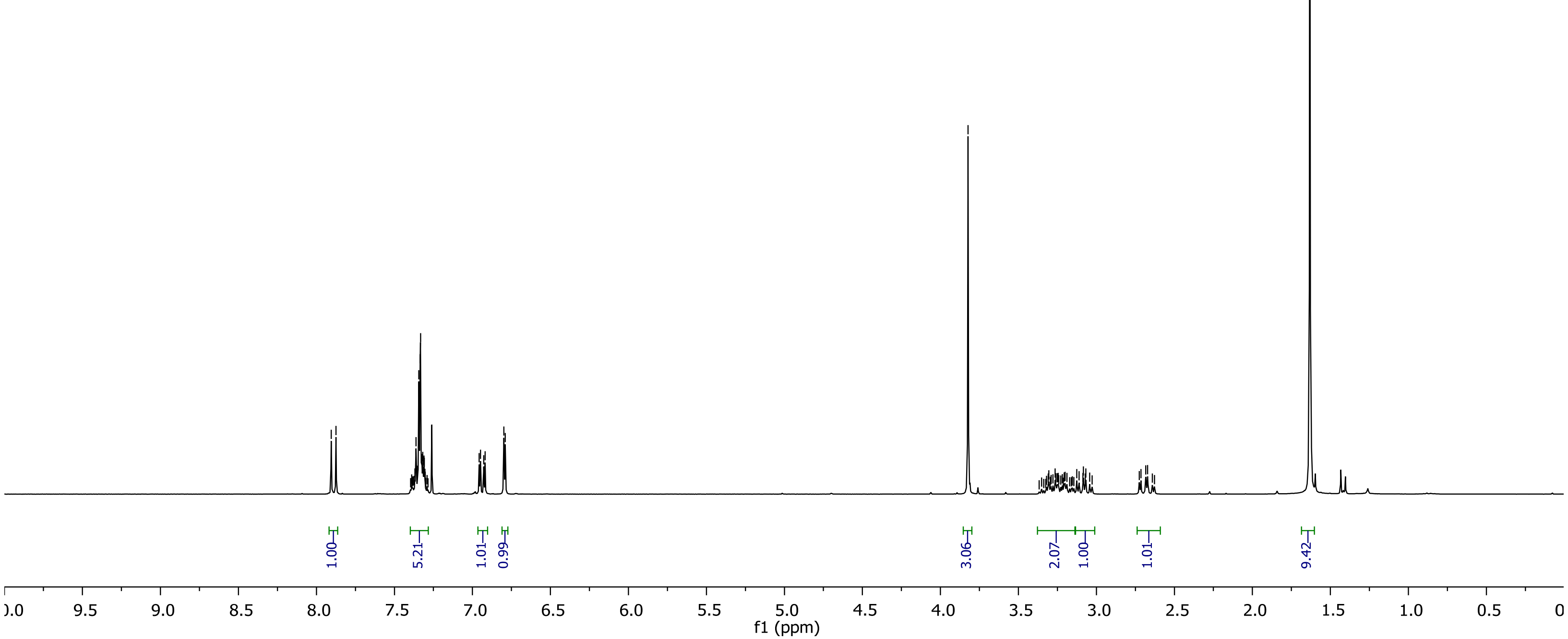

2g

^{13}C NMR (75 MHz, $CDCl_3$)

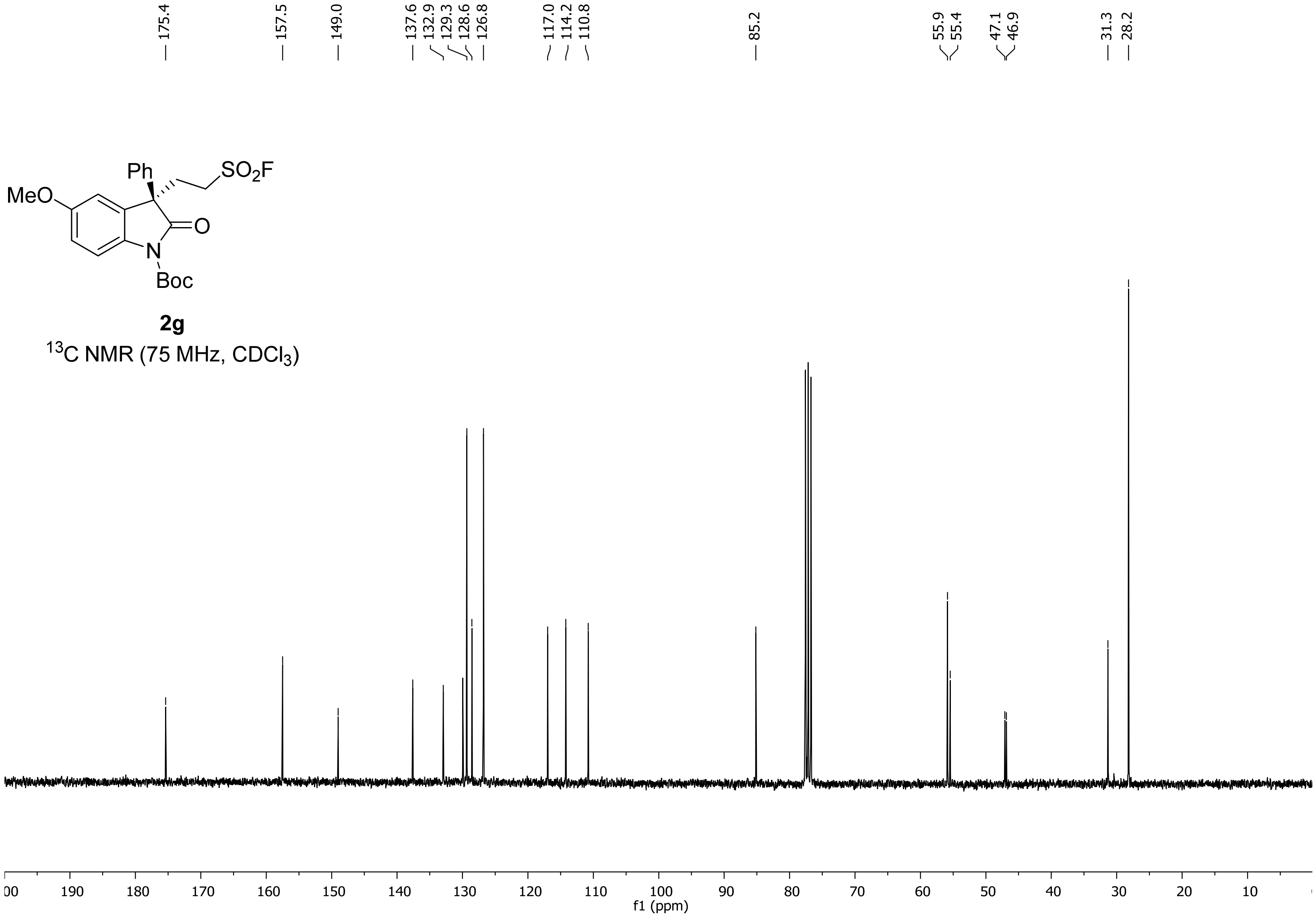

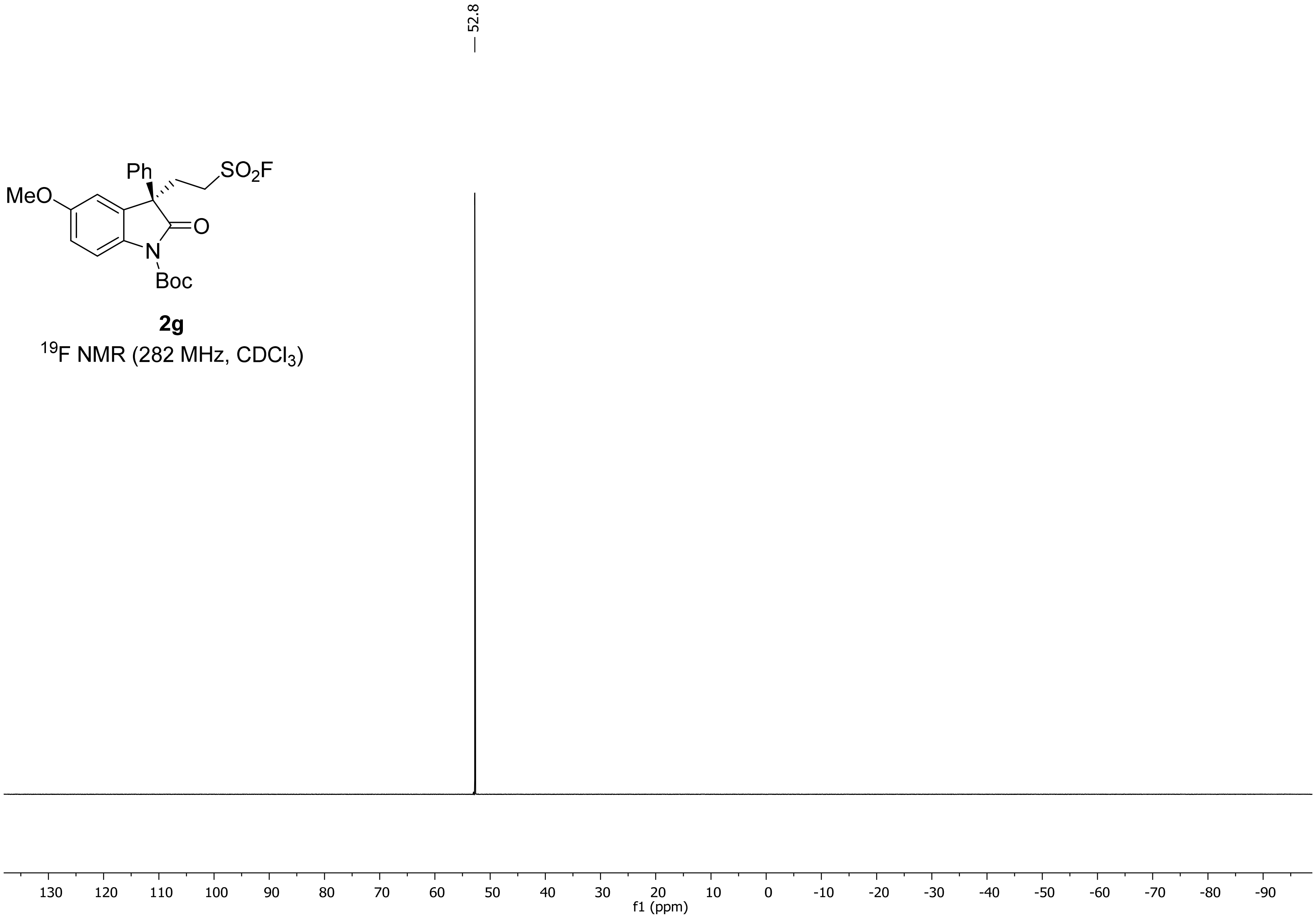

2g

^{19}F NMR (282 MHz, $CDCl_3$)

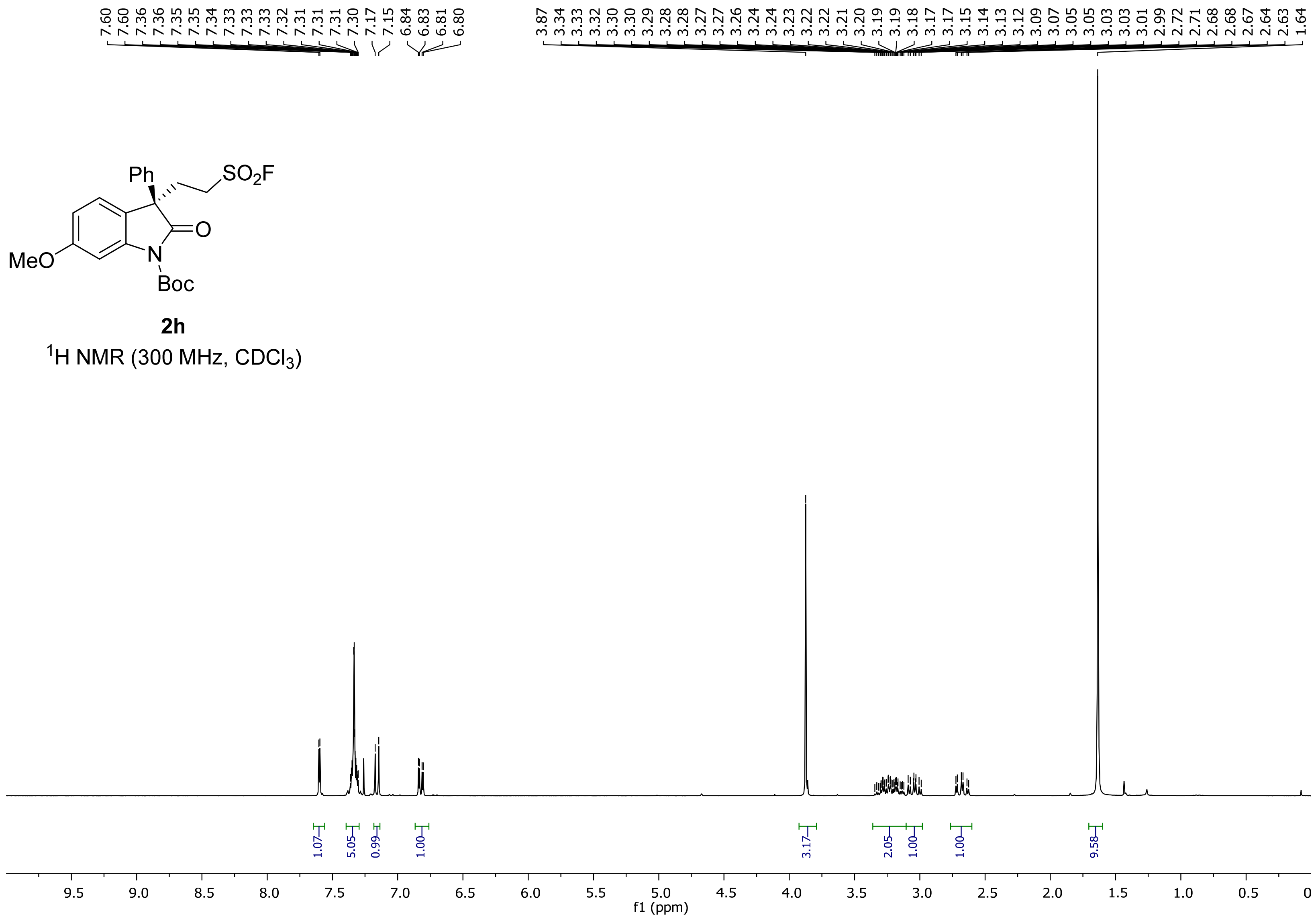

2h

^1H NMR (300 MHz, $CDCl_3$)

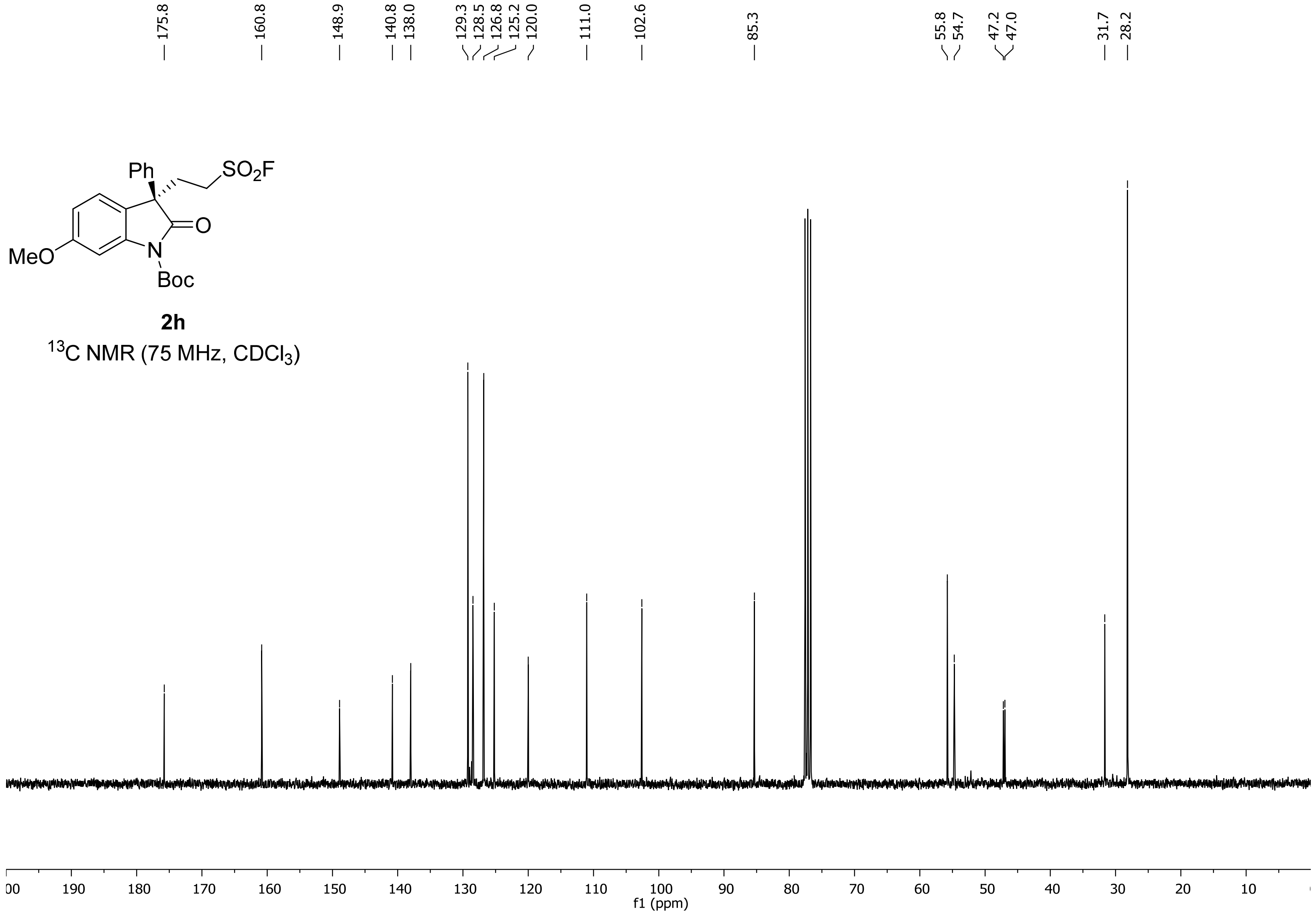

2h

^{13}C NMR (75 MHz, $CDCl_3$)

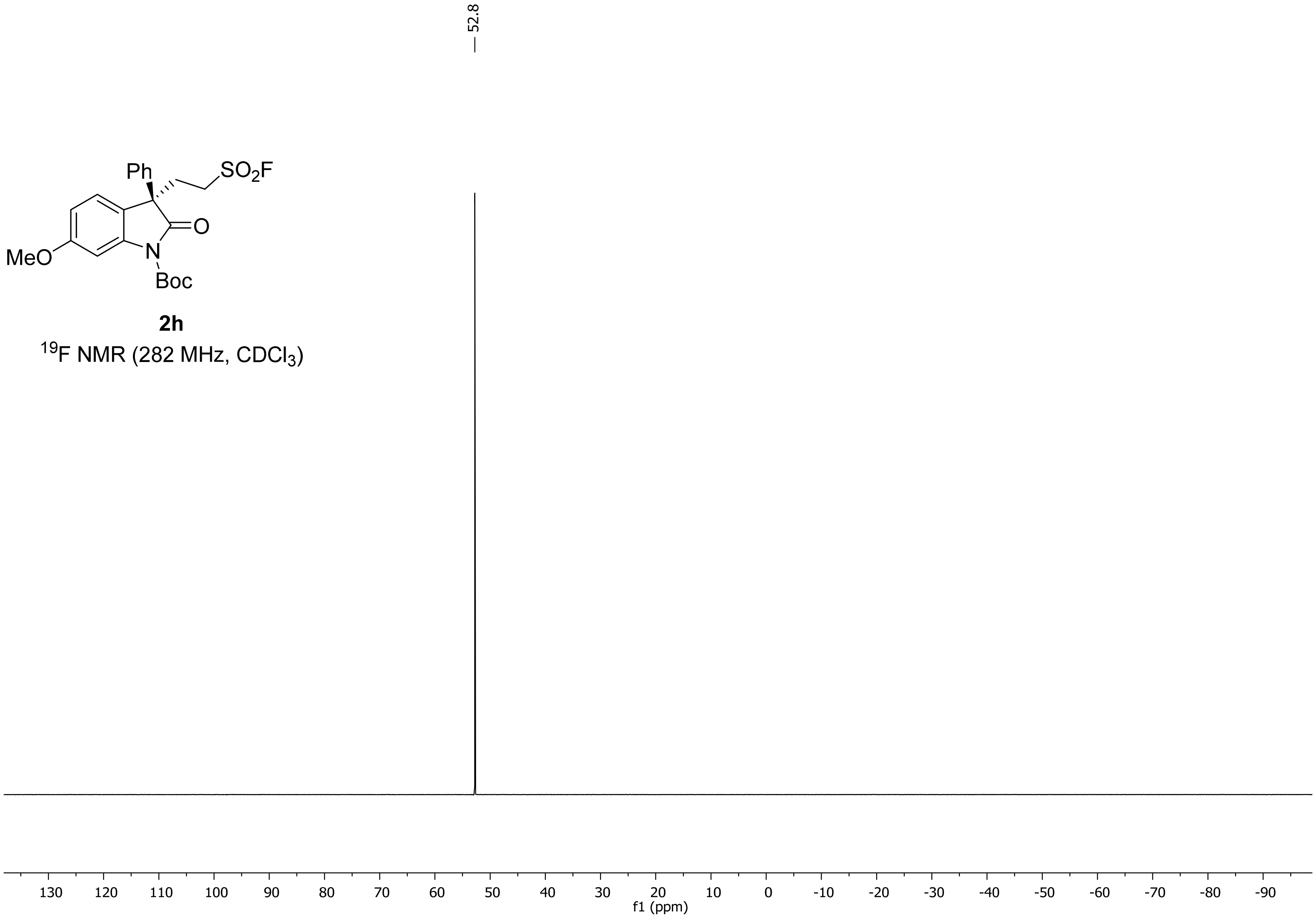

2h

^{19}F NMR (282 MHz, $CDCl_3$)

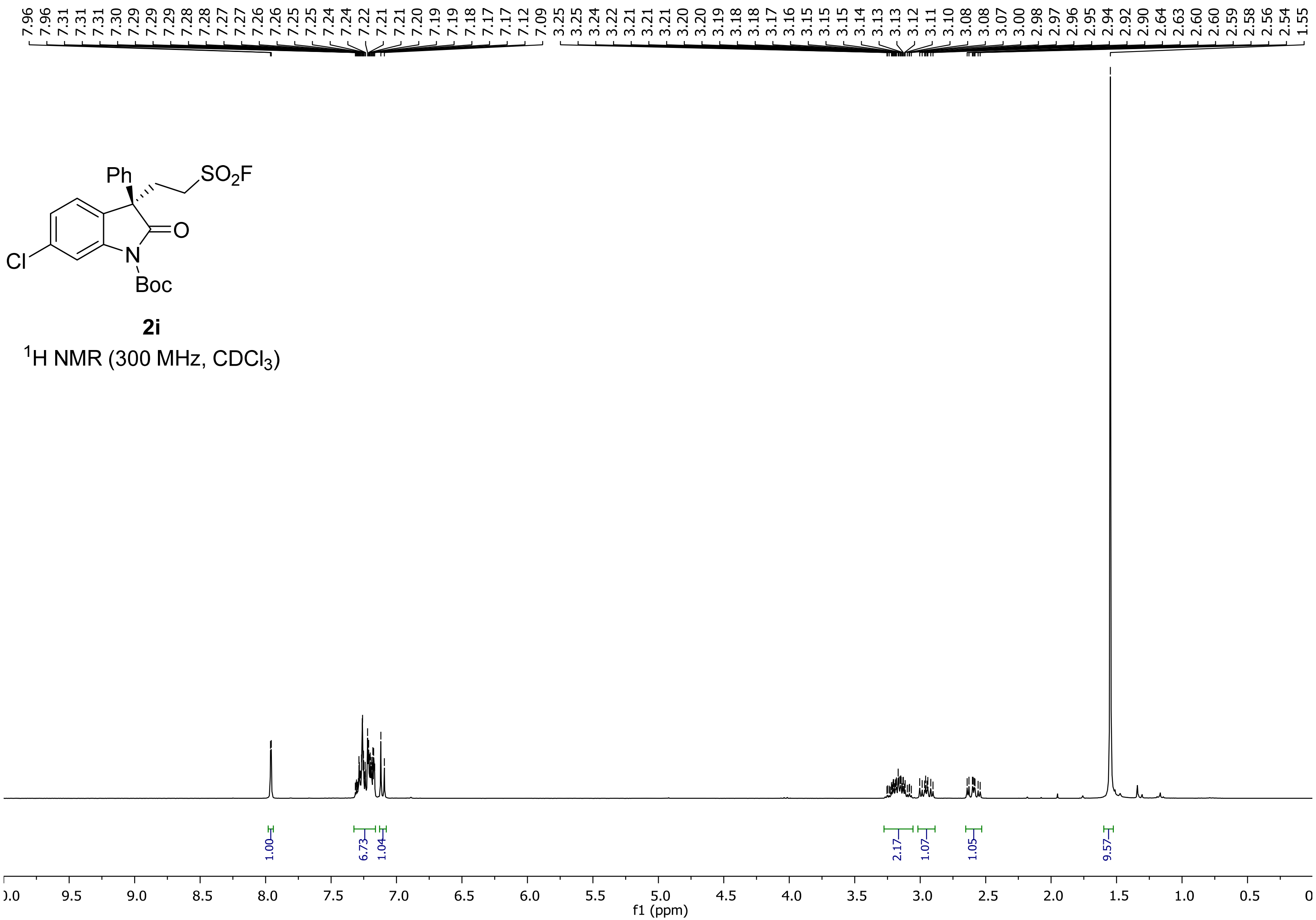
7.96 7.96 7.31 7.31 7.31 7.30 7.29 7.29 7.29 7.28 7.28 7.27 7.27 7.26 7.26 7.25 7.25 7.24 7.24 7.22 7.21 7.21 7.20 7.19 7.19 7.18 7.17 7.17 7.12 7.09 3.25 3.25 3.24 3.22 3.21 3.21 3.21 3.20 3.20 3.19 3.18 3.18 3.17 3.16 3.15 3.15 3.15 3.14 3.13 3.13 3.12 3.11 3.10 3.08 3.08 3.07 3.00 2.98 2.97 2.96 2.95 2.94 2.92 2.90 2.64 2.63 2.60 2.60 2.59 2.58 2.56 2.54 1.55
Ph
SO2F
O
N
Cl
Boc
2i
1H NMR (300 MHz, CDCl3)
1.00
6.73
1.04
2.17
1.07
1.05
9.57
f1 (ppm)

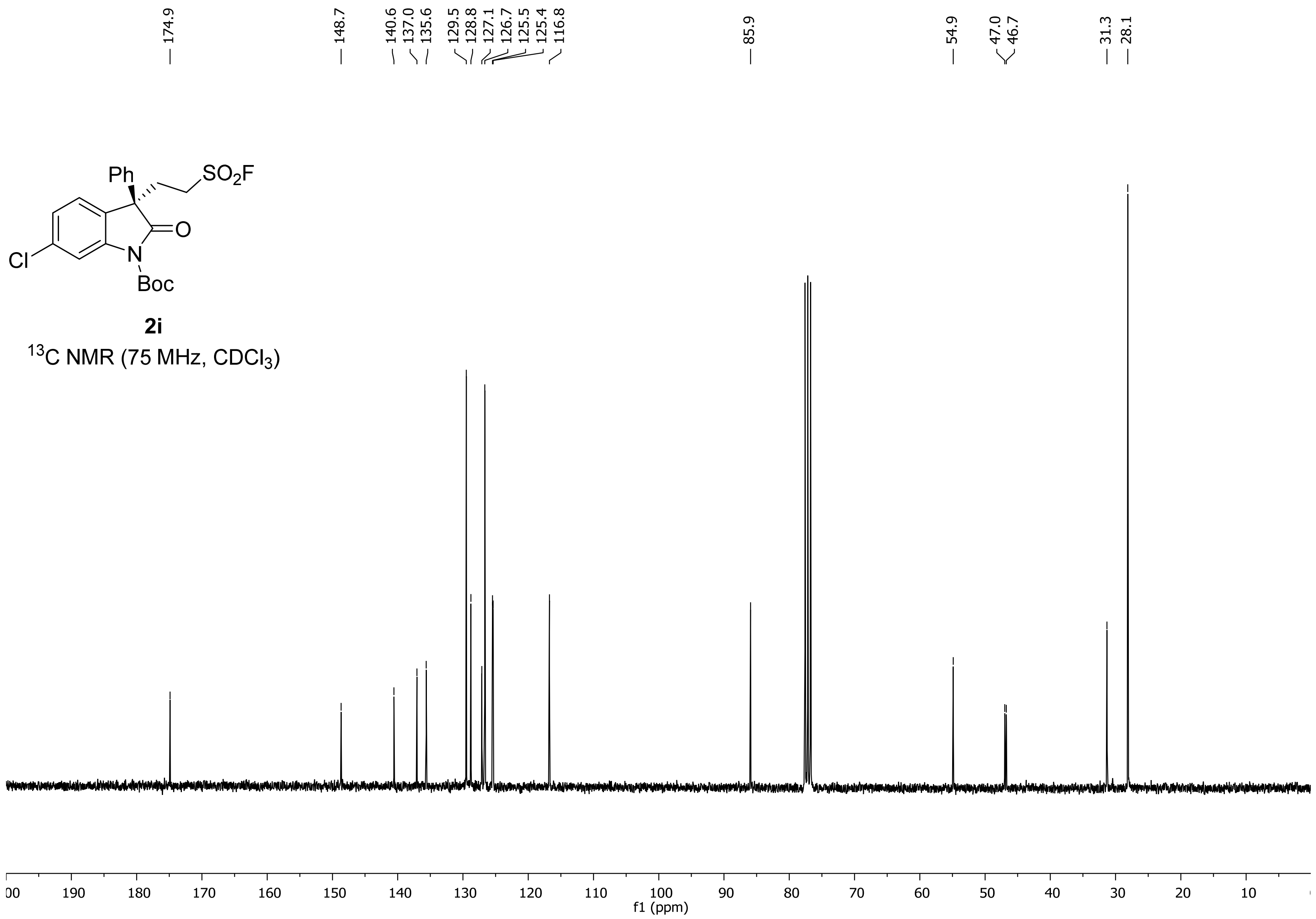

2i

^{13}C NMR (75 MHz, $CDCl_3$)

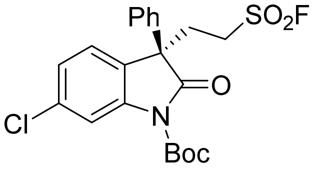

2i

^{19}F NMR (282 MHz, $CDCl_3$)

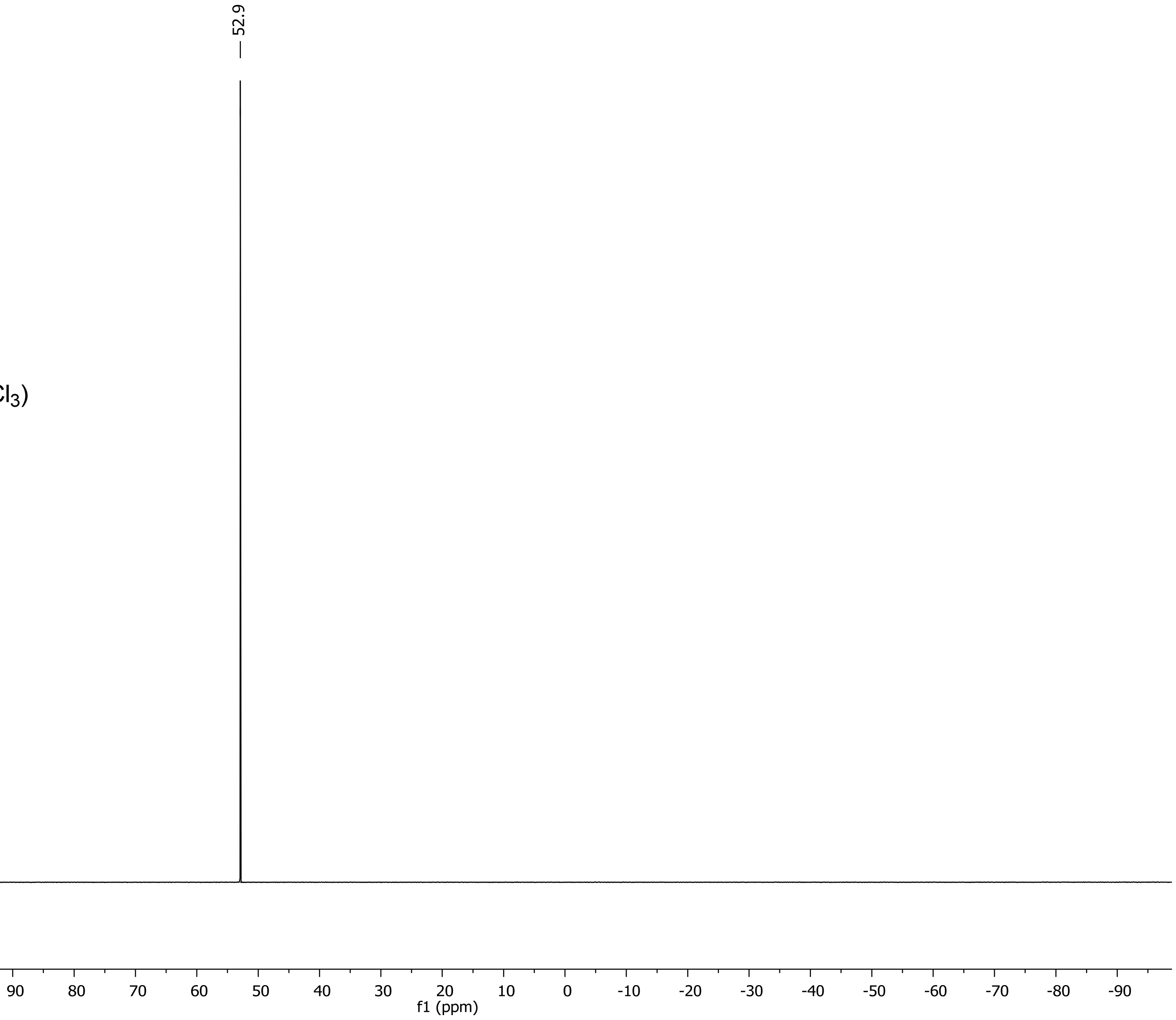

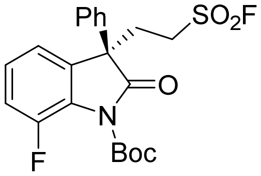

2j

^{1}H NMR (300 MHz, $CDCl_3$)

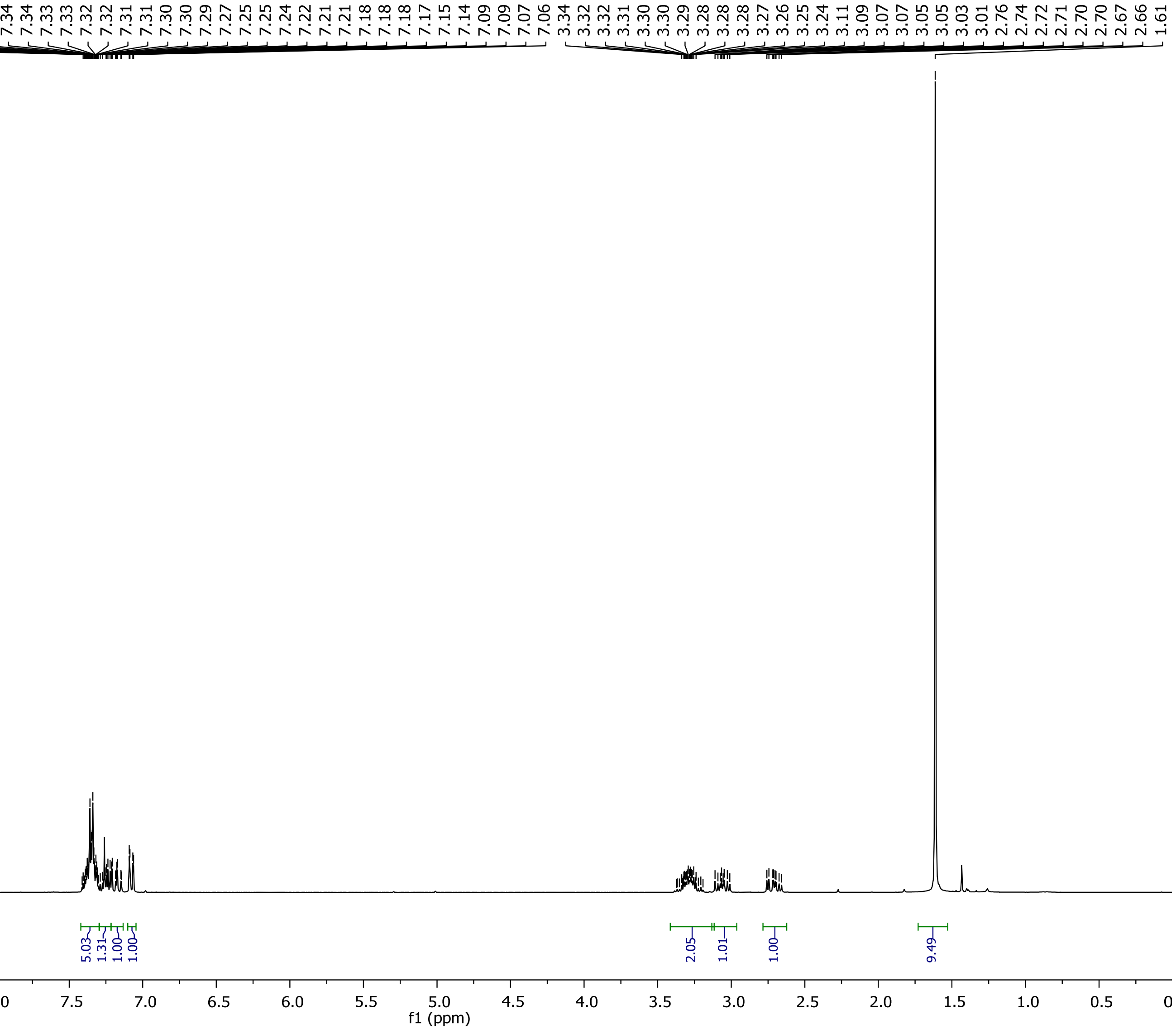

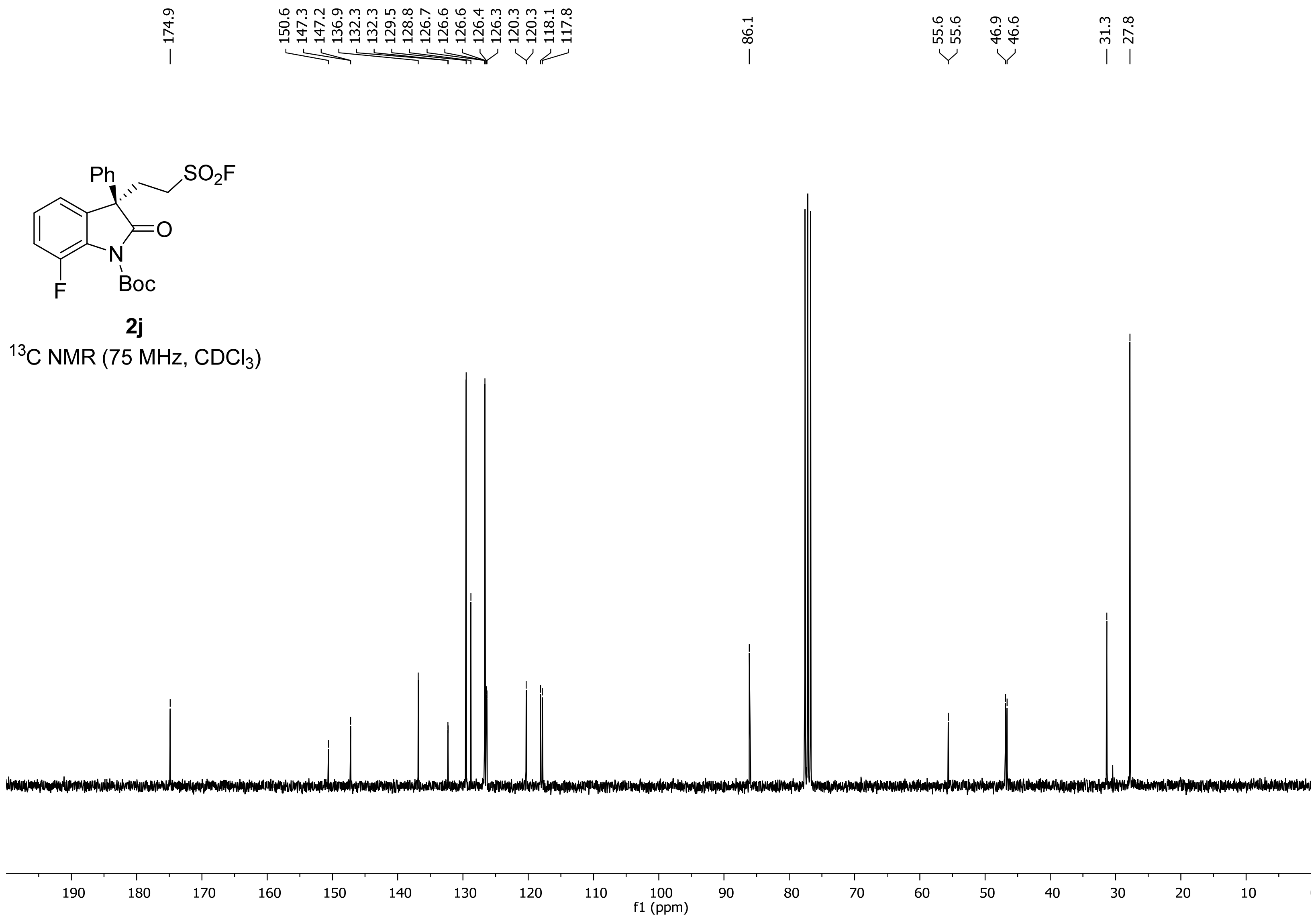
174.9
150.6
147.3
147.2
136.9
132.3
132.3
129.5
128.8
126.7
126.6
126.6
126.4
126.3
120.3
120.3
118.1
117.8
86.1
55.6
55.6
46.9
46.6
31.3
27.8
Ph
SO2F
O
N
Boc
F
2j
13C NMR (75 MHz, CDCl3)
190 180 170 160 150 140 130 120 110 100 90 80 70 60 50 40 30 20 10
f1 (ppm)

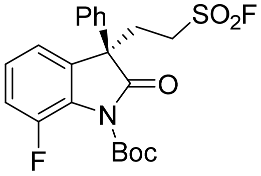

2j

^{19}F NMR (282 MHz, CDCl$_3$)

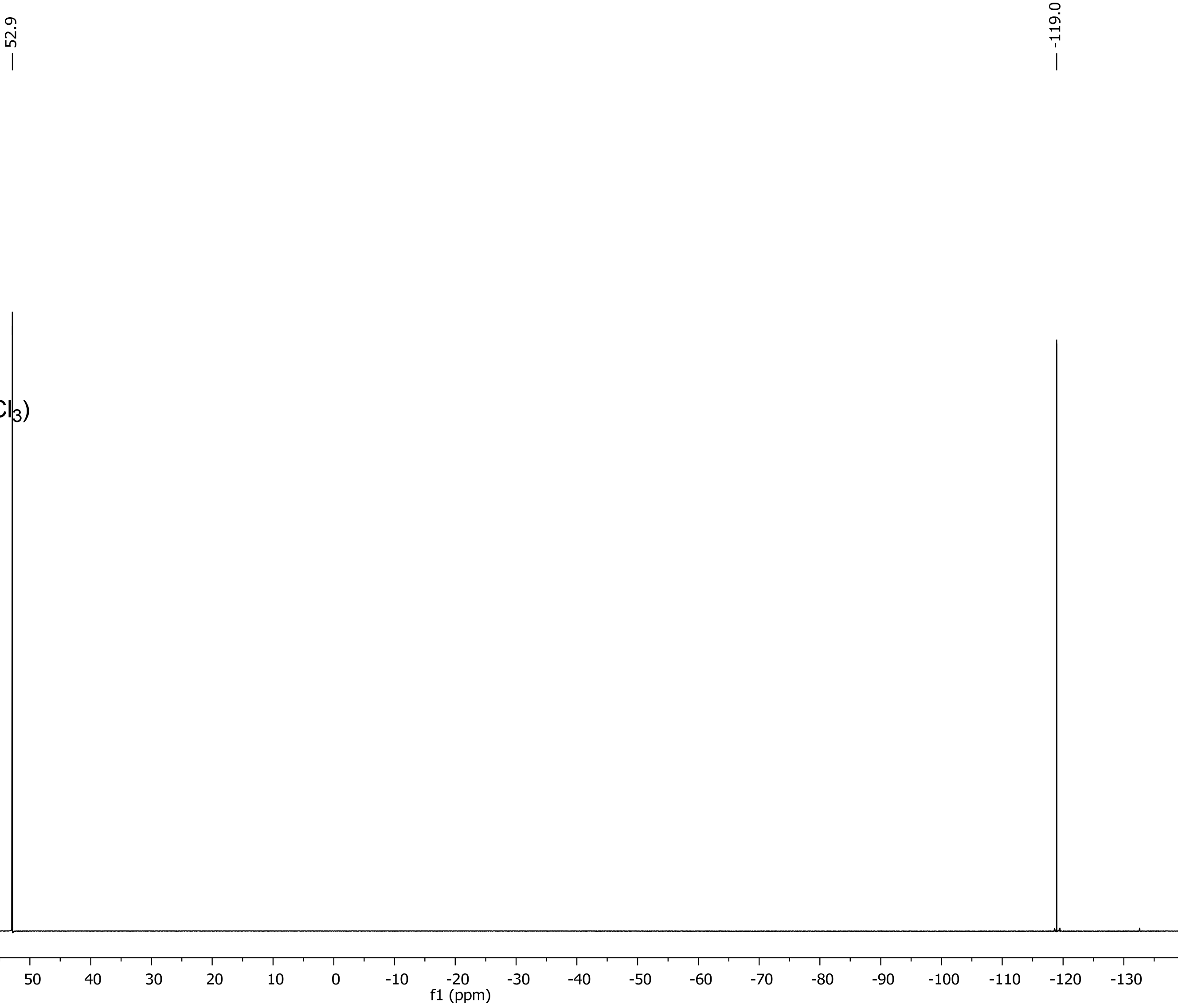

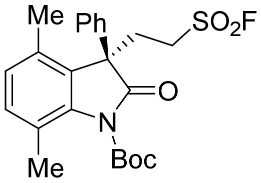

2k

^{1}H NMR (300 MHz, $CDCl_3$)

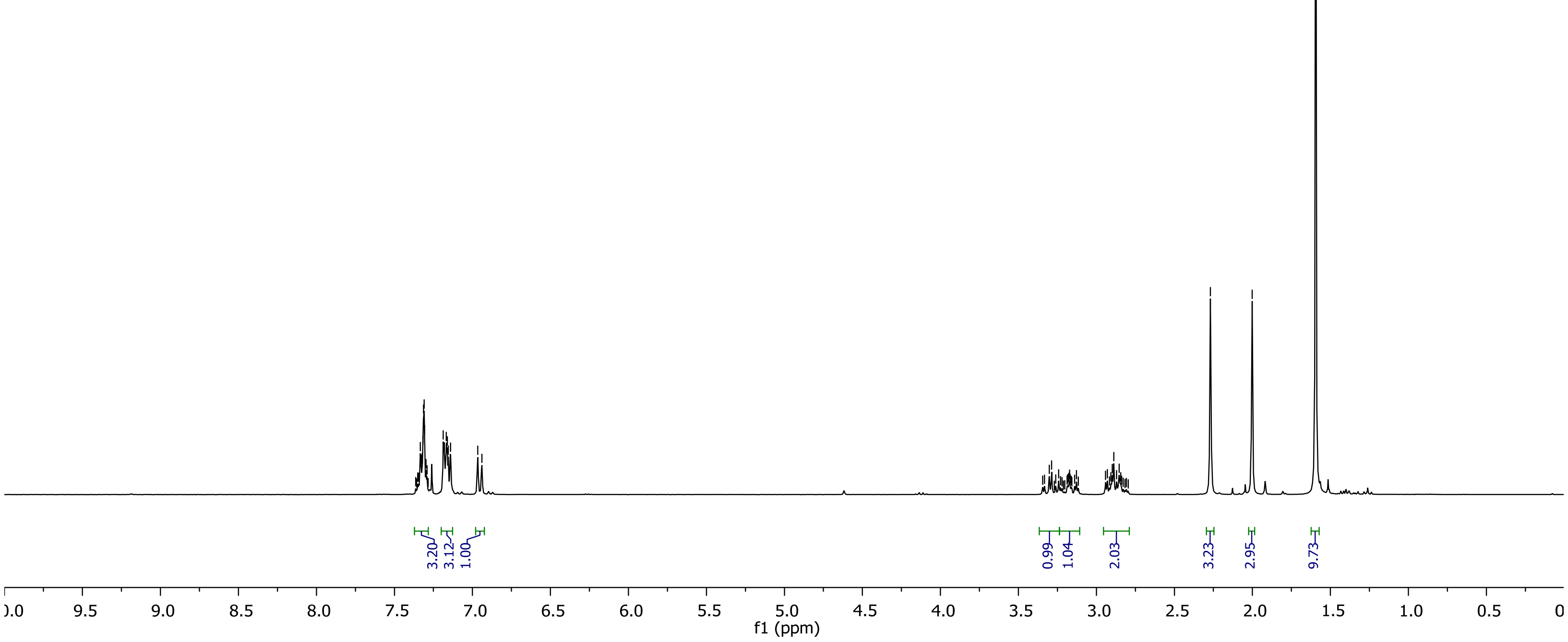

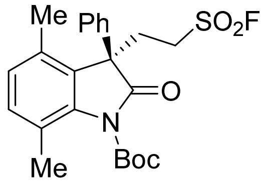

2k

^{13}C NMR (75 MHz, $CDCl_3$)

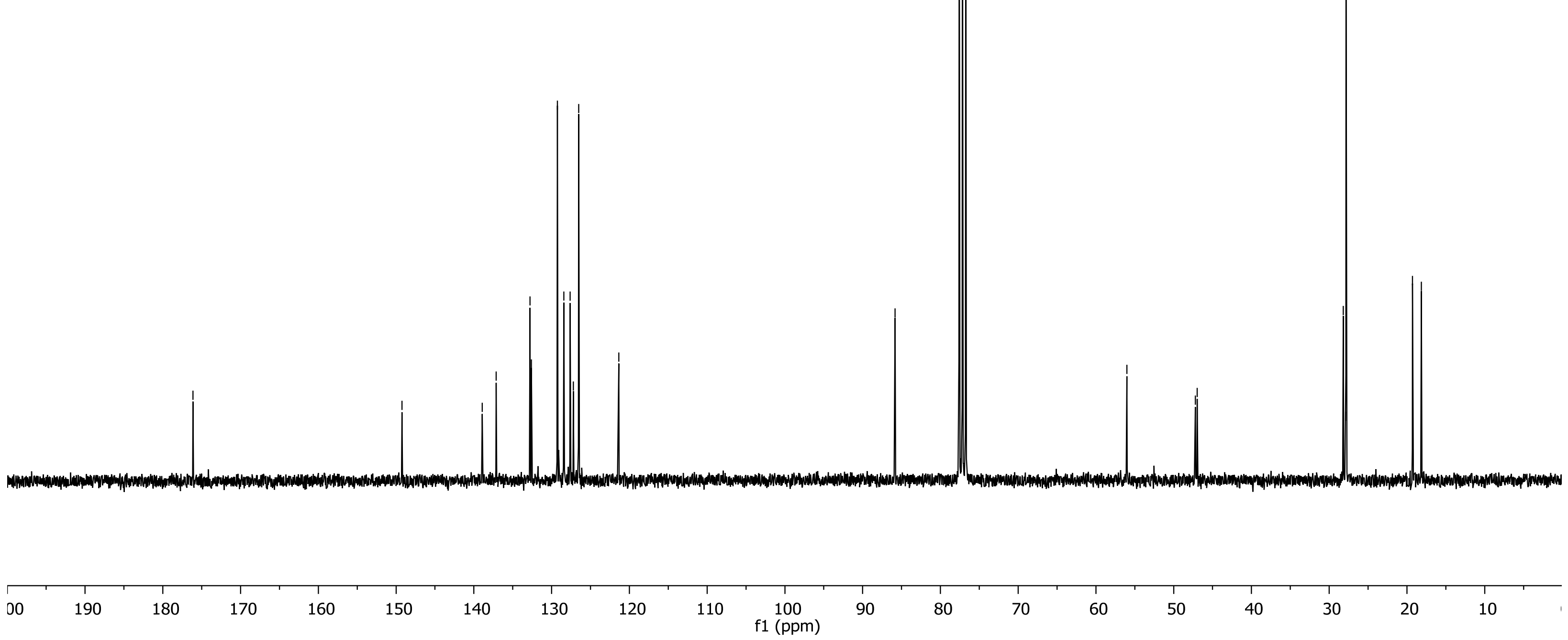

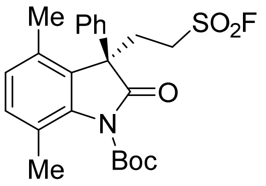

2k

^{19}F NMR (282 MHz, $CDCl_3$)

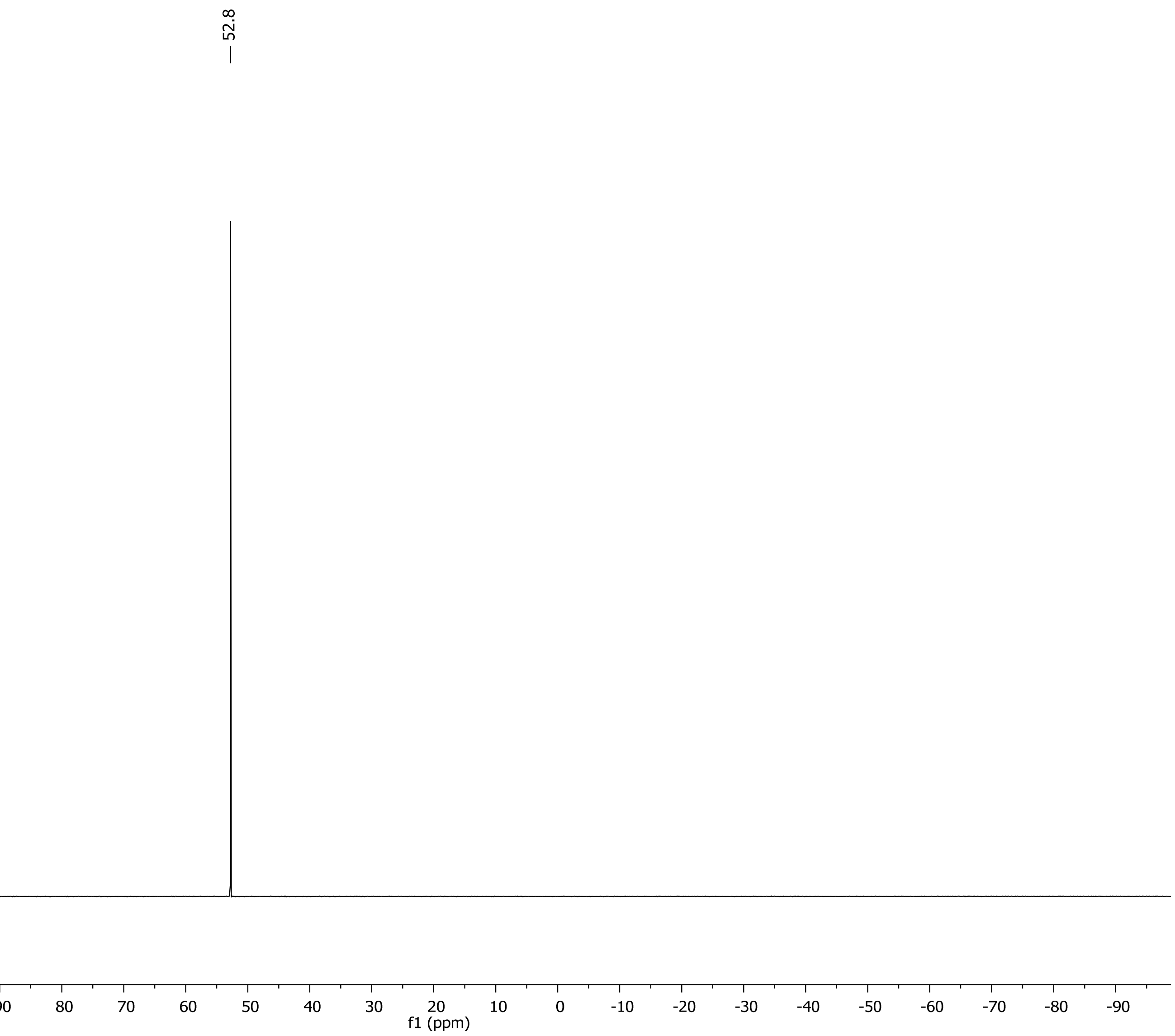

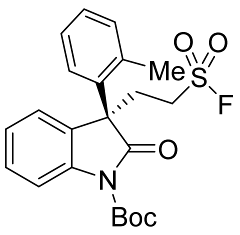

2l

^{1}H NMR (300 MHz, $CDCl_3$)

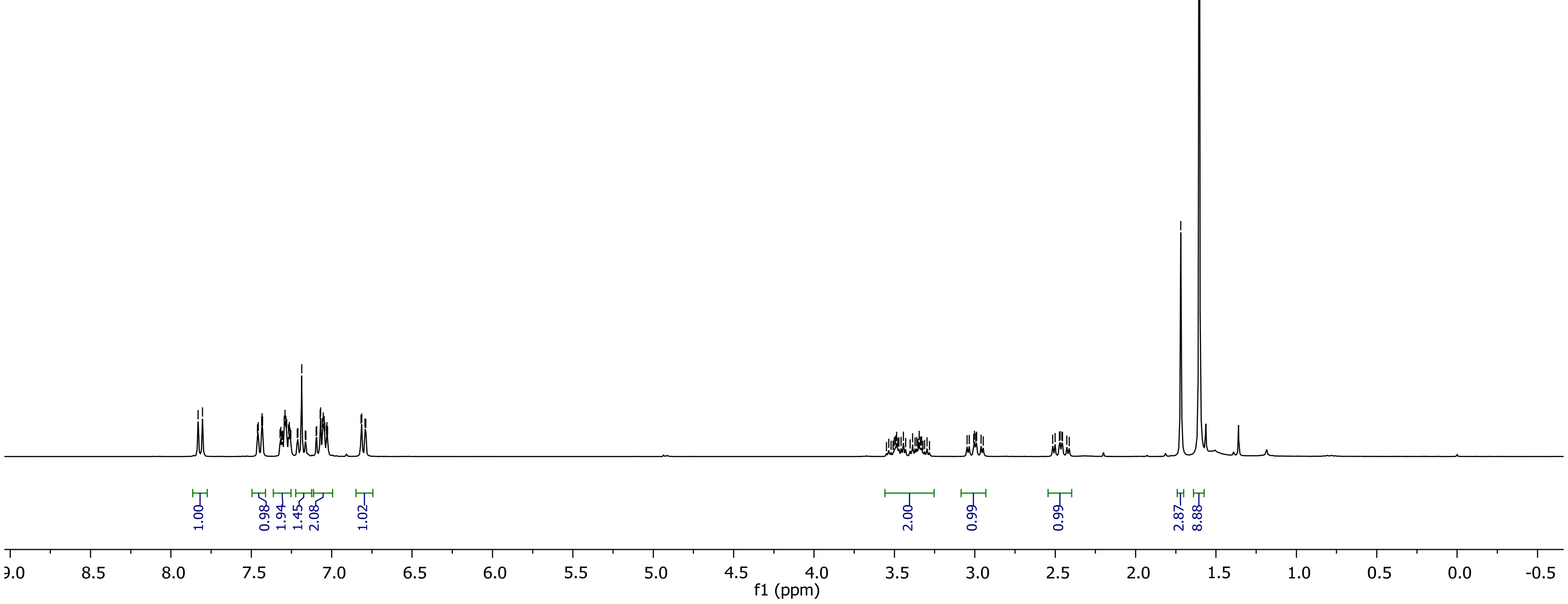

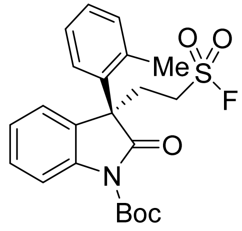

2l

^{13}C NMR (75 MHz, $CDCl_3$)

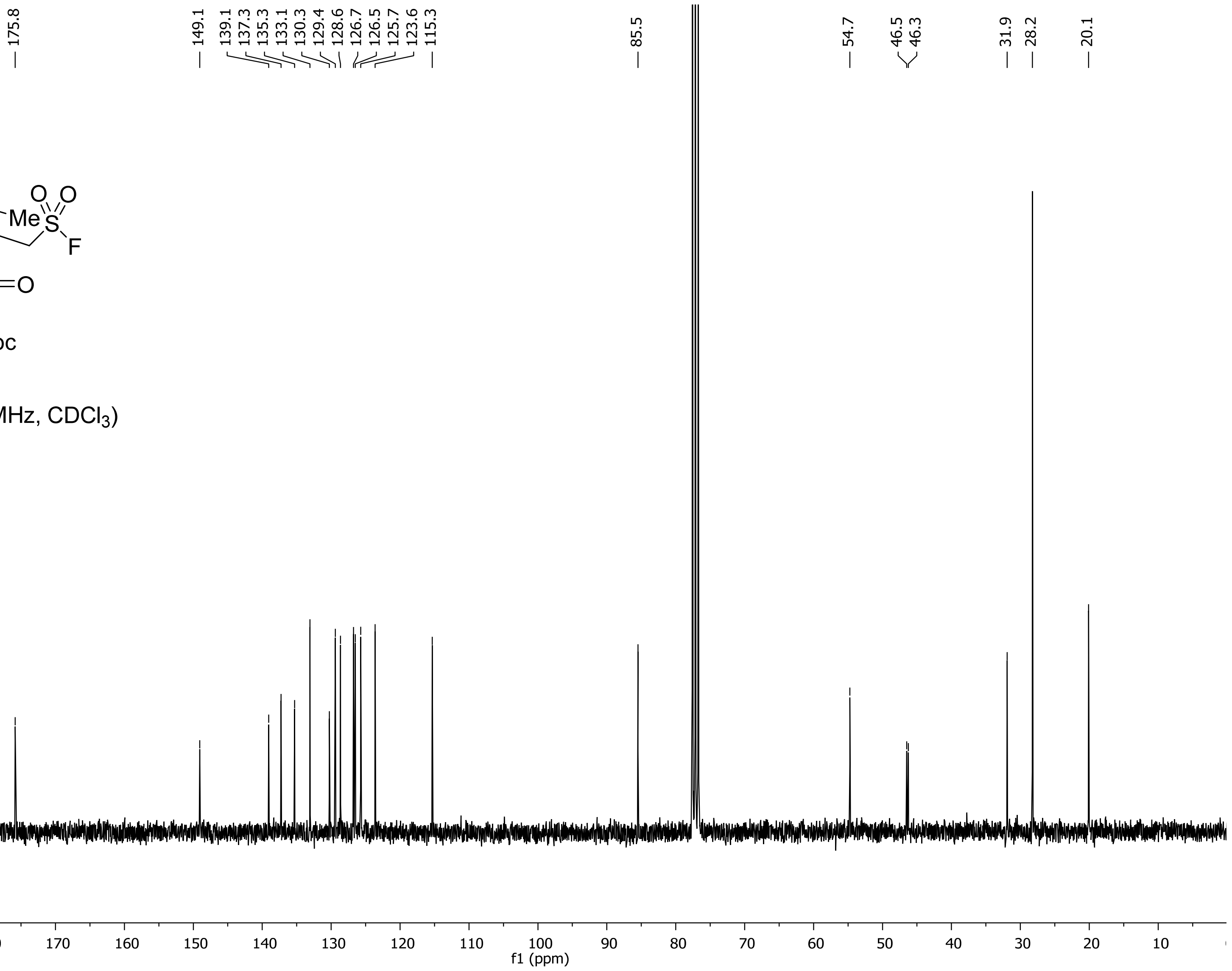

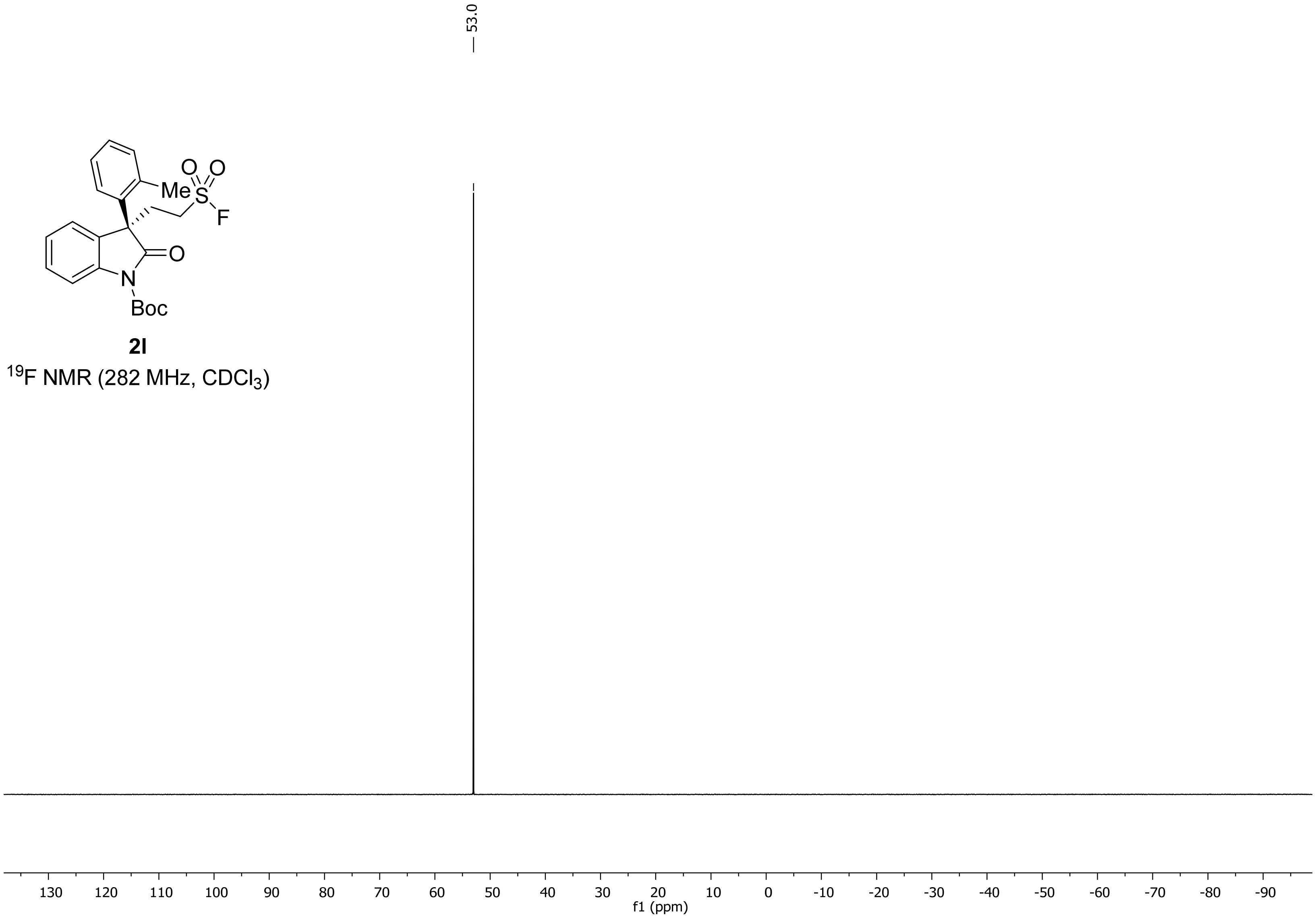

2l

^{19}F NMR (282 MHz, $CDCl_3$)

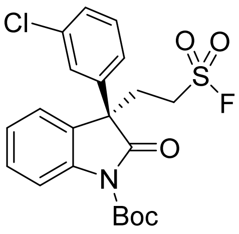

2m

^{1}H NMR (300 MHz, $CDCl_3$)

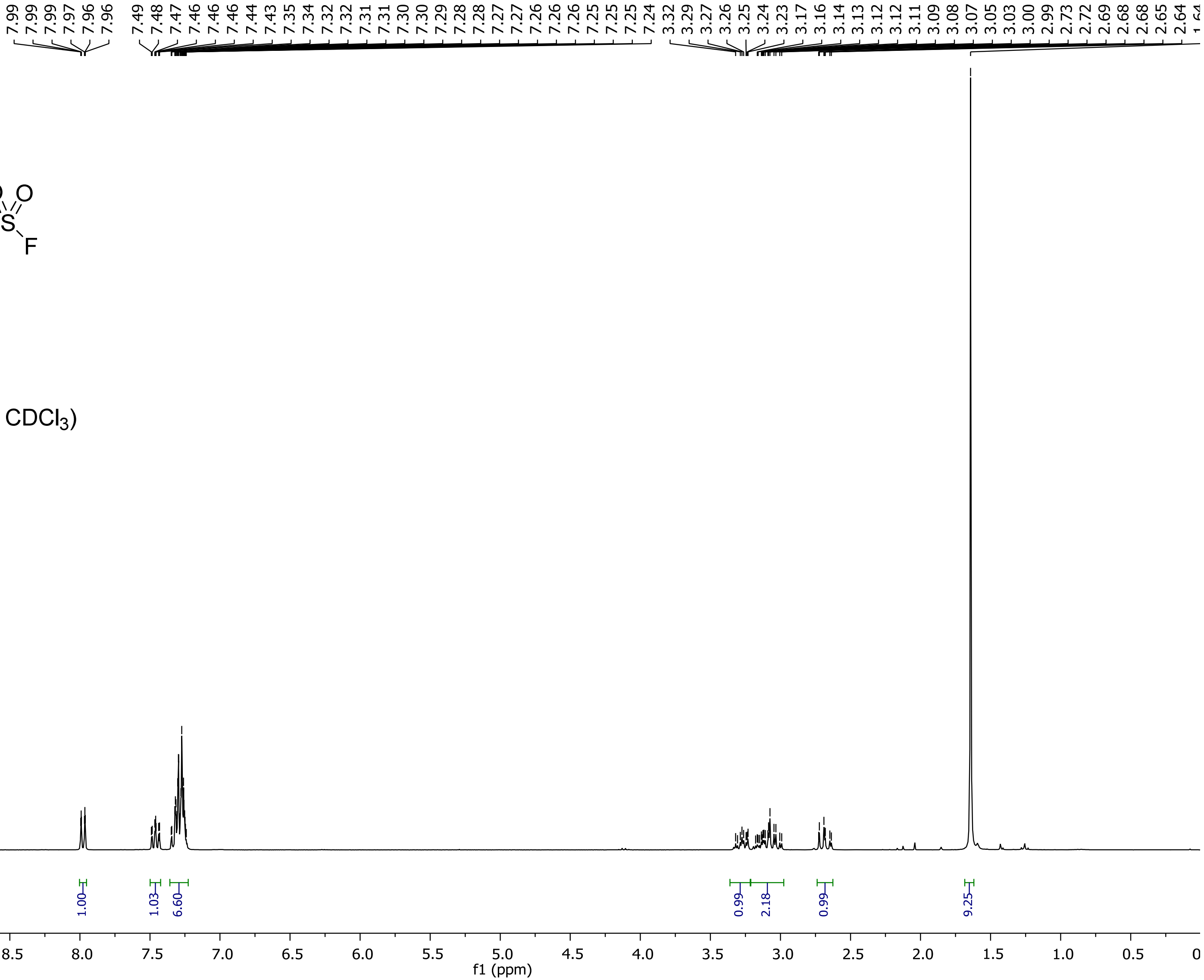

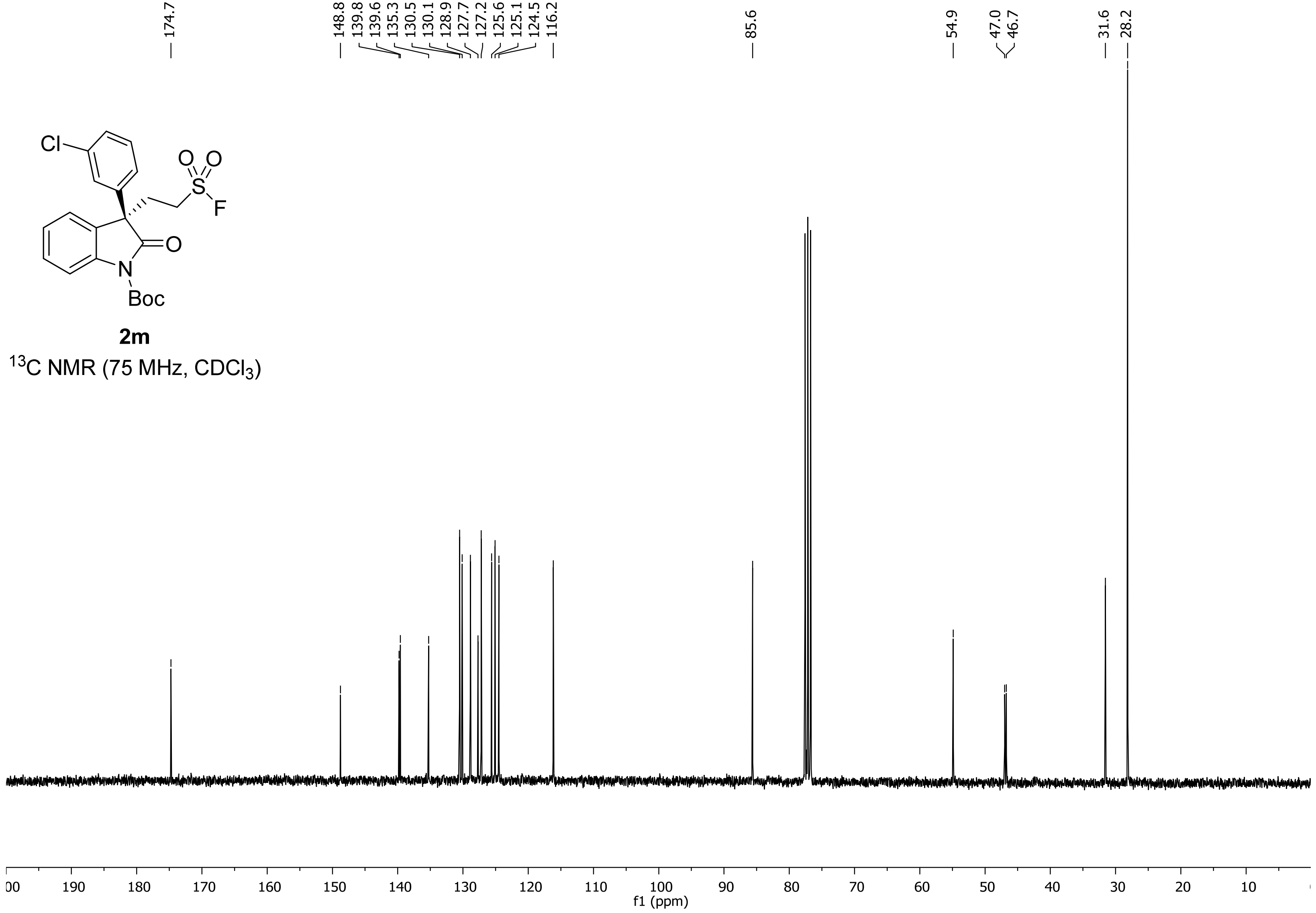
174.7
148.8
139.8
139.6
135.3
130.5
130.1
128.9
127.7
127.2
125.6
125.1
124.5
116.2
85.6
54.9
47.0
46.7
31.6
28.2
Cl
O
O
S
F
O
N
Boc
2m
^{13}C NMR (75 MHz, $CDCl_3$)
190
180
170
160
150
140
130
120
110
100
90
80
70
60
50
40
30
20
10
f1 (ppm)

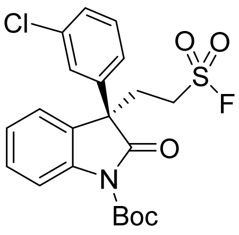

2m

^{19}F NMR (282 MHz, $CDCl_3$)

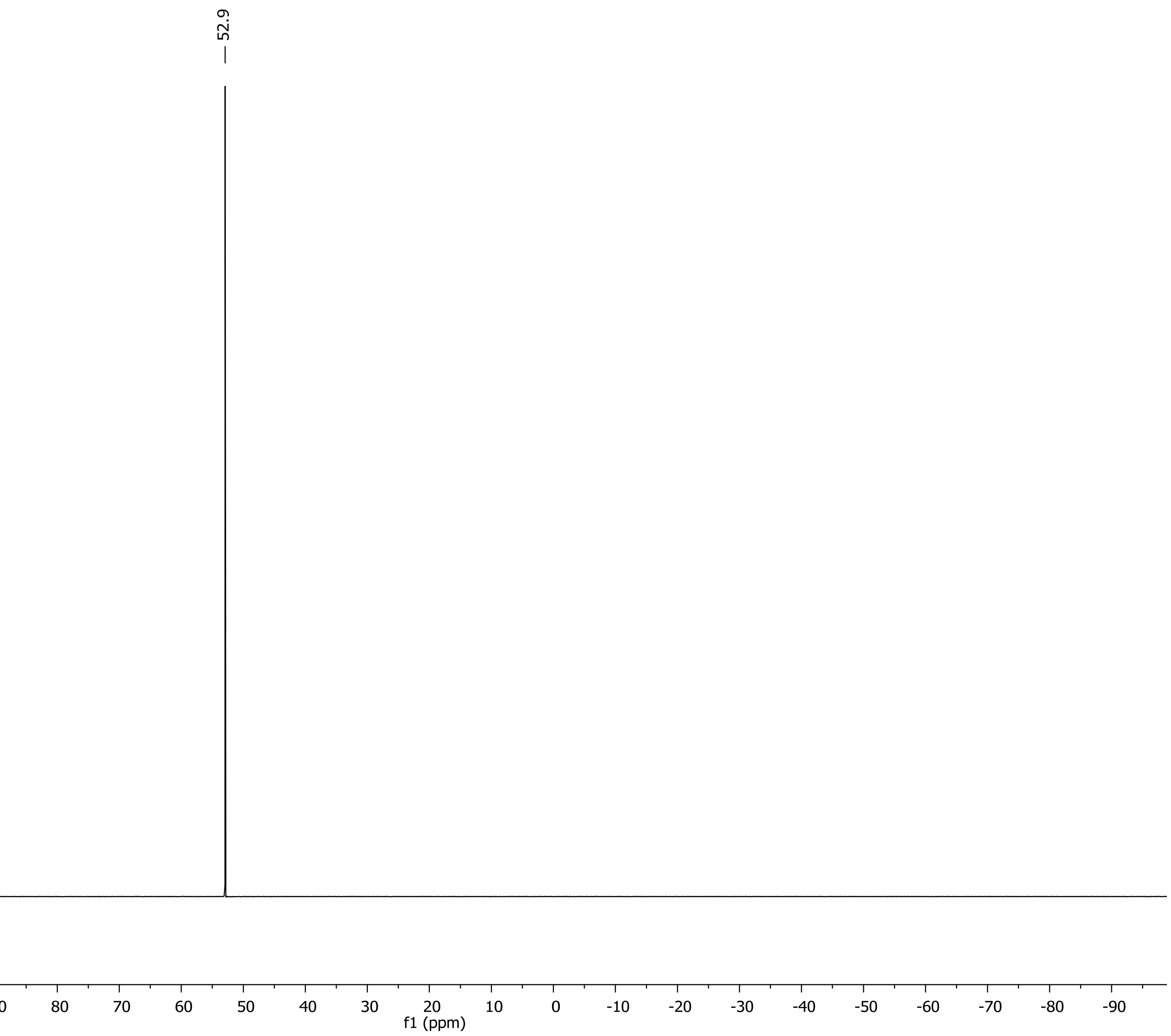

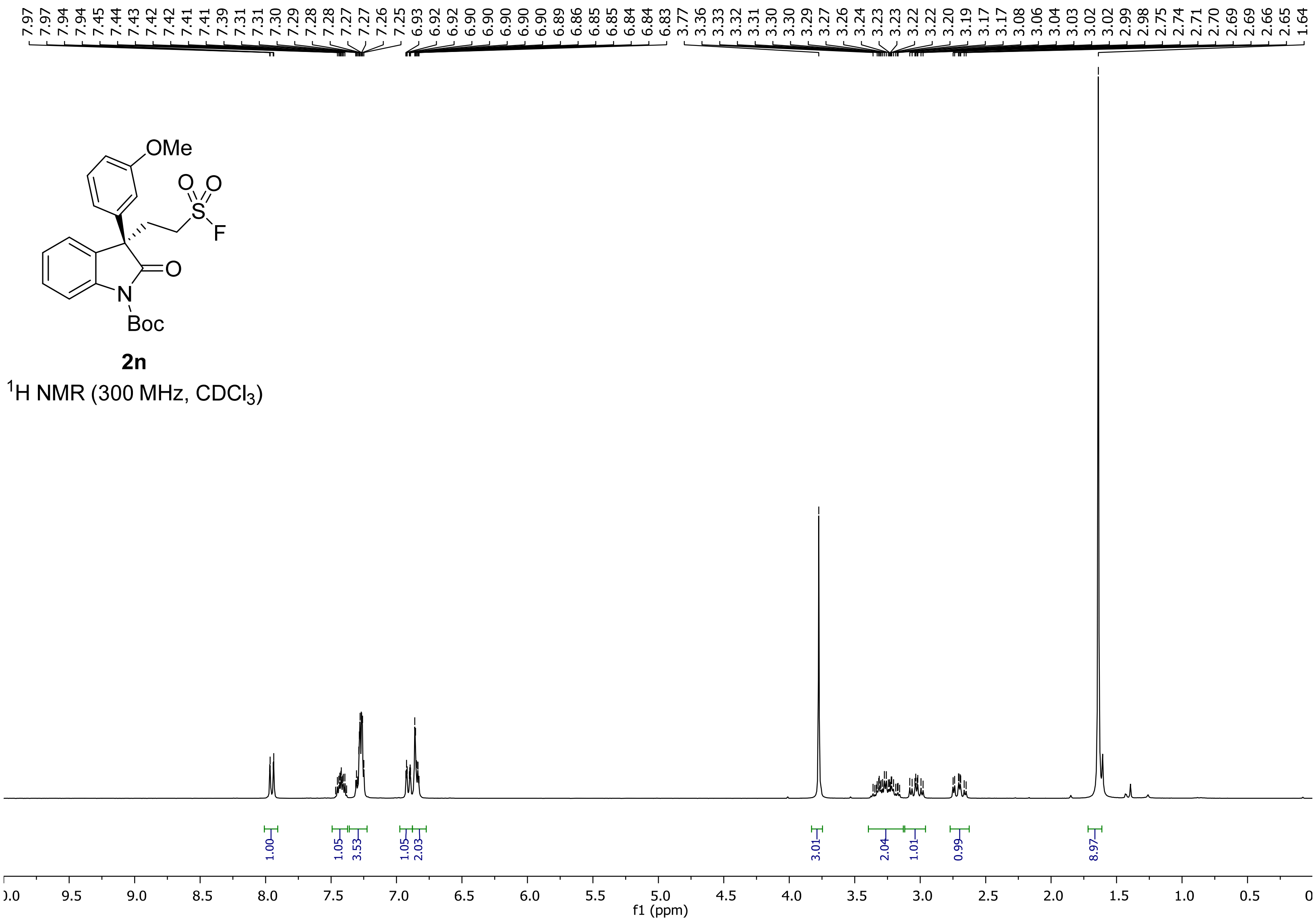

2n

^{1}H NMR (300 MHz, $CDCl_3$)

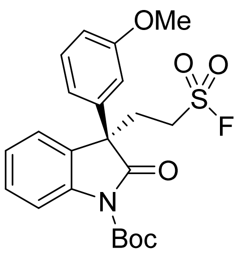

2n

^{13}C NMR (75 MHz, $CDCl_3$)

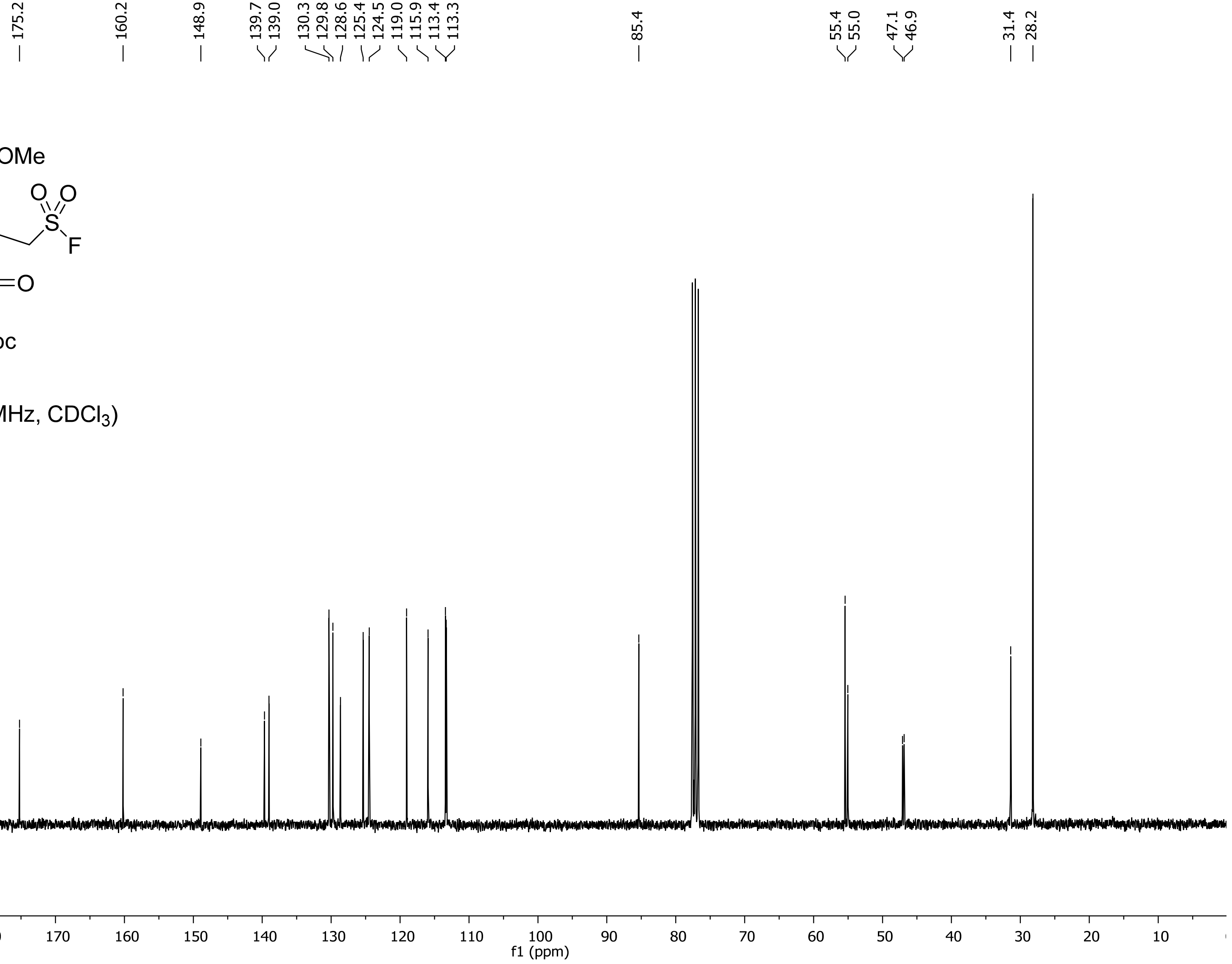

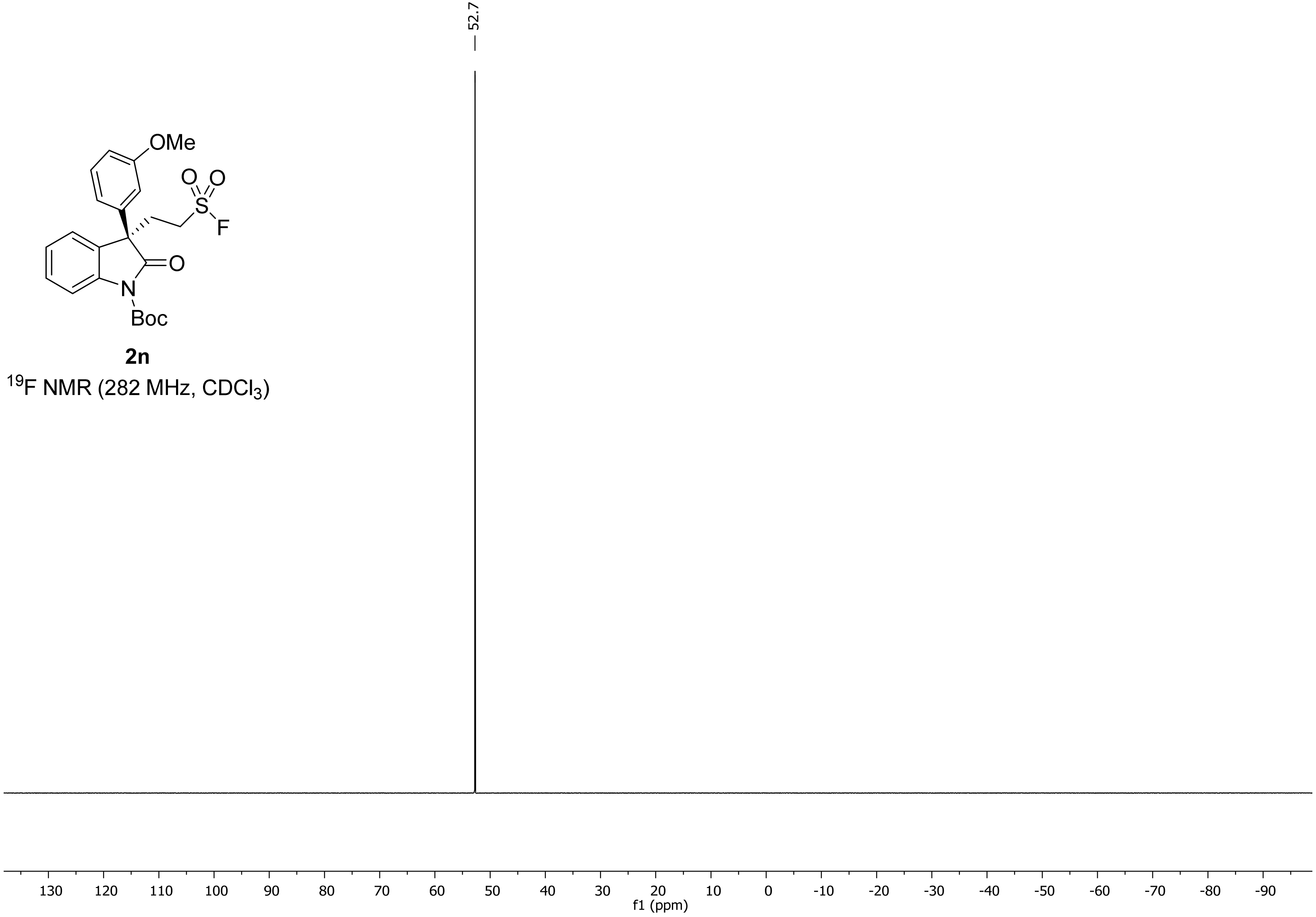

2n

^{19}F NMR (282 MHz, $CDCl_3$)

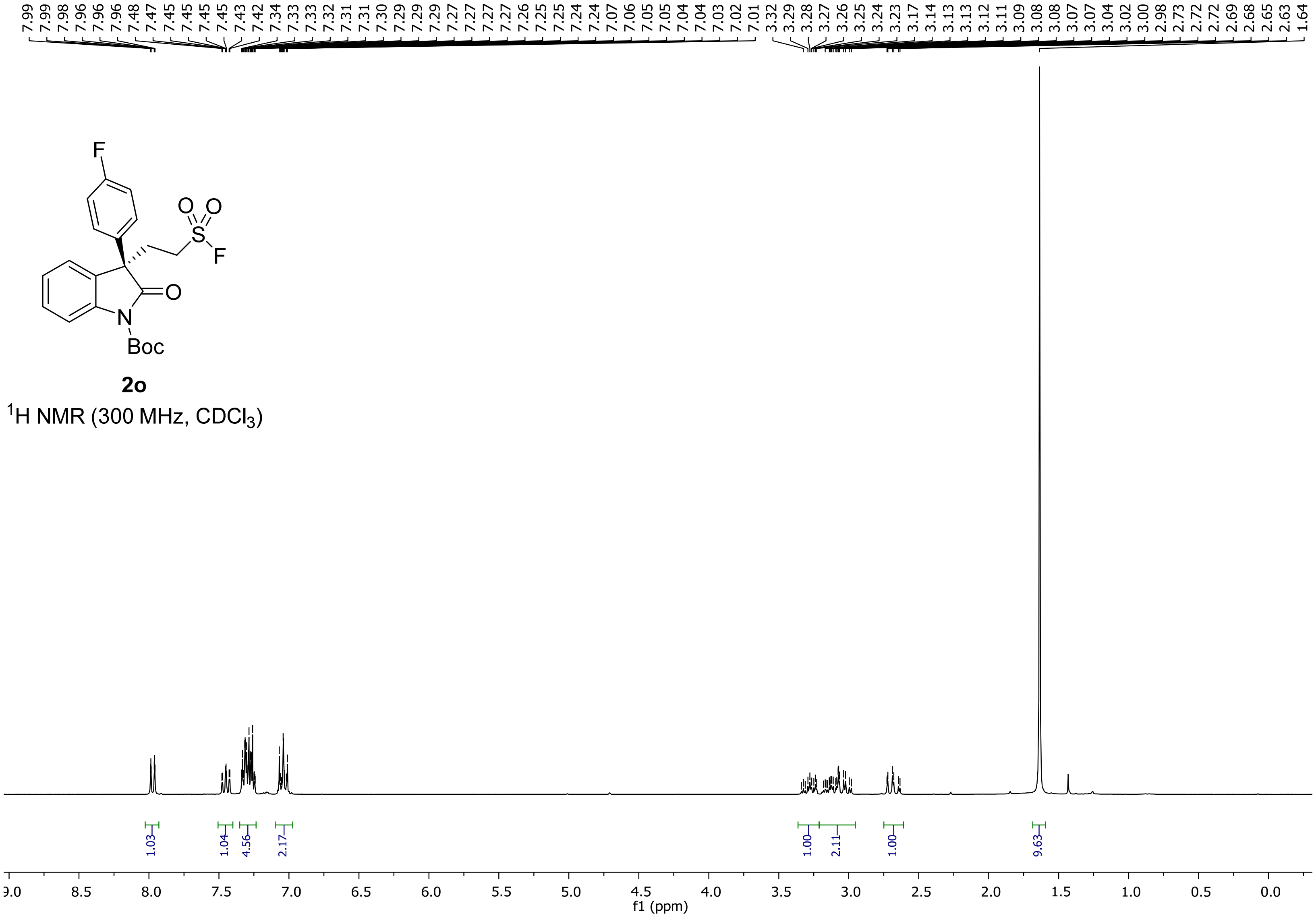

2o

^{1}H NMR (300 MHz, $CDCl_3$)

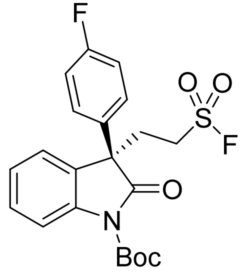

2o

^{13}C NMR (75 MHz, $CDCl_3$)

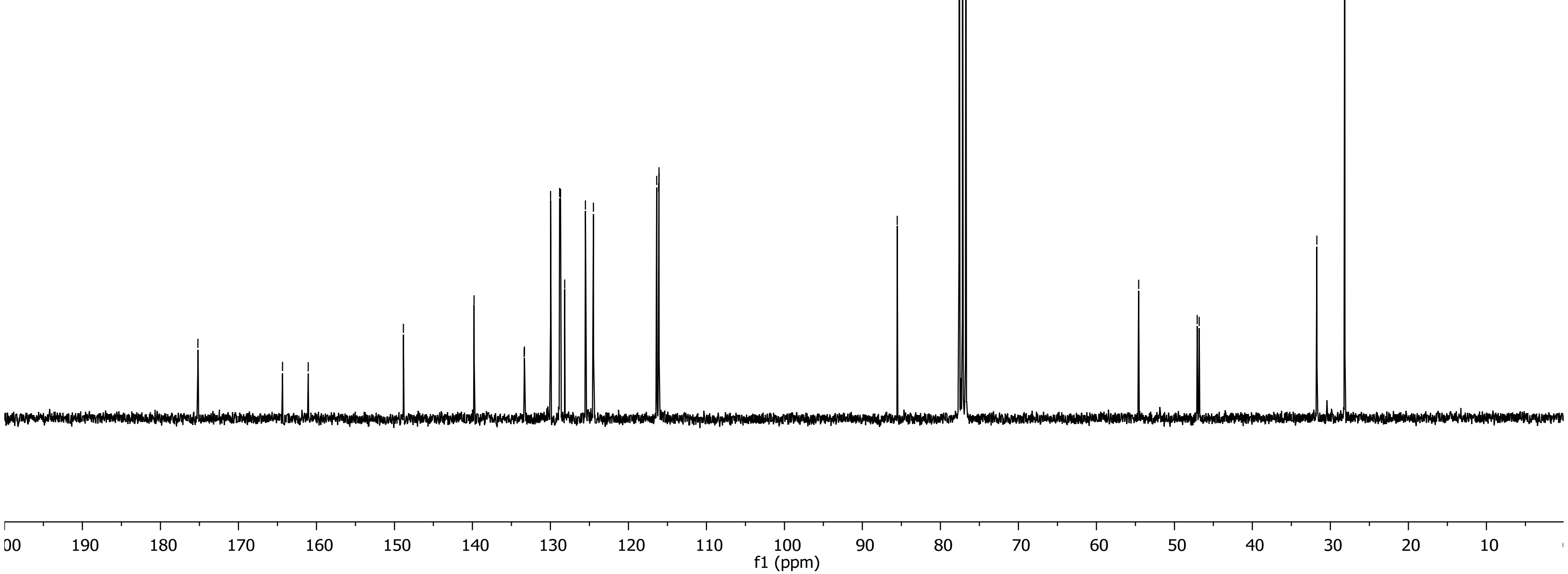

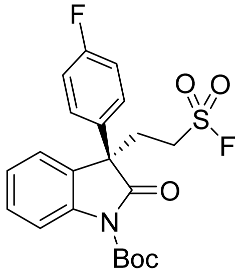

2o

^{19}F NMR (282 MHz, $CDCl_3$)

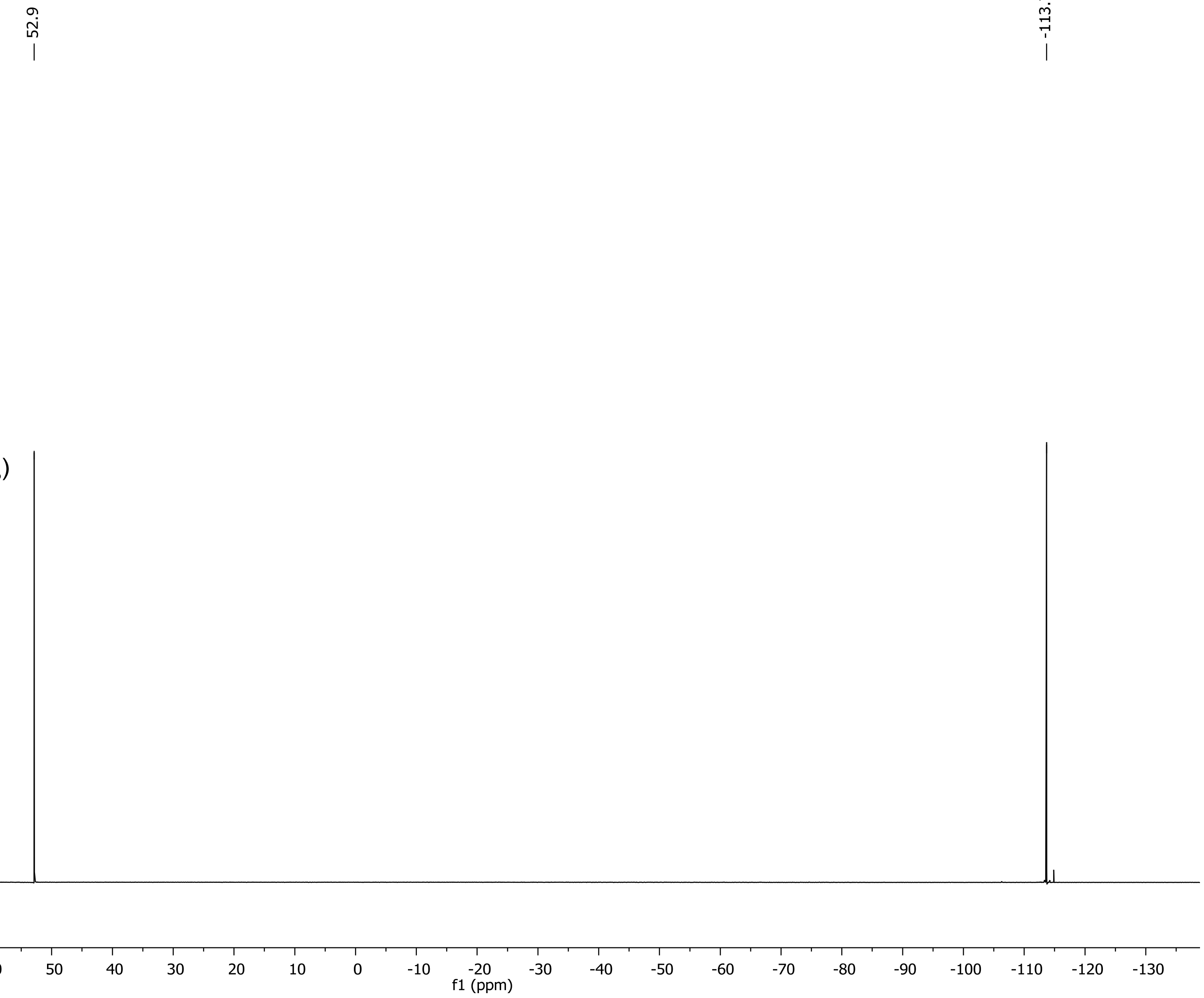

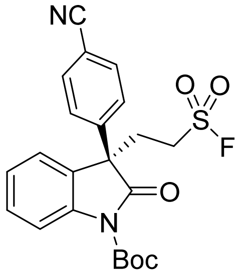

2p

^{1}H NMR (300 MHz, $CDCl_3$)

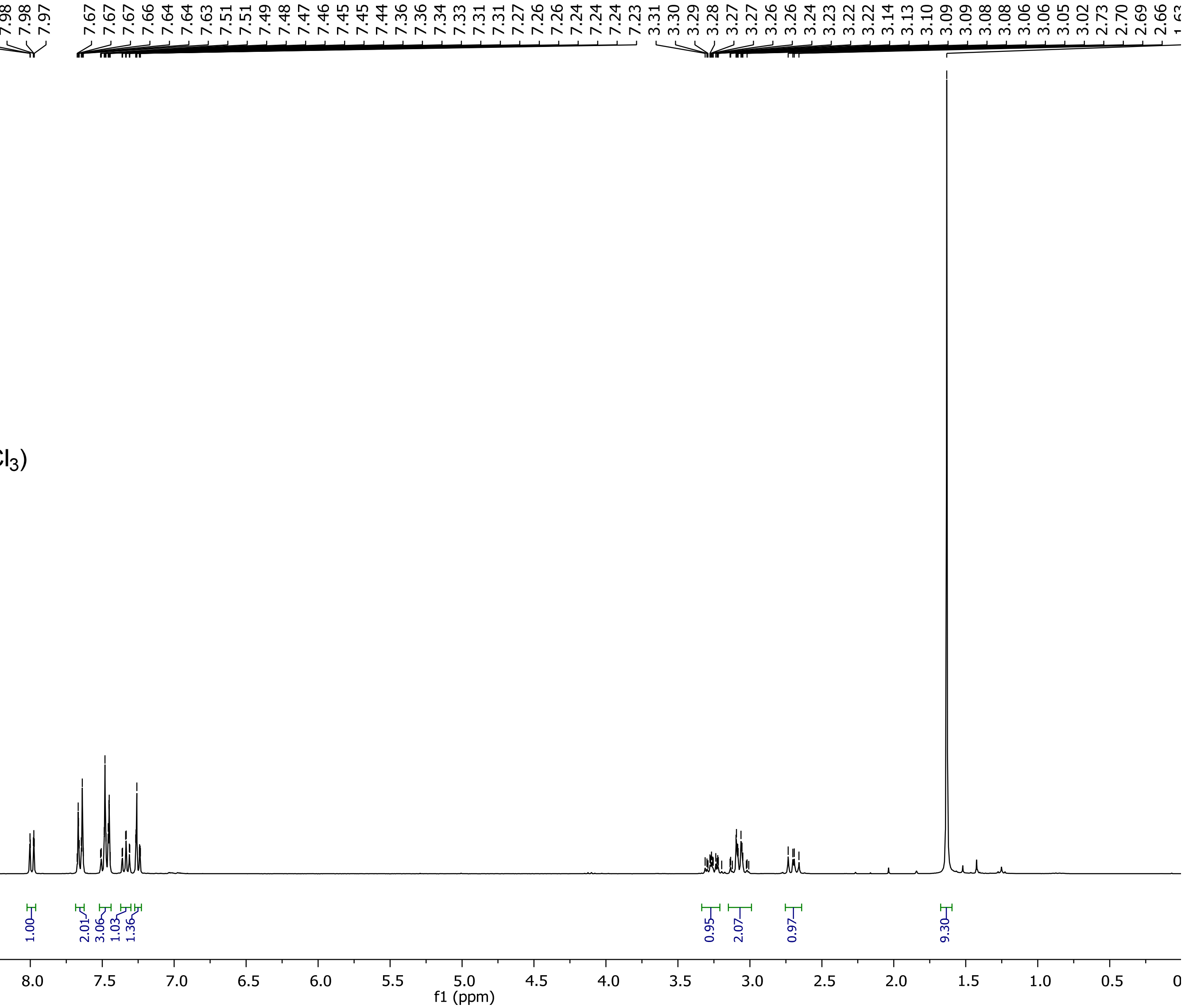

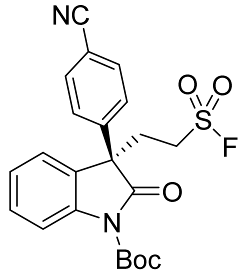

2p

^{13}C NMR (75 MHz, $CDCl_3$)

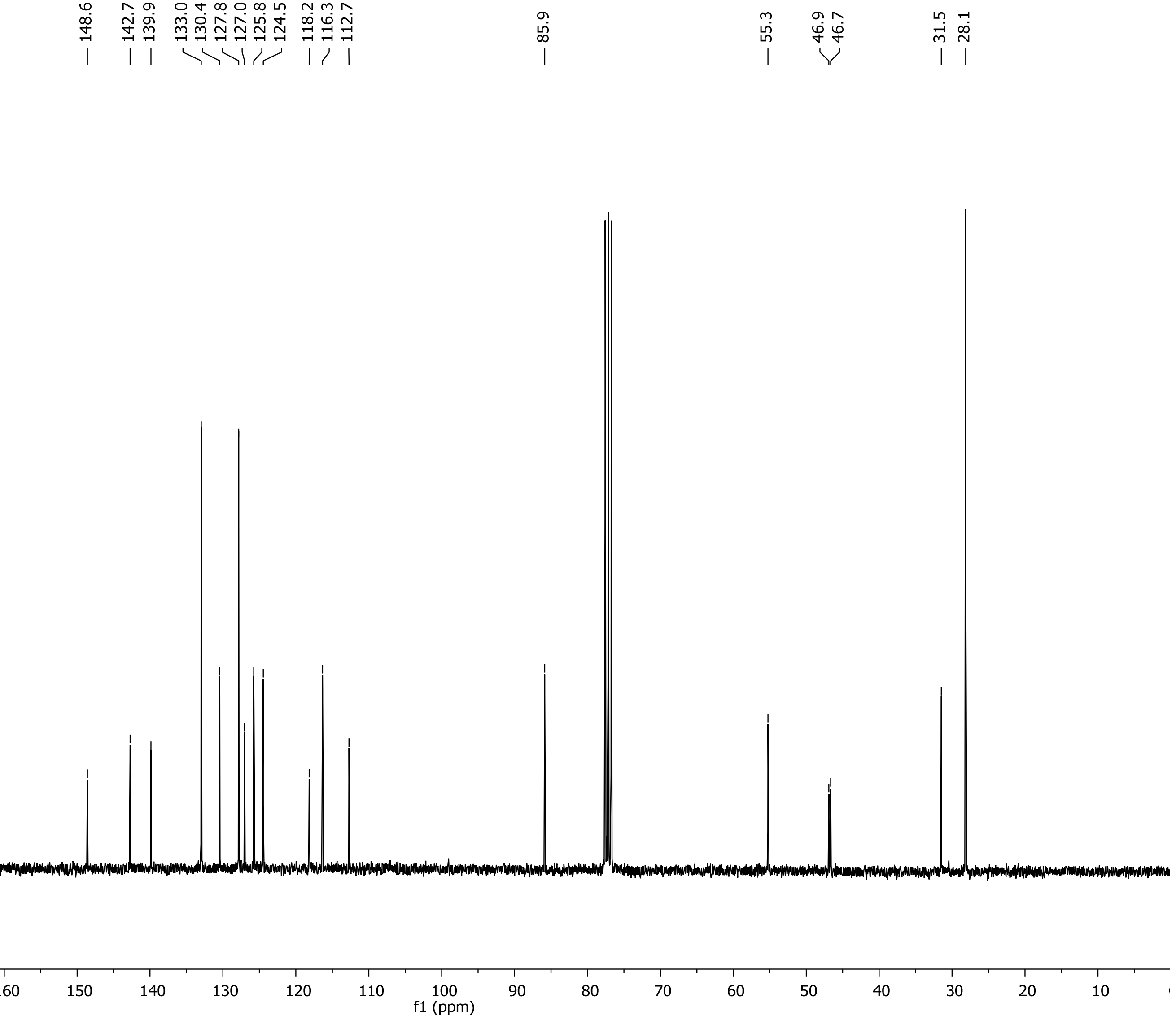

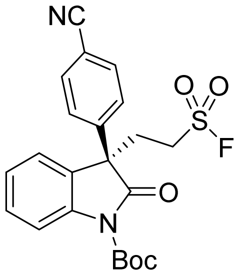

2p

^{19}F NMR (282 MHz, $CDCl_3$)

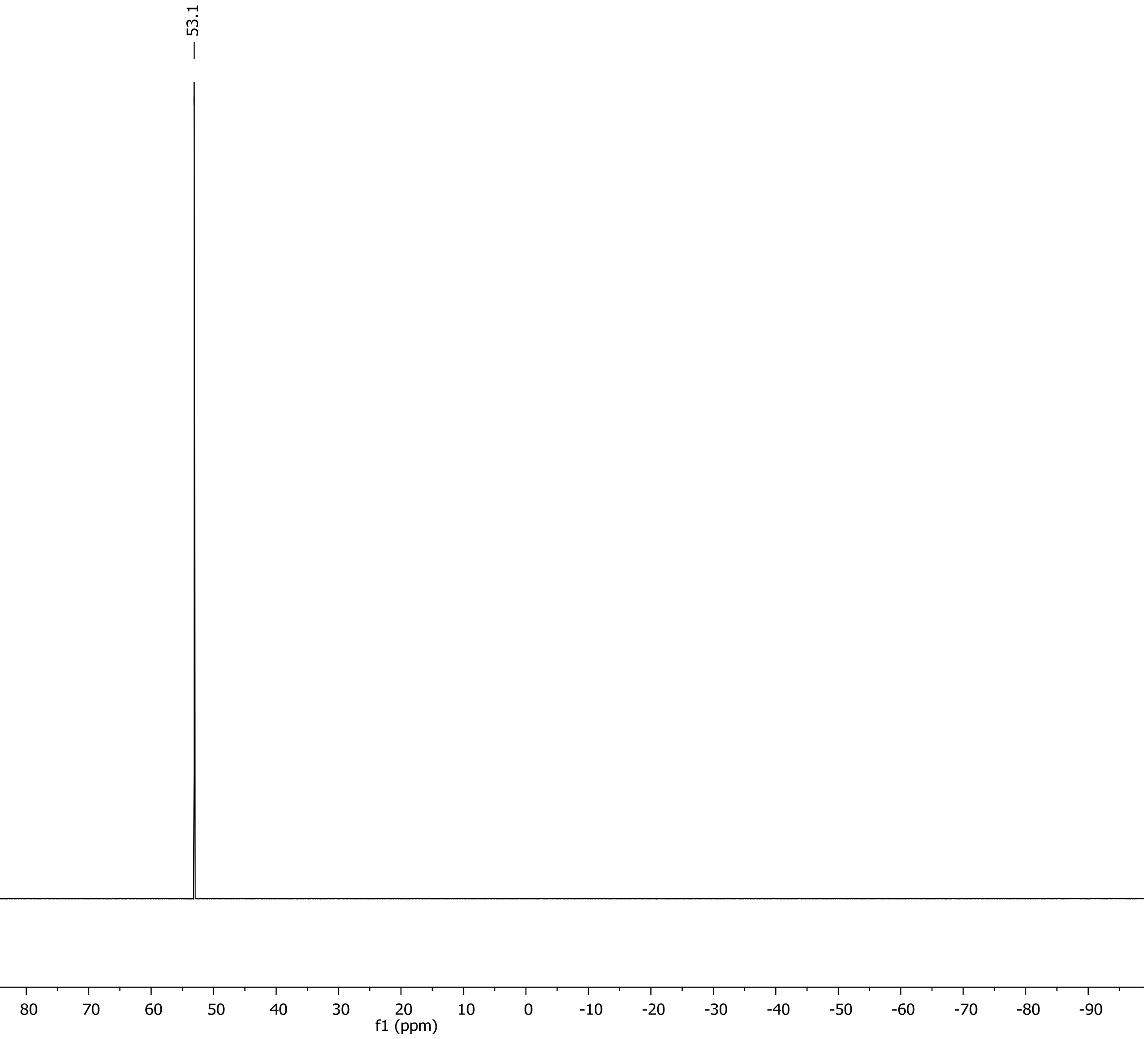

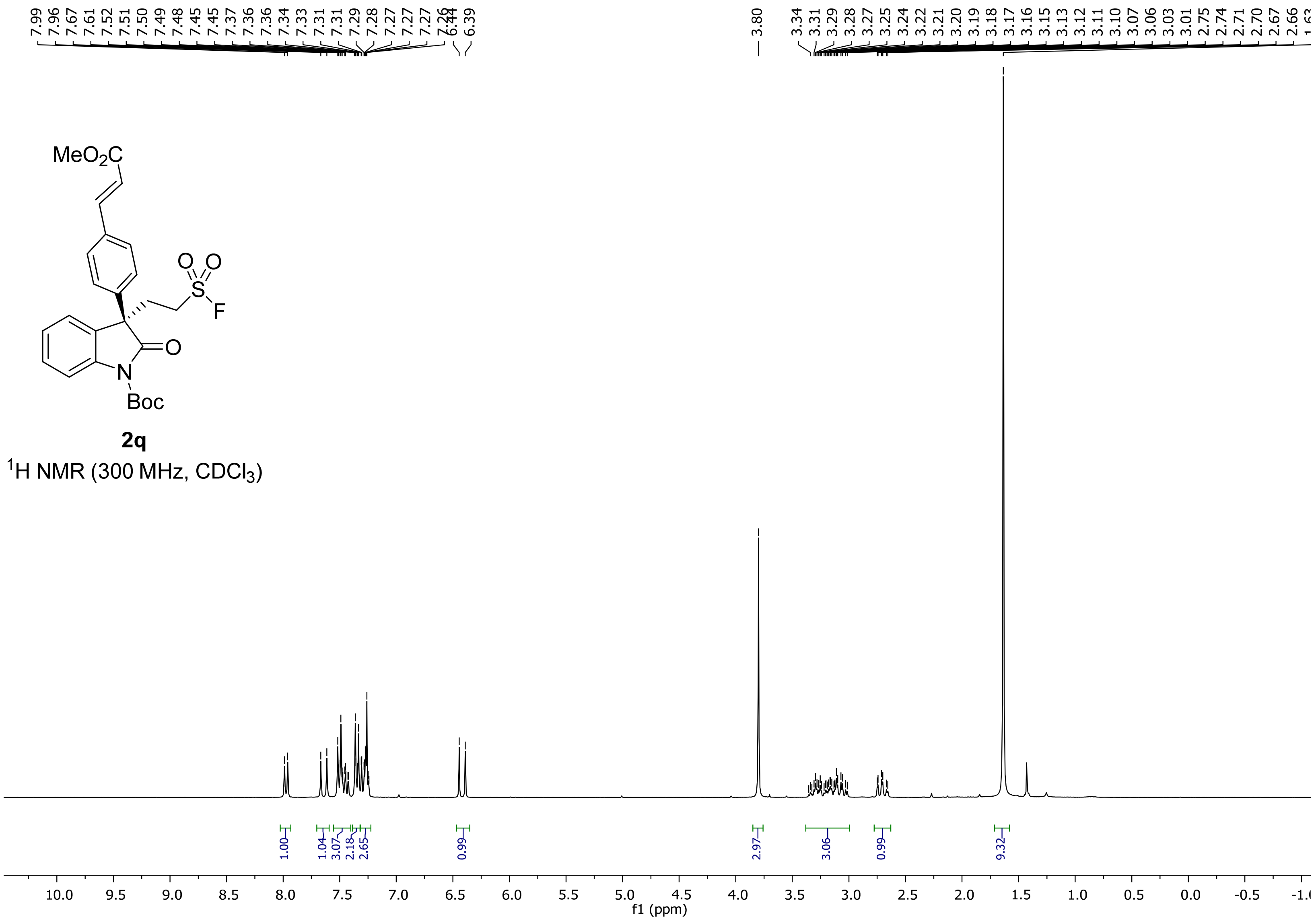

2q

^1H NMR (300 MHz, $CDCl_3$)

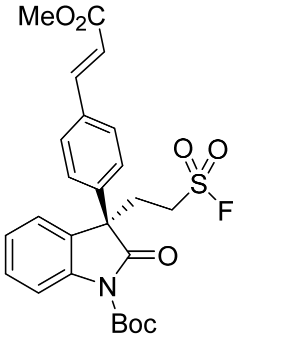

2q

^{13}C NMR (75 MHz, $CDCl_3$)

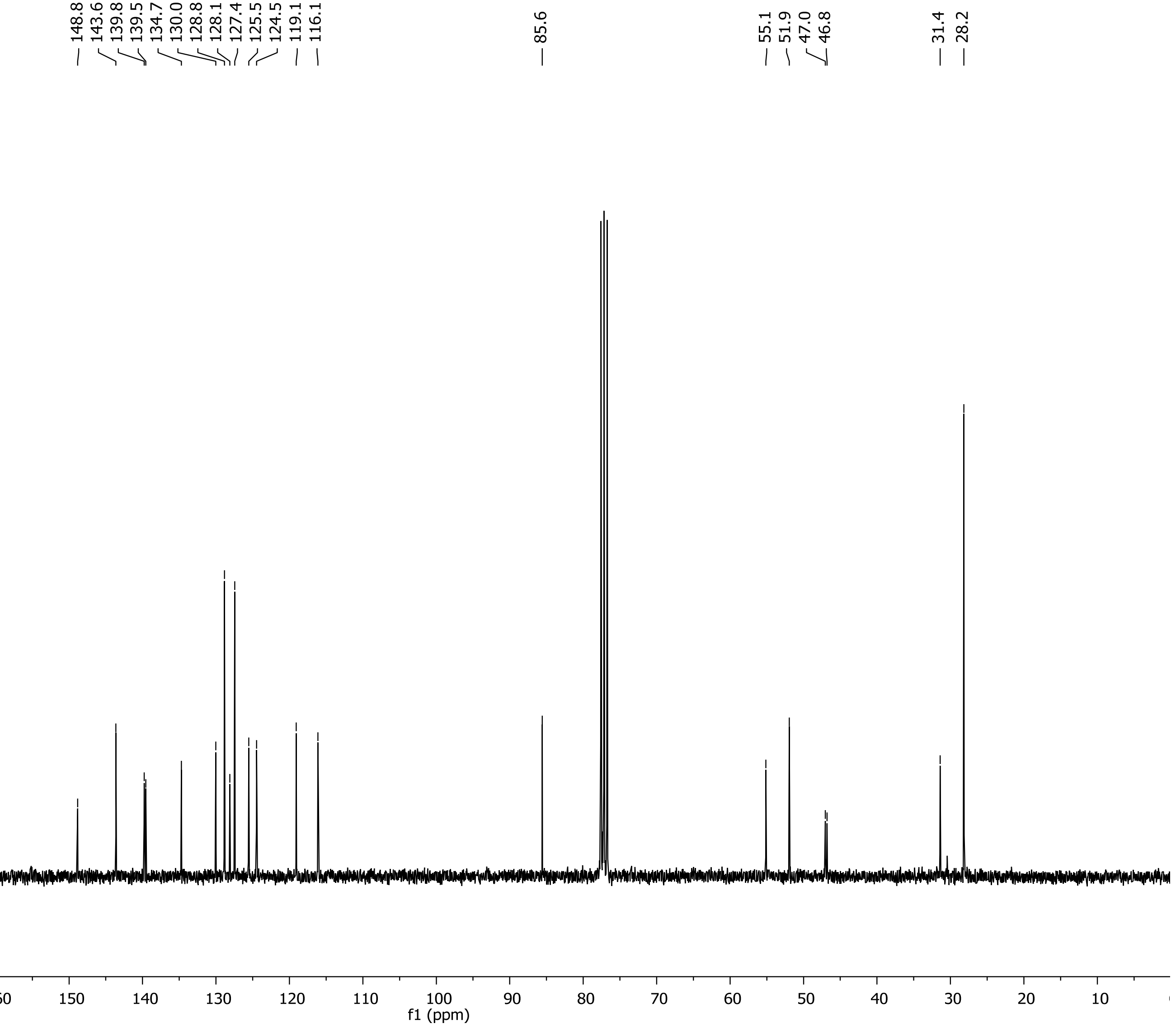

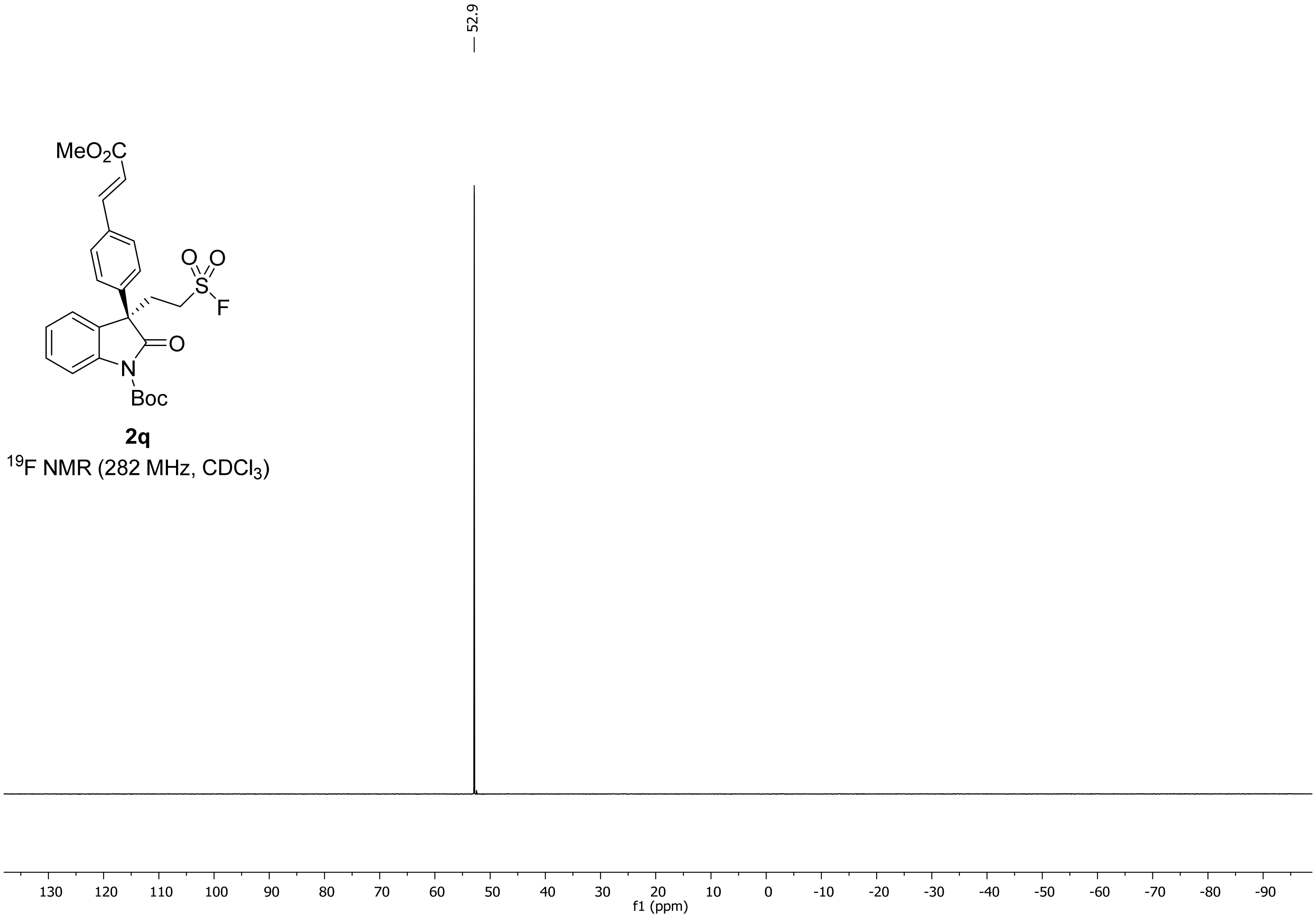

2q

^{19}F NMR (282 MHz, $CDCl_3$)

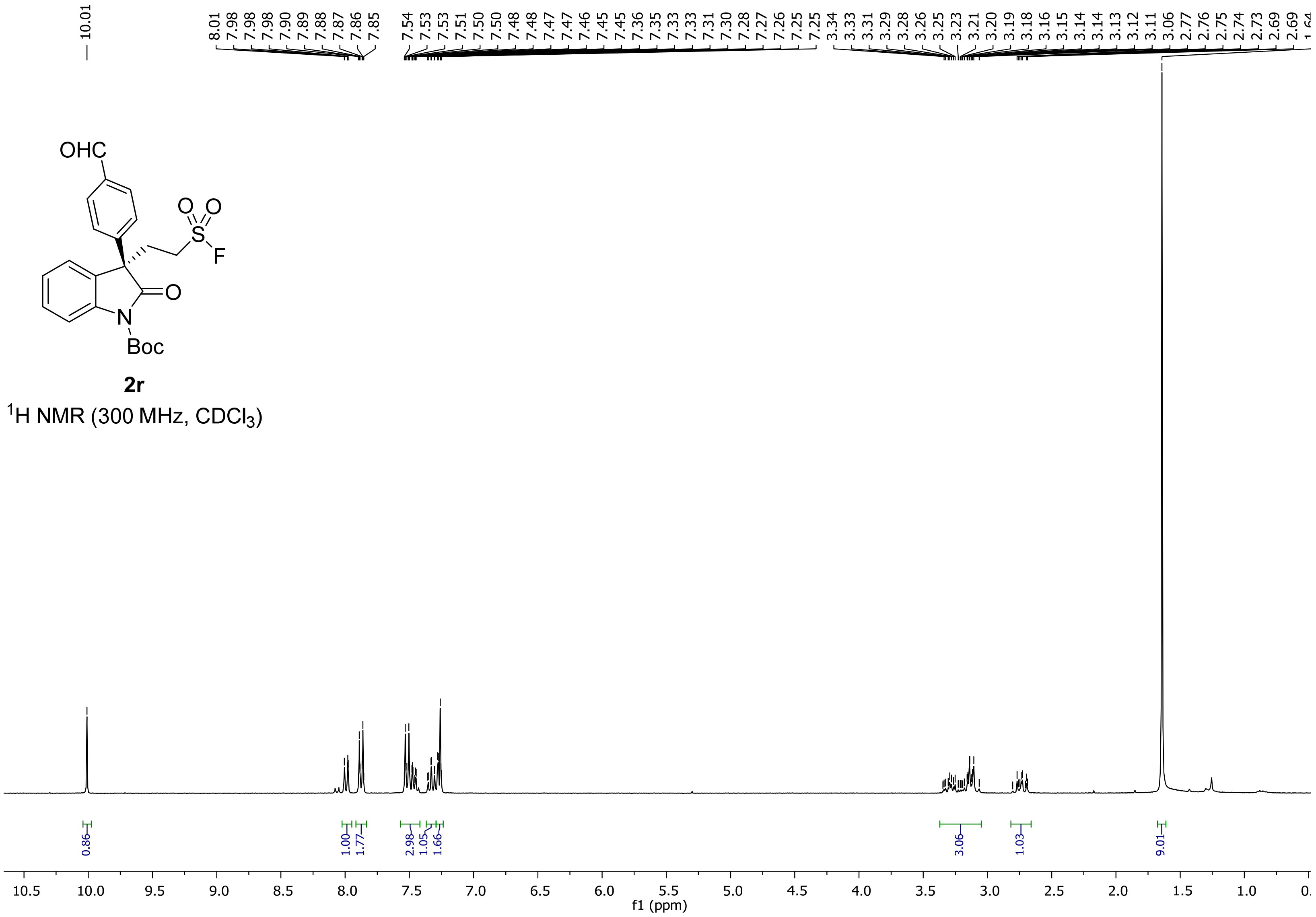

2r

^{1}H NMR (300 MHz, $CDCl_3$)

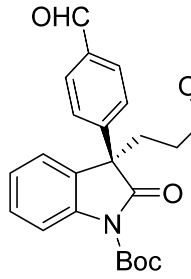

2r

^{13}C NMR (75 MHz, $CDCl_3$)

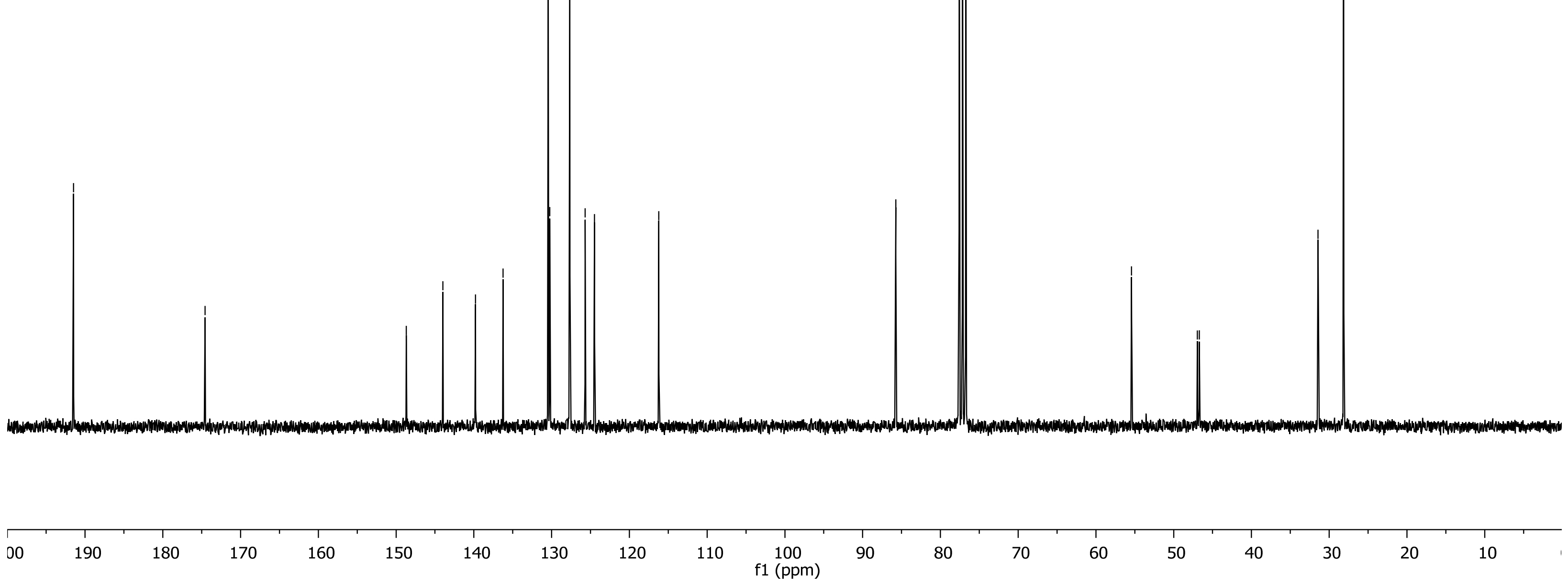

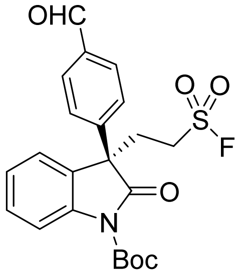

2r

^{19}F NMR (282 MHz, $CDCl_3$)

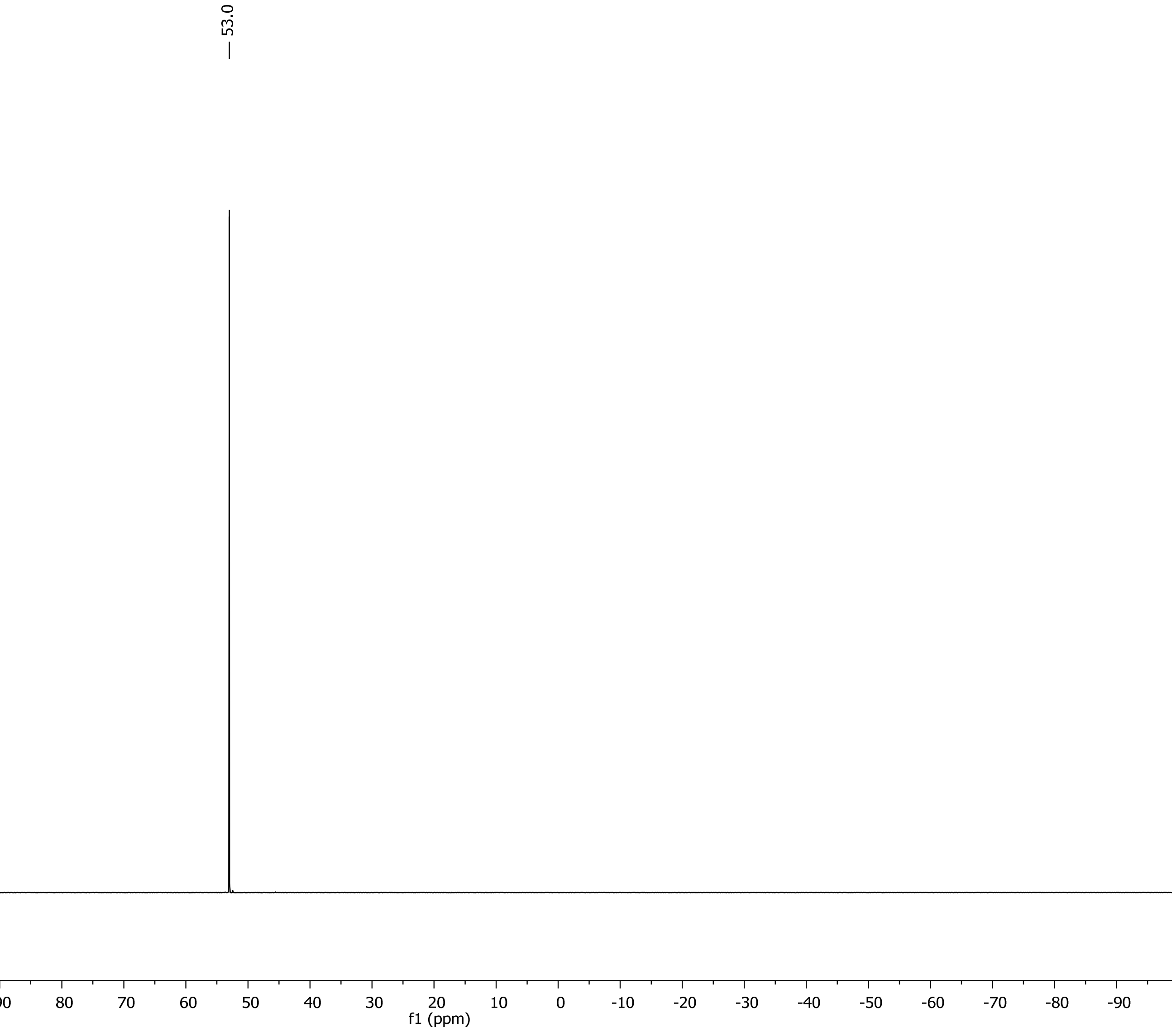

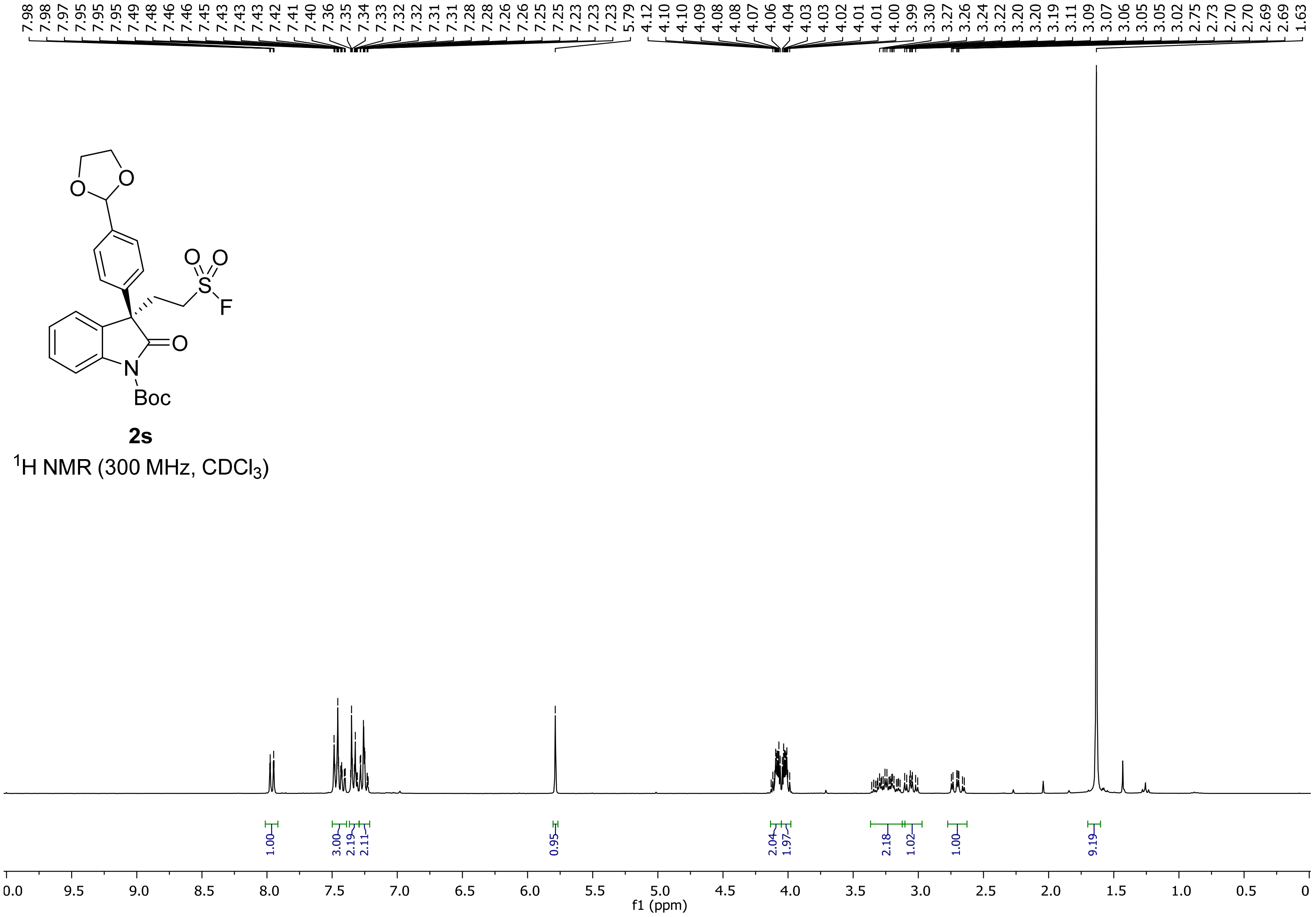

2s

^{1}H NMR (300 MHz, $CDCl_3$)

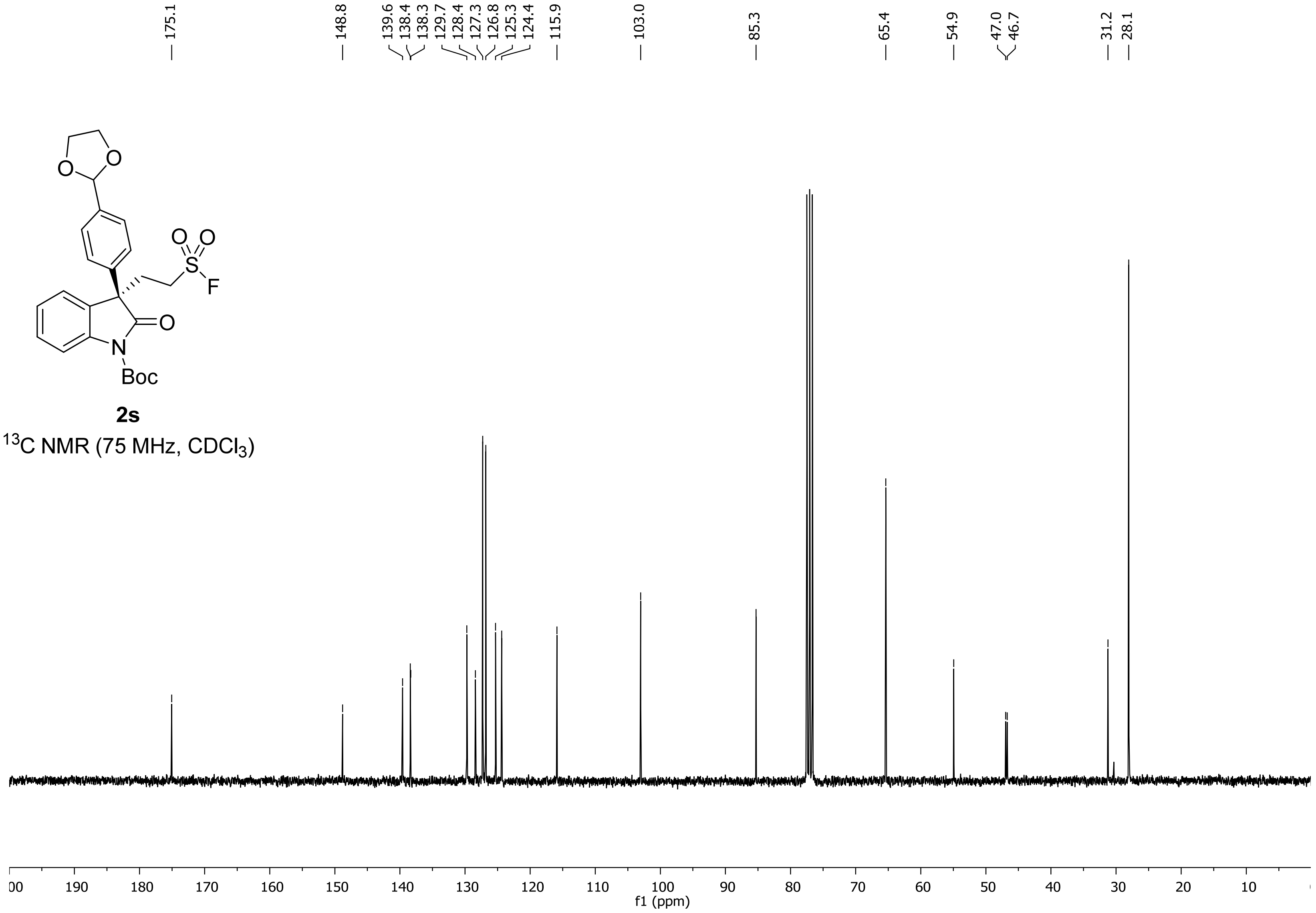

2s

^{13}C NMR (75 MHz, $CDCl_3$)

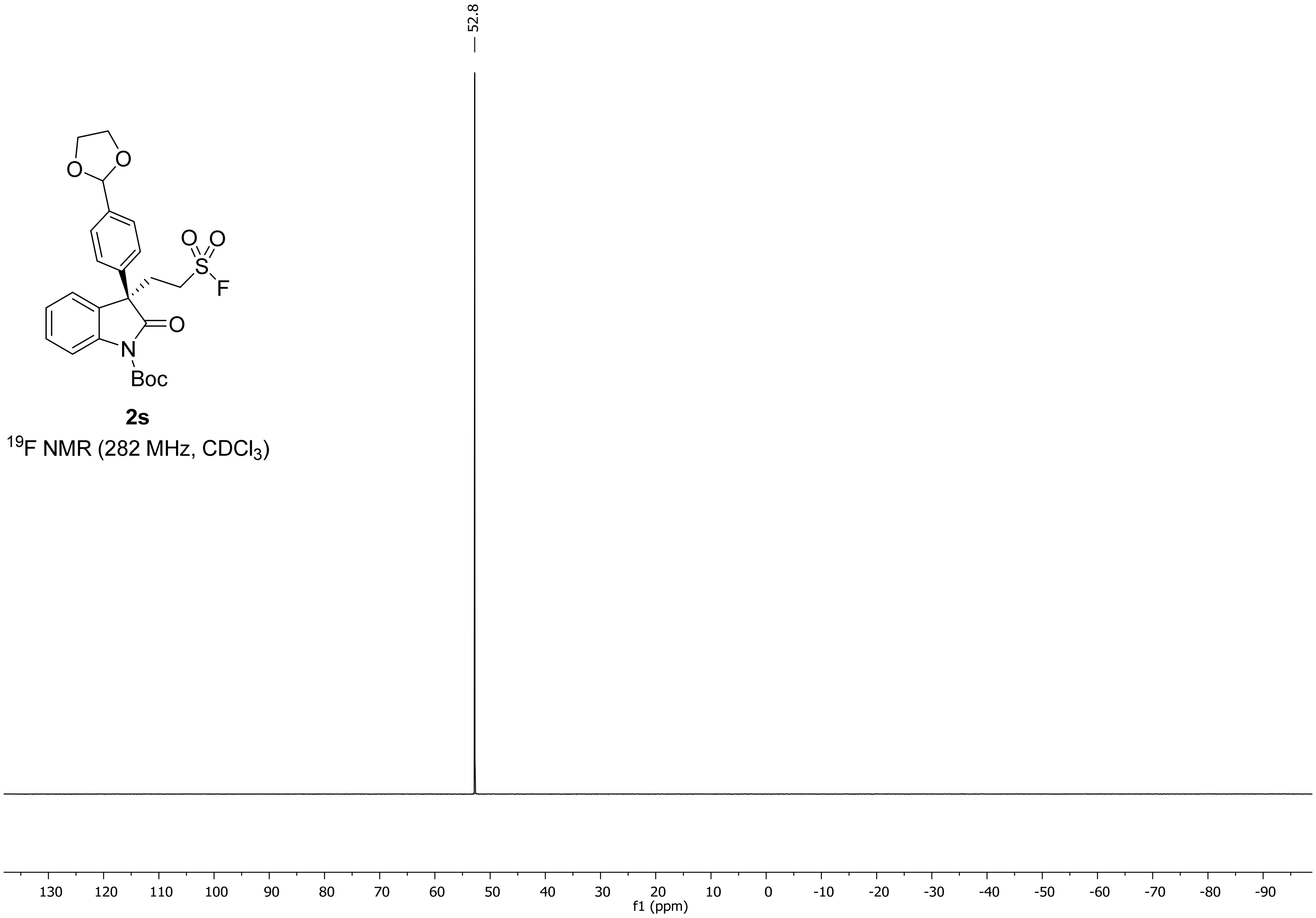

2s

^{19}F NMR (282 MHz, $CDCl_3$)

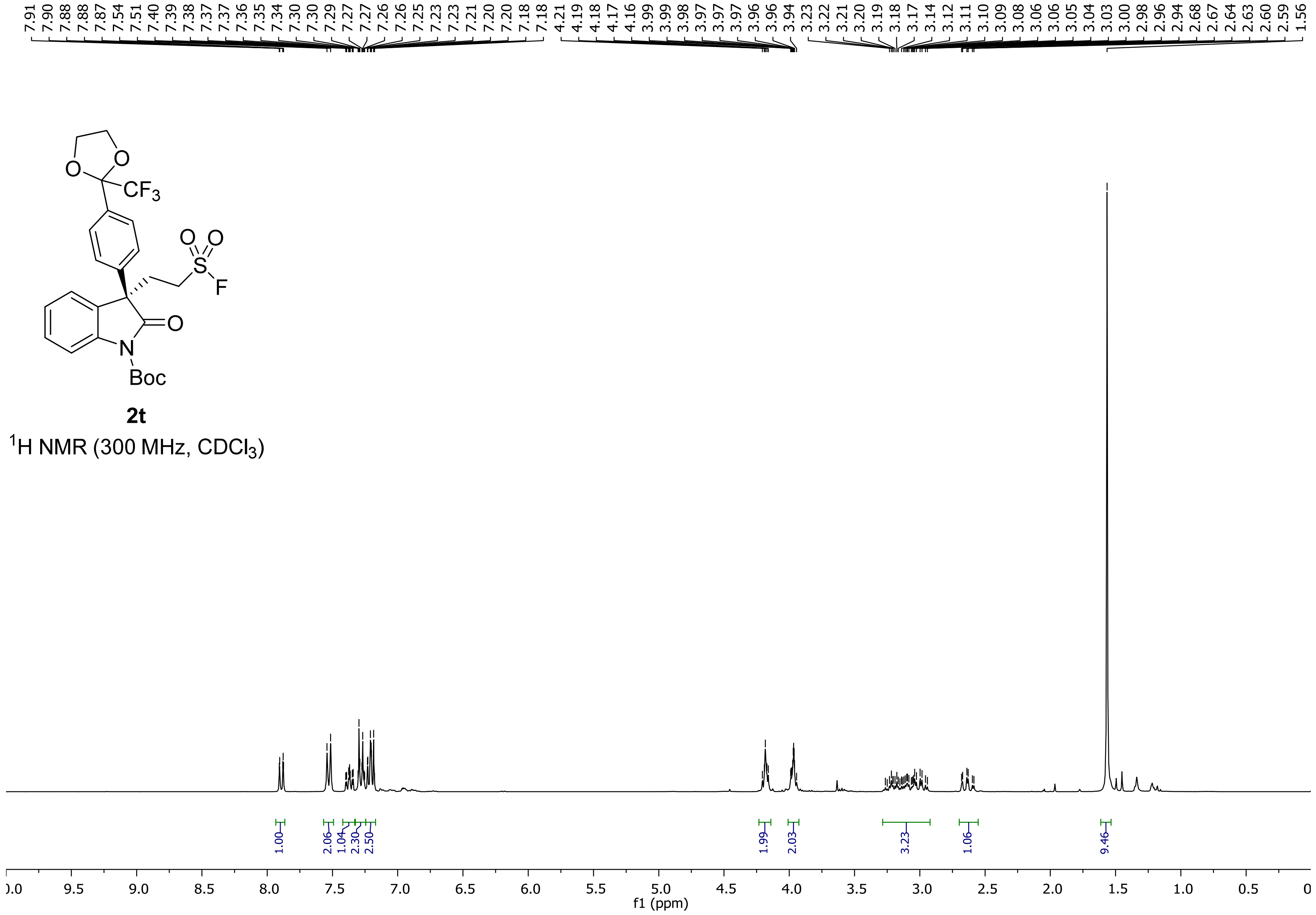

2t

^{1}H NMR (300 MHz, $CDCl_3$)

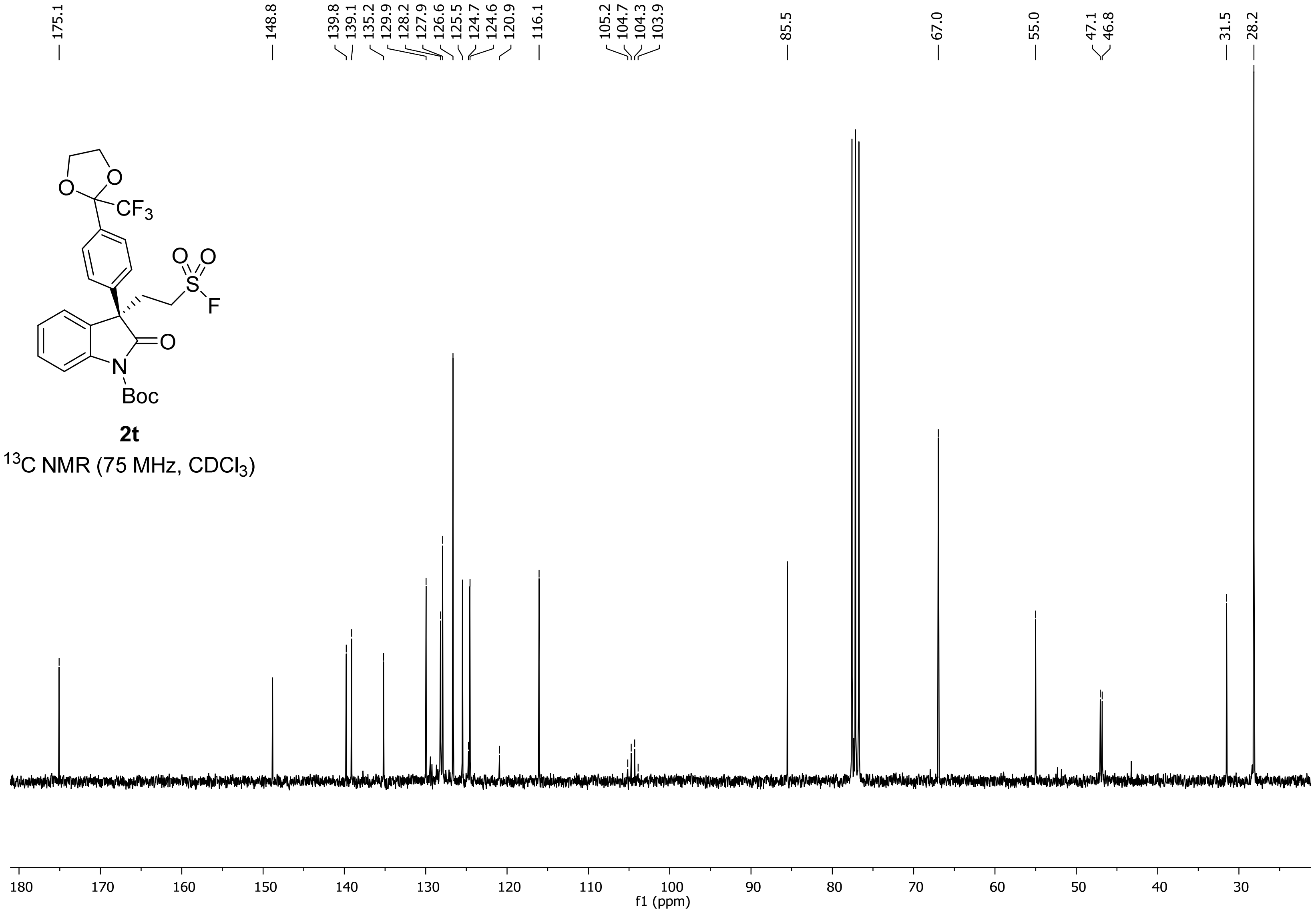

175.1
148.8
139.8
139.1
135.2
129.9
128.2
127.9
126.6
125.5
124.7
124.6
120.9
116.1
105.2
104.7
104.3
103.9
85.5
67.0
55.0
47.1
46.8
31.5
28.2
O
O
CF3
O
O
S
F
O
N
Boc
2t
13C NMR (75 MHz, CDCl3)
180
170
160
150
140
130
120
110
100
90
80
70
60
50
40
30
f1 (ppm)

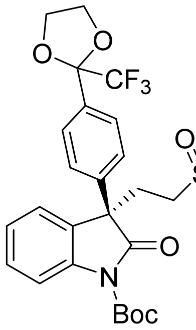

2t

^{19}F NMR (282 MHz, $CDCl_3$)

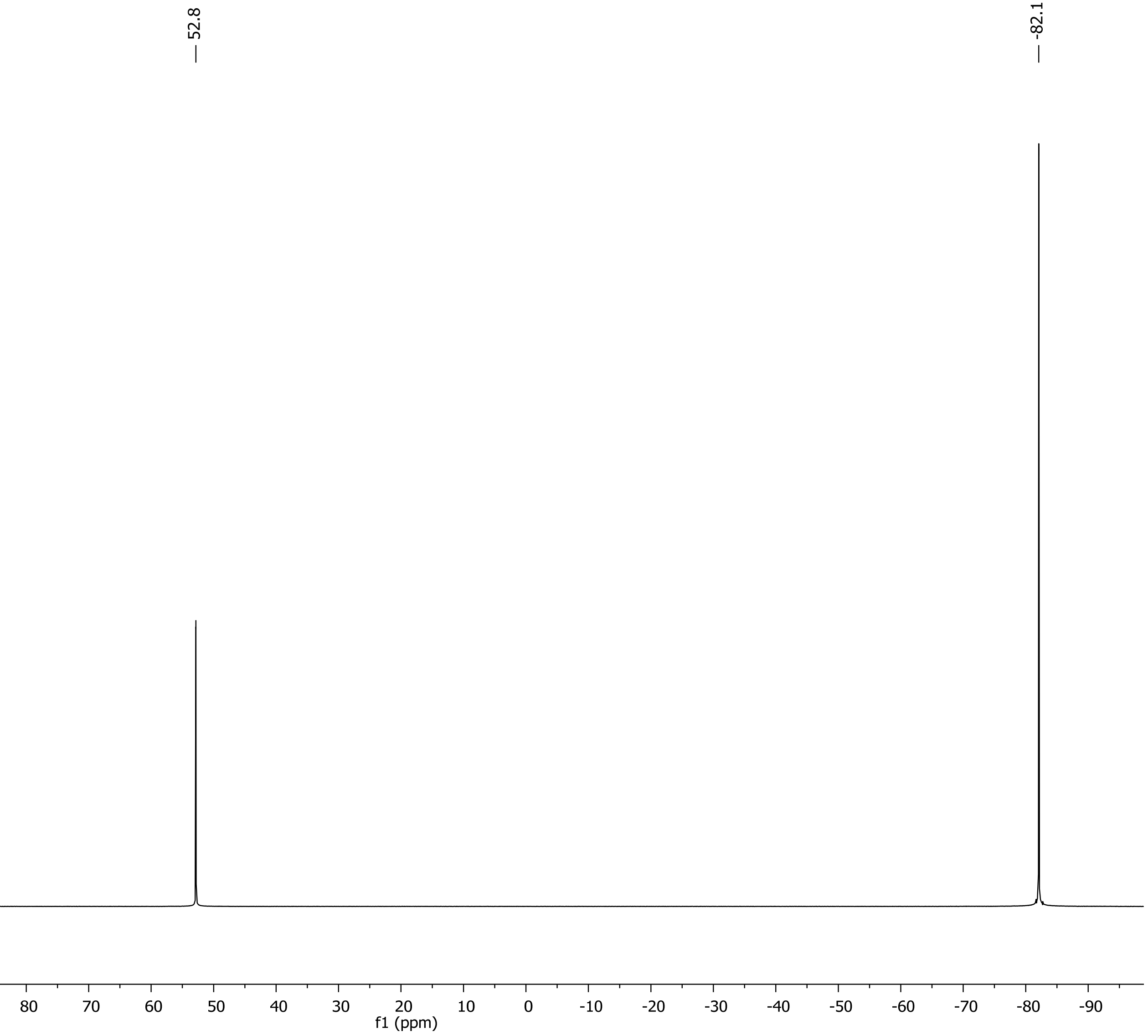

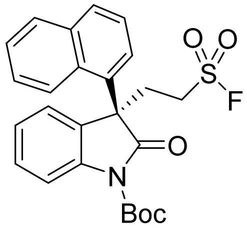

2u

^{1}H NMR (300 MHz, $CDCl_3$)

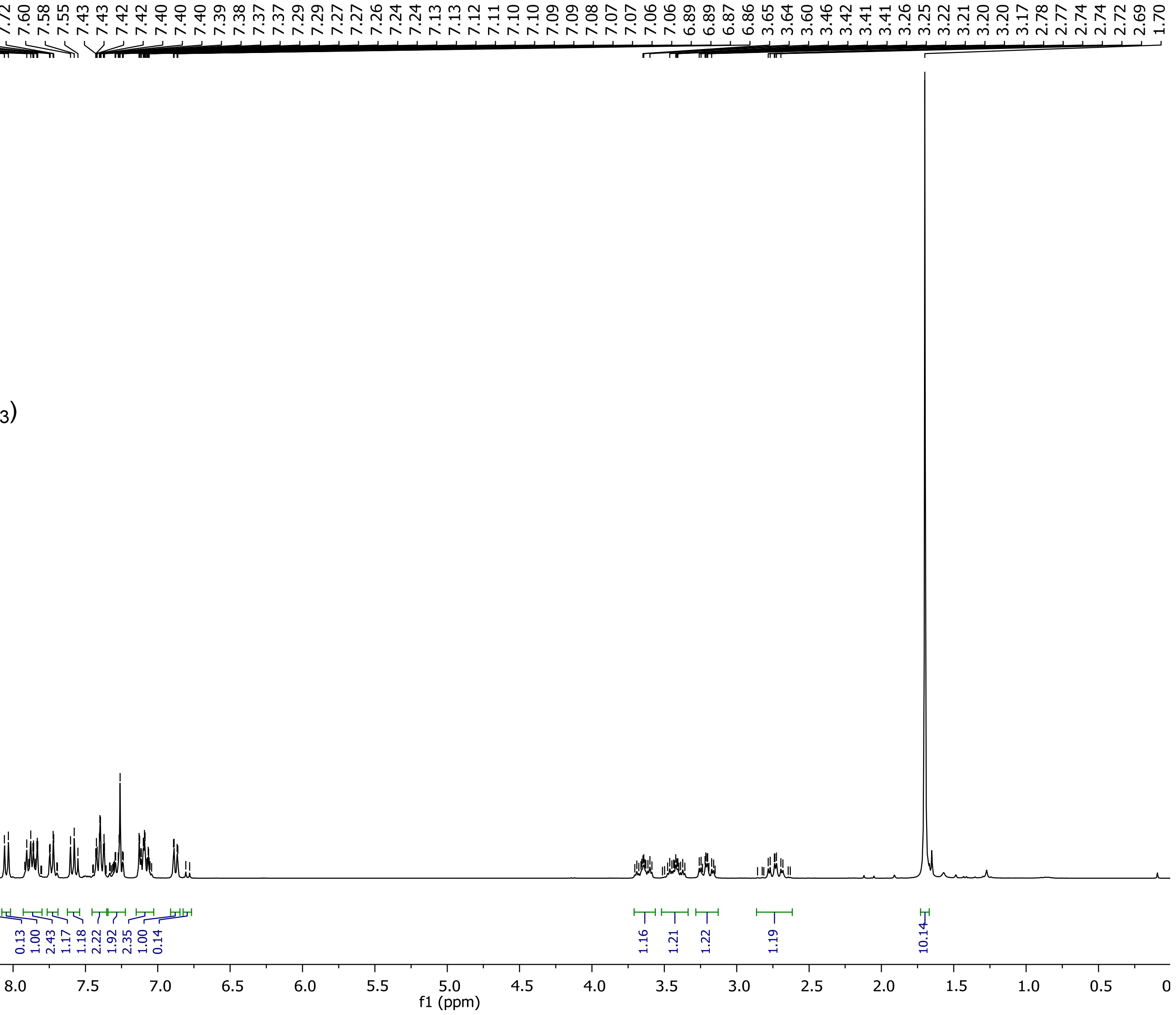

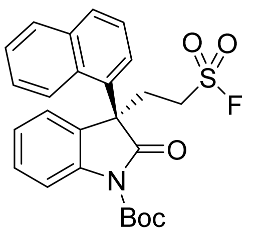

2u

^{13}C NMR (75 MHz, $CDCl_3$)

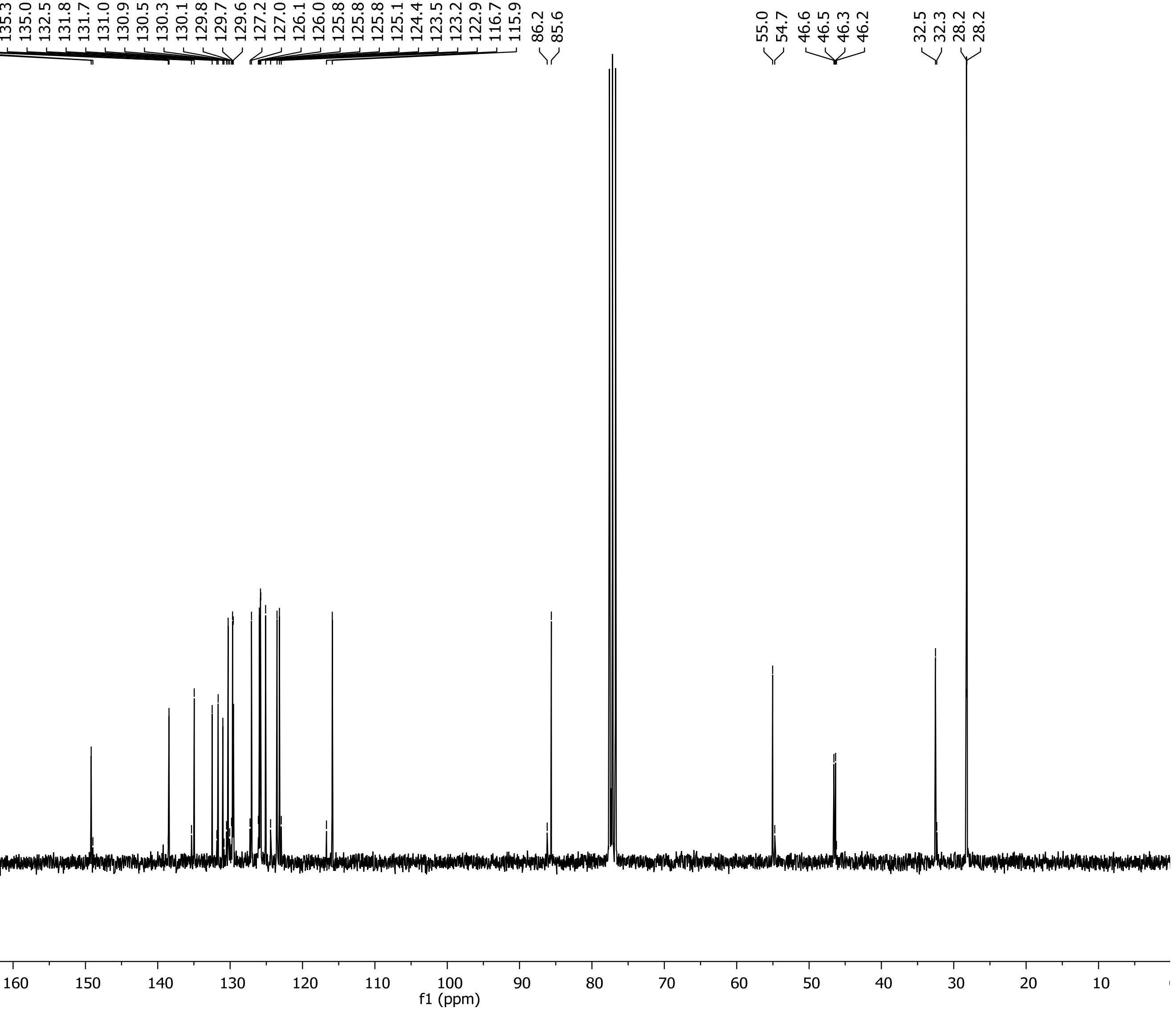

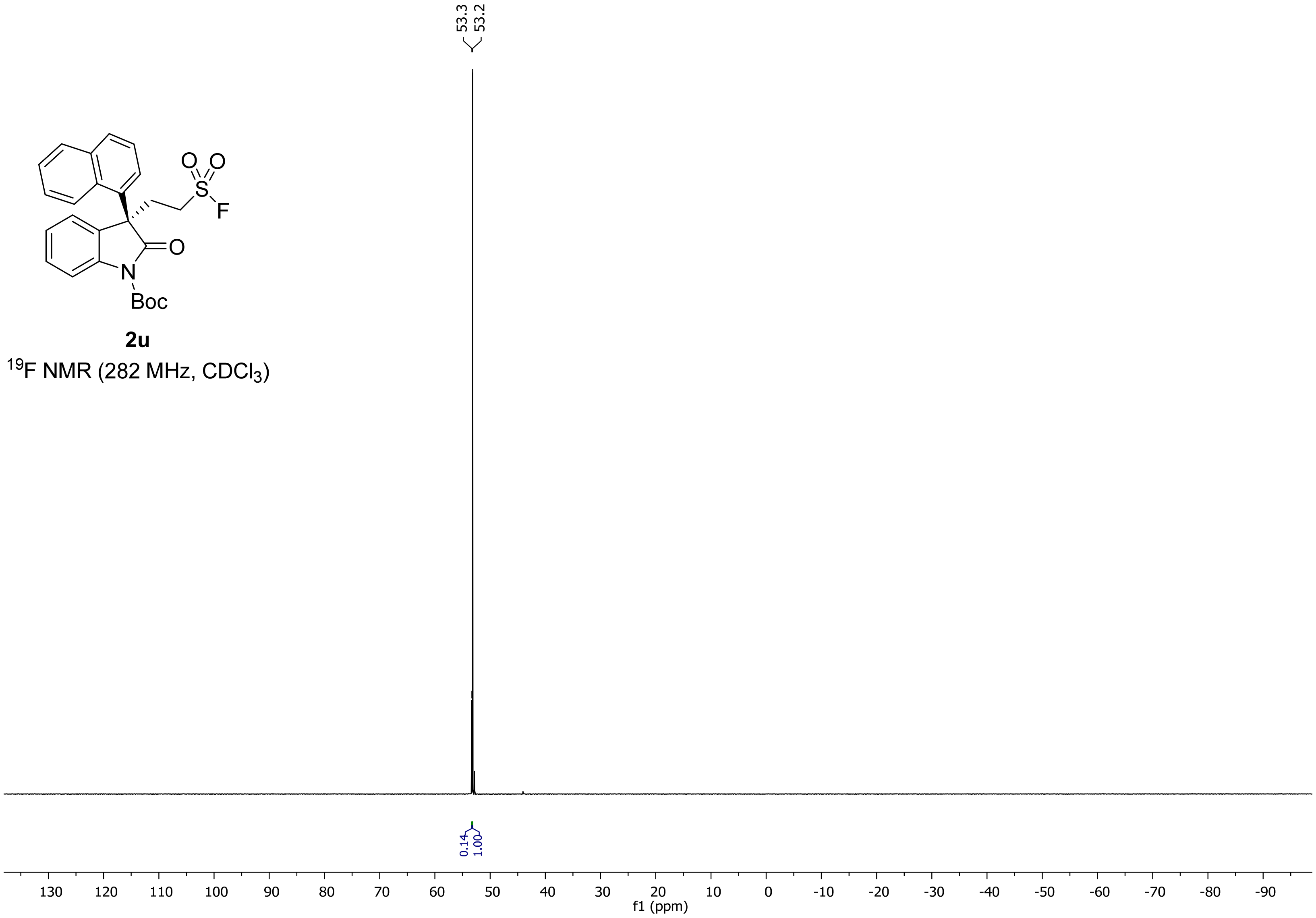

2u

^{19}F NMR (282 MHz, $CDCl_3$)

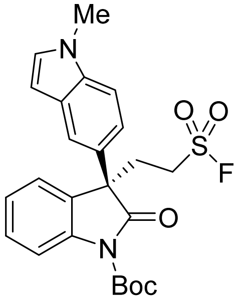

2v

^{1}H NMR (300 MHz, $CDCl_3$)

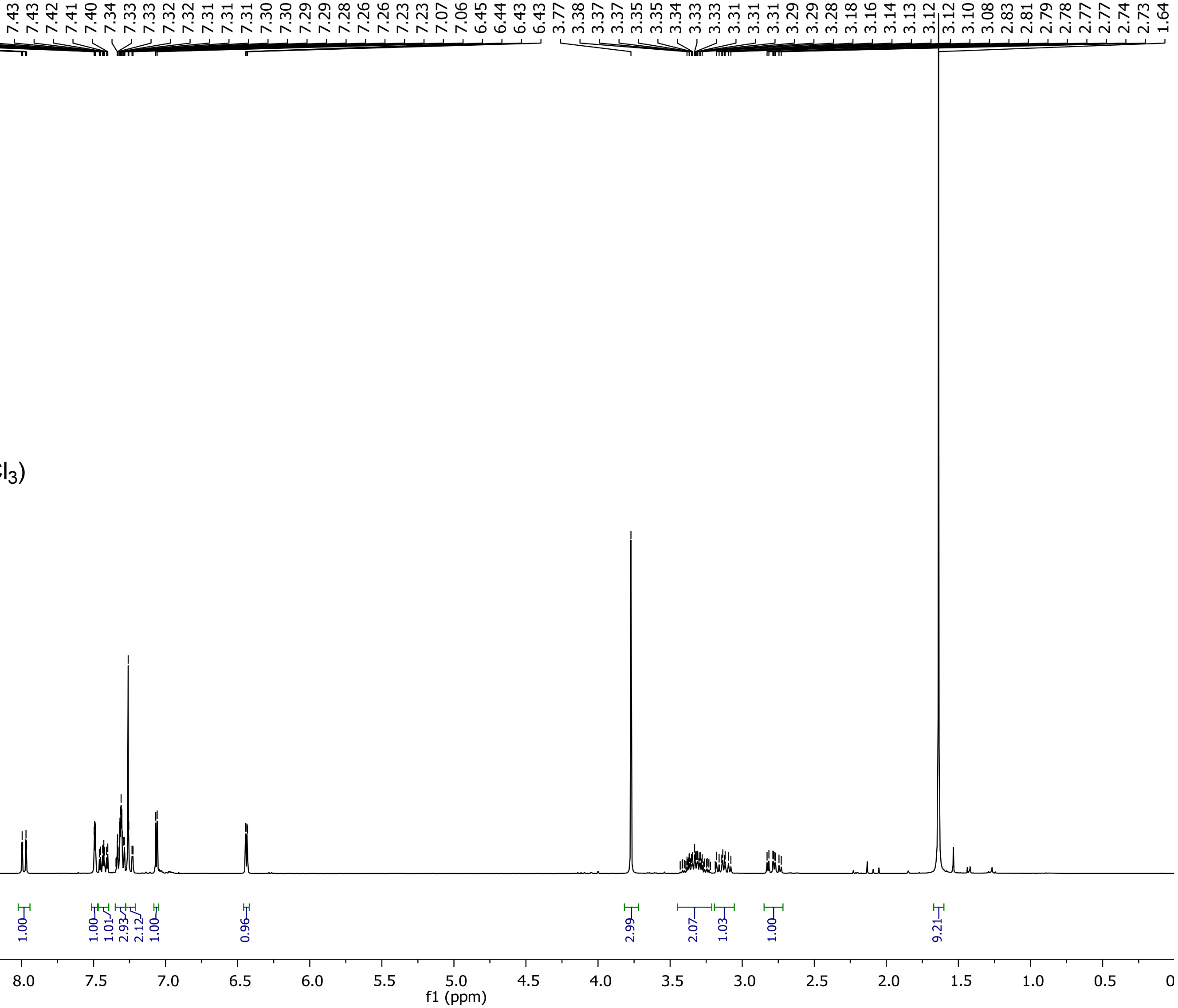

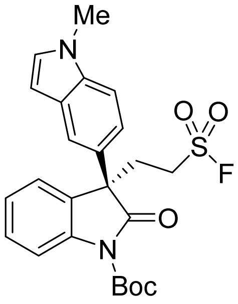

2v

^{13}C NMR (75 MHz, $CDCl_3$)

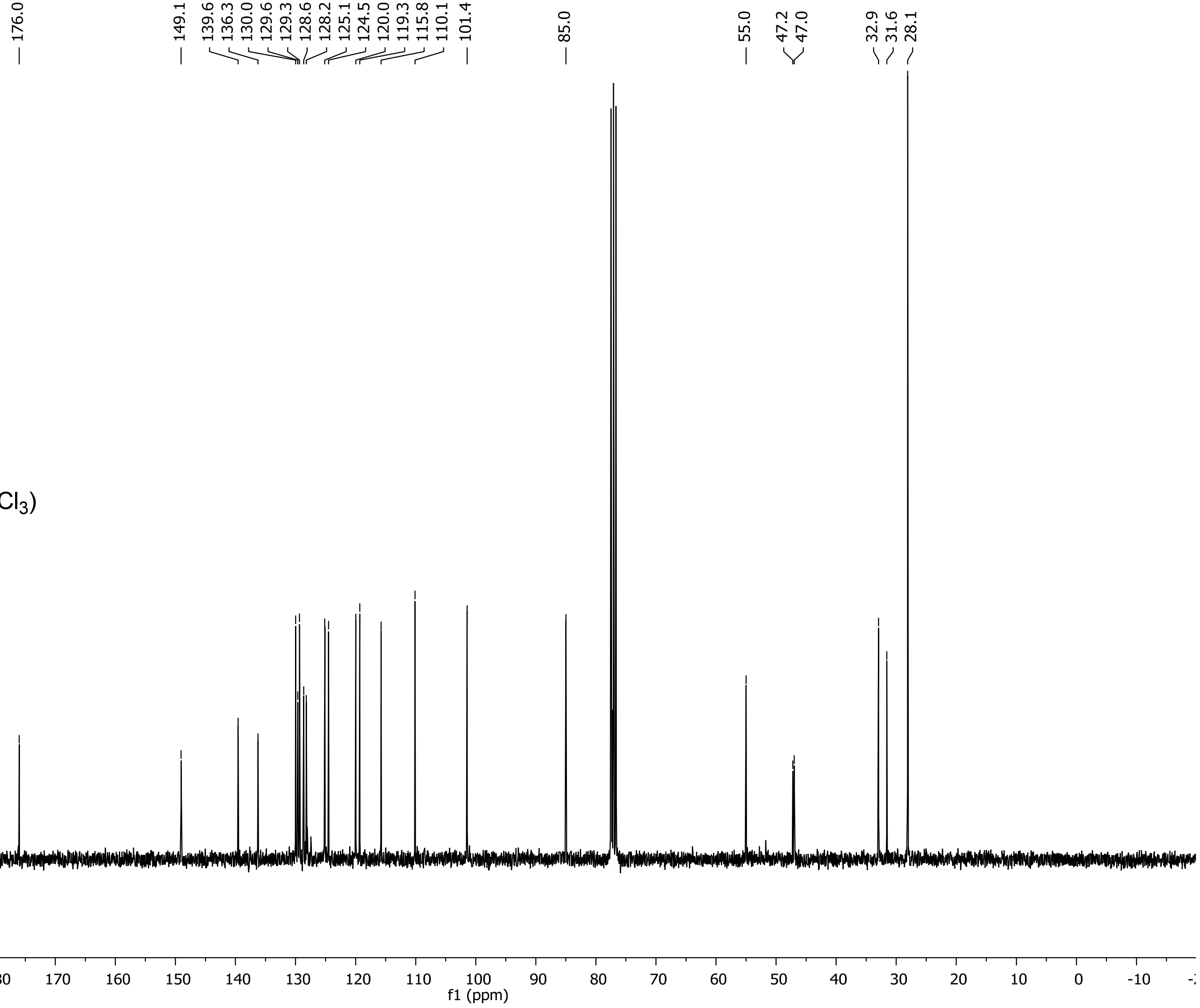

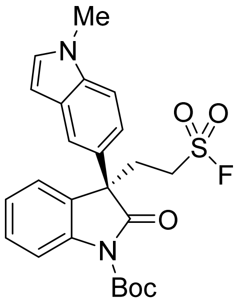

2v

^{19}F NMR (282 MHz, $CDCl_3$)

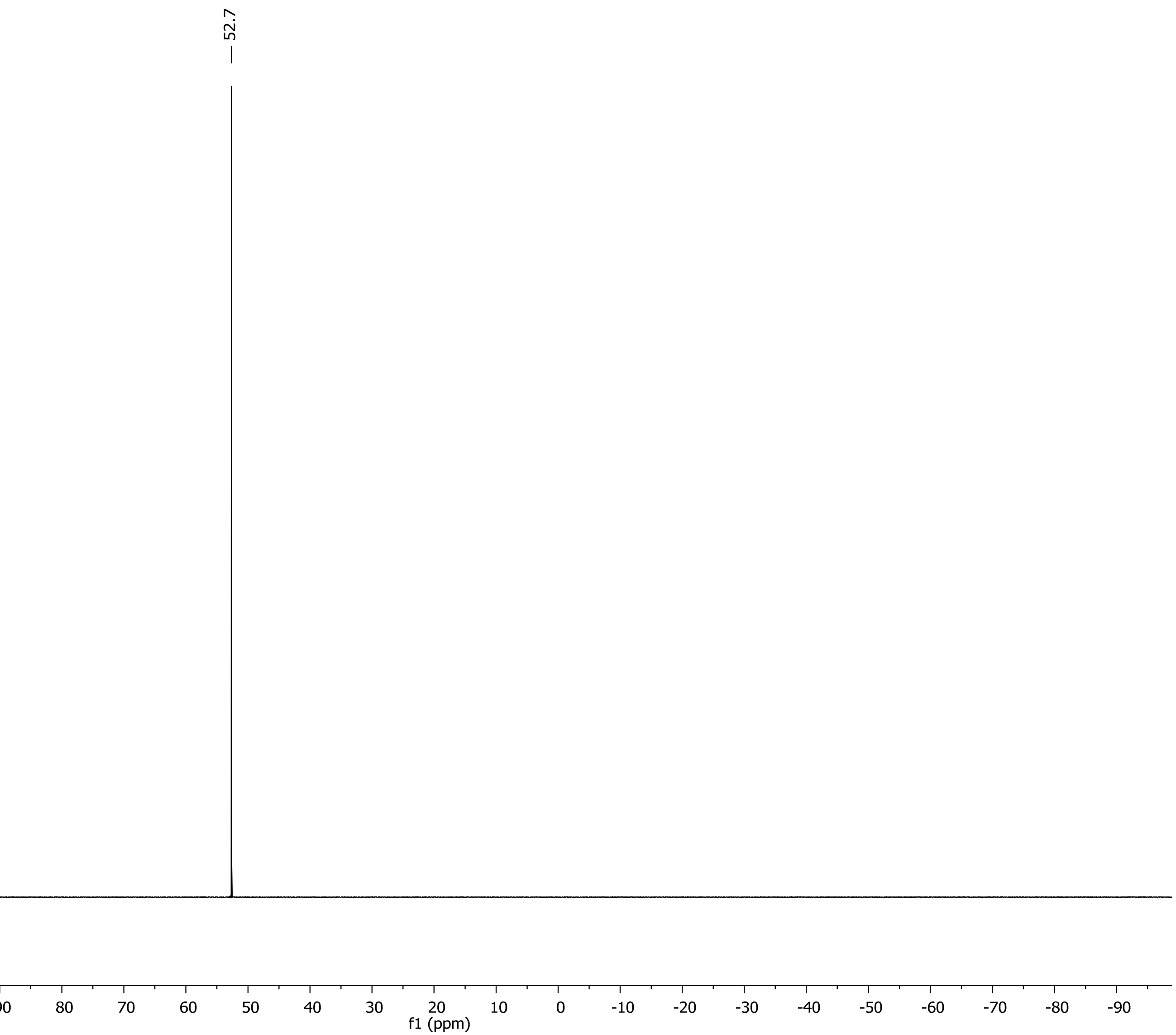

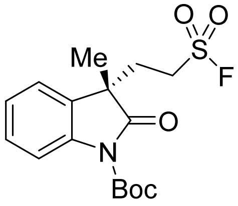

2w

^{1}H NMR (300 MHz, $CDCl_3$)

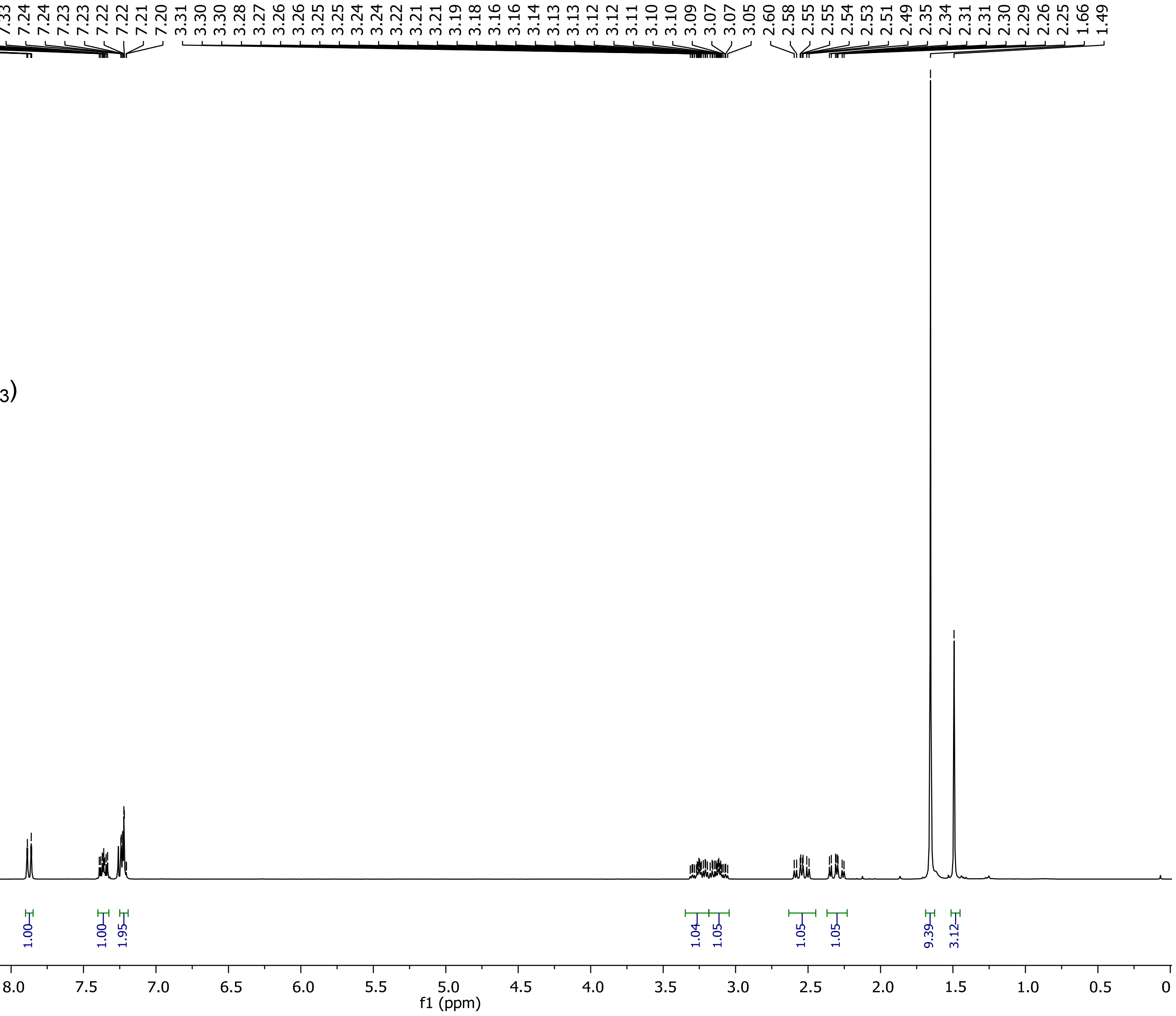

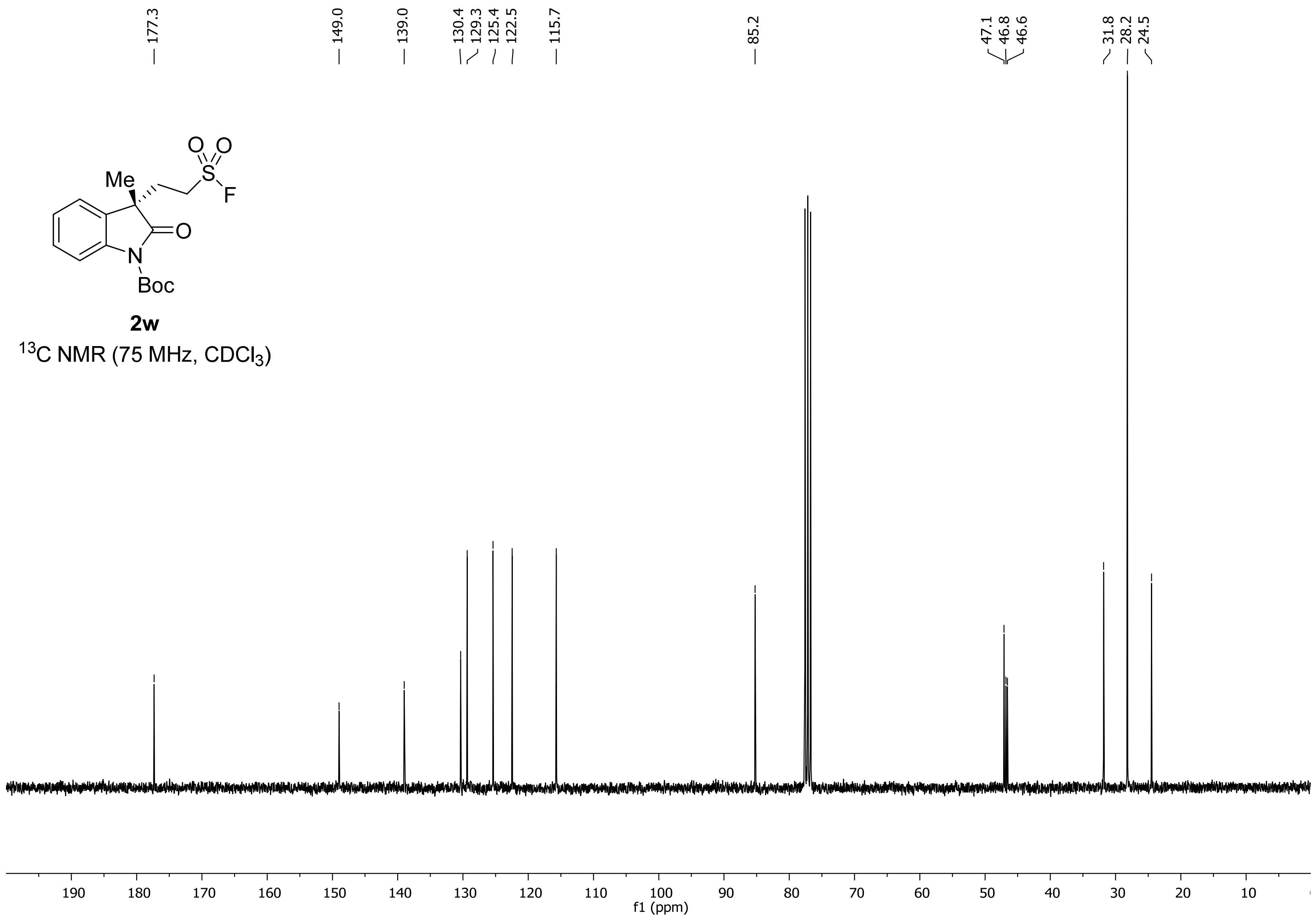

2w

^{13}C NMR (75 MHz, $CDCl_3$)

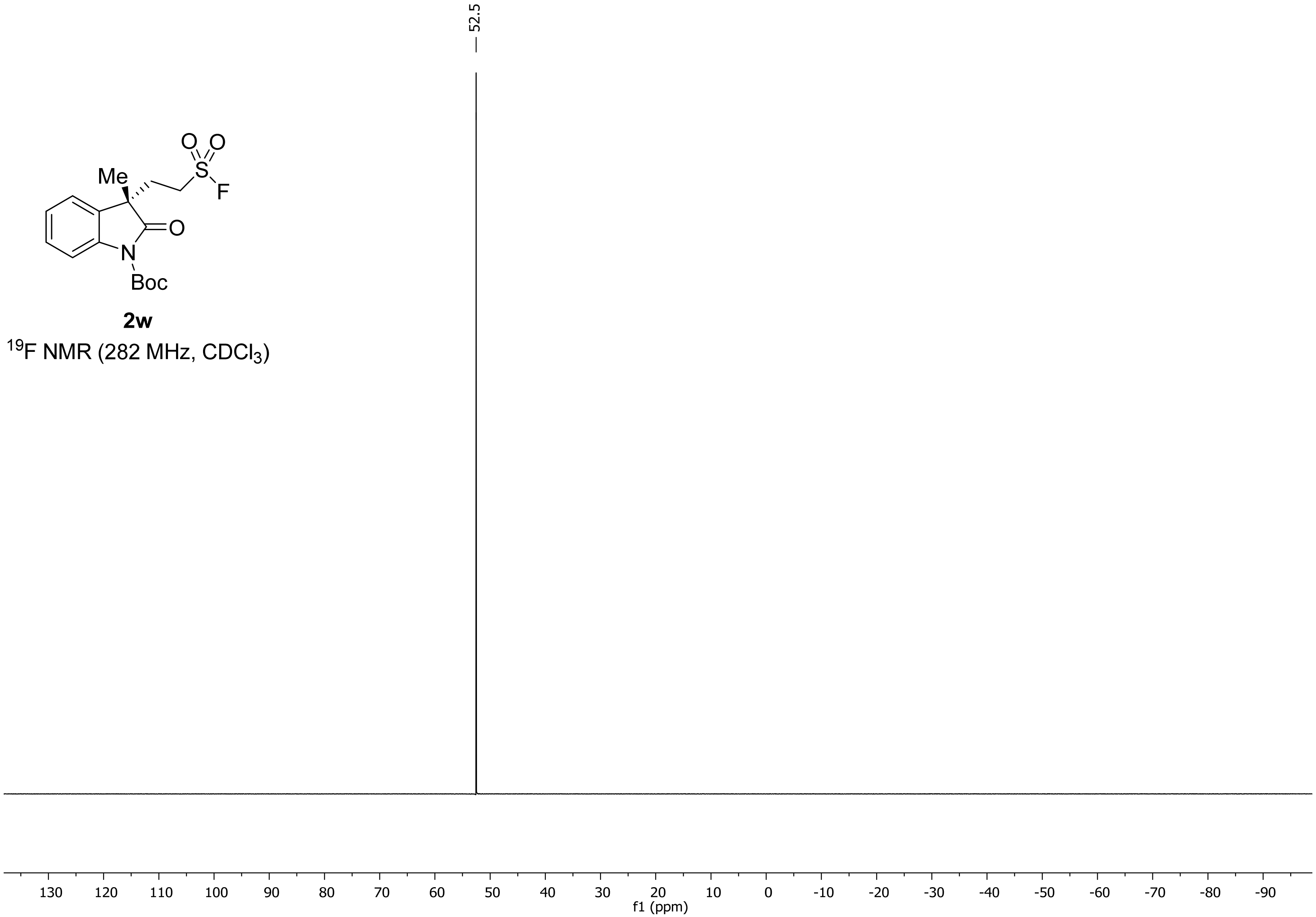

2w
19F NMR (282 MHz, CDCl3)
52.5
130
120
110
100
90
80
70
60
50
40
30
20
10
0
-10
-20
-30
-40
-50
-60
-70
-80
-90
f1 (ppm)

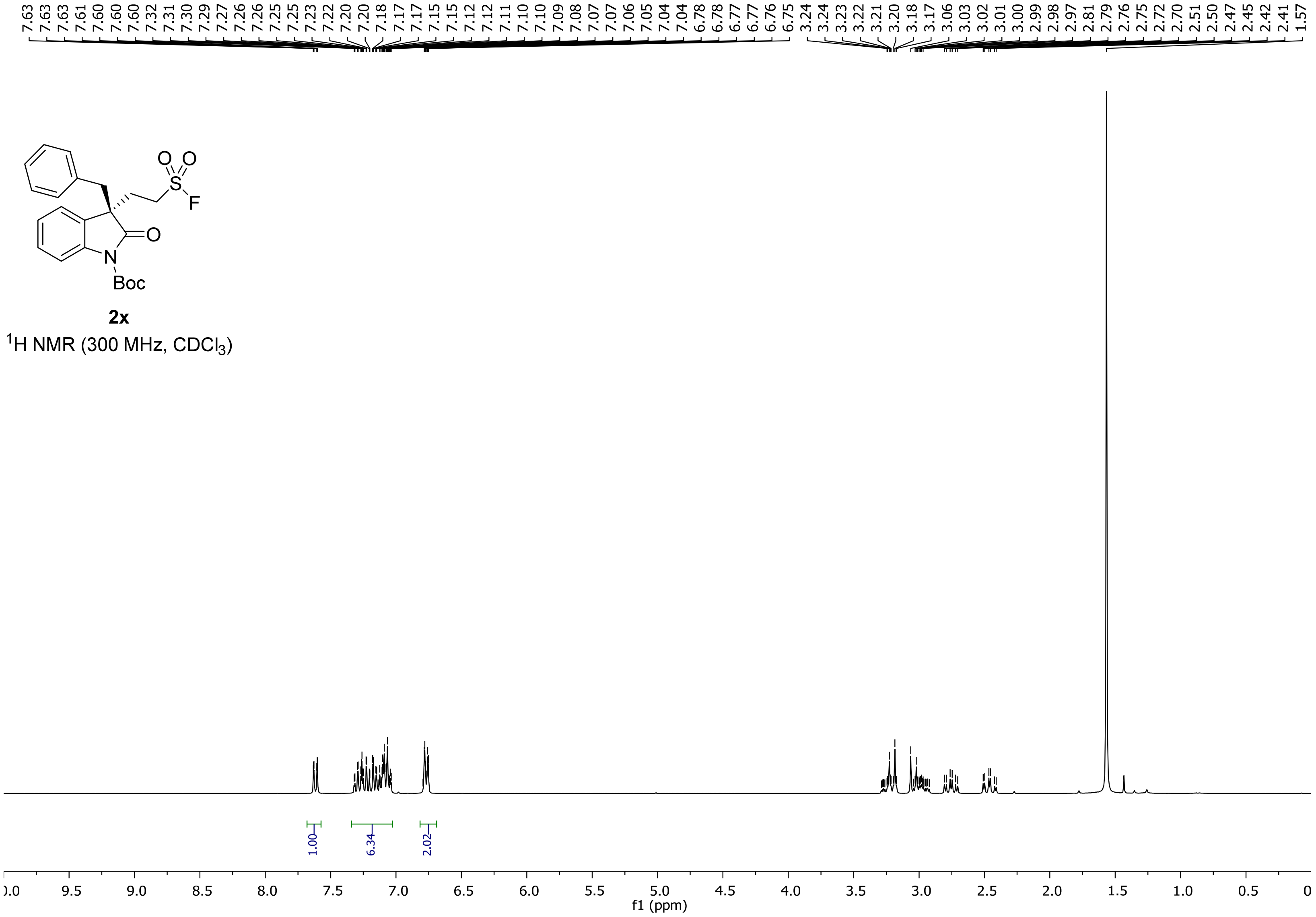

2x

^{1}H NMR (300 MHz, $CDCl_3$)

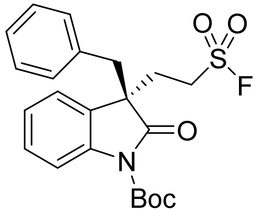

2x

^{13}C NMR (75 MHz, $CDCl_3$)

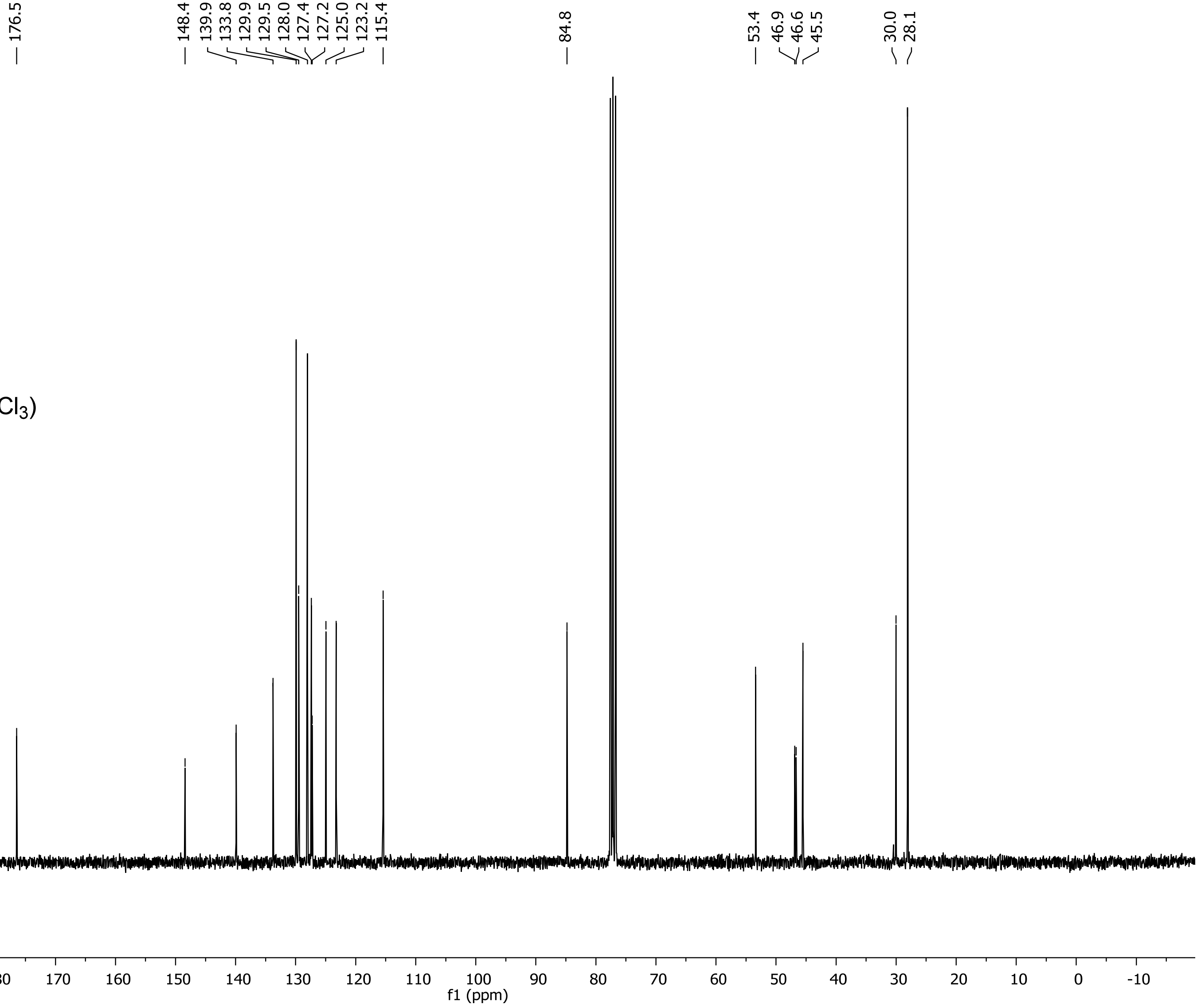

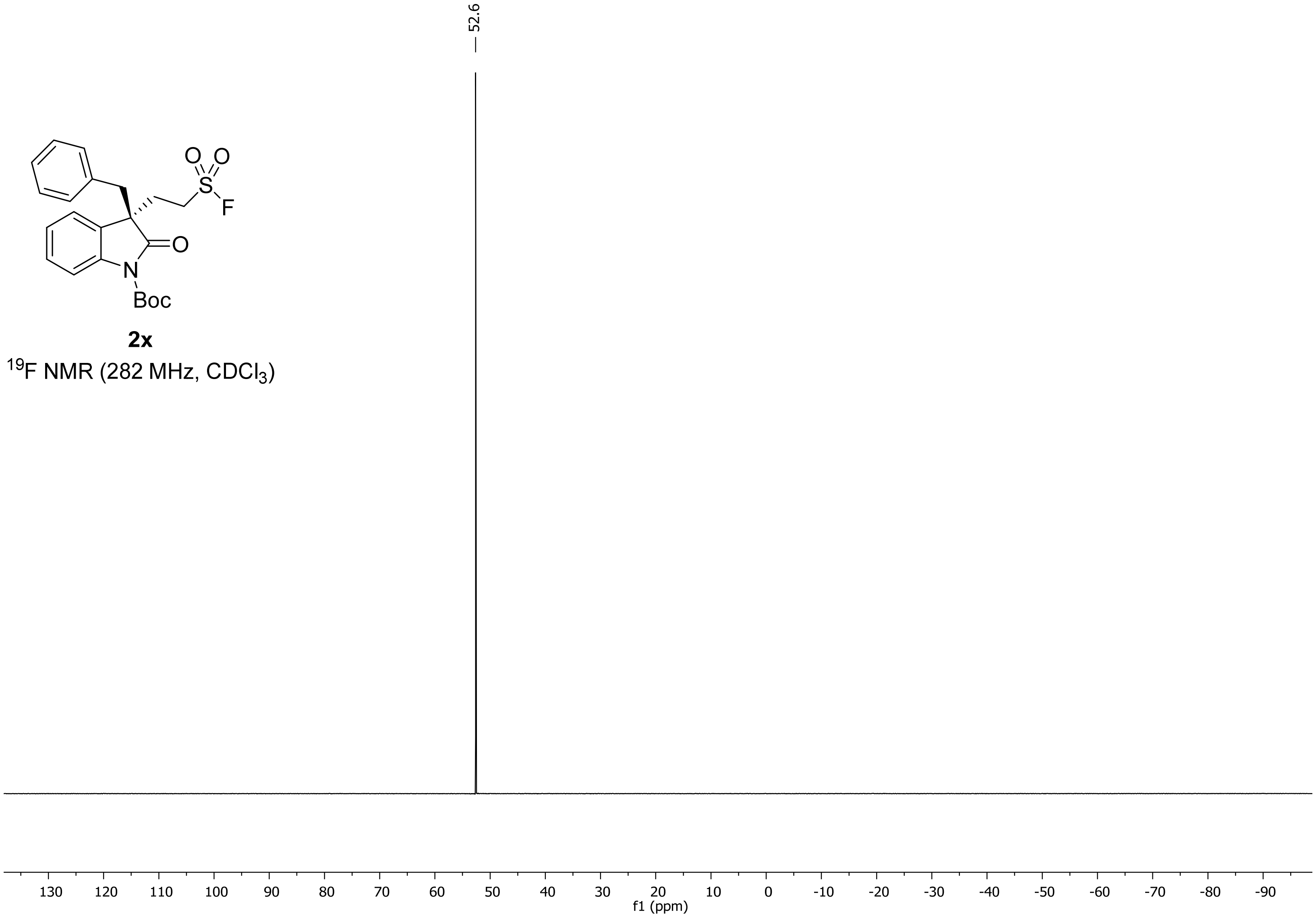

2x

^{19}F NMR (282 MHz, $CDCl_3$)

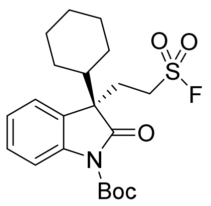

2y

^{1}H NMR (500 MHz, $CDCl_3$)

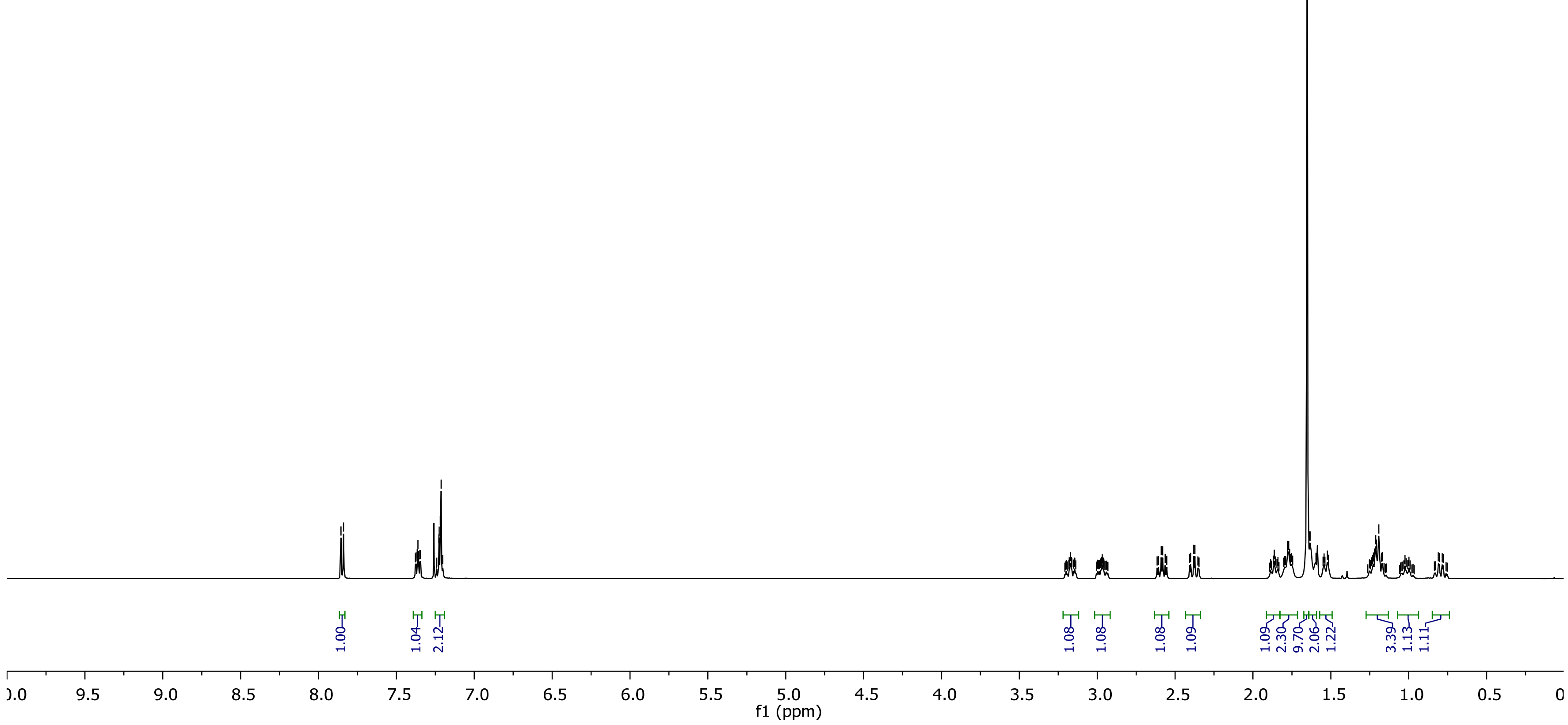

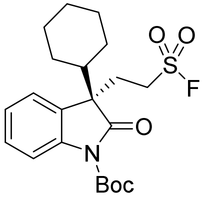

2y

^{13}C NMR (500 MHz, $CDCl_3$)

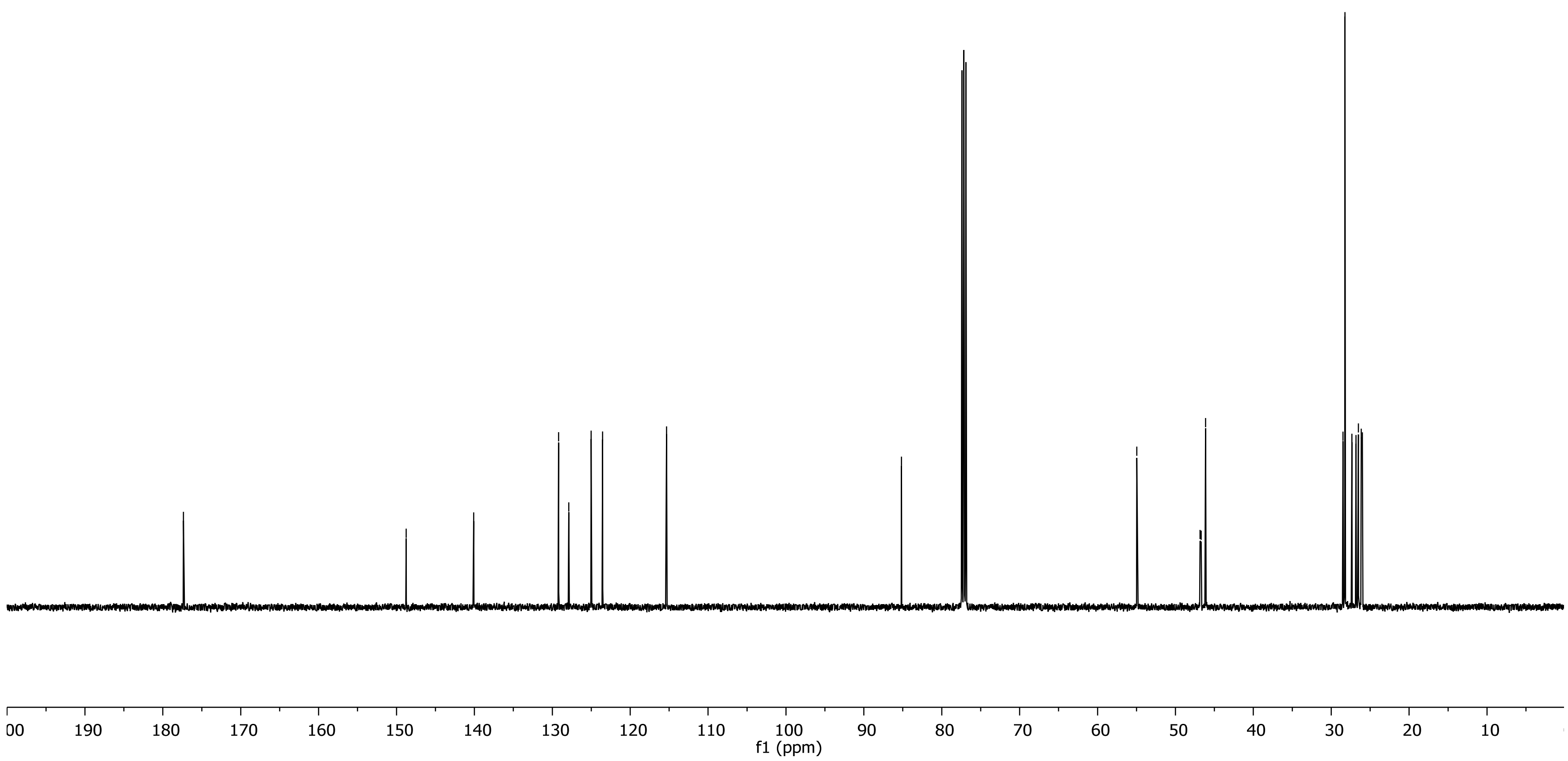

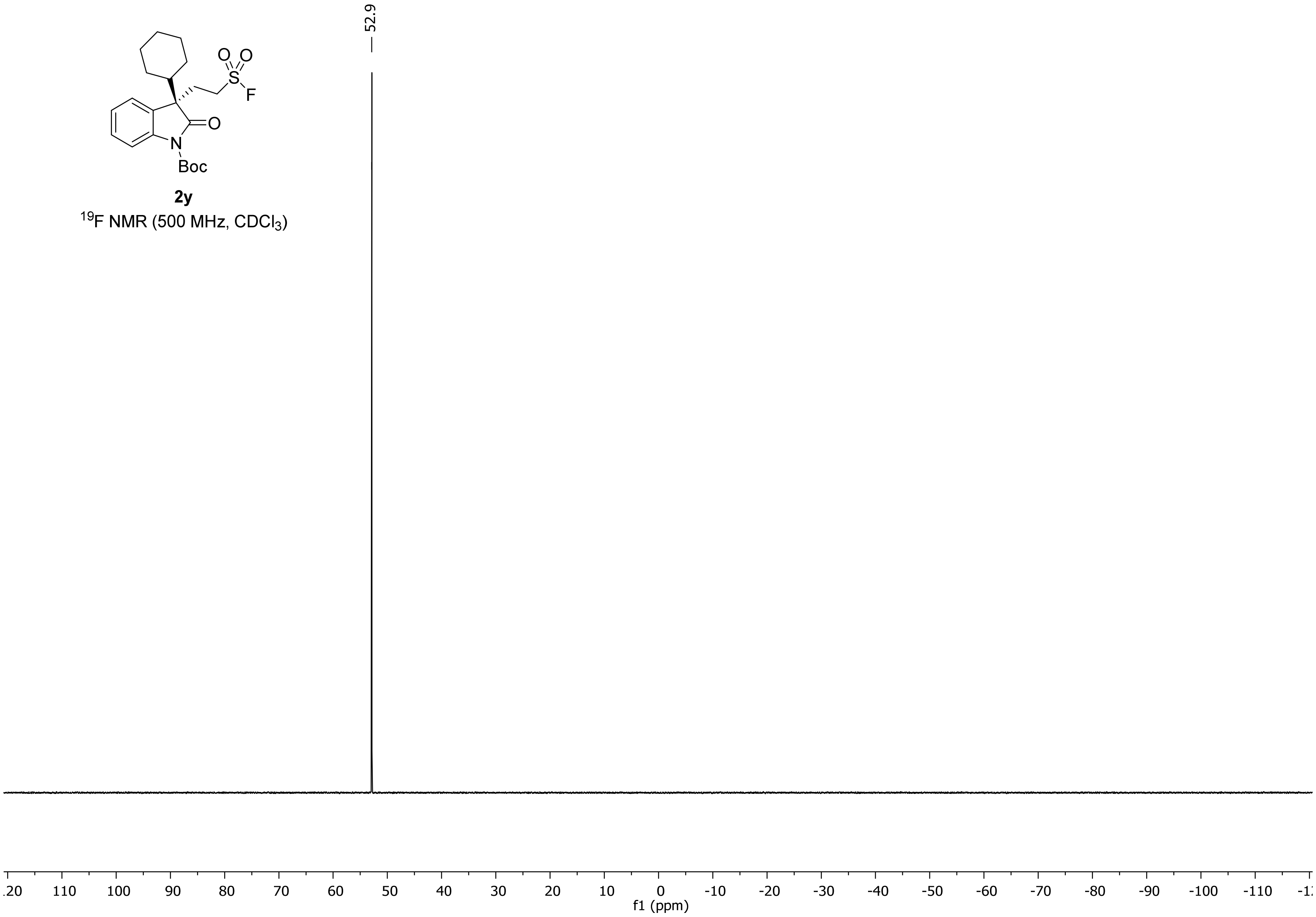

2y

^{19}F NMR (500 MHz, $CDCl_3$)

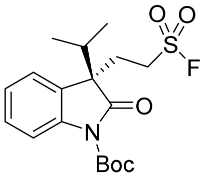

2z

^{1}H NMR (500 MHz, $CDCl_3$)

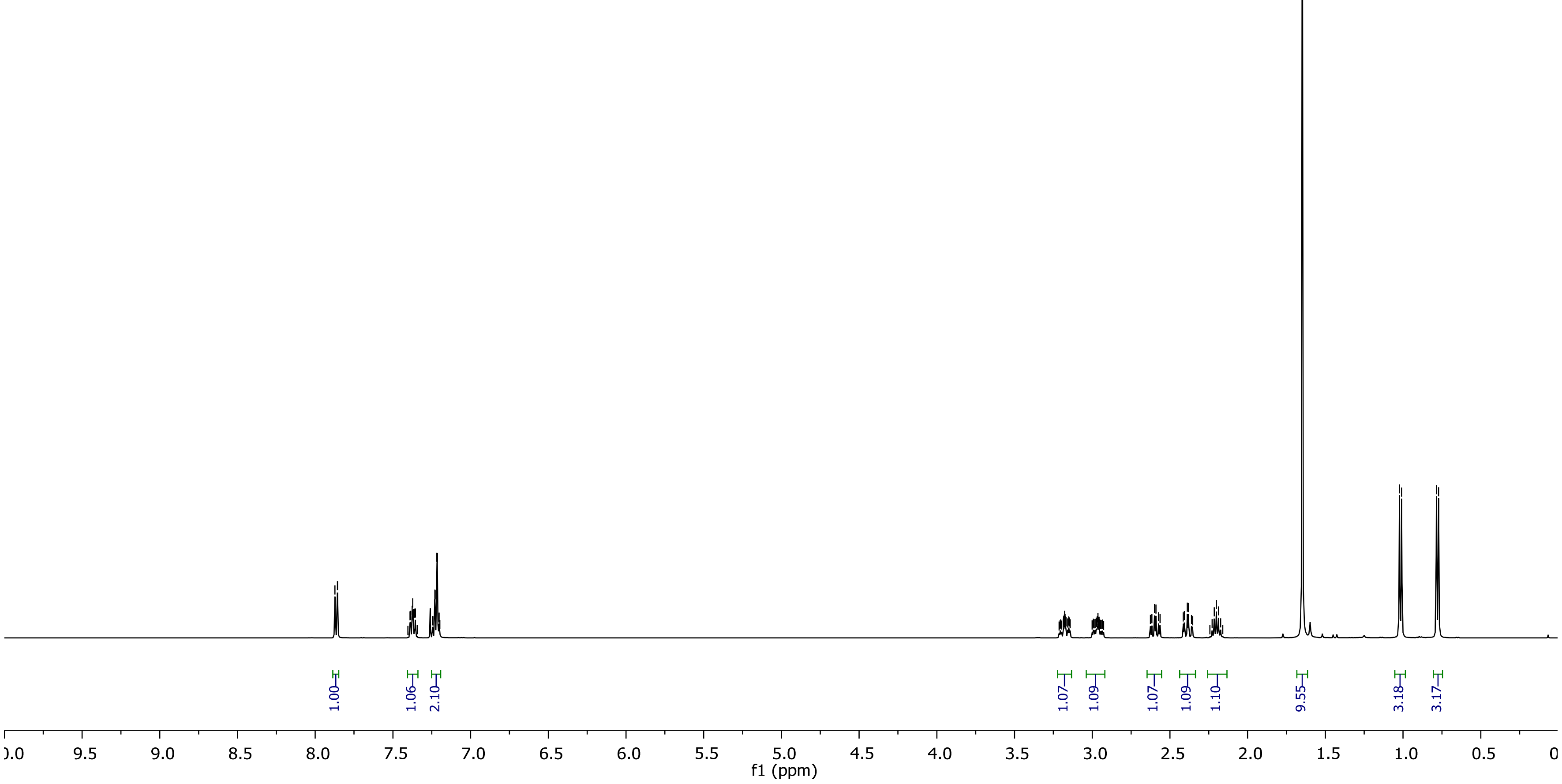

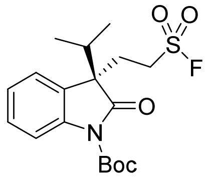

2z

^{13}C NMR (500 MHz, $CDCl_3$)

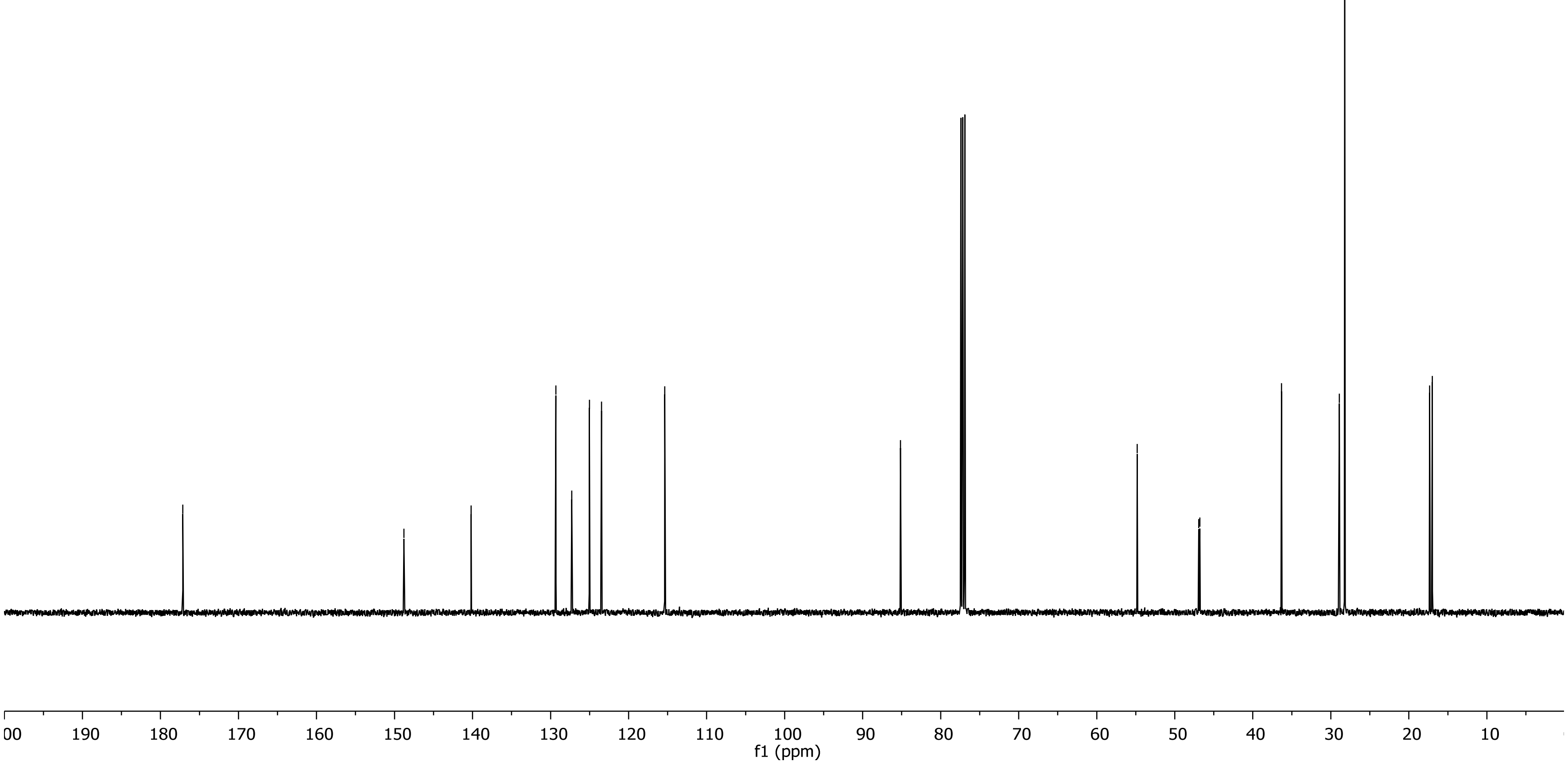

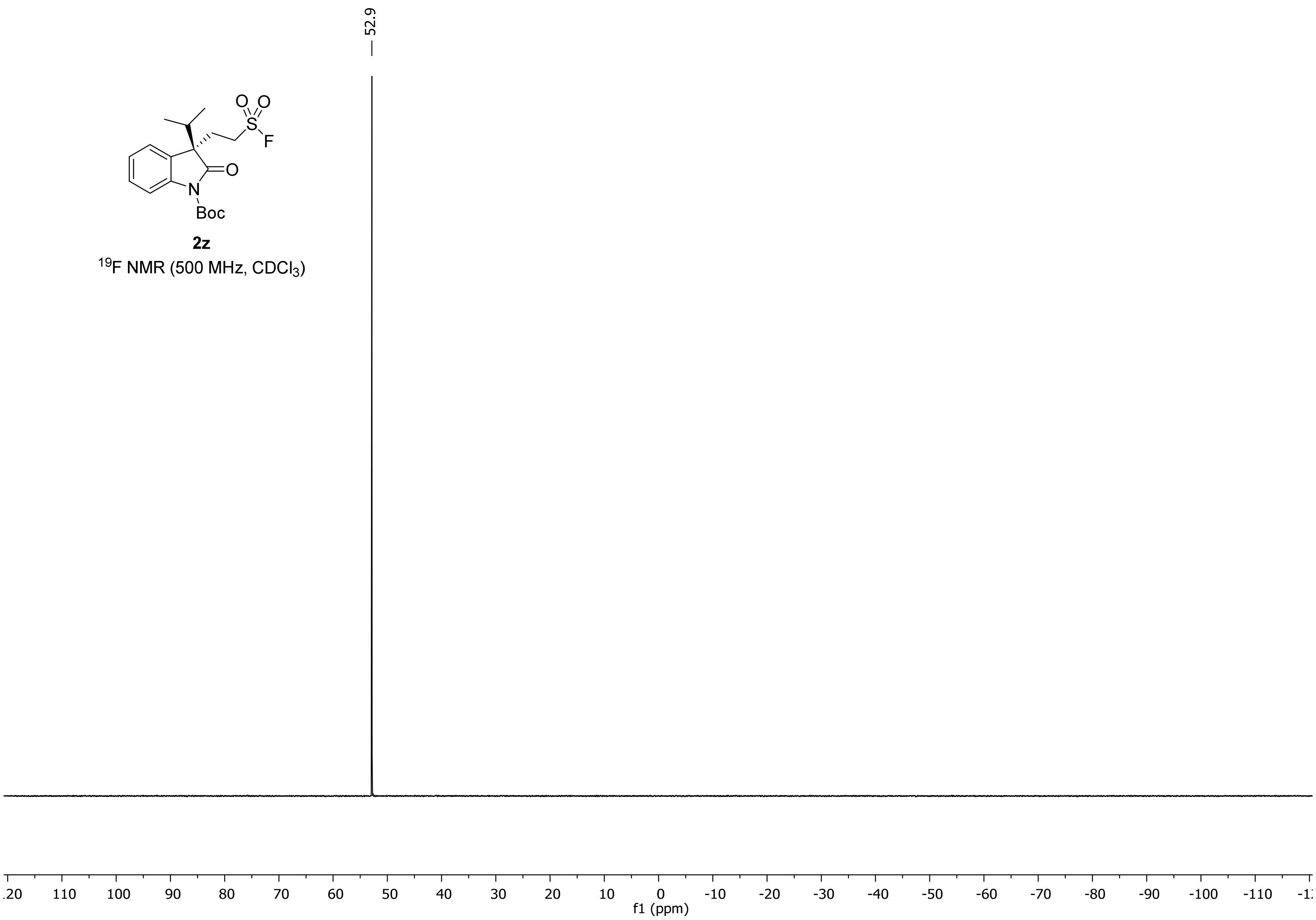
52.9
2z
19F NMR (500 MHz, CDCl3)
Boc
f1 (ppm)
110 100 90 80 70 60 50 40 30 20 10 0 -10 -20 -30 -40 -50 -60 -70 -80 -90 -100 -110

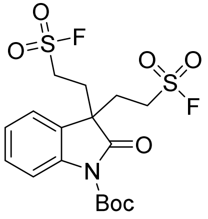

17

^{1}H NMR (300 MHz, $CDCl_3$)

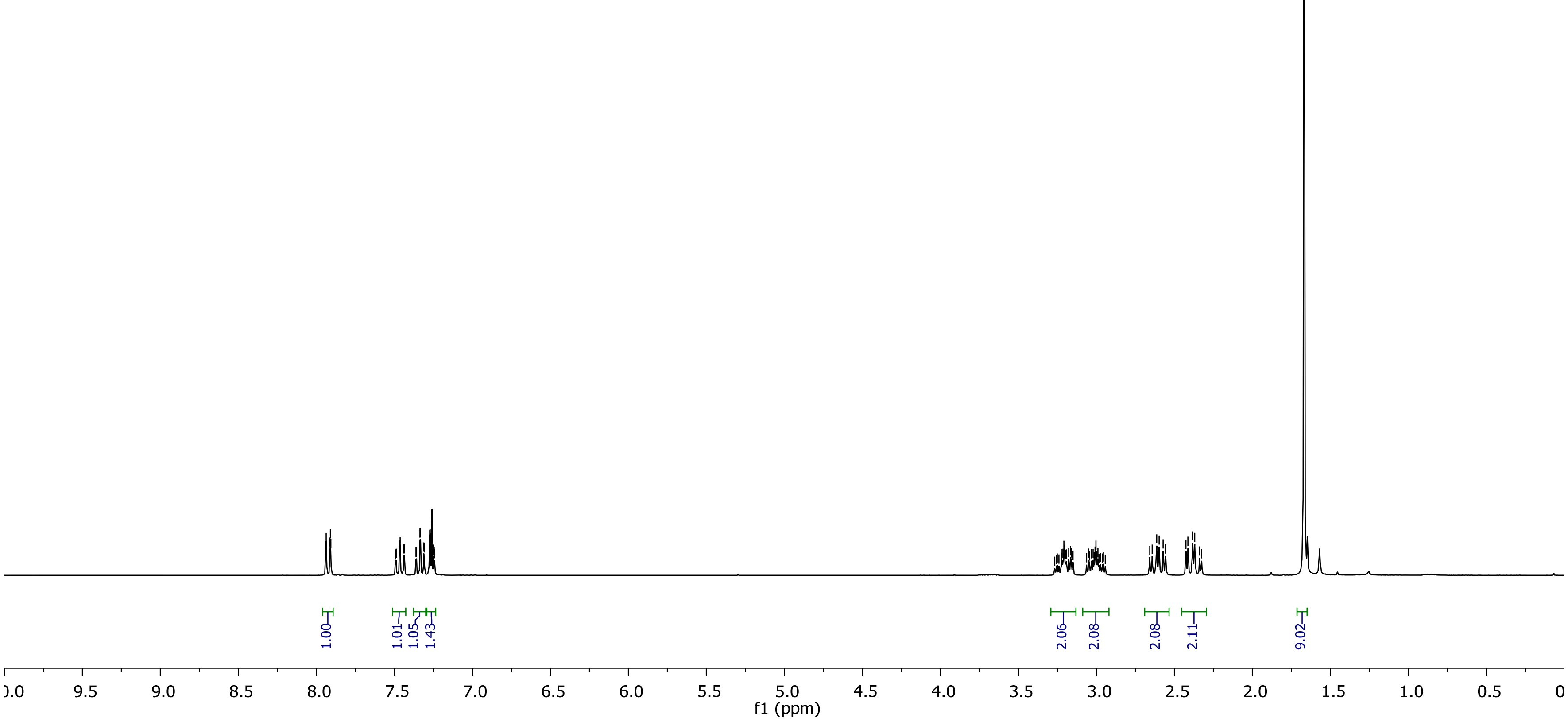

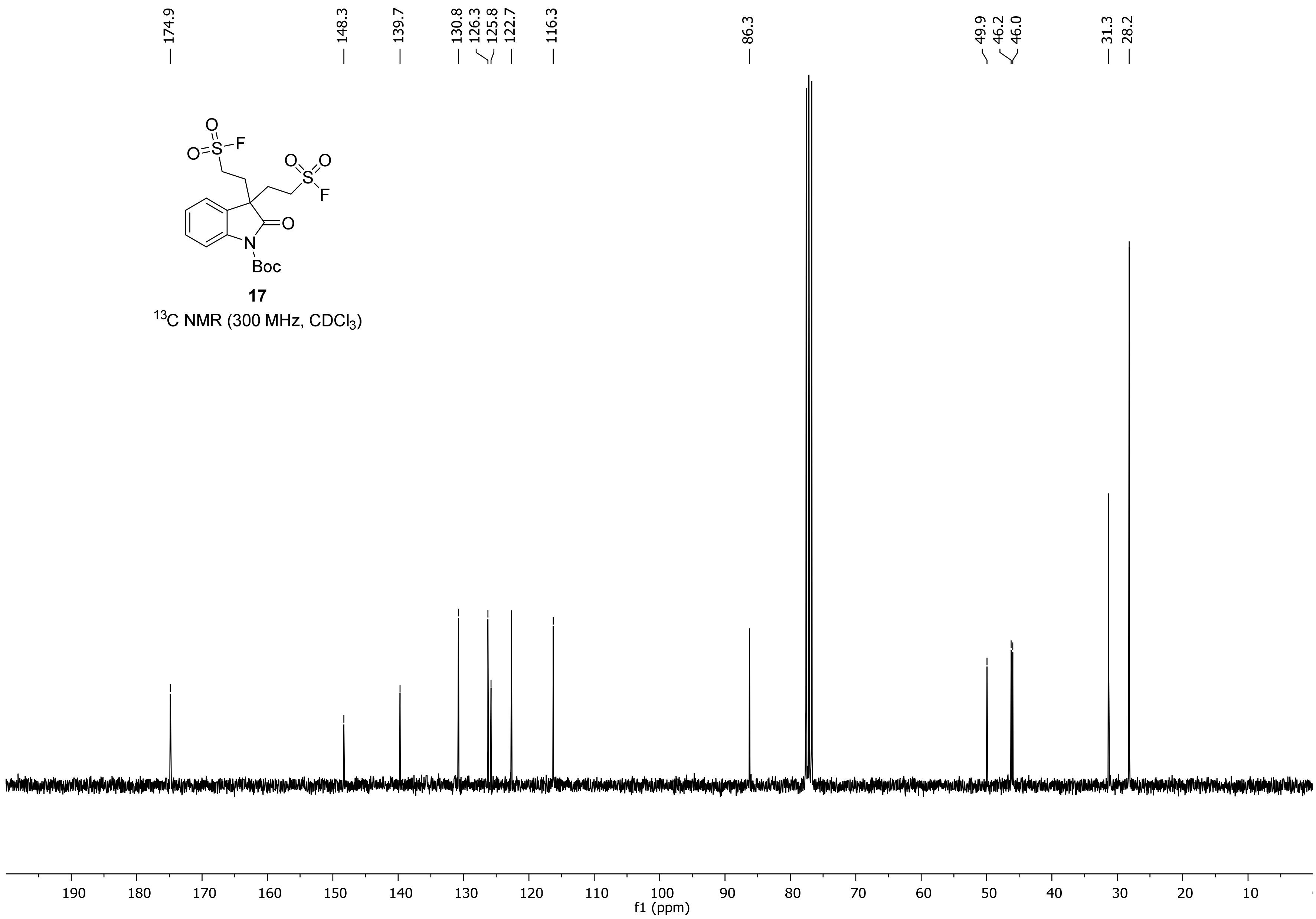
174.9
148.3
139.7
130.8
126.3
125.8
122.7
116.3
86.3
49.9
46.2
46.0
31.3
28.2
O
S
F
O
O
S
O
F
O
N
Boc
17
^{13}C NMR (300 MHz, $CDCl_3$)
190 180 170 160 150 140 130 120 110 100 90 80 70 60 50 40 30 20 10
f1 (ppm)

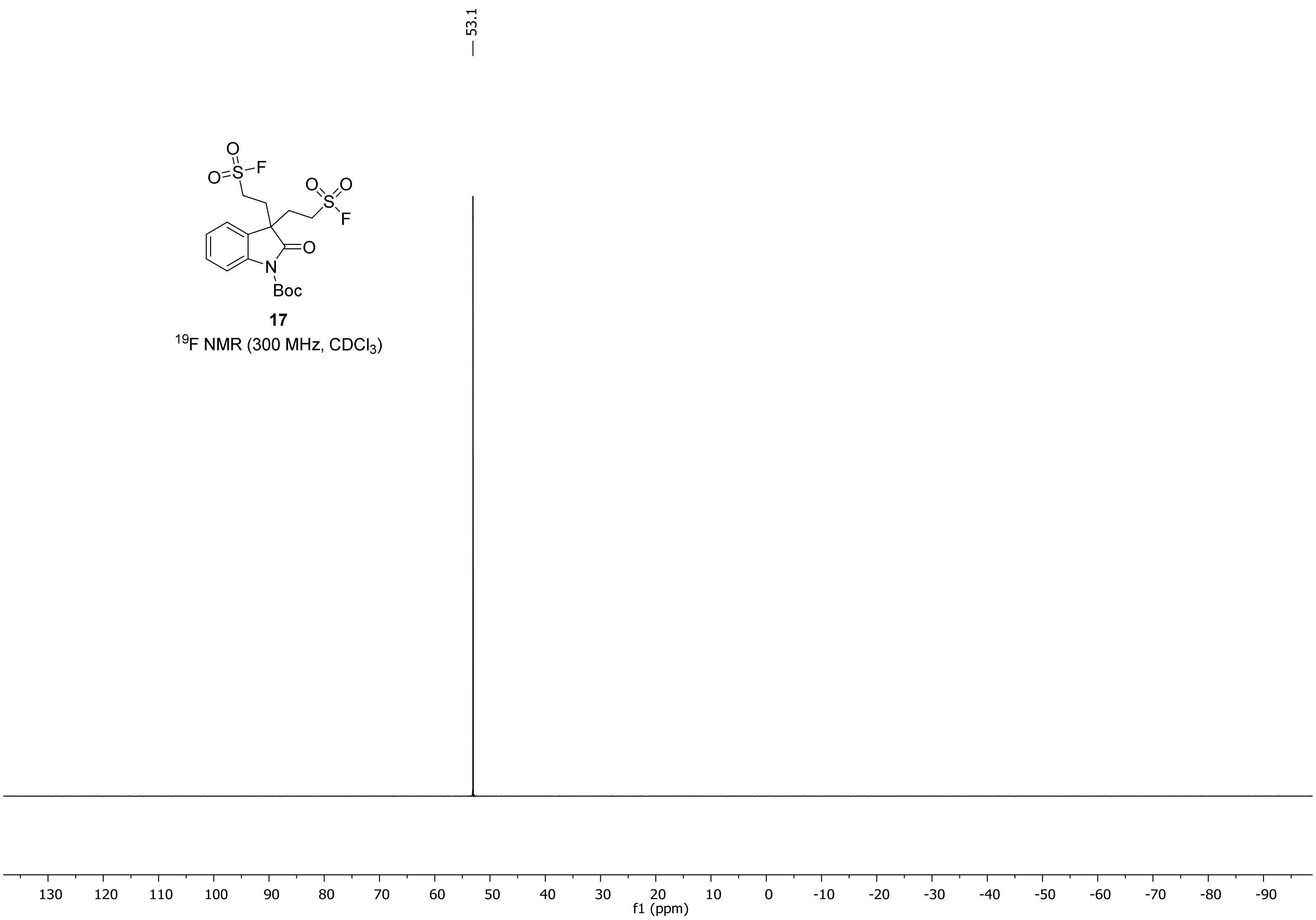

17
^{19}F NMR (300 MHz, $CDCl_3$)
53.1
130 120 110 100 90 80 70 60 50 40 30 20 10 0 -10 -20 -30 -40 -50 -60 -70 -80 -90
f1 (ppm)

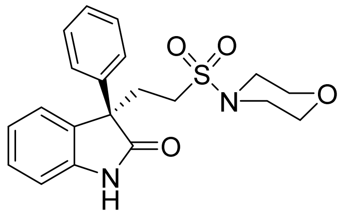

3

^{1}H NMR (300 MHz, $CDCl_3$)

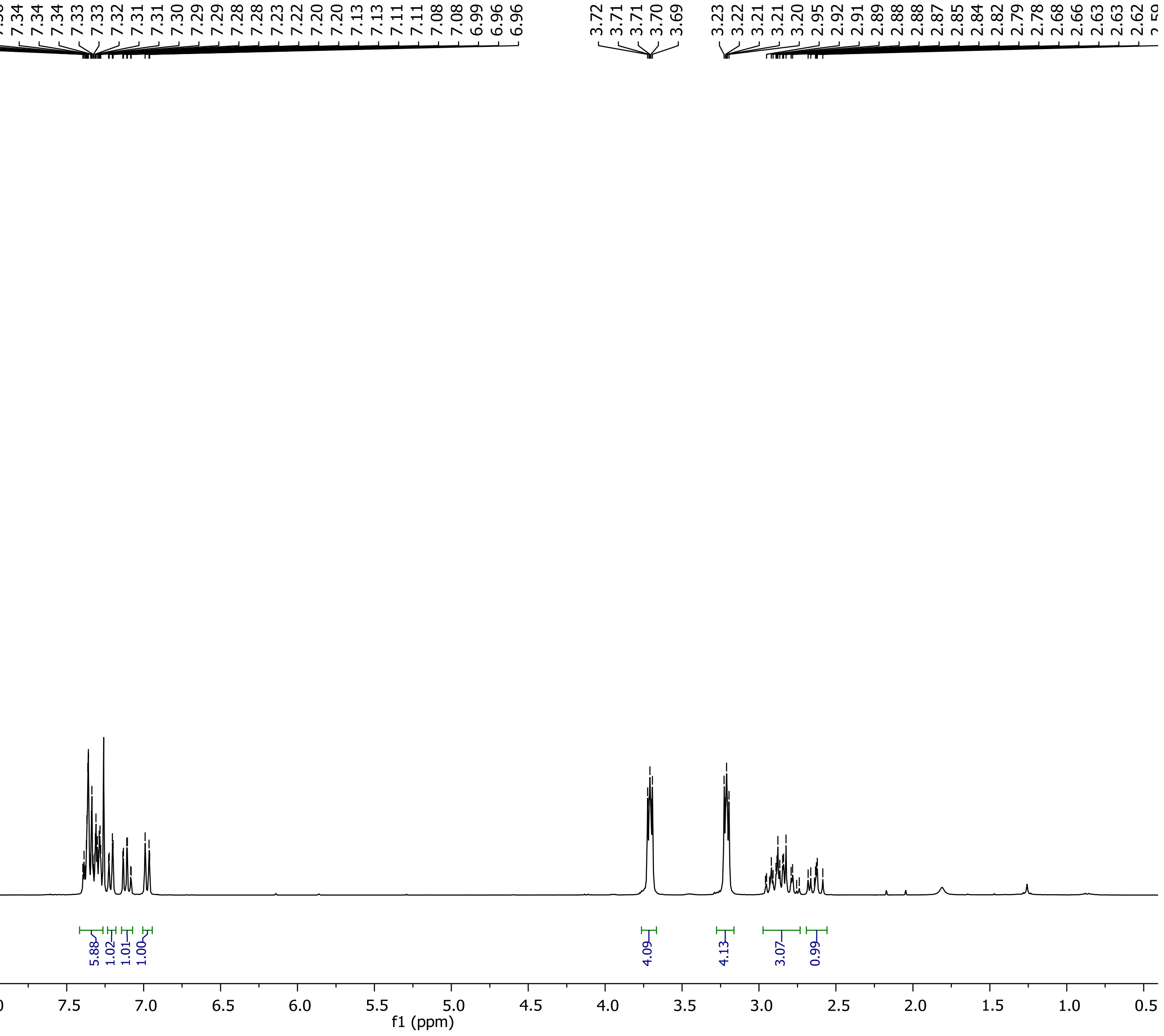

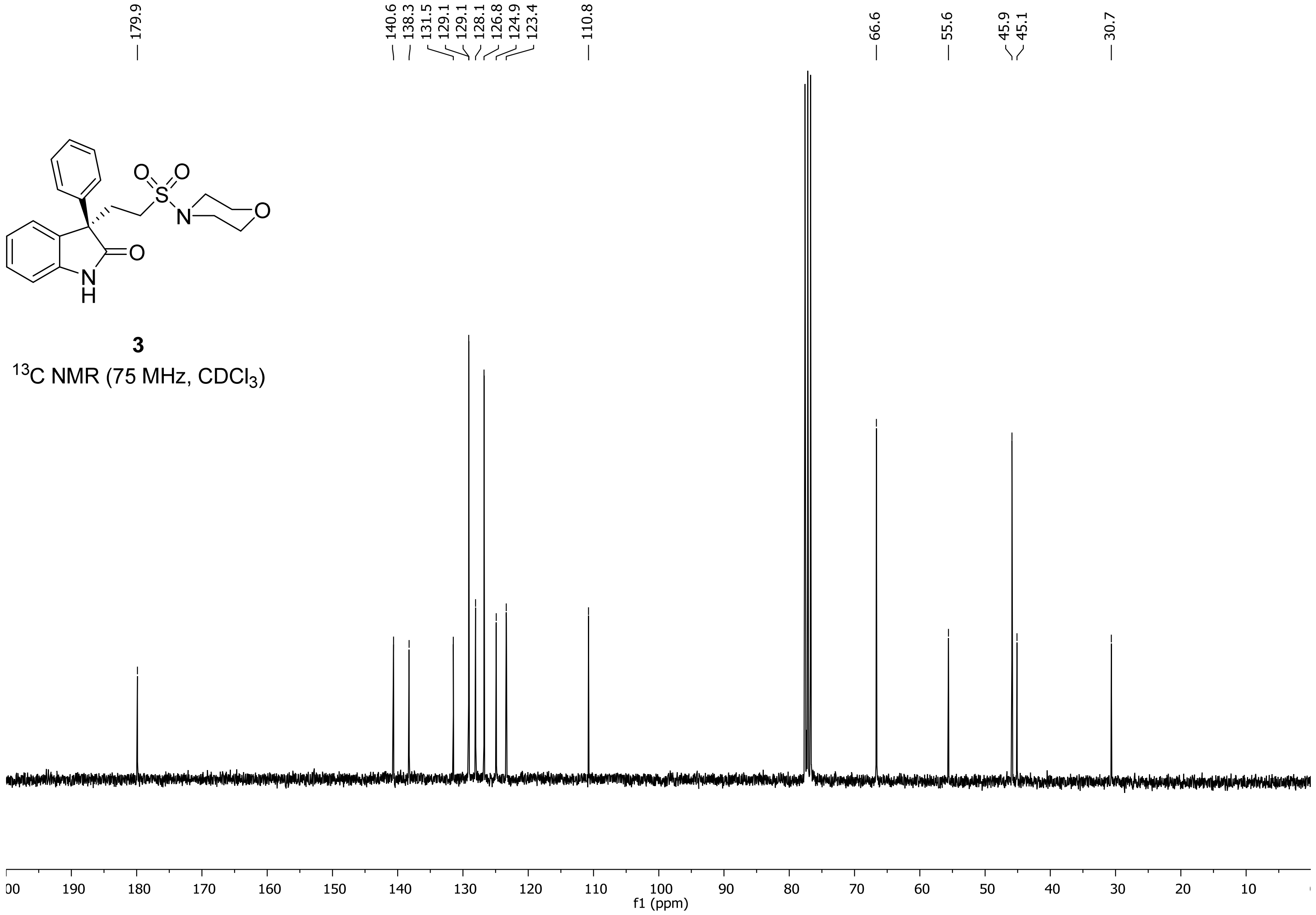

3

^{13}C NMR (75 MHz, $CDCl_3$)

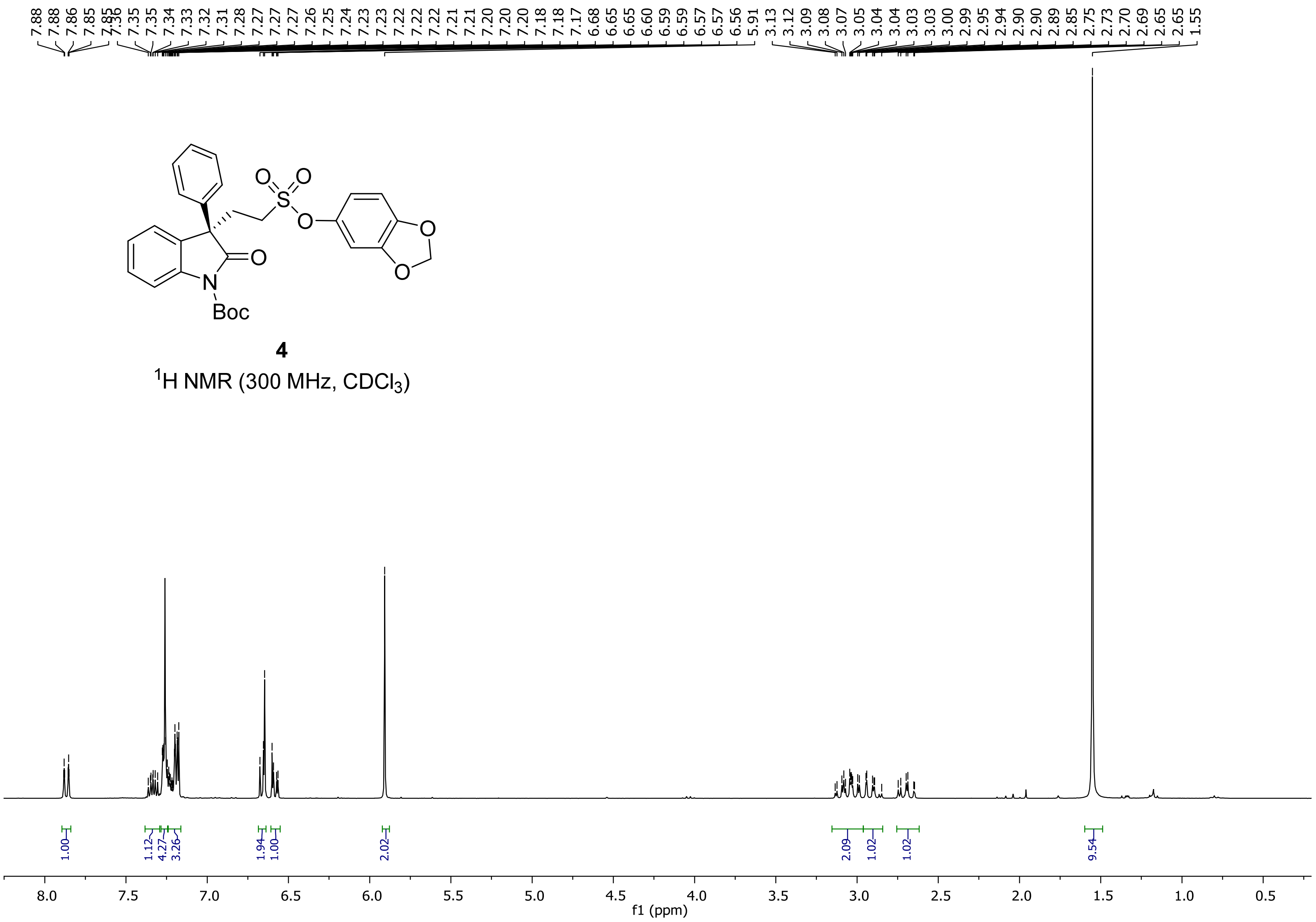

4

^{1}H NMR (300 MHz, $CDCl_3$)

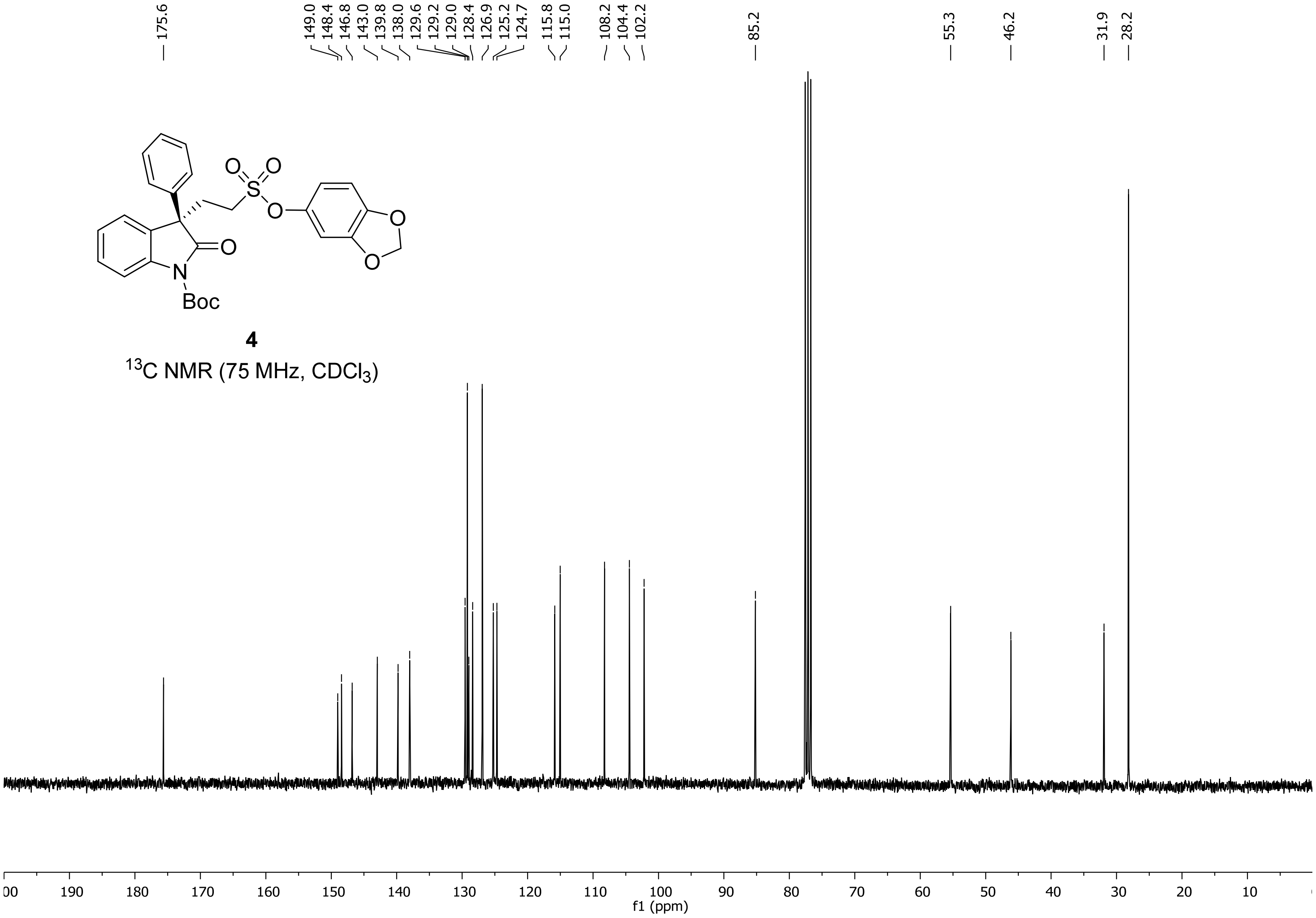
175.6
149.0
148.4
146.8
143.0
139.8
138.0
129.6
129.2
129.0
128.4
126.9
125.2
124.7
115.8
115.0
108.2
104.4
102.2
85.2
55.3
46.2
31.9
28.2
Boc
4
^{13}C NMR (75 MHz, $CDCl_3$)
00
190
180
170
160
150
140
130
120
110
100
90
80
70
60
50
40
30
20
10
f1 (ppm)

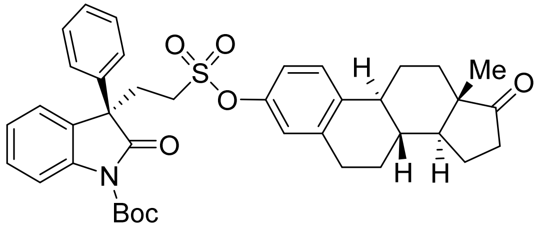

5

^{1}H NMR (500 MHz, $CDCl_3$)

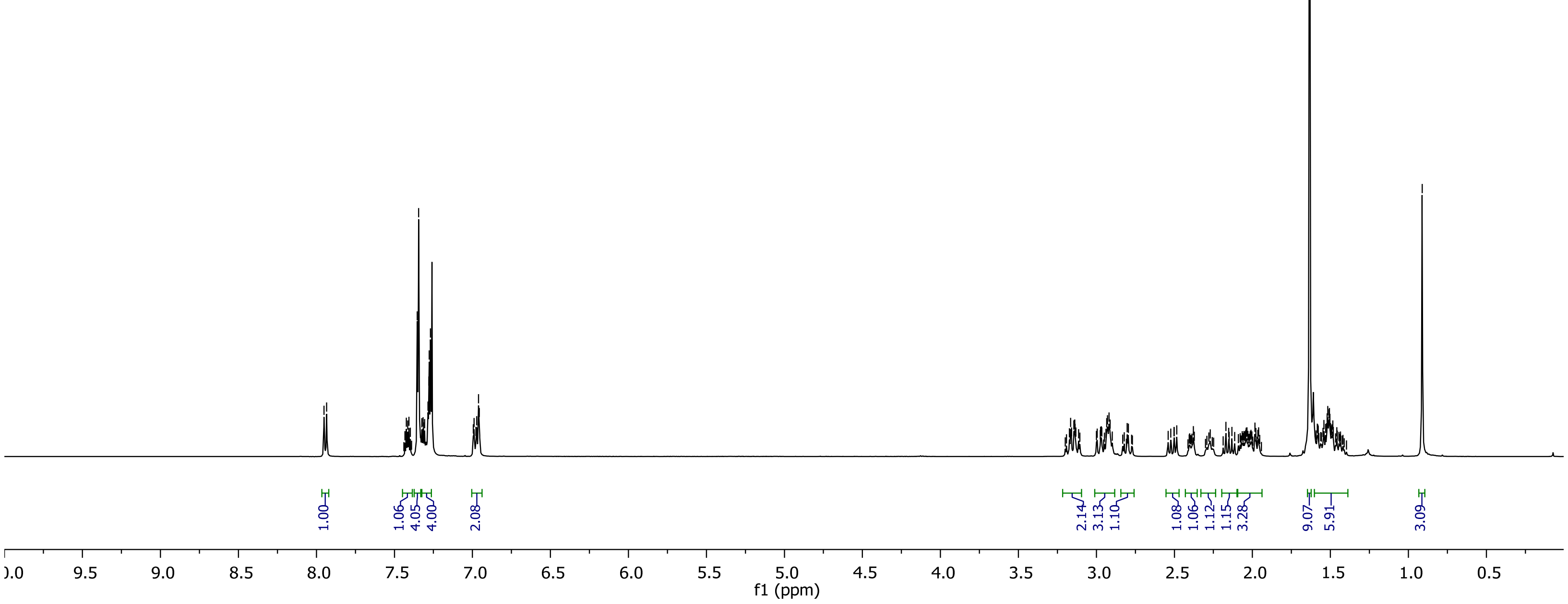

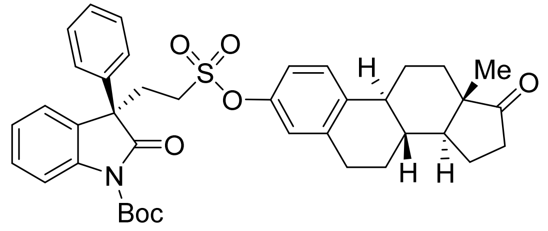

5

^{13}C NMR (75 MHz, $CDCl_3$)

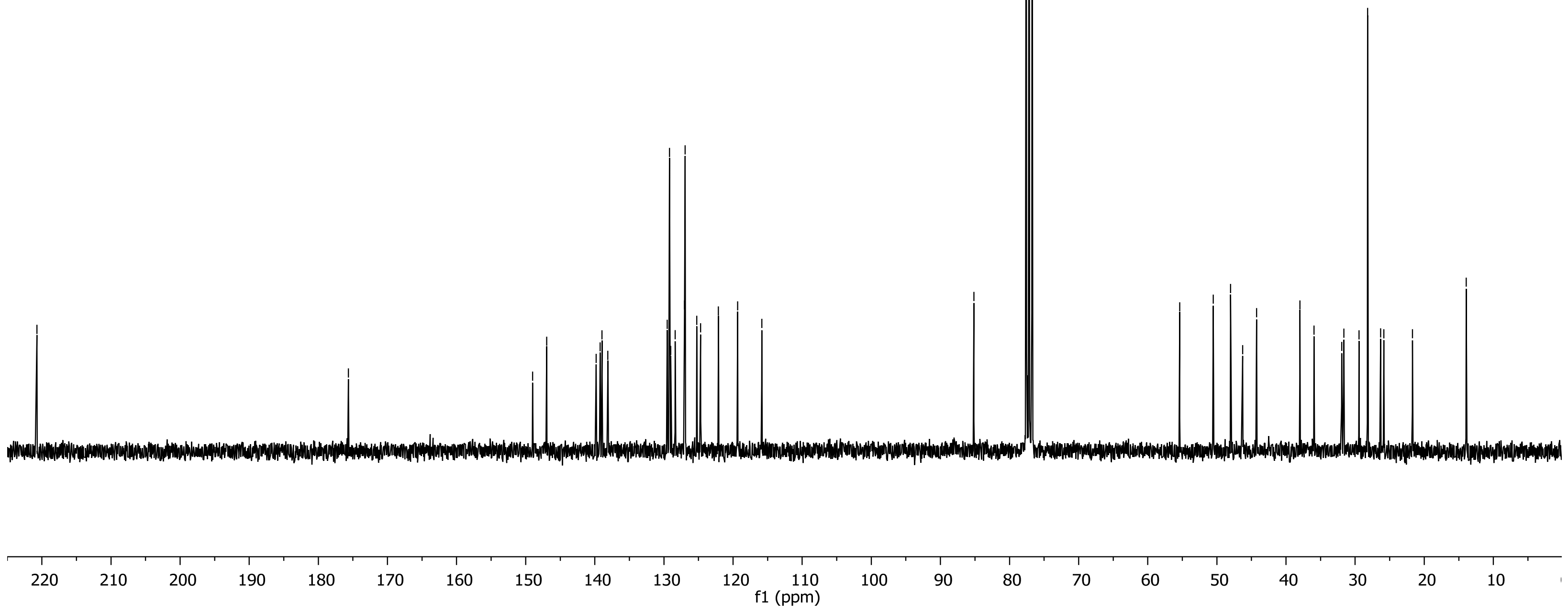

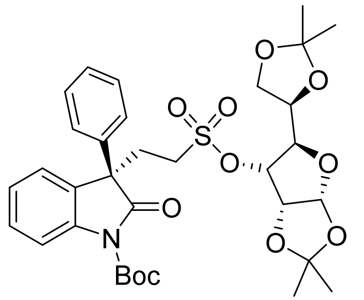

6

^{1}H NMR (300 MHz, $CDCl_3$)

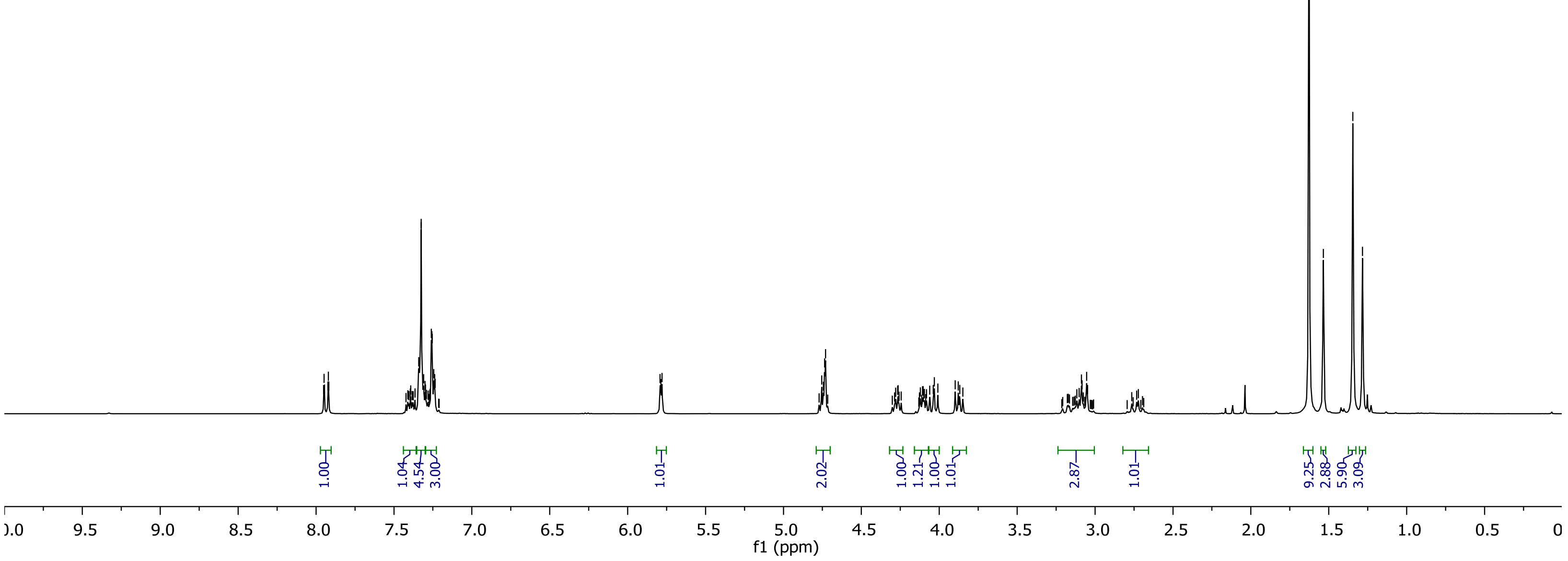

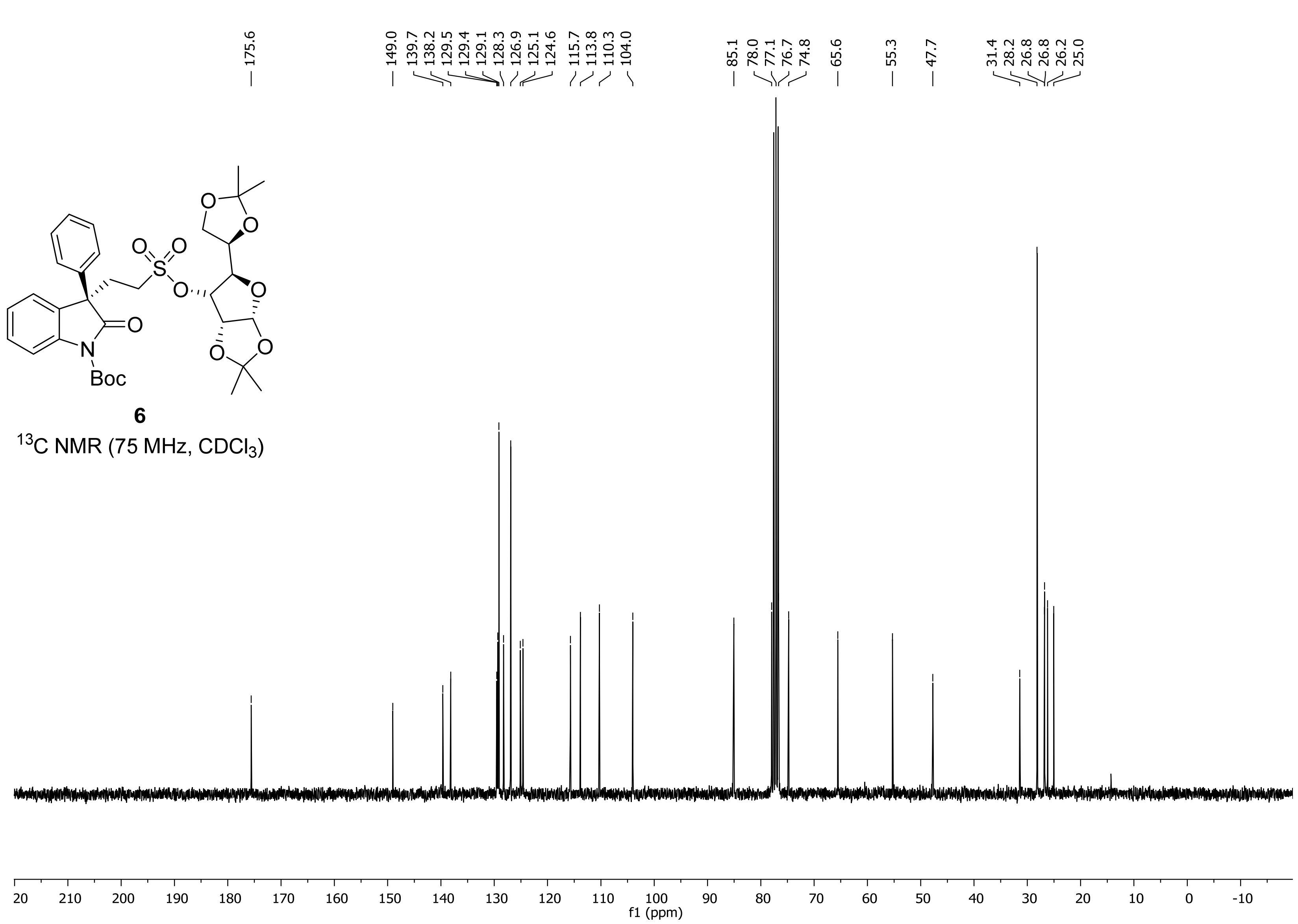

175.6
149.0
139.7
138.2
129.5
129.4
129.1
128.3
126.9
125.1
124.6
115.7
113.8
110.3
104.0
85.1
78.0
77.1
76.7
74.8
65.6
55.3
47.7
31.4
28.2
26.8
26.8
26.2
25.0
Boc
6
^{13}C NMR (75 MHz, $CDCl_3$)
f1 (ppm)

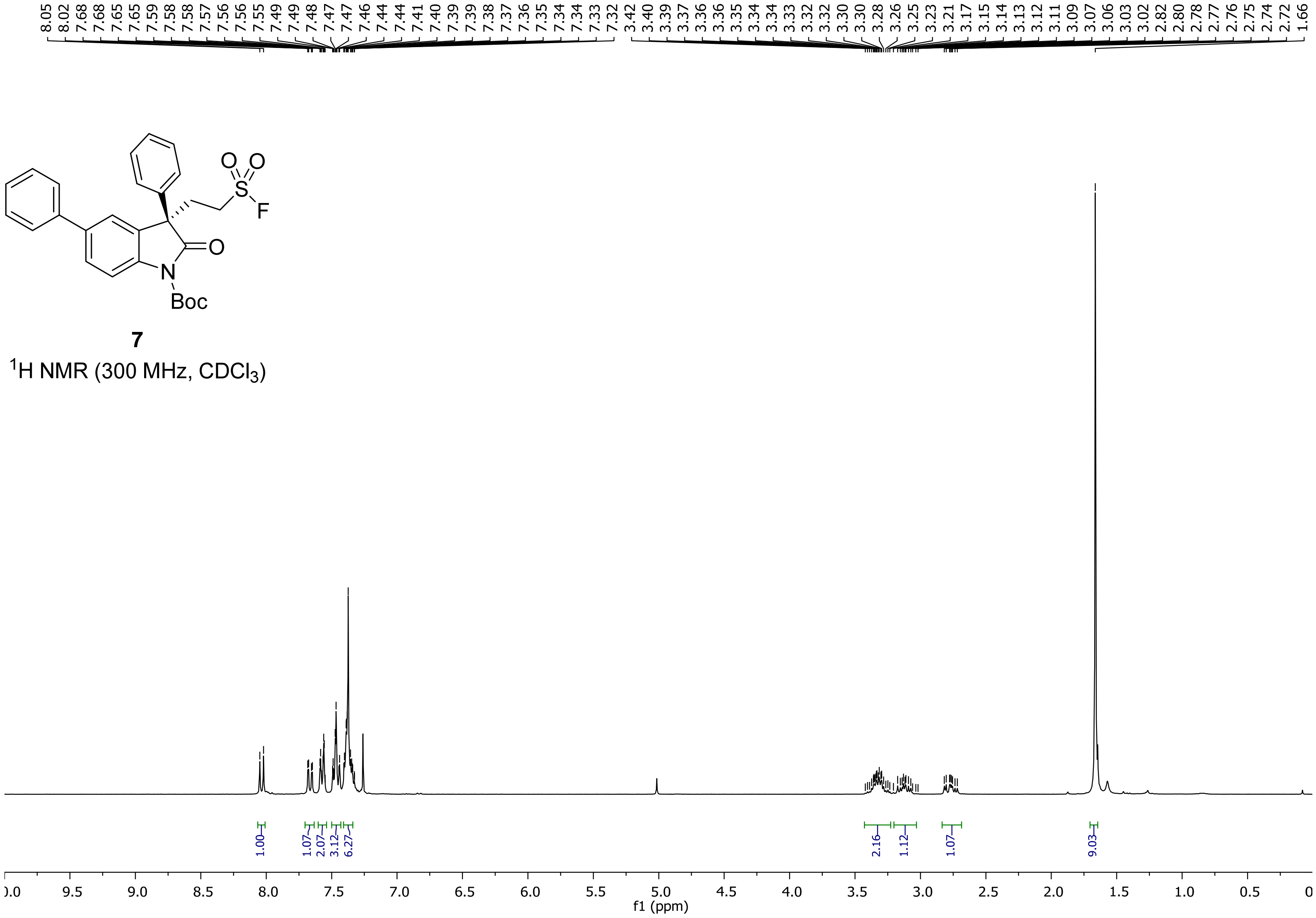

7

^{1}H NMR (300 MHz, $CDCl_3$)

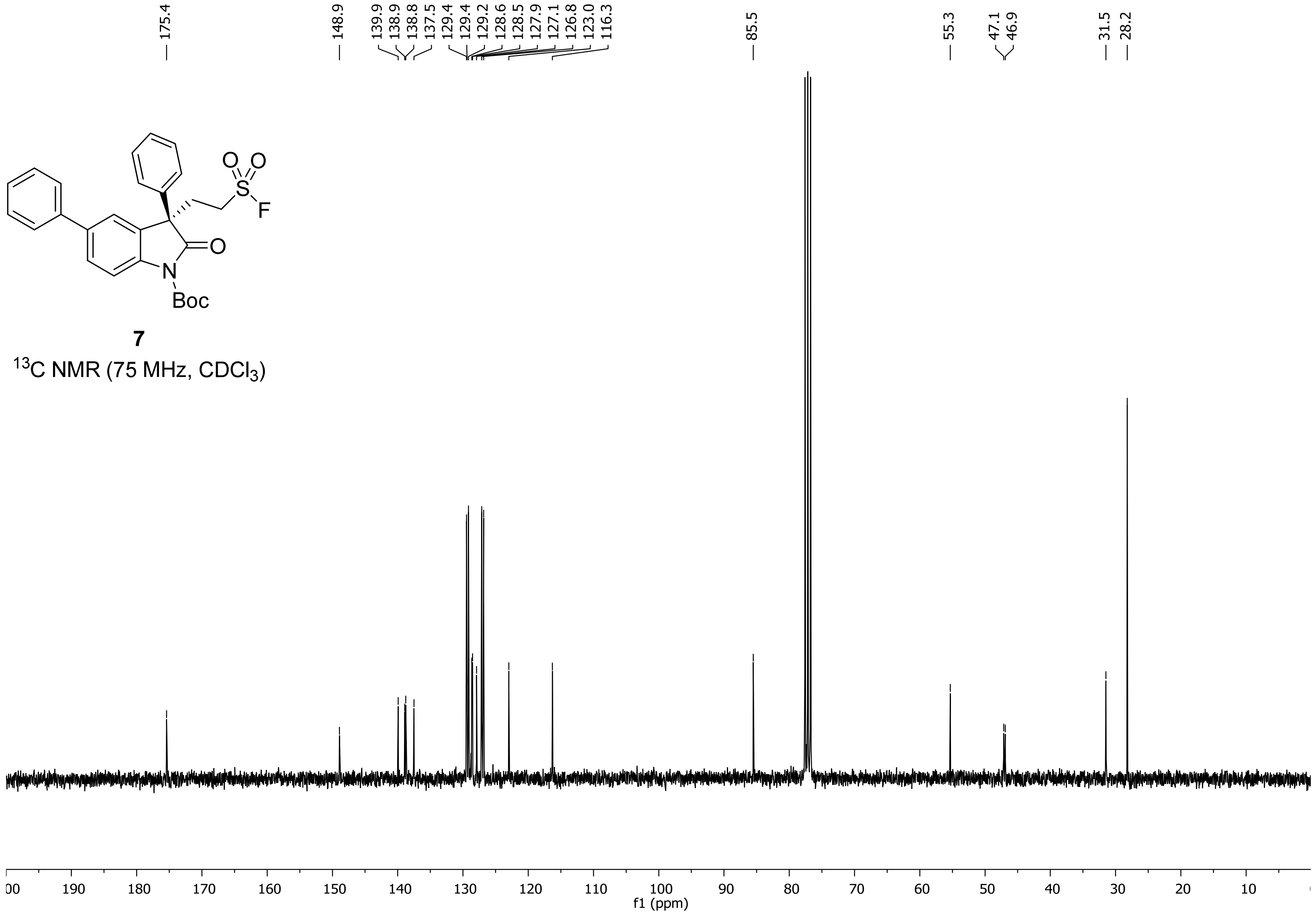

7

^{13}C NMR (75 MHz, $CDCl_3$)

HPLC Analysis

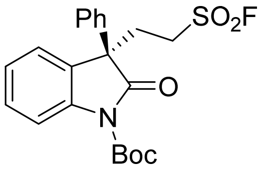

2a

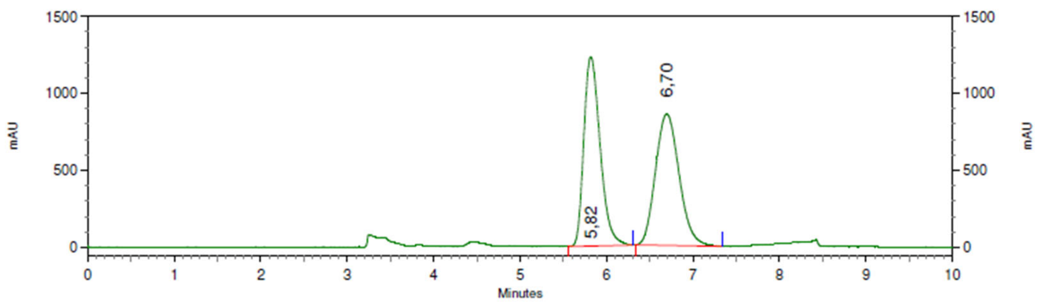

36: 204 nm, 4 nm
Results

Retention Time	Area	Area Percent
5,82	64511933	49,805
6,70	65017370	50,195

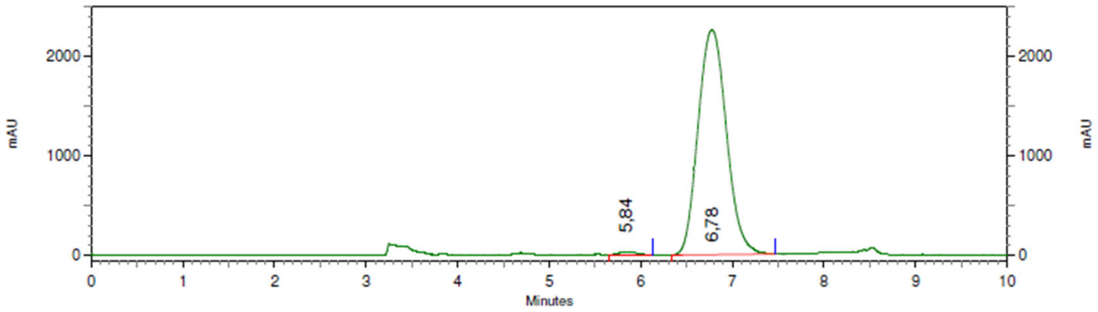

36: 204 nm, 4 nm
Results

Retention Time	Area	Area Percent
5,84	1570629	0,800
6,78	194638484	99,200

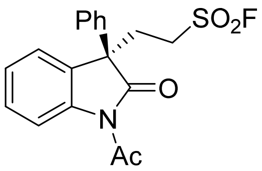

2b

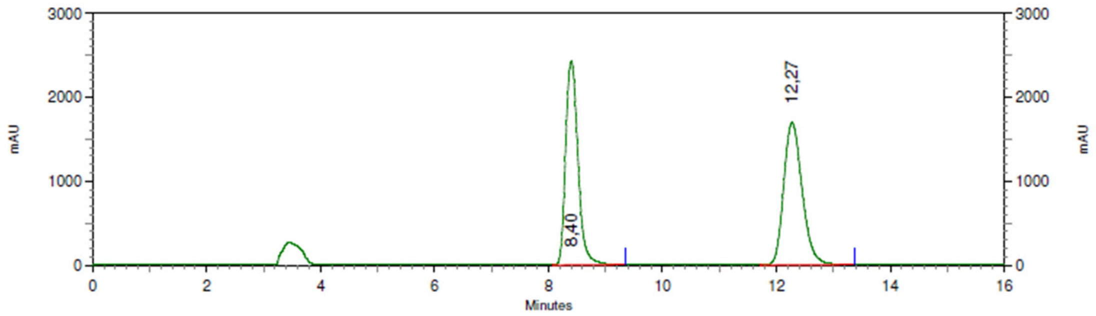

38: 205 nm, 4 nm
Results

Retention Time	Area	Area Percent
8,40	145832574	48,966
12,27	151992758	51,034

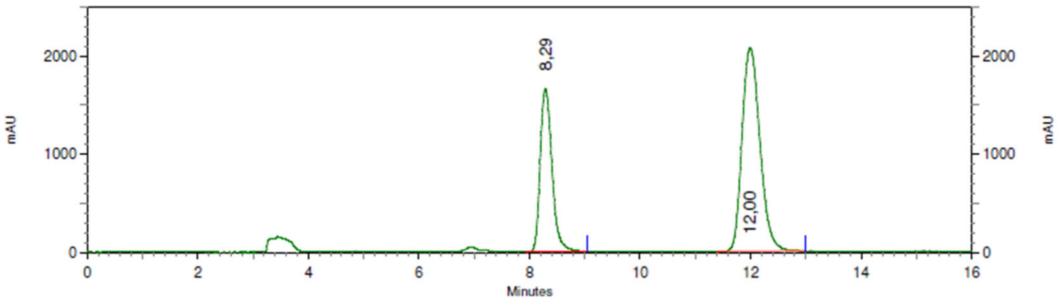

39: 203 nm, 4 nm
Results

Retention Time	Area	Area Percent
8,29	98292989	34,502
12,00	186599655	65,498

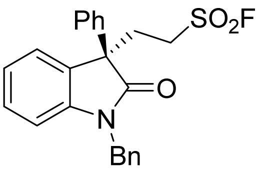

2c

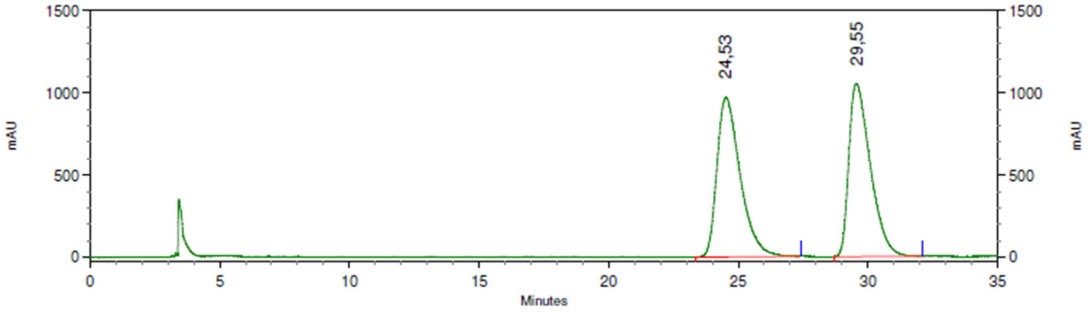

10: 204 nm, 4 nm
Results

Retention Time	Area	Area Percent
24,53	242260649	49,990
29,55	242353943	50,010

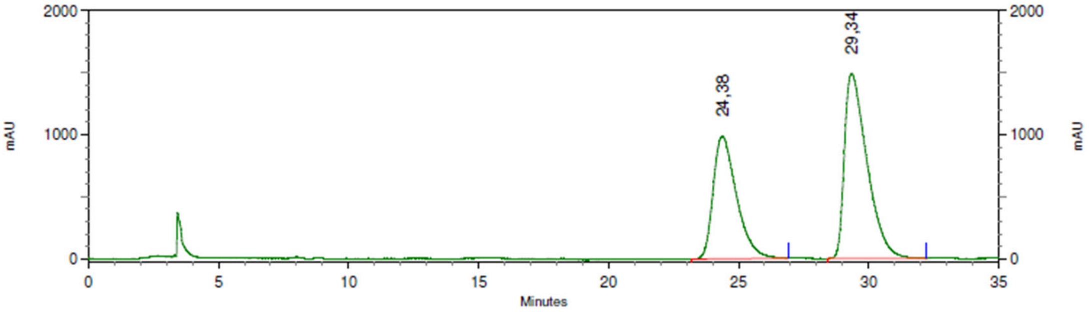

10: 204 nm, 4 nm
Results

Retention Time	Area	Area Percent
24,38	246904343	40,710
29,34	359595038	59,290

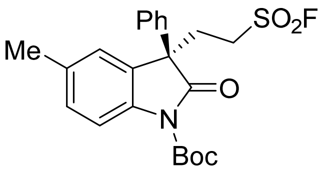

2d

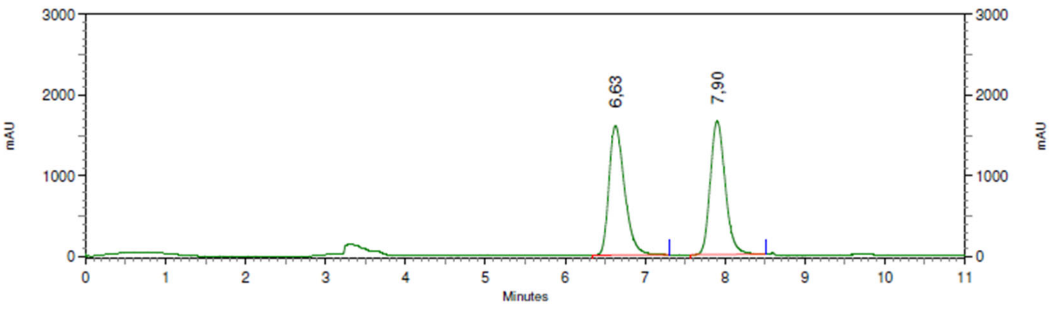

39: 203 nm, 4 nm
Results

Retention Time	Area	Area Percent
6,63	86608274	50,082
7,90	86324707	49,918

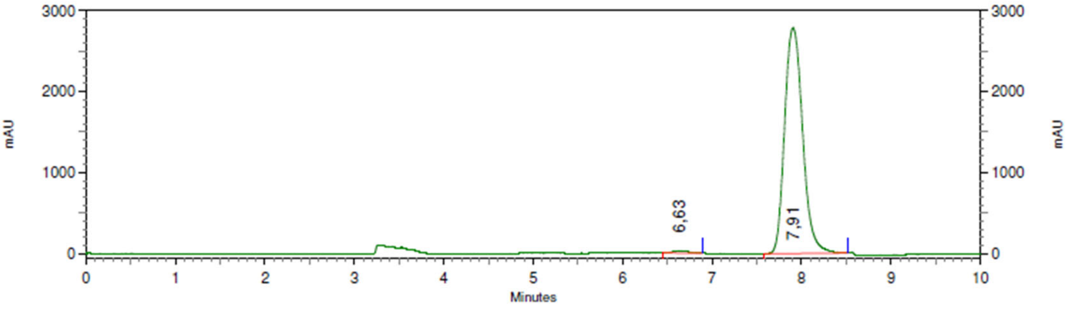

39: 203 nm, 4 nm
Results

Retention Time	Area	Area Percent
6,63	1460795	0,926
7,91	156304399	99,074

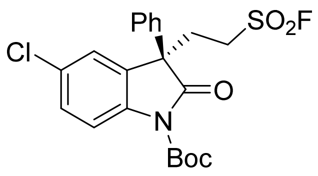

2e

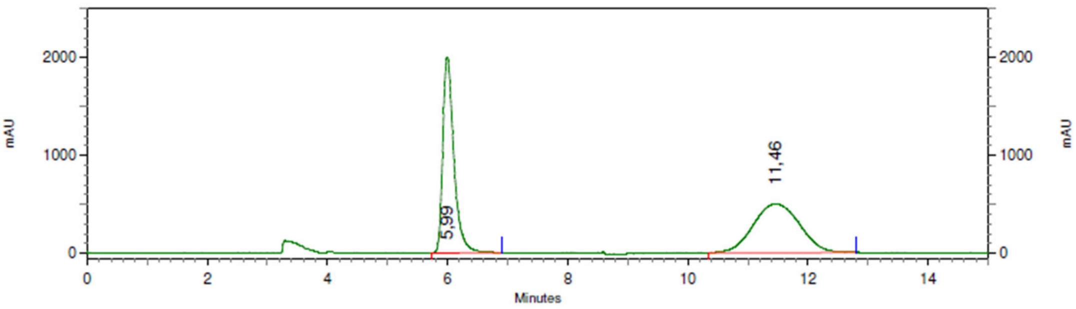

36: 204 nm, 4 nm
Results

Retention Time	Area	Area Percent
5,99	102316165	48,963
11,46	106652154	51,037

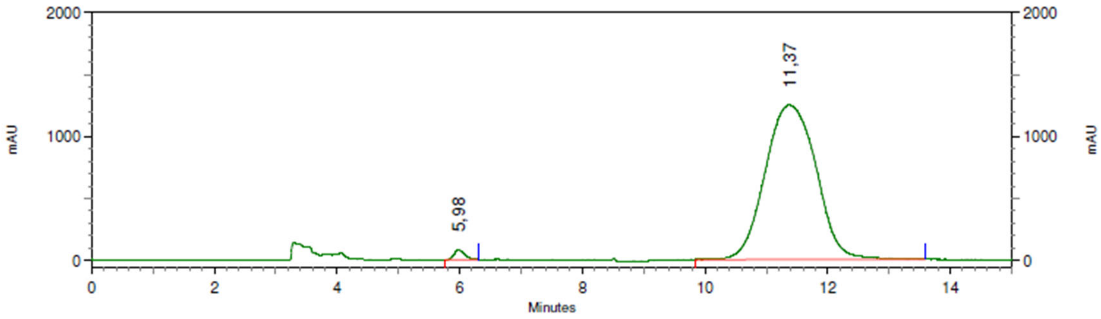

36: 204 nm, 4 nm
Results

Retention Time	Area	Area Percent
5,98	3820351	1,308
11,37	288281245	98,692

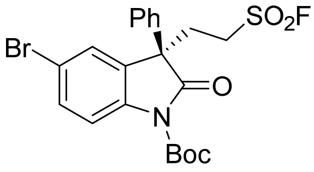

2f

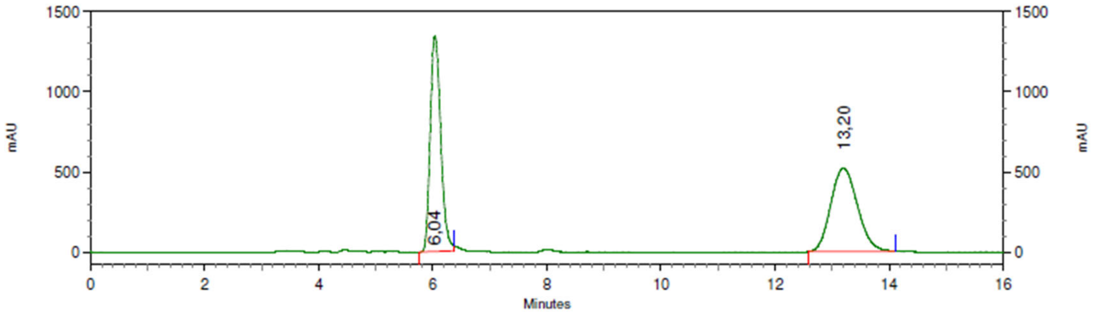

43: 239 nm, 4 nm
Results

Retention Time	Area	Area Percent
6,04	68639261	50,386
13,20	67587977	49,614

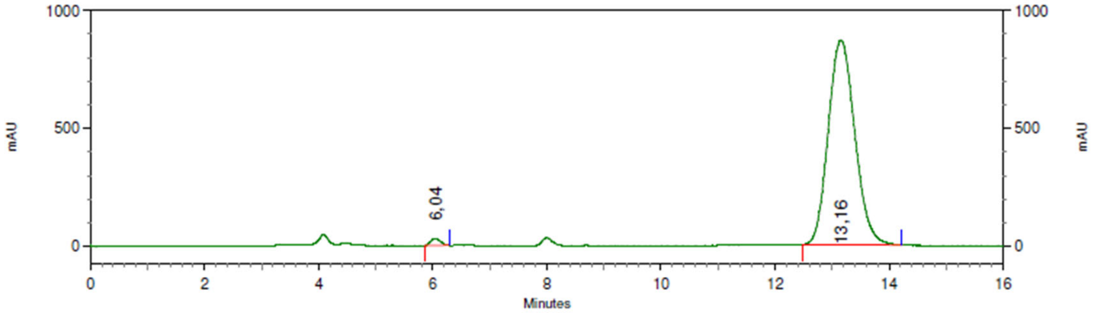

43: 239 nm, 4 nm
Results

Retention Time	Area	Area Percent
6,04	1342262	1,180
13,16	112385075	98,820

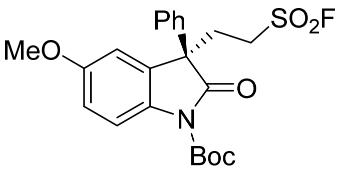

2g

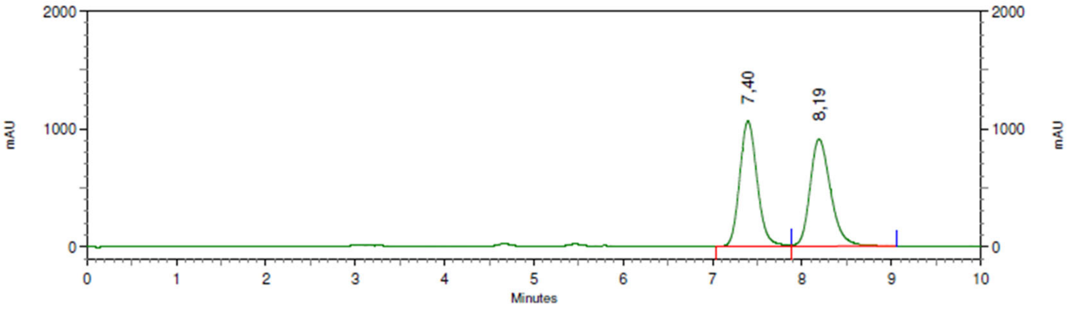

40: 236 nm, 4 nm
Results

Retention Time	Area	Area Percent
7,40	58169923	49,880
8,19	58450494	50,120

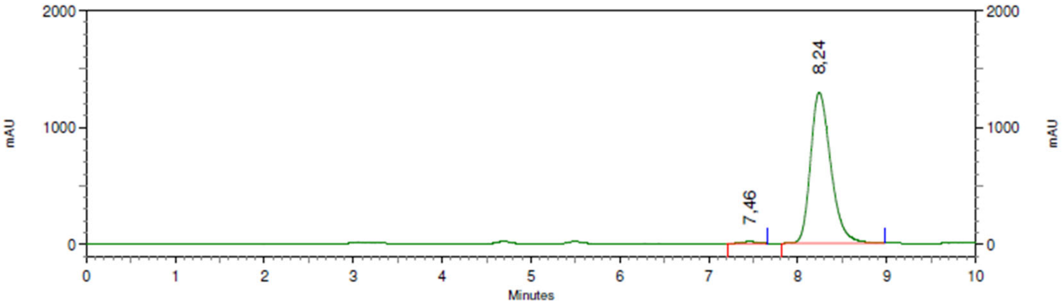

40: 236 nm, 4 nm
Results

Retention Time	Area	Area Percent
7,46	834597	1,010
8,24	81798946	98,990

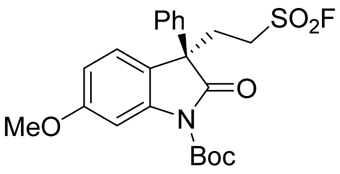

2h

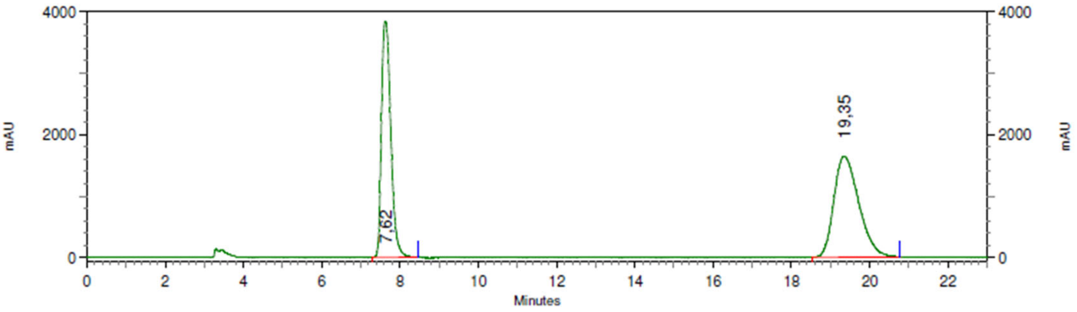

20: 206 nm, 4 nm
Results

Retention Time	Area	Area Percent
7,62	253750523	46,572
19,35	291101802	53,428

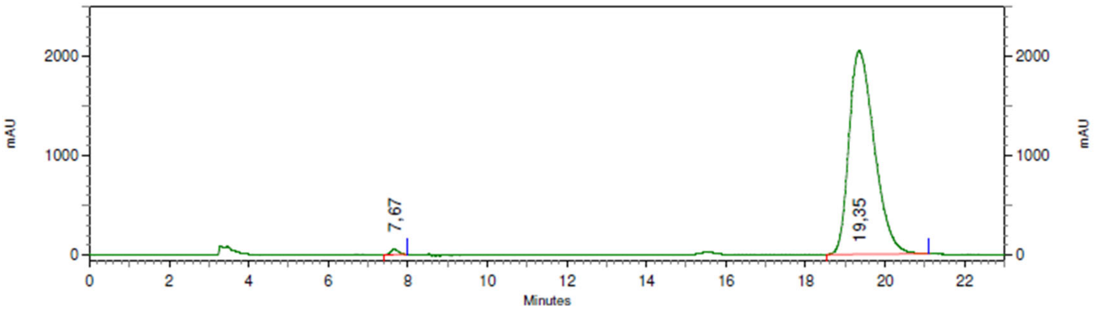

20: 206 nm, 4 nm
Results

Retention Time	Area	Area Percent
7,67	2944382	0,804
19,35	363409529	99,196

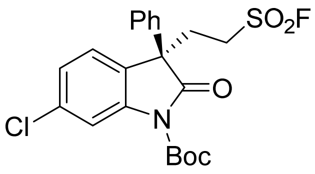

2i

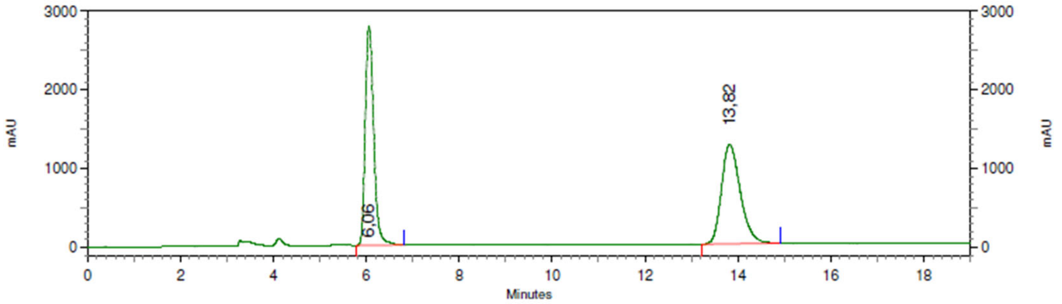

38: 205 nm, 4 nm
Results

Retention Time	Area	Area Percent
6,06	140470362	49,637
13,82	142523463	50,363

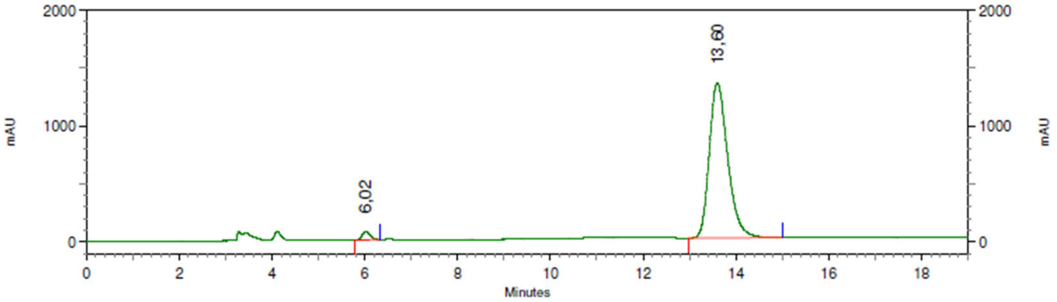

38: 205 nm, 4 nm
Results

Retention Time	Area	Area Percent
6,02	3546563	2,338
13,60	148130753	97,662

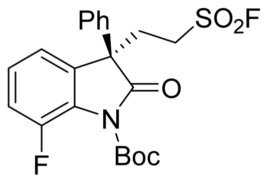

2j

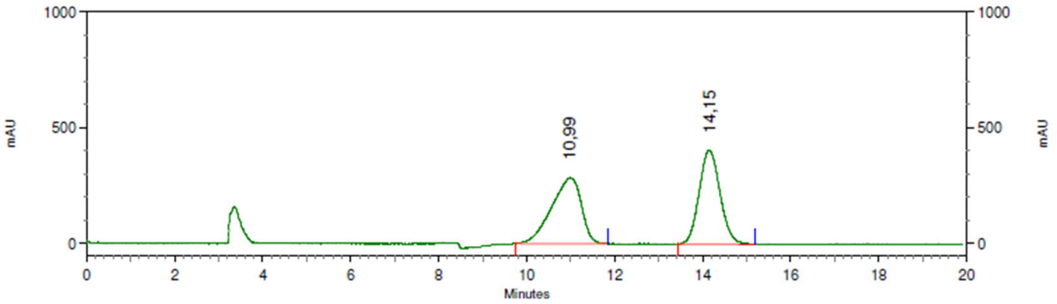

36: 204 nm, 4 nm
Results

Retention Time	Area	Area Percent
10,99	53374376	49,842
14,15	53712385	50,158

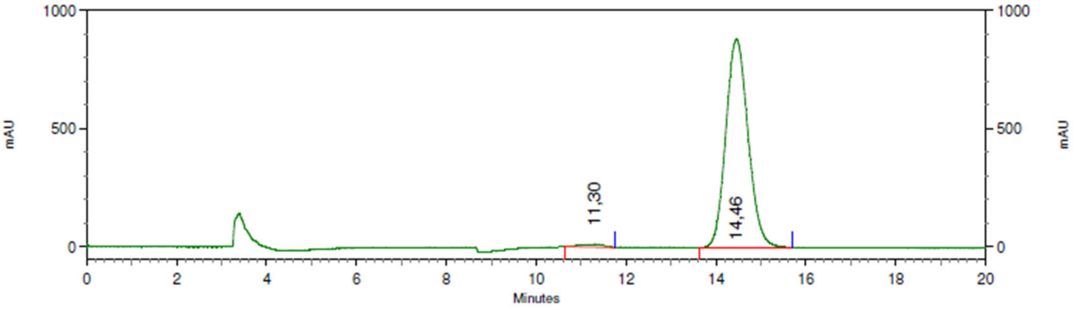

36: 204 nm, 4 nm
Results

Retention Time	Area	Area Percent
11,30	1671049	1,353
14,46	121828033	98,647

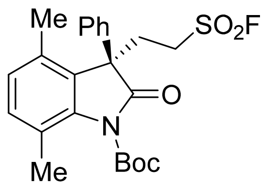

2k

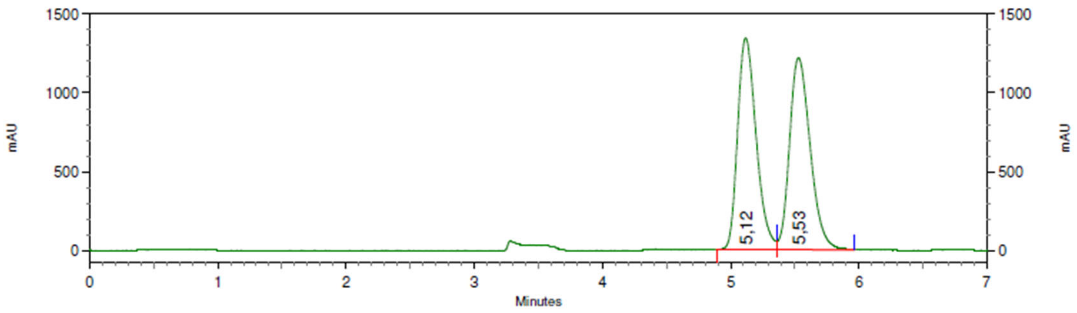

20: 206 nm, 4 nm
Results

Retention Time	Area	Area Percent
5,12	53902789	49,665
5,53	54629502	50,335

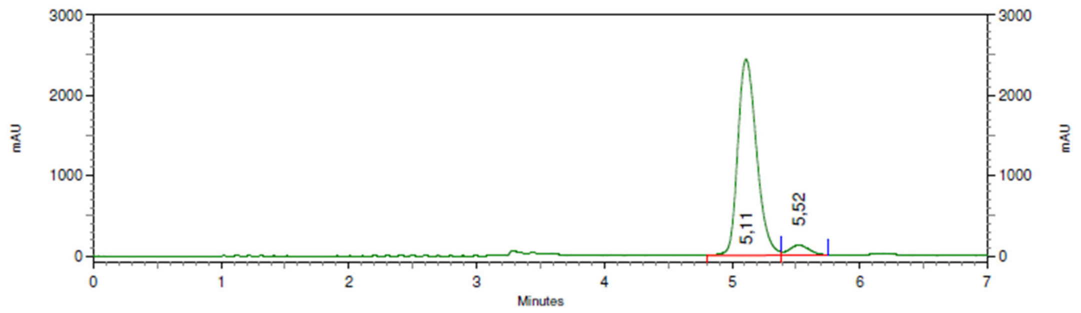

20: 206 nm, 4 nm
Results

Retention Time	Area	Area Percent
5,11	99853565	94,846
5,52	5426546	5,154

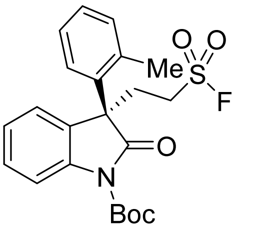

2l

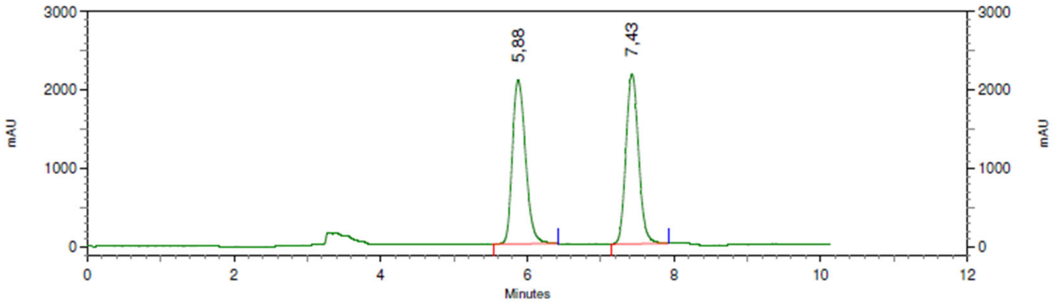

36: 204 nm, 4 nm
Results

Retention Time	Area	Area Percent
5,88	104160363	50,129
7,43	103626285	49,871

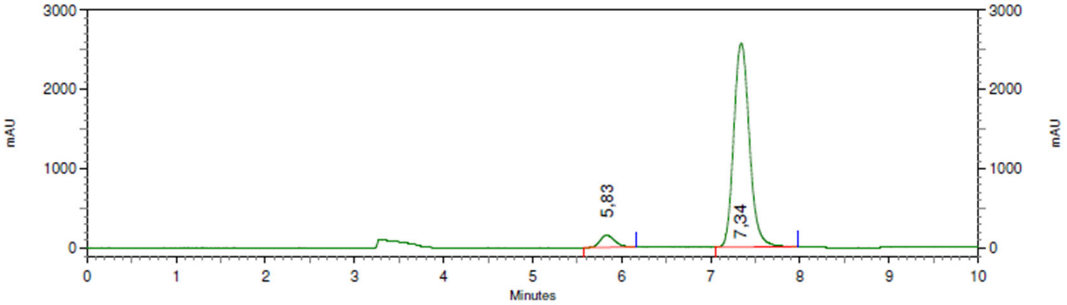

36: 204 nm, 4 nm
Results

Retention Time	Area	Area Percent
5,83	7111793	5,370
7,34	125332734	94,630

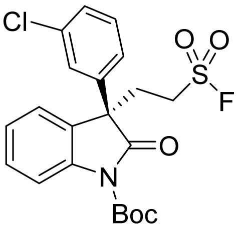

2m

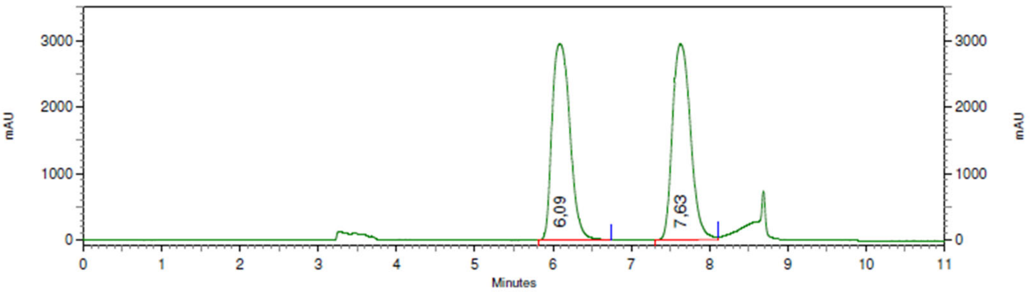

39: 203 nm, 4 nm
Results

Retention Time	Area	Area Percent
6,09	187112733	49,773
7,63	188822920	50,227

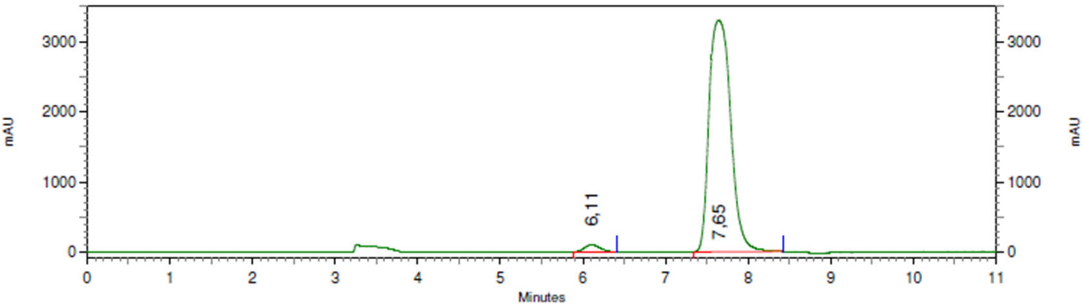

39: 203 nm, 4 nm
Results

Retention Time	Area	Area Percent
6,11	5245209	2,144
7,65	239426754	97,856

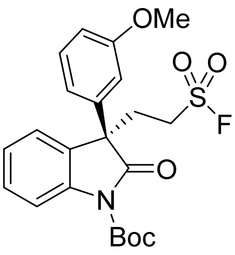

2n

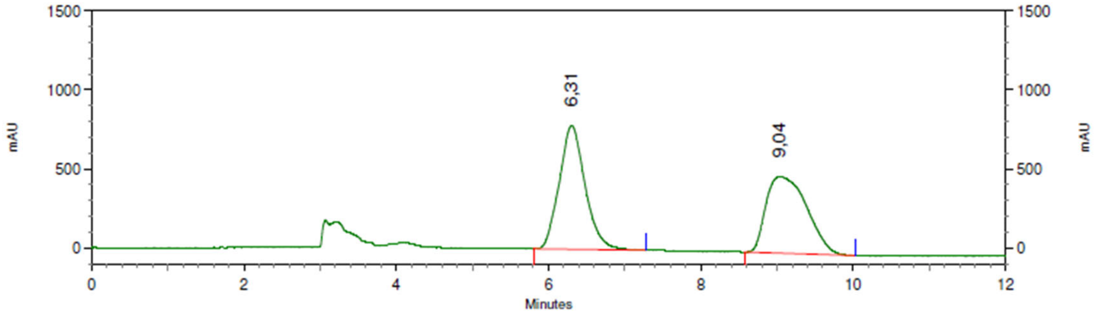

36: 204 nm, 4 nm
Results

Retention Time	Area	Area Percent
6,31	73888568	50,590
9,04	72165294	49,410

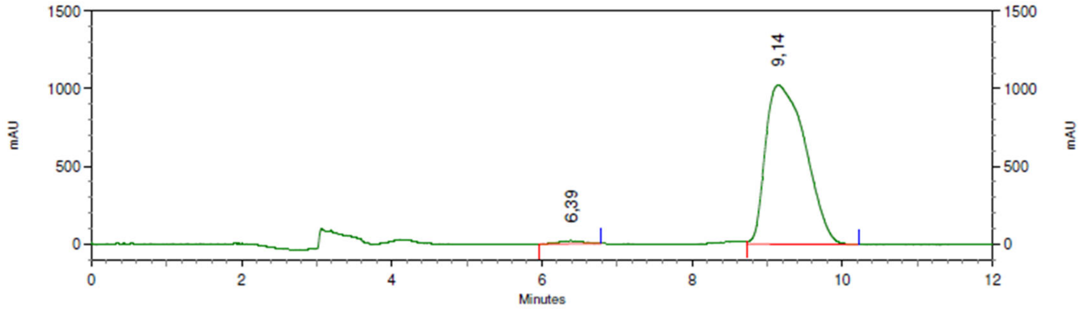

39: 203 nm, 4 nm
Results

Retention Time	Area	Area Percent
6,39	1836665	1,193
9,14	152061930	98,807

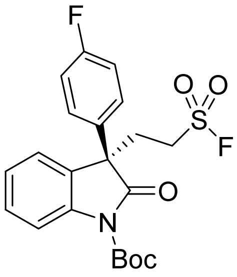

2o

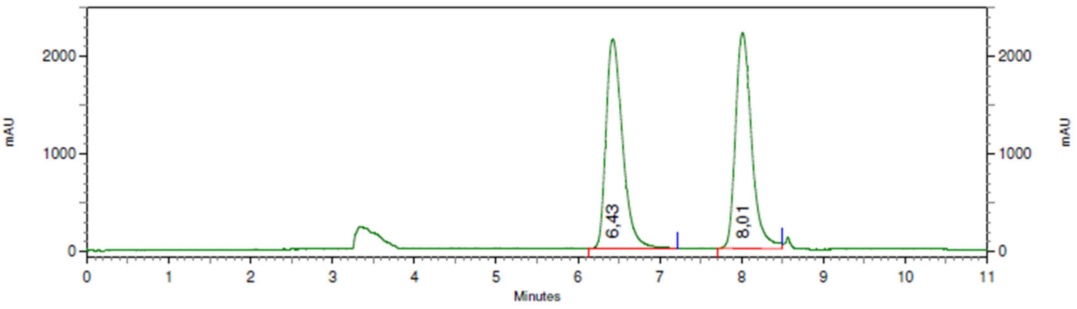

39: 203 nm, 4 nm
Results

Retention Time	Area	Area Percent
6,43	120069938	49,803
8,01	121021781	50,197

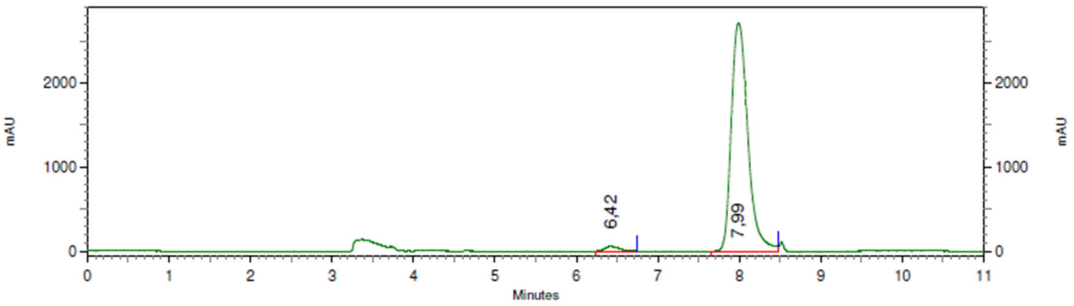

39: 203 nm, 4 nm
Results

Retention Time	Area	Area Percent
6,42	2876391	1,841
7,99	153341165	98,159

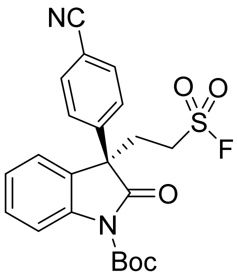

2p

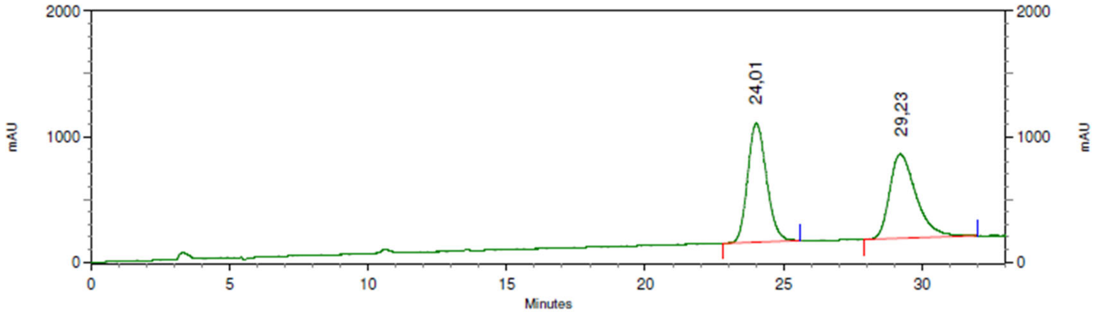

46: 201 nm, 4 nm
Results

Retention Time	Area	Area Percent
24,01	179917176	50,334
29,23	177532951	49,666

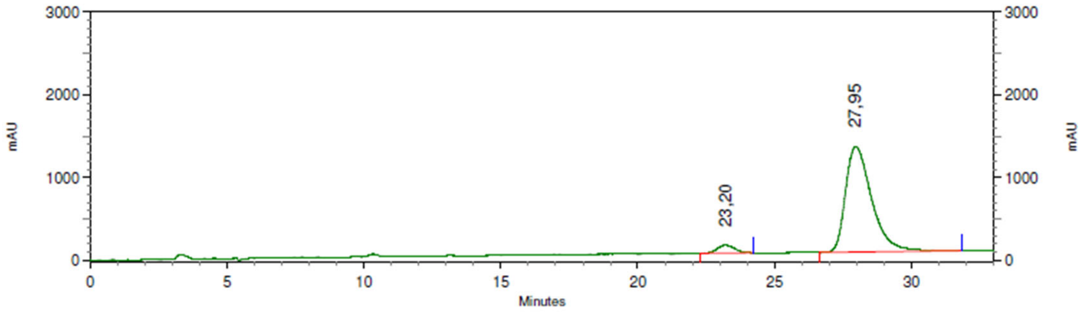

46: 201 nm, 4 nm
Results

Retention Time	Area	Area Percent
23,20	17086906	4,803
27,95	338637138	95,197

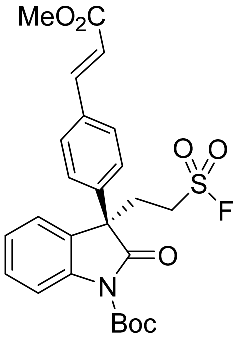

2q

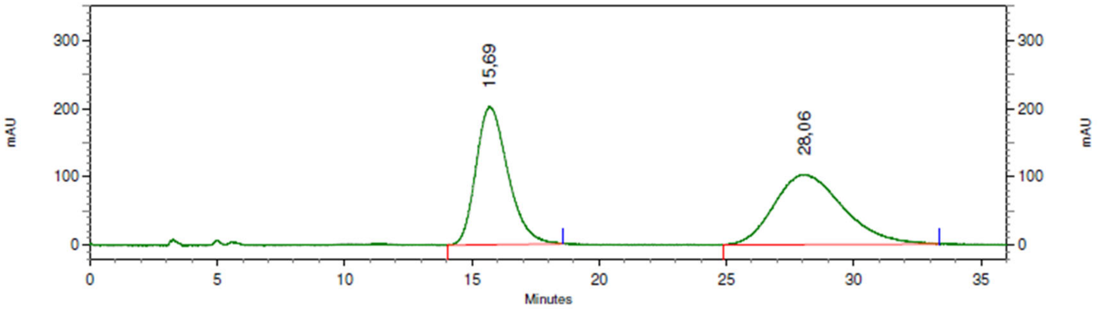

25: 280 nm, 4 nm
Results

Retention Time	Area	Area Percent
15,69	70509519	47,667
28,06	77412953	52,333

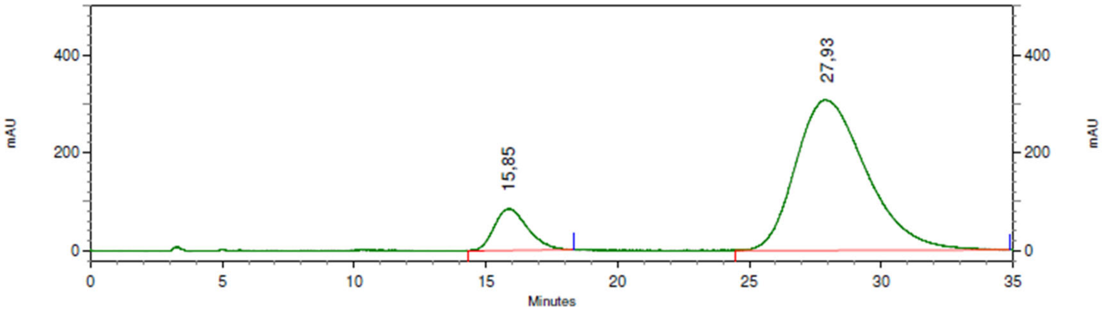

25: 280 nm, 4 nm
Results

Retention Time	Area	Area Percent
15,85	30226146	11,605
27,93	230236671	88,395

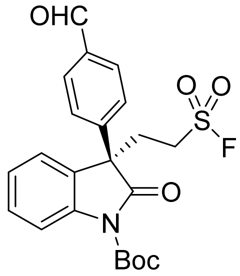

2r

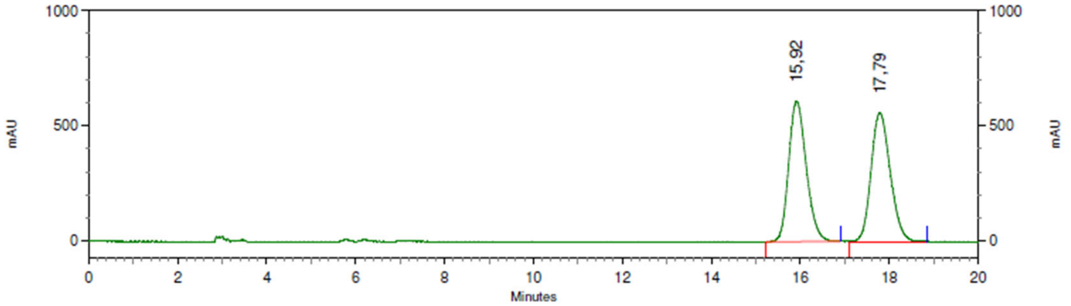

35: 246 nm, 4 nm
Results

Retention Time	Area	Area Percent
15,92	67062075	49,998
17,79	67068332	50,002

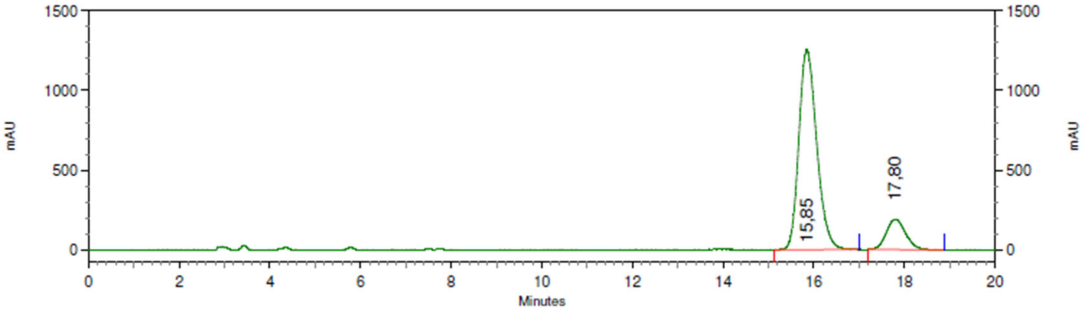

35: 246 nm, 4 nm
Results

Retention Time	Area	Area Percent
15,85	137145874	85,840
17,80	22623292	14,160

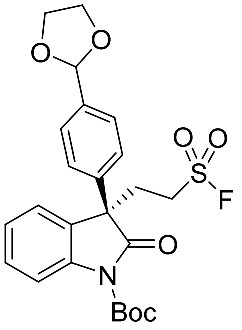

2s

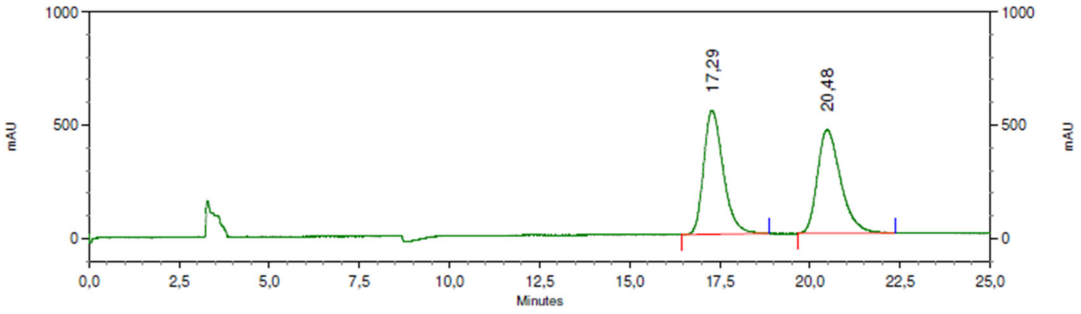

13: 202 nm, 4 nm
Results

Retention Time	Area	Area Percent
17,29	82648869	49,962
20,48	82773653	50,038

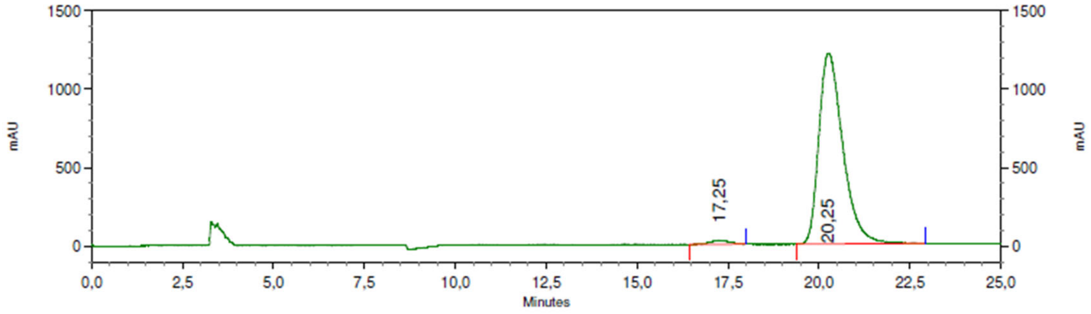

13: 202 nm, 4 nm
Results

Retention Time	Area	Area Percent
17,25	3611751	1,592
20,25	223310506	98,408

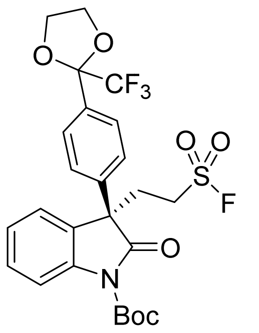

2t

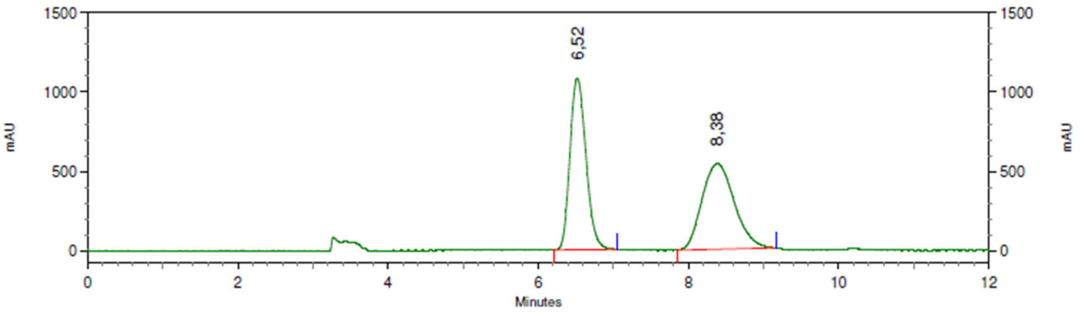

39: 203 nm, 4 nm
Results

Retention Time	Area	Area Percent
6,52	64156098	49,863
8,38	64509683	50,137

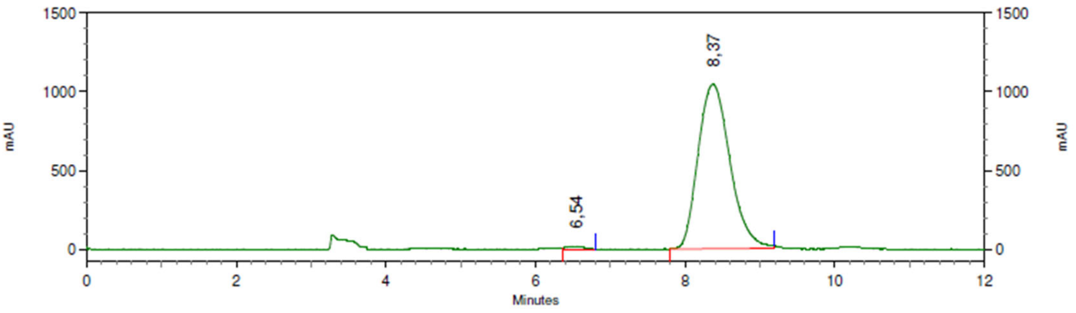

39: 203 nm, 4 nm
Results

Retention Time	Area	Area Percent
6,54	1158569	0,929
8,37	123548779	99,071

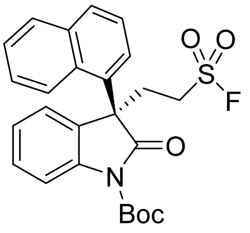

2u

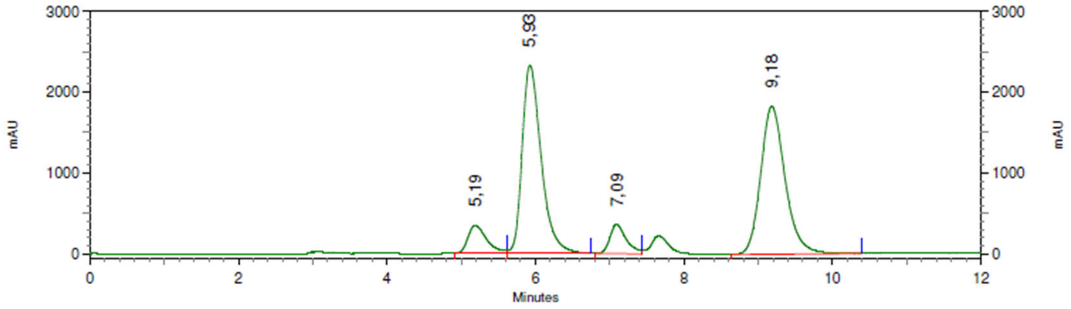

20: 223 nm, 4 nm
Results

Retention Time	Area	Area Percent
5,19	24550147	6,463
5,93	165971820	43,695
7,09	22077704	5,812
9,18	167241215	44,029

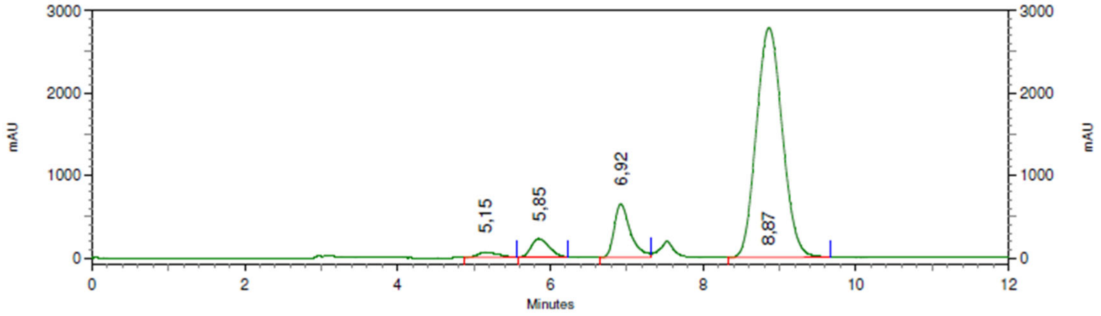

10: 223 nm, 4 nm
Results

Retention Time	Area	Area Percent
5,15	4595530	1,431
5,85	14476946	4,509
6,92	38394841	11,958
8,87	263600469	82,101

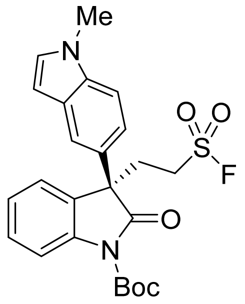

2v

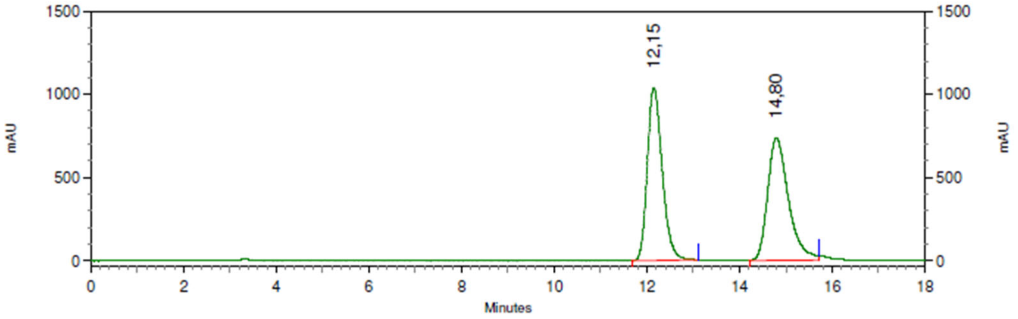

46: 228 nm, 4 nm
Results

Retention Time	Area	Area Percent
12,15	92768960	50,320
14,80	91590036	49,680

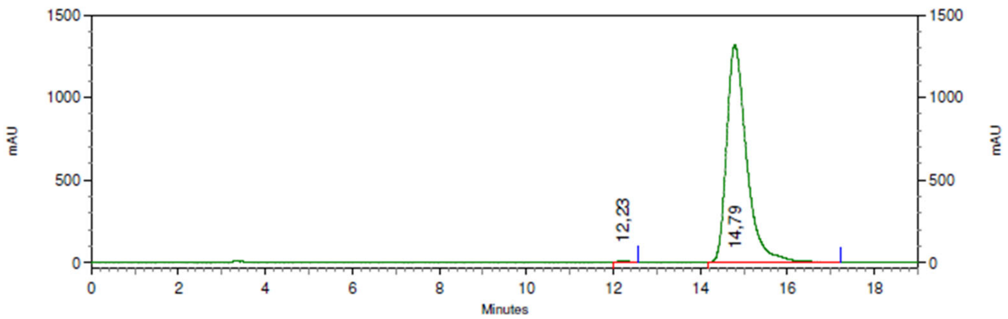

46: 228 nm, 4 nm
Results

Retention Time	Area	Area Percent
12,23	613221	0,362
14,79	168690603	99,638

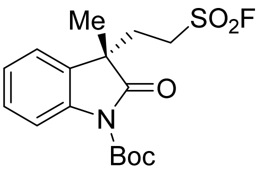

2w

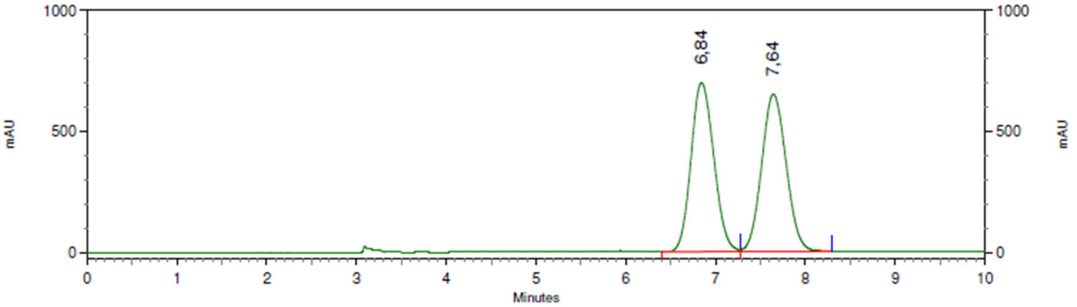

9: 225 nm, 4 nm Results

Retention Time	Area	Area Percent
6,84	49047459	49,927
7,64	49190214	50,073

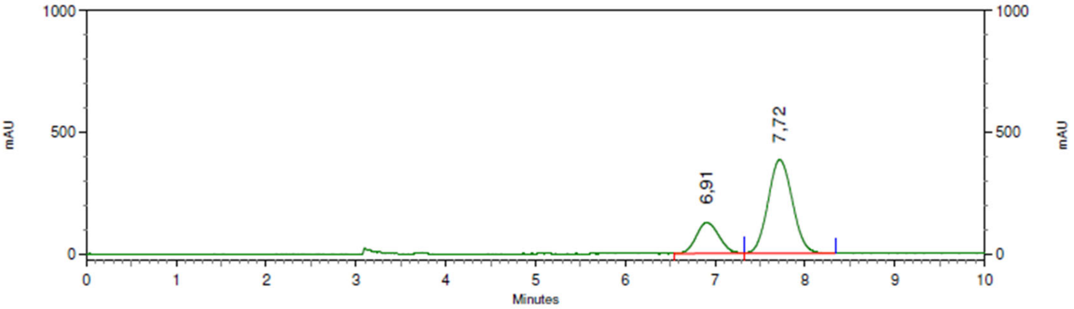

9: 225 nm, 4 nm Results

Retention Time	Area	Area Percent
6,91	8948789	23,594
7,72	28979479	76,406

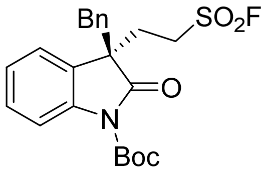

2x

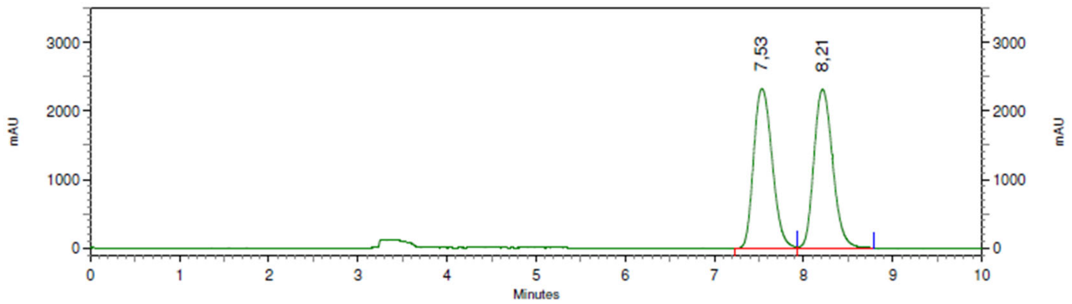

39: 203 nm, 4 nm
Results

Retention Time	Area	Area Percent
7,53	134937337	49,710
8,21	136511361	50,290

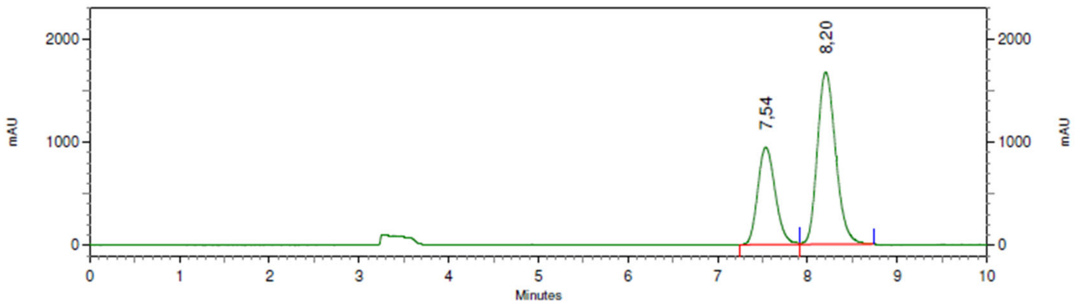

39: 203 nm, 4 nm
Results

Retention Time	Area	Area Percent
7,54	51394284	35,161
8,20	94774380	64,839

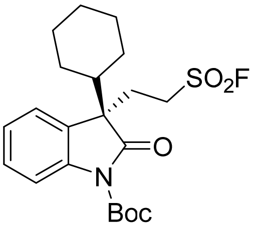

2y

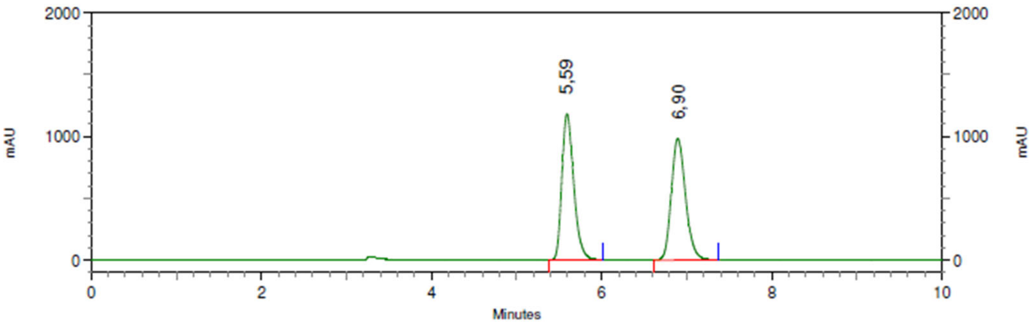

26: 226 nm, 4 nm
Results

Retention Time	Area	Area Percent
5,59	45764926	50,025
6,90	45719524	49,975

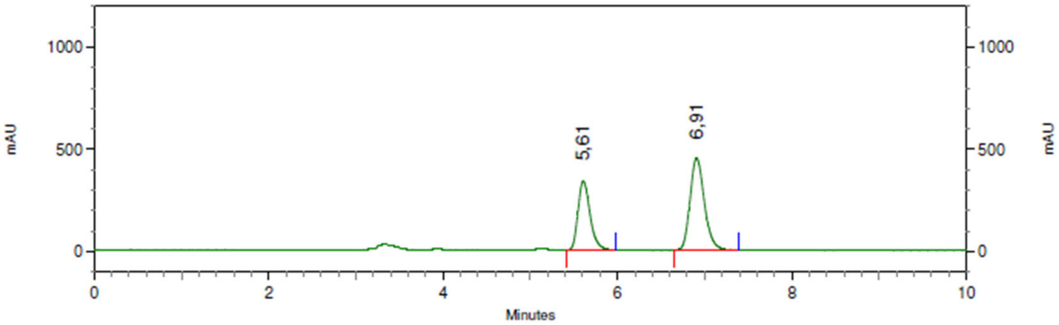

26: 226 nm, 4 nm
Results

Retention Time	Area	Area Percent
5,61	13041166	38,685
6,91	20670136	61,315

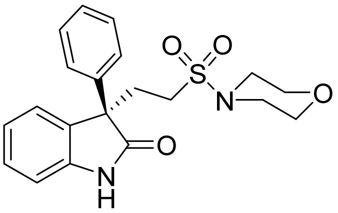

3

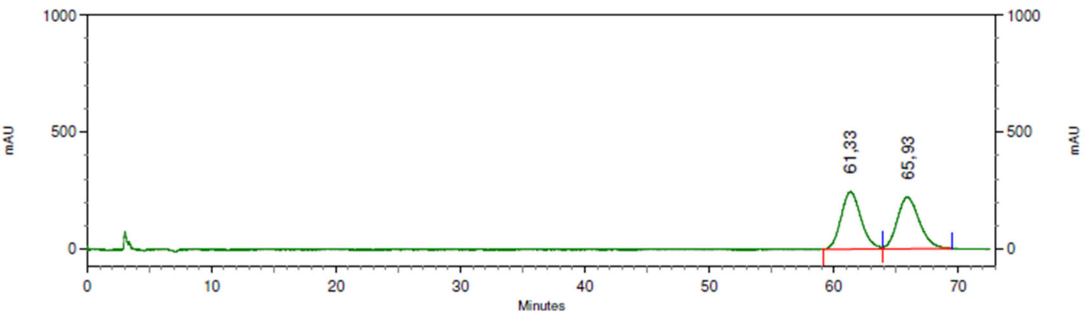

20: 206 nm, 4 nm
Results

Retention Time	Area	Area Percent
61,33	108945878	50,065
65,93	108661269	49,935

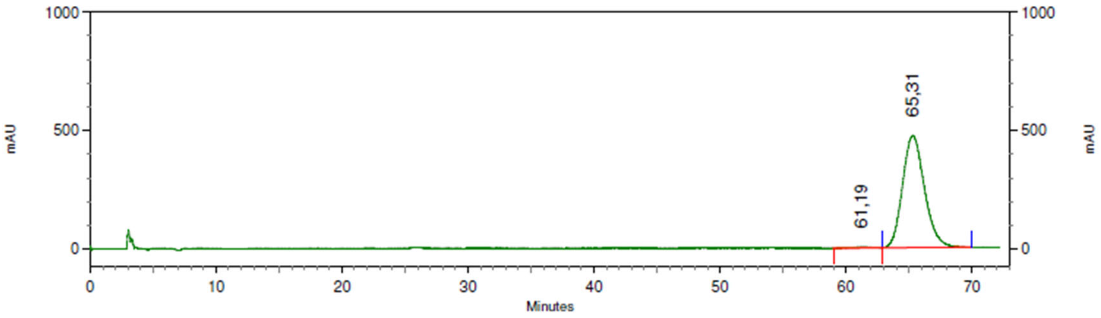

20: 206 nm, 4 nm
Results

Retention Time	Area	Area Percent
61,19	2315106	1,003
65,31	228536564	98,997

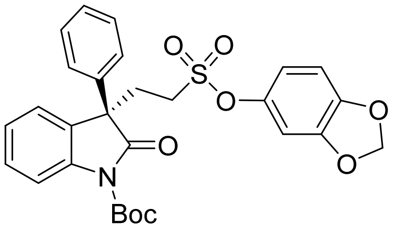

4

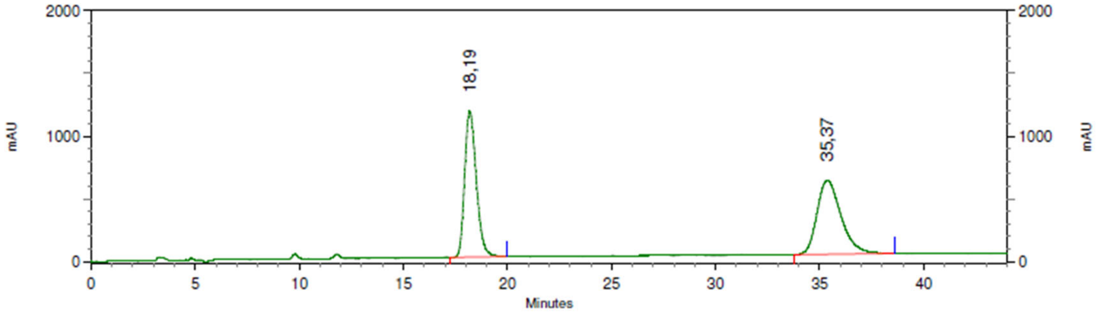

47: 203 nm, 4 nm
Results

Retention Time	Area	Area Percent
18,19	181664156	48,973
35,37	189279742	51,027

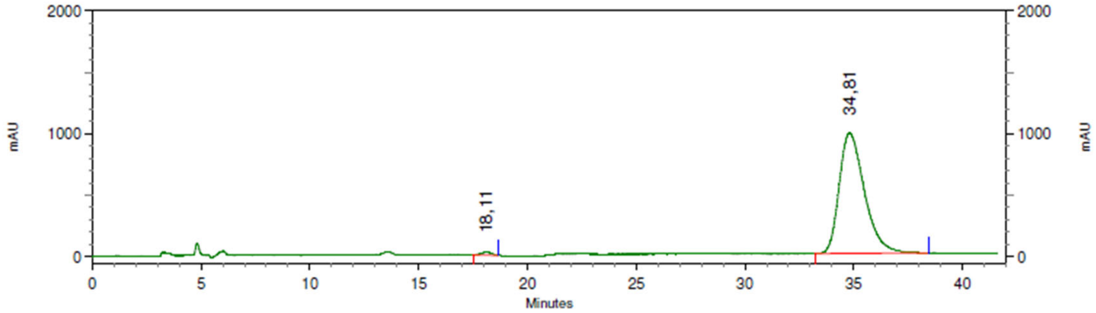

47: 203 nm, 4 nm
Results

Retention Time	Area	Area Percent
18,11	3385024	1,058
34,81	316656816	98,942

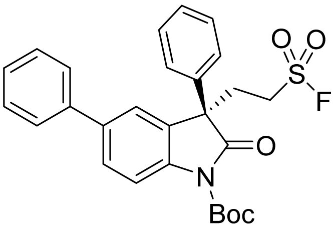

7

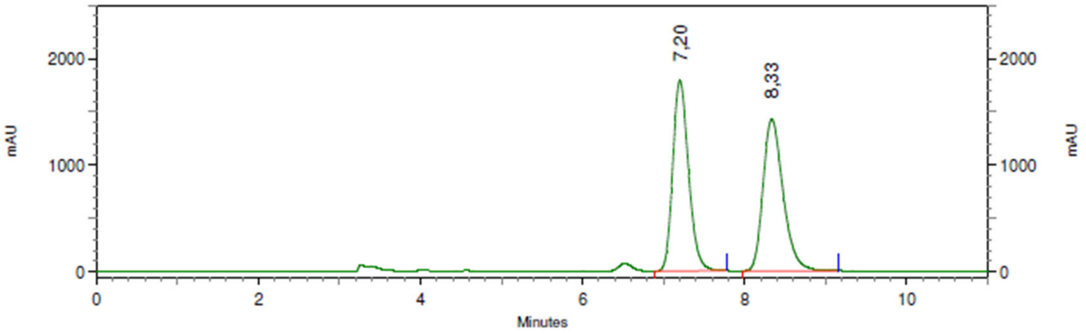

20: 206 nm, 4 nm
Results

Retention Time	Area	Area Percent
7,20	96808580	49,593
8,33	98397609	50,407

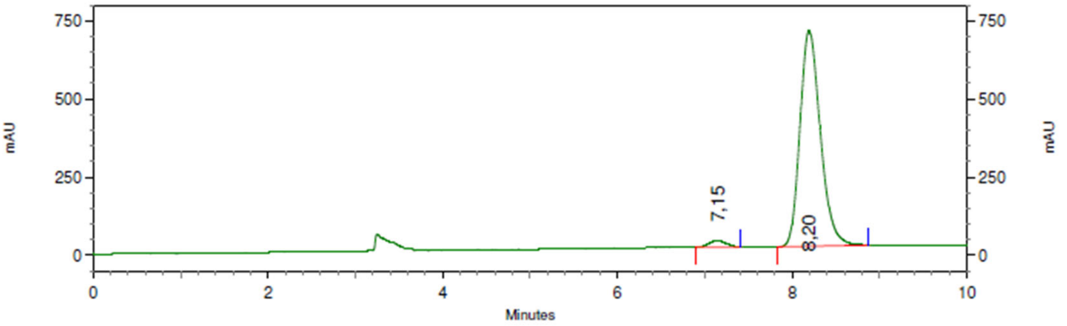

20: 206 nm, 4 nm
Results

Retention Time	Area	Area Percent
7,15	1172473	2,549
8,20	44820690	97,451

References

(1) Hamashima, Y.; Suzuki, T.; Takano, H.; Shimura, Y.; Sodeoka, M. Catalytic enantioselective fluorination of oxindoles. *J. Am. Chem. Soc.* **2005**, *127*, 10164–10165.

(2) Sharma, M.; Tamanna, N.; Chauhan, P. Asymmetric synthesis of hydrophenanthrenones bearing multiple stereogenic centers via squaramide-catalyzed domino 1,4-/1,4-addition desymmetrization sequence. *Org. Lett.* **2023**, *25*, 7911–7916.

(3) Cheng, L.; liu, L.; Wang, D.; Chen, Y.-J. Highly Enantioselective and Organocatalytic α-Amination of Oxindoles. *Org. Lett.* **2009**, *11*, 3874-3877.

(4) Durbin, M. J.; Willis, M. C. Palladium-catalyzed α-arylation of oxindoles. *Org. Lett.* **2008**, *10*, 1413–1415.

(5) Li, E.; Liao, X.; Guo, F.; Huang, Y. ; Chen, J. N-Heterocyclic Carbene-Catalyzed Asymmetric SN2 Alkylation via Noncovalent Activation. *Org. Lett.* **2024**, *26*, 7479-7483.

(6) Barroso, S.; Blay, G.; Cardona, L.; Fernández, I.; García, B.; Pedro, J. R. Highly Diastereoselective Arylation of (S)-Mandelic Acid Enolate: Enantioselective Synthesis of Substituted (*R*)-3-Hydroxy-3-phenyloxindoles and (*R*)-Benzylic Acids and Synthesis of Nitrobenzophenones. *J. Org. Chem.* **2004**, *69*, 6821.-6829.

(7) Toullec, Patrick Y.; Jagt, Richard B. C.; De Vries, Johannes G.; Feringa, Ben L.; Minnaard, Adriaan J. Rhodium-Catalyzed Addition of Arylboronic Acids to Isatins: An Entry to Diversity in 3-Aryl-3-Hydroxyoxindoles. *Org. Lett.* **2006**, *8*, 2715–2718

(8) Zhang, X. X. Wang, P. K.; Zhang, S. L. Synthesis of α-Aryl-oxindoles via Grignard Reaction with Isatin in the Presence of Diphenyl Phosphite. *Chem Asian J.* **2024**, *19*, e202400297.

(10) Yin, L.; Kanai, M.; Shibasaki, M. A Facile Pathway to Enantiomerically Enriched 3-Hydroxy-2-Oxindoles: Asymmetric Intramolecular Arylation of a-Keto AmidesCatalyzed by a Palladium–DifluorPhos Complex. *Angew. Chem. Int. Ed.* **2011**, *50*, 7620 –7623.

(9) Cai, Y.; Li, J.; Chen, W.; Xie, M.; Liu, X.; Lin, L.; Feng, X. Catalytic Asymmetric Sulfenylation of Unprotected 3-Substituted Oxindoles. *Org. Lett.* **2012**, *14*, 2726-2729.

(11) Xiao, Z.-K.; Ying, H.-Y.; Shao, L.-X. N-Heterocyclic Carbene-Palladium(II)-1-Methylimidazole Complex Catalyzed α-Arylation of Oxindoles with Aryl Chlorides and Aerobic Oxidation of the Products in a One-Pot Procedure. *Org. Lett*. **2013**, *15*, 1254-1257.

(12) Lin, J.; Jia, M.; Ma, S. Pd-Catalyzed 2,3-Allenylation of Oxindoles with 2,3-Allenylic Carbonate. Chin. *J. Chem.* **2021**, *39*, 3044—3050.

(13)] Jin, Q.-W.; Chai, Z.; Huang, Y.-M.; Zou, G.; Zhao, G. Asymmetric α-amination of 3-substituted oxindoles using chiral bifunctional phosphine catalysts. *Beilstein J. Org. Chem.* **2016,** *12,* 725–731.

(14) Ulikowski, A.; Furman, B. Schwartz's Reagent-Mediated Regiospecific Synthesis of 2,3-Disubstituted Indoles from Isatins. *Org. Lett.* **2016**, *18*, 2, 149–151.

(15) Amaya, Toru; Kurata, Izumi; Hirao, Toshikazu. Synthesis of oxindoles via reductive CO_2 fixation. *Org. Chem. Front.* **2016**, *3*, 929–933.

(16) Ma, S.; Han, X.; Krishnan, S.; Virgil, S. C.; Stolz, B. M. Catalytic Enantioselective Stereoablative Alkylation of 3-Halooxindoles: Facile Access to Oxindoles with C3 All-Carbon Quaternary Stereocenters. *Angew. Chem. Int. Ed.* **2009**, *48*, 8037 –8041.

(17) Badiola, E.; Fiser, B.; Gómez-Bengoa, E.; Mielgo, A.; Olaizola, I.; Urruzuno, I.; García, J. M.; Odriozola, J. M.; Razkin, J.; Oiarbide, M.; Palomo, C. Enantioselective construction of tetrasubstituted stereogenic carbons through Brønsted base catalyzed Michael reactions: α′-hydroxy enones as key enoate equivalent. *J. Am. Chem. Soc.* **2014**, *136*, 17869–17881.

(18) Duan, S.-W.; An, J.; Chen, J.-R.; Xiao, W.-J. Facile Synthesis of Enantioenriched Cγ-Tetrasubstituted α-Amino Acid Derivatives via an Asymmetric Nucleophilic Addition/Protonation Cascade. *Org. Lett.* **2011**, *13*, 2290–2293.

(19) Ning, P. F.; Wei, Y.; Chen, X. Y.; Yang, Y. F.; Gao, F. C.; Hong, K. A general method to access sterically encumbered geminal bis(boronates) via formal umpolung transformation of terminal diboron compounds. *Angew. Chem. Int. Ed.* **2024**, *63*, e202315232.

(20) Li, Y.; Zhu, D.-X.; Xu, M.-H. A new versatile approach to synthesise enantioenriched 3-hydroxyoxindoles, 1,3-dihydroisobenzofuran and 3-isochromanone derivatives by a rhodium-catalyzed asymmetric arylation–cyclization sequence. *Chem. Commun.* **2013**, *49*, 11659-11661.

(21) Lei, N.; Zhang, Q.; Tao, P.; Lu, C.; Lei. Q.; Zheng, K. Dearomative difunctionalization of arenes via highly selective radical relay reactions. *Org. Chem. Front.* **2024**, *11*, 4654-4662.

(22) Rajeswaran, W. G.; Cohen, L. A. Studies on protection of oxindoles. *Tetrahedron.* **1998**, *54*, 11375–11380.

(23) Mosiagin, I.; Fernandes, A. J.; Budinská, A.; Hayriyan, L.; Ylijoki, K. E. O.; Katayev, D. Catalytic *ipso*-nitration of organosilanes enabled by electrophilic *N*-nitrosaccharin reagent. *Angew. Chem. Int. Ed.* **2023**, *62*, e202310851.

(24) Barbasiewicz, M.; Mąkosza, M. Intermolecular reactions of chlorohydrine anions: acetalization of carbonyl compounds under basic conditions. *Org. Lett.* **2006**, *8*, 3745–3748.

(25) Zheng, L.; Gao, F.; Yang, C.; Gao, G. L.; Zhao, Y.; Gao, Y.; Xia, W. Visible-light-mediated anti-regioselective nitrone 1,3-dipolar cycloaddition reaction and synthesis of bisindolylmethanes. *Org. Let.t* **2017**, *19*, 5086–5089.

(26) Sofiadis, M.; Kalaitzakis, D.; Sarris, J.; Montagnon, T.; Vassilikogiannakis, G. Vinylogous reactivity of cyclic 2-enones: organocatalysed asymmetric addition to 2-enals to synthesize fused carbocycles. *Angew. Chem. Int. Ed.* **2019**, *58*, 6742–6746.

(27) Perkins, W.; Fischer, F. R. Inserting porphyrin quantum dots in bottom-up synthesized graphene nanoribbons. *Chem. Eur. J.* **2017**, *23*, 17687–17691.

(28) Silm, E.; Järving, I.; Kanger, T. Asymmetric organocatalytic Michael addition of cyclopentane-1,2-dione to alkylidene oxindole. *Beilstein J. Org. Chem.* **2022**, *18*, 167–173.

(29) Trost, B. M.; Cramer,M.; Silverman, S- M. Enantioselective Construction of Spirocyclic Oxindolic Cyclopentanes by Palladium-Catalyzed Trimethylenemethane-[3+2]-Cycloaddition.. *J. Am.Chem. Soc.* **2007** *129*, 12396-12397.

(30) Rossetti, A.; Sacchetti, A.; Bonfanti, M.; Roda, G.; Rainoldi, G.; Silvani, A.Biocatalysed olefin reduction of 3-alkylidene oxindoles by baker's yeast. *Tetrahedron* **2017**, *73*, 4584-4590.

(31) Voituriez, A.; Pinto, N.; Neel, M.; Retailleau, P.; Marinetti, A. An Organocatalytic [3+2] Cyclisation Strategy for the Highly Enantioselective Synthesis of Spirooxindoles. *Chem. Eur. J.* **2010**, *16*, 12541 – 12544.

(32) Ming, Y.-C.; Lv, X.-J.; Wang, C.; Liu, Y.-K. Diversity-Oriented Synthesis of Spirooxindoles via Asymmetric Organocatalytic Regiodivergent Cascade Reactions. *Org. Lett*. **2024**, *26*, 10853-10858)

(33) Rassu, G.; Zambrano, V.; Tanca, R.; Sartori, A.; Battistini, L.; Zanardi, F.; Curti, C.; Casiraghi, G. 3-Alkenyl-2-silyloxyindoles: An Enabling, Yet Understated Progeny of Vinylogous Carbon Nucleophiles. *Eur. J. Org. Chem*. **2012**, 466-470.

(34) Ishimaru, T, Shibata, N.; Nagai, J.; Nakamura, S.; Toru, T.; Kanemasa, S.Lewis Acid-Catalyzed Enantioselective Hydroxylation Reactions of Oxindoles and β-Keto Esters Using DBFOX Ligand *J. Am. Chem. Soc.* **2006**, *128*, 16488-16489.

(35) Chen, Z. Da; Zhou, X.; Yi, J. T.; Diao, H. J.; Chen, Q. L.; Lu, G.; Weng, J. Catalytic decarboxylative fluorosulfonylation enabled by energy-transfer-mediated photocatalysis. *Org. Lett.* **2022**, *24*, 2474–2478.

(36) Liu, C.; Yang, C.; Hwang, S.; Ferraro, S. L.; Flynn, J. P.; Niu, J. A general approach to O-sulfation by a Sulfur(IV) Fluoride Exchange reaction. *Angew. Chem. Int. Ed.* **2020**, *59*, 18435–18441.

(37) Qiu, B.; Xu, D.; Sun, Q.; Lin, J.; Sun, W. Manganese-catalyzed asymmetric oxidation of methylene C-H of spirocyclic oxindoles and dihydroquinolinones with hydrogen peroxide. *Org. Lett.* **2019**, *21*, 618–622.
